# The efficacy of virtual reality in adults during puncture biopsy: A systematic review and meta-analysis of randomized controlled trials

**DOI:** 10.1371/journal.pone.0330364

**Published:** 2025-08-26

**Authors:** Xueling Qiu, Wei Gong, Cui Li, Xiaochen Jiang, Weifeng Wang, Fan Sun, Chenxi Sun, Wenjuan Cao, Lu Tang

**Affiliations:** 1 School of Nursing, Shandong First Medical University, Tai’an, China; 2 Department of Stomatology, the 960th hospital of People’s Liberation Army of China (PLA), Jinan, China; 3 Department of the Food and Drug Inspection, Drug and Instrument Supervision and Inspection Station of Shenyang Joint Logistics Support Center, Shenyang, China; 4 Department of Rehabilitation, the 960th hospital of People’s Liberation Army of China (PLA), Jinan, China; 5 School of Nursing, Shandong Second Medical University, Weifang, China; 6 School of nursing, Shandong University of Traditional Chinese Medicine (TCM), Jinan, China; 7 School of Nursing, Jinzhou Medical University, Jinzhou, China; The Chinese University of Hong Kong, HONG KONG

## Abstract

**Background:**

Virtual reality, as a nascent technology, possesses the potential to enhance patient comfort by mitigating pain and anxiety during medical procedures. This study aimed to evaluate the efficacy of virtual reality intervention in managing pain for adults undergoing puncture biopsy procedures.

**Methods:**

From the outset of the development of databases until October 8, 2024, a comprehensive search of the published literature was performed across eight electronic databases. The collected data was consolidated and subjected to meta-analysis by using RevMan 5.4. The quality of the inclusion of randomized controlled trials in terms of methodological quality was assessed by the Cochrane Risk of Bias Assessment Tool. Additionally, strength and certainty of the evidence was assessed by the GRADE system.

**Results:**

The study was conducted on data from a sample of 445 patients from six randomized controlled trials. The pooled analysis revealed that the virtual reality group demonstrated an analgesic effect (MD = −1.61, 95% CI: −2.54 to −0.68; *p* = 0.0007; *I*^*2 *^= 90%). Moreover, the virtual reality intervention was found to reduce patient anxiety during puncture biopsy procedures (MD = −9.49, 95% CI −14.47 to −4.50; *p* = 0.0002; *I*^*2 *^= 88%). In addition, three studies that could not be included in the meta-analysis also reported a positive impact of increasing patient satisfaction with the operation.

**Conclusions:**

Virtual reality can be used as an analgesic method in adult puncture biopsies and as a reliable alternative therapy in clinical settings, providing a valuable non-pharmacologic approach to pain management. Nevertheless, the level of evidence is relatively low, thus further high-quality studies are required to substantiate our conclusion.

**Systematic review registration:**

PROSPERO database, CRD4202459303.

## Introduction

Puncture biopsy represents the primary method of acquiring a tumor tissue or cell sample for histopathologic diagnosis [1,2]. It can provide important clinical significance and value for early diagnosis and treatment of diseases. Nevertheless, puncture biopsy frequently gives rise to pain. A total of 58 patients (95%) experienced local pain during thyroid fine-needle aspiration, as revealed by a recent study [3]. Similarly, between 40% and 64% of patients reported experiencing moderate to severe pain during bone marrow biopsy, and more than 20% of patients expressed dissatisfaction with the pain control measures employed [4–6]. A study of prostate puncture biopsy revealed that 20% of biopsy events were classified as severe in terms of pain, and with 10% of these events requiring the use of analgesics [7]. Consequently, pain has constituted one of the most significant challenges associated with puncture biopsy procedures, necessitating prompt and effective resolution.

To date, the majority of puncture biopsies have been conducted with the patient under local anesthesia [8–11]. Nonetheless, a number of studies have shown that local anesthesia is an insufficient analgesic during puncture biopsy [12,13]. Moreover, analgesic and sedative medications, including opioids, benzodiazepines, and nitrous oxide, are commonly administered to alleviate pain [14,15]. However, these medications do not provide complete pain relief during the procedure or alleviate the anxiety associated with subsequent recall of the pain, meanwhile, they can precipitate adverse effects, as well as prolonged hospitalization time [16–18]. This may impact patients’ willingness to adhere to the procedure, in addition to their future diagnosis and treatment of the disease. Furthermore, some patients who require regular biopsies are reluctant to undergo re-biopsy due to a resistance to pain, which may result in the failure to detect early lesions and thus delay the patients' condition.

The notion of pain management being a fundamental human right has become increasingly prominent recently, attracting significant public interest [19]. Ensuring adequate pain management for optimal patient compliance is critical, which may ultimately affect patient satisfaction and acceptability of re-biopsy. Nowadays, the advent and evolution of non-pharmacological treatments have resulted in the growing utilization of various non-pharmacological therapies in the field of pain management. A substantial body of evidence from previous studies indicated that non-pharmacological techniques, such as music, acupuncture, and hypnosis, appear to be a method that can relieve pain during puncture biopsy [20–22]. Nevertheless, implementation may necessitate specialized training for interventionists, which may be constrained in some healthcare settings [23]. Concurrently, in the contemporary epoch of accelerated technological advancement, it is possible that these traditional non-pharmacological methods may be unable to fulfill the demands and aspirations of patients. Given the limitations of earlier non-pharmacological interventions, the emergence of new and sophisticated technologies, offers a promising approach to the management of intraoperative pain.

Virtual reality (VR) is the synthesization of an artificial, computer-generated environment that is designed to supplant the patients’ sensory input derived from the real world [24]. Unlike numerous painkillers, VR has the potential to disrupt the C-fiber pathway, which transmits signals to the central nervous system that are perceived as painful. This disruption may be influenced by factors such as attention, concentration, and mood changes [25]. The initial description of the utilization of VR in pain was published in 2000, during the process of changing dressings for patients with acute burns. The preliminary findings of the study indicated that immersive VR may represent a promising method for the management of severe aches and pains, warranting further investigation [26]. The utilization of immersive VR as an adjunct to therapeutic interventions has increased in recent years, coinciding with a reduction in the cost of VR systems [27]. The efficacy of VR has been substantiated in a multitude of procedures, including those conducted in pediatric settings, in the context of burns, dressing changes, dentistry, labor and delivery, and hysteroscopy, etc [28–33].

To date, numerous researches have been conducted with the objective of evaluating the efficacy of VR for adults undergoing puncture biopsy procedures. Nevertheless, the efficiency of VR in pain and distress relief remains a point of contention in published trials. Some clinical trials have demonstrated the efficacy of VR in mitigating pain and anxiety during puncture biopsy. Korkmaz et al. found that the viewing of VRG video streams led to a reduction in discomfort and pain experienced by patients during bone marrow puncture and biopsy [26]. In a similar vein, the efficacy and practicality of VR are comparable to those of prostate puncture biopsy [32]. However, Prabhu et al. found that no significant difference was observed in pain scores during breast puncture biopsy in the VR intervention group compared to the standard care control group [33]. Consequently, the extant clinical findings are inadequate to substantiate the analgesic effect of VR in puncture biopsy in adults. In light of the current evidence, it is not possible to suggest that VR is an effective therapeutic modality for pain management during puncture biopsy in adults. Therefore, the objective of our study was to synthesize evidence to assess the analgesic effectiveness of VR during puncture biopsy for adults.

## Objective

The objective of our systematic review and meta-analysis is to synthesize the results of randomized controlled trials investigating the efficacy of VR in reducing pain and anxiety during puncture biopsy in adults. The authors hypothesized that VR intervention would be effective in relieving pain and anxiety during puncture biopsy in adults.

## Methods

### Registration

In order to enhance the clarity and rigor of this study, the Preferred Reporting Items for Systematic Reviews and Meta-Analyses (PRISMA) guidelines have been meticulously adhered to (S1 Table). Furthermore, the study has been registered on the Prospero (CRD42024539303) [34].

### Eligibility criteria

The selection strategy was based on the PICOS:

(a) Participants: patients over the age of 18 who underwent puncture biopsy procedures and receiving VR therapy; (b) Intervention: VR, and there were no restrictions on the general characteristics of VR; (c) Control: comparison with other interventions (e.g., analgesic medicine, other methods of distraction, no intervention); (d) Outcome: pain level and anxiety level associated with puncture biopsy; (e) Study designs: only randomized controlled trials.

### Information sources

In accordance with the established research protocol, the published literature was searched from the inception of the databases until 8 October 2024. The literature was then re-searched and updated in electronic databases, including PubMed, Web of Science, Cochrane Library, Scopus, EMBASE, Chinese National Knowledge Infrastructure (CNKI), Wan-fang Data, and Chinese Biomedical Database (CBM). Furthermore, a comprehensive review of the references for each study was conducted.

### Search strategy

In order to ensure the most comprehensive search results, relevant medical subject headings and free words were used in the search. The following search terms were employed: (“Virtual reality” or VR) and (“Biopsy, Needle” or “Puncture Biopsy” or Puncture or Biopsy). The search strategies may be adapted according to the specific requirements of the desired database. The search strategies are outlined in S2 Table.

### Selection process

The literature search was conducted independently by two authors. The actual electronic searches were completed between October 9 and October 16, 2024. The results were downloaded to Endnote X9, where they went through to a deduplication process. The study screening process was achieved through a two-step process. The initial stage of the literature review entailed the exclusion of studies based on the evaluation of their titles and abstracts, which were deemed to be inconsistent with the established inclusion criteria. Two authors (XLQ and WFW) then undertook a comprehensive review of the full studies in order to identify those that met the pre-established inclusion criteria. In instances where a discrepancy regarding the selection was observed, another author was requested to arbitrate the dispute (S3 Table).

### Data collection process

The data extracted included the following information about each study: authors’ names, publication year, and country; demographics (sample size, average age, sex, and type of procedure); type of VR; study design; adjunctive analgesics; outcomes. Where available, the data were obtained from the text and tables by means of a systematic process of extraction. Two authors (XLQ and WFW) completed the data extraction process in an independent manner. The study data was managed using an Excel spreadsheet that could be easily analyzed and reconciled (S4 Table).

### Outcomes

#### Primary outcome.

The primary outcome was the level of pain experienced by patients undergoing puncture biopsy, encompassing both discomfort during the procedure and pain associated with the biopsy itself. The primary outcome was expressed using the mean of a visual analogue scale (VAS). Transformations were applied, when necessary, for example, when expressing pain as a mean of 0–100 or median.

#### Secondary outcome.

The secondary outcome was the level of anxiety, which was assessed using the State Trait Anxiety Inventory (STAI). It consisted of two separate 4-point Likert-type scales, the Trait Anxiety Inventory (STAI-T) and the State Anxiety Inventory (STAI-S), ranging from 1 (no anxiety) to 4 (complete anxiety), each consisting of 20 items [35]. Total scores obtain from the scales range from 20 to 80, with higher scores indicating higher levels of anxiety. The STAI-S assesses the subject’s current state of anxiety, whereas the STAI-T evaluates the subject’s relatively stable aspects [35].

### Study risk of bias assessment

The authors (XLQ and WFW) used the Cochrane risk of bias assessment tool to evaluate the risk of bias. The quality of the methodology employed in each study was evaluated according to six primary criteria. The third author (XCJ) conducted a review of the unresolved disagreements. Subsequently, the data were imported into the Review Manager software in order to create the risk-of-bias plots.

### Assessment of the quality of evidence

Two authors (XLQ and WFW) conducted a comprehensive assessment of the strength of evidence pertaining to all outcomes. The level of the evidence was evaluated in accordance with the GRADE methodology [36].

### Data analysis

The data were subjected to analysis using RevMan 5.4. In the instance of continuous variables, the mean difference (MD) is employed as a means of representation. Interval estimates are shown with 95% confidence intervals (CI). The presence of statistical heterogeneity among the studies was evaluated using the *I*^*2*^. In instances where significant heterogeneity was identified, random effects model was employed, subgroup analysis was performed to elucidate the potential heterogeneity across the studies. Subgroup analysis based on comparison of type of procedure (bone marrow biopsy, prostate biopsy, breast biopsy), sex (male or female), and local anesthesia (with or without). The sensitivity analysis was needed for evaluation of stability and reliability of results. The findings of the meta-analysis were evaluated based on their clinical and statistical significance.

## Results

### Search and selection

**Fig 1:**
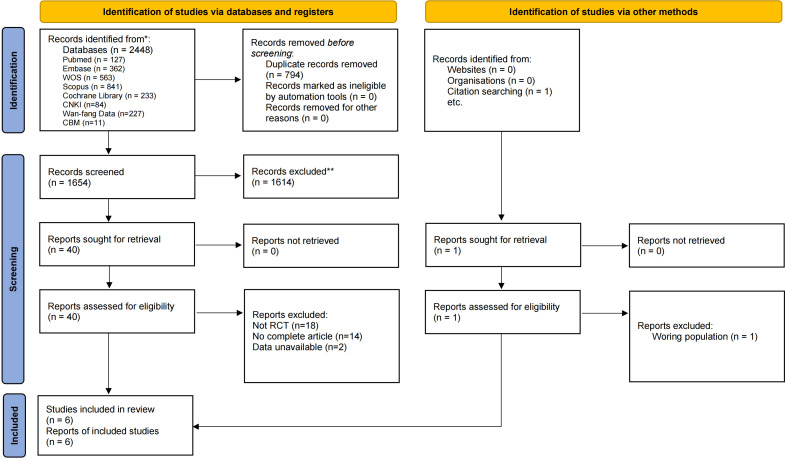
Flowchart of the study selection.

2,448 records were identified from eight electronic databases for subsequent analysis. After the removal of duplicates, 1,654 studies remained, and 40 studies remained after screening by title and abstract. In addition, a search of the references revealed one study that met our inclusion criteria. A full-text search was conducted on the 41 studies that met the aforementioned criteria. Ultimately, the six studies were ultimately incorporated into the final quantitative analysis [37–42]. The particular process was showed in Fig 1.

### Characteristics

#### Study characteristics.

All six studies were performed in the English language. The studies were conducted in Europe (France, Turkey), and America (USA). The studies were published between 2021 and 2024, and included 445 participants, with 219 participants in the intervention group and 226 participants in the control group. In the intervention group, all studies used VR; In the control group, one study [37] used MEOPA, five studies [38–42] had standard treatment or care. In addition, three studies [37,38,42] had adjunctive use of local anesthesia in the intervention and control groups, all with lidocaine (Table 1).

**Table 1:** Summary of the included studies.

Study	Study design	Patients(I/C)	Sex(M/F)	Age(I/C)	Operation type	Intervention	Control	Adjuvant analgesics	Outcomes
Le Du et al. 2023 [37] France	RCT	58/60	64/54	66 (38-87) 66 (18-87)	Marrow Biopsy	**Device:** VR, GearVR head-mounted **Scenarios:** Nohara, Kaitei, Uchuu, and Mori	MEOPA	Lidocaine	**Pain** VAS **Anxiety** STAI
Korkmaz et al. 2023 [38] Turkey	RCT	35/40	40/35	50.18 ± 16.26 49.86 ± 15.63	Marrow Biopsy	**Device:** VR, VR goggles **Scenarios:** Underwater, museum, park, and hiking image	Standard procedure	10ml of 2% lidocaine solution	**Pain** VAS **Anxiety** STAI-S
Genç et al. 2022 [39] Turkey	RCT	32/32	64	70.59 ± 7.92 73.12 ± 8.217	Prostate Biopsy	**Device:** VR, VR glasses **Scenarios:** Nature video scenes	Standard procedure	None	**Pain** VAS **Vital signs**
Toraman et al. 2024 [40] Turkey	RCT	35/35	70	64.11 ± 5.34 64.31 ± 4.52	Prostate Biopsy	**Device:** VR, VR Glasses **Scenarios:** Parks, nature,seaside walks, undersea views, and museum tours	The standard care	None	**Pain** VAS **Anxiety** STAI-S STAI-T **Satisfaction**
Karaman et al. 2021 [41] Turkey	RCT	30/30	60	46.9 ± 11.2 41.4 ± 12.9	Breast Biopsy	**Device:** VR, VR Box Glasses **Scenarios:** A walk on the beach	Standard procedure	None	**Pain** VAS **Anxiety** STAI-S
Prabhu et al. 2023 [42] US	RCT	29/29	58	51.1 ± 14.7 56.0 ± 14.9	Breast Biopsy	**Device:** VR, Oculus Go Standalone VR headset **Scenarios:** The beach environment	The standard care	A buffered solution of 1% lidocaine	**Pain** VAS **Anxiety** STAI **Satisfaction**

RCT: randomized controlled trial; VR: virtual reality; MEOPA: equimolar mixture of oxygen and nitrous oxide; VAS: visual analogue scale; STAI: state-trait anxiety inventory; STAI-S: state-trait anxiety inventory-state; STAI-T: state-trait anxiety inventory-trait.

#### Participant characteristics.

Two studies [37,38] recruited participants of both sexes and there were more male participants (104) than female participants (75). Two studies [39,40] included only male participants and two studies [41,42] included only female participants. The main types of procedures included bone marrow biopsy [37,38], prostate biopsy [39,40], and breast biopsy [41,42].

### Quality and certainty of evidence assessment

**Fig 2:**
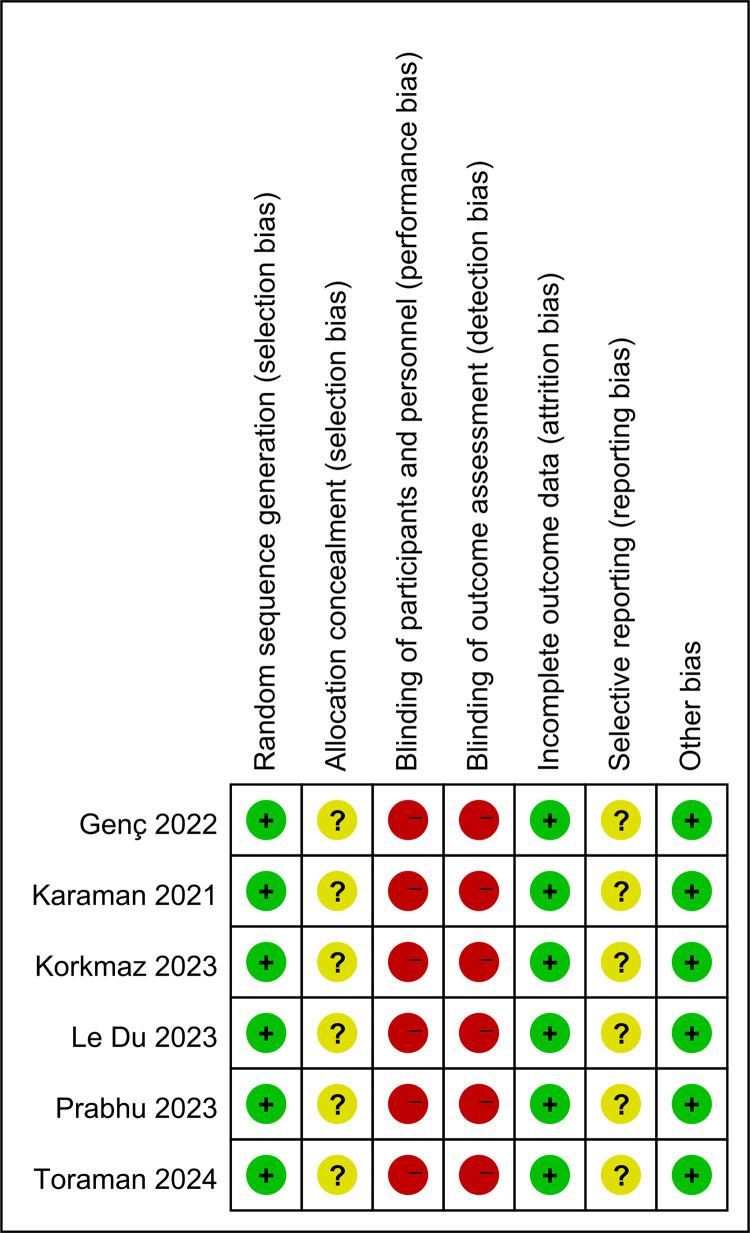
Risk of bias summary (Green represents a low risk of bias, yellow some concerns, and red a high risk of bias).

We assessed the risk of bias for the six studies included in this review (Fig 2). In terms of randomization sequence generation, all studies were low risk. In terms of allocation concealment, all six included randomized controlled trials showed an unclear risk of bias. This was judged to be unclear due to the lack of sufficient information on allocation concealment in the original studies. Regarding blinding (participants, personnel and outcome assessment), all six randomized controlled trials showed a high risk of bias. Blinding of patients or personnel during VR applications is very difficult. Therefore, blinding of patients or personnel was not achieved in any of the included studies. As for incomplete outcome data, all randomized controlled trials included a low risk regarding risk. In regard to selective reporting, all six studies demonstrated an unclear risk of bias, as the available information regarding the registration network was incomplete, a detailed comparison of the content of the article and the registration program to assess the risk could not be conducted. In regard to other potential sources of bias, all six randomized controlled trials were deemed to be at low risk. In conclusion, all studies exhibited some concerns regarding the overall risk (S5 Table).

### Outcomes

The primary outcome was assessed using the VAS to quantify the level of pain experienced by participants. The tool utilized a 10-point scale, with 0 indicating no pain and 10 representing the worst imaginable pain. For secondary outcomes, anxiety level was assessed in five studies. Five studies [37,38,40–42] used the STAI. Two studies [37,42] expressed as STAI total scores, two studies [38,41] as scores on a subscale: STAI-S only, and one study [40] as scores on both subscales: STAI-S and STAI-T.

#### Pain.

**Fig 3:**
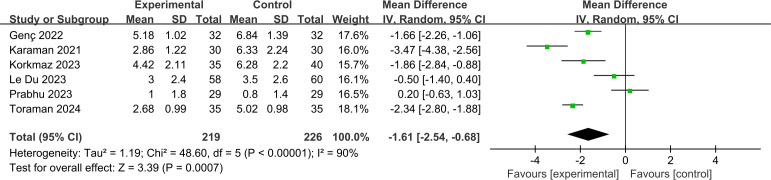
Forest plot for pain score.

The six studies evaluated the efficacy of the VR group in comparison to the control group on pain, with a total sample size of 445. We used a random effects model to estimate the effects in summary. The random effects models demonstrated that the VR intervention resulted in a statistically significant reduction in pain intensity when compared with the control group (MD = −1.61, 95% CI: −2.54 to −0.68; *p* = 0.0007; *I*^*2 *^= 90%) (Fig 3). We used an iterative method to exclude studies one by one and observe the change in heterogeneity. We found a significant decrease in heterogeneity (I²from 90% to 77%) after excluding two studies (Prabhu and Karaman), and analyzed that the decrease in heterogeneity may be related to breast biopsies, which are inherently less painful. After excluding them, the point estimate was −1.65 with a confidence interval [−2.38, −0.91]. The conclusions did not change and remained statistically different. This suggested that the two studies may be drivers of heterogeneity, but a high level of heterogeneity still exists.

#### Anxiety.

**Fig 4:**
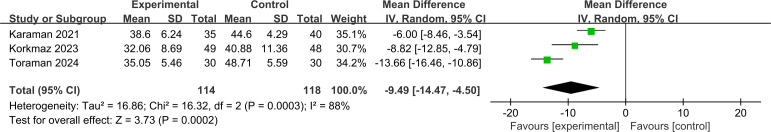
Forest plot for anxiety score.

Three studies were conducted to investigate the impact of VR on state anxiety score [38,40,41]. These trials involved a total of 232 participants. The overall effect showed a significant VR effect with reduced state anxiety score compared to the control group (MD = −9.49, 95% CI −14.47 to −4.50; *p* = 0.0002) (Fig 4). However, the pooled studies exhibited heterogeneity under a random-effects model (*I*^*2*^* *= 88%).

### Subgroup analysis

Given the significant heterogeneity observed, subgroup analyses of pain score were performed based on comparison of type of procedures, sex, and local anesthesia.

#### Types of procedures.

**Fig 5:**
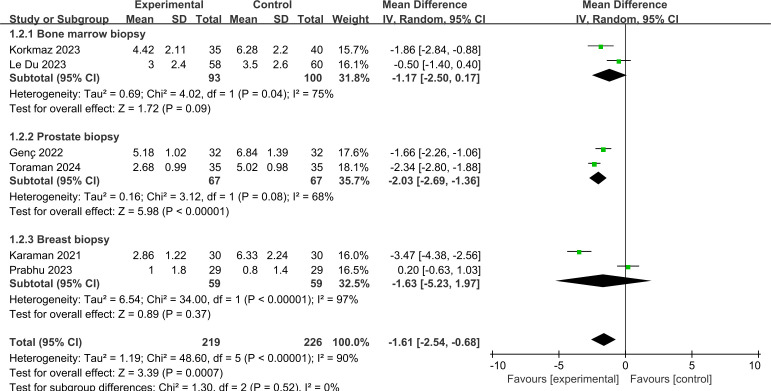
Forest plot of subgroup analysis for pain score by different type of procedures.

Fig 5 demonstrates the pain score for different types of procedures. It demonstrated that prostate biopsy resulted in a difference in pain intensity when compared to other procedure types (MD = −2.03, 95% CI: −2.69 to −1.36; p < 0.00001).

#### Sex.

**Fig 6:**
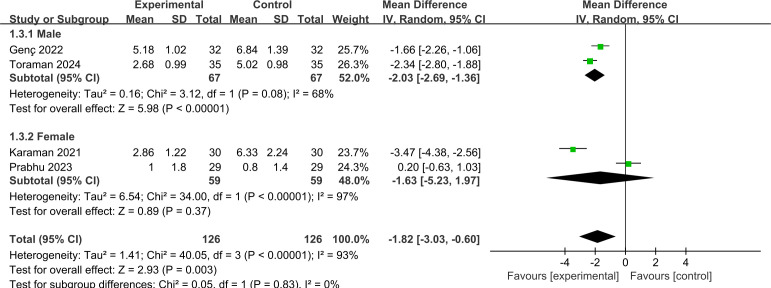
Forest plot of subgroup analysis for pain score by sex.

Fig 6 demonstrates the pain score for different sex. In the subgroup analysis, four studies were included in the investigation of sex [39–42]. The findings indicated that the intensity of pain was markedly diminished in the male cohort (MD = −2.03, 95% CI: −2.69 to −1.36; p < 0.00001; *I*^*2*^ = 68%). However, no statistically significant difference was observed in the female group (MD = −1.63, 95% CI: −5.23to 1.97; *p* = 0.37; *I*^2^ = 94%).

#### Local anesthesia.

**Fig 7:**
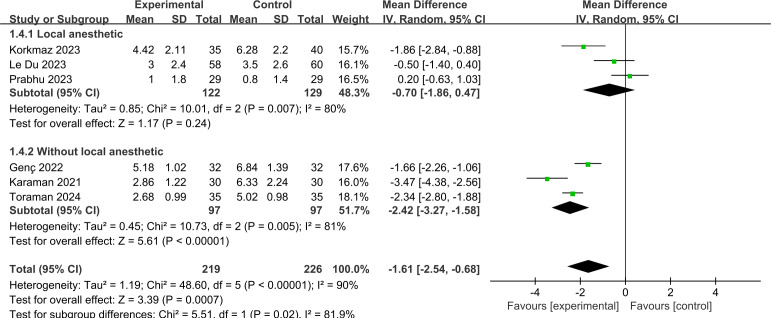
Forest plot of subgroup analysis for pain score by local anesthesia.

Fig 7 demonstrates the pain score with and without the use of local anesthesia. A reduction in pain was observed in the subgroup that did not receive local anesthesia (MD = −2.42, 95% CI: −3.27 to −1.58; *p* < 0.00001; *I*^*2*^ = 81%), whereas no statistically significant improvement in pain was evident in the subgroup that did receive local anesthesia (MD = −0.70, 95% CI: −1.86 to 0.47; *p* = 0.24; *I*^*2*^ = 80%).

### GRADE evaluation of the outcomes

All outcomes were assessed in accordance with GRADE criteria. Both the pain score and the anxiety score were of low quality, indicating that the results were of limited confidence and that differences between the actual effects and the results were more likely to be presented (S6 Table). This may be due to the increased risk of bias, imprecision and high heterogeneity in the included studies.

### Publication bias

Detected by the Egger’s test (*p* = 0.519), suggesting that there did not appear to be statistically significant publication bias in these studies (S1 File).

## Discussion

### Analysis of results

The analysis of the data yielded the conclusion that the VR intervention resulted in a reduction of pain and anxiety levels experienced by adult participants during puncture biopsies, in comparison to the levels reported by the control group. Therefore, VR can be utilized as an analgesic method during puncture biopsy, offering a valuable non-pharmacological approach to pain management.

The results of this study demonstrated that the utilization of VR technology led to a statistically reduction in pain. This suggested that VR may provide analgesia for adults during puncture biopsy. A number of theories have been proposed regarding the mechanisms by which VR alters pain perception. One such theory, put forth by Gold et al., suggested that VR analgesia is elicited by inter-cortical modulation in the pain substrate signaling pathway through attention, emotion, memory, and other senses (e.g., tactile, auditory, and visual), ultimately leading to analgesia [43]. Furthermore, Li et al. postulated that frontal/pain inhibitory pathways, pivotal neural gating mechanisms, and/or additional neurochemical processes may be initiated during VR, resulting in pain reduction, enhanced top-down inhibition, and potential VR analgesia [44]. Currently, the underlying neurobiological mechanisms of VR remain somewhat enigmatic. Moreover, our subgroup analysis found that the analgesic effect of VR seemed to be more pronounced for the prostate puncture biopsy in male. This may be attributable to the observation that men and women may exhibit disparate responses to non-pharmacological interventions [45]. Females showed greater pain sensitivity, stronger pain facilitation and lower pain suppression compared to males [45]. Therefore, further research is needed to investigate whether sex is one of the factors influencing the analgesic effect of VR. In addition, we found that the analgesic effect of VR was not effective when standard local anesthesia was used in both groups. Prabhu and colleagues put forth the hypothesis that VR interventions may prove to be comparatively ineffectual in mitigating pain when the pain in question has already reached a relatively minimal level [42]. Therefore, VR may be considered an effective alternative or adjunct when other analgesic methods or standard local anesthesia are not suitable for puncture biopsy or do not provide adequate analgesia. In light of these findings, the integration of VR into clinical pain management practices holds significant clinical implications.

The aggregated results of three studies demonstrated that VR reduced patients’ anxiety level, which was statistically distinct from those of the control group. Given the identical evaluation metrics and the restricted number of studies, subgroup analysis of anxiety scores was not conducted. The source of anxiety of patients may be related to a lack of understanding of various issues, including preoperative preparation and postoperative care, potential complications during the procedure, and the temporal uncertainty of the outcome and the fear of an unknown result, which causes anxiety in most patients. Furthermore, research has indicated that those with increased anxiety levels are more likely to perceive pain as intense during invasive procedures [15]. Pain and anxiety have a reciprocal relationship, with one affecting the other: high anxiety levels lead to increased sensitivity to pain, while the experience of pain itself can also result in elevated anxiety levels [46]. Consequently, while anxiety is reduced, pain during the procedure is simultaneously reduced. It is therefore imperative that we provide patients with comprehensive preoperative and postoperative education, monitor their psychological state closely, and deliver high-quality humanistic care.

Patient satisfaction is a key indicator of qualitative aspects of an nurses’ healthcare provision. It can be defined as an evaluation of the healthcare services received by an individual, and is shaped by the patient’s expectations and the outcomes of those services [47]. The findings of three studies indicate that the utilization of VR can enhance patient satisfaction during puncture biopsy procedures [37,40,42]. A number of factors may contribute to the level of satisfaction experienced by patients, including the patients’ past experience, pain threshold, age, sex or procedural experience of healthcare professionals. The study conducted by Espinoza et al. concluded that the utilization of VR glasses in the context of oncology hospitalization could potentially lead to an enhancement in patient satisfaction [48]. The positive effects of relaxation and natural imagery and sounds during biopsy procedures, patient-nurse communication, accessibility, empathy, symptom management and treatment practices, and pain and anxiety reduction were all identified as factors influencing patient satisfaction [40]. It is therefore recommended that greater attention be paid to the psychological feelings of the patients during the surgical process, with a view to improving the patients’ surgical satisfaction and thus increasing the degree of acceptance and compliance with the procedure. However, a quantitative analysis of the satisfaction results was not possible due to the unavailability of the requisite data. Therefore, future studies should emphasize the assessment of patient and healthcare worker satisfaction to present results by obtaining objective data with uniform measurement tools to facilitate more reliable conclusions.

Nevertheless, a considerable degree of heterogeneity was still observed across the included studies. We performed subgroup analyses of patients by type of procedure, sex, and local anesthesia, and although heterogeneity was reduced to some extent, heterogeneity remained relatively high. Therefore, heterogeneity of the studies may be attributed to other factors, such as the use of different VR (differences of clarity, processor speed, and fit), the time of intervention or experience of clinician operation. Given the limited number of included studies and the lack of clarity in the description of some of them, and the failure to adequately characterize the specific features of the VR devices, it was not possible to perform subgroup analyses of these factors. Our findings suggested that the VR intervention may be more effective in certain subgroups. However, these results were exploratory and warrant further validation in future studies.

### Clinical implications

Virtual reality technology is a non-pharmacological analgesic treatment that is safer and less physically and psychologically harmful to patients than pharmacological analgesia. It has no significant side effects and is more acceptable to patients. Moreover, in comparison with alternative non-pharmacological interventions, this approach is more expeditious and convenient, as it does not necessitate professional training. As an emerging high technology, it is more efficacious for treatment, as it captures patients’ attention and engenders interest. The pain experienced during puncture biopsy is typically acute and brief. In such cases, the utilization of VR technology offers several advantages: VR technology is convenient to use, does not require too much preparation in advance, can be used by wearing it during the procedure, and can be taken off at the end of the surgery, which is practical. Furthermore, it can be adapted to suit the diverse requirements of patients’ video content, thereby enhancing their satisfaction and acceptance. This approach is designed to address the individual needs of each patient, fostering improved treatment cooperation. Consequently, VR is a viable non-pharmacological analgesic method for adults undergoing puncture biopsy.

### Implications for future research

A plethora of issues must be given due consideration and addressed in future research endeavors. Firstly, there is a necessity for a greater number of high-quality randomized controlled trials in order to provide the evidence required for the development of evidence-based medicine. Furthermore, the issue of high heterogeneity may be attributable to numerous factors. These may include variations in the technical equipment of the intervention, the duration or frequency of the intervention. Subsequent studies may have the opportunity to perform subgroup analyses through the use of standardized intervention procedures in combination with the use of uniform intervention devices, or a clearer description of their characteristics and duration of intervention. This could potentially reduce methodological heterogeneity and thus ensure the reliability of the study. Moreover, in order to guarantee the success and security of a VR implementation, it is imperative to take into account a number of crucial factors. Firstly, it is crucial to acknowledge that the utilization of VR may potentially elicit certain adverse effects, including dizziness, blurred vision, and headaches [49]. However, these effects are typically transient and can be alleviated through rest. It is therefore essential to control the duration of immersion in virtual reality and to enhance the monitoring of the patient’s performance during the procedure. It is recommended that future research focus on the practical applications, with the objective of determining the optimal time for VR intervention, with the goal of producing the best physical and mental effects while avoiding patient fatigue and side effects. Secondly, uniform standards and standardized application protocols for VR should be established in the clinic [50]. Such protocols should include standard procedures for VR use, indications and contraindications, time limits for use, and maintenance of the equipment. The establishment of such protocols would facilitate clinical promotion and standardized application by healthcare professionals. Furthermore, virtual content designed specifically for patients undergoing puncture biopsy can be created to address their psychological needs from their own perspective, thereby facilitating the achievement of optimal pain and anxiety relief. Concurrently, objective observation indicators, such as patients’ vital signs, satisfaction, acceptance, and so forth, were incorporated into the assessment process to gain a more comprehensive understanding of patients’ analgesia-related needs and to guarantee patient safety.

### Limitations and strengths

This study is subject to several limitations. Firstly, some of the included randomized controlled trials lacked sufficient detail regarding the methods of randomization and blinding. This may have introduced a significant risk of bias, which could potentially impact the reliability of the conclusions drawn from the results. Secondly, outcome indicators included in the analysis was restricted to those pertaining to pain and anxiety. It would be advantageous for future studies to incorporate a greater number of outcome indicators, thus enhancing the reliability of the findings. Thirdly, the point in time at which data were collected varied depending on the studies included in the analysis. In addition, the VR devices as well as the timing of the interventions were inconsistent across studies. Differences in analgesic effects produced by different VR devices, such as immersive headset versus headset-only substitution, may have been present. The duration of the intervention may also contribute to differences in analgesic effects, again one of the factors that may influence analgesia that needs to be investigated in the future. All of these conditions may have contributed to the high degree of heterogeneity observed, thus reducing the robustness of our results. Consequently, the inclusion of additional high-quality studies in the future is essential to strengthen the reliability of the findings. The current meta-analysis also has certain advantages. Firstly, the exclusive utilization of RCTs guaranteed the consistency of data across all analytical procedures, thereby enhancing the reliability of the findings. Secondly, the impact of VR was evaluated by incorporating patient cohorts with disparate forms of puncture biopsies. This approach represents a novel method of pain management during puncture biopsy procedures, with significant clinical implications and value.

## Conclusion

With respect to the available studies, VR modestly reduced pain and anxiety during puncture biopsy in adults compared with controls, and VR has the potential to be an analgesic method for adults undergoing puncture biopsy. However, the results of this study could not be confirmed due to the inclusion of poor-quality studies and insufficient evidence. Therefore, in order to achieve this goal, we suggest that further high-quality trials are necessary.

## Supporting information

S1 Table PRISMA 2020 checklist. (DOCX)

S2 Table Search strategies. (DOCX)

S3 Table Table of all studies. (DOCX)

S4 Table All data extracted from the primary research sources. (XLSX)

S5 Table Quality and certainty of evidence assessment. (DOCX)

S6 Table Summary of GRADE evidence profile. (DOCX)

S1 File Egger’s test. (DOCX)
