# Supplementary material for: The efficacy of virtual reality in adults during puncture biopsy: A systematic review and meta-analysis of randomized controlled trials
Source: PLoS One. 2025 Aug 26;20(8):e0330364. doi: 10.1371/journal.pone.0330364 (PMC12380292; doi:10.1371/journal.pone.0330364)
Supplement: S2 Table — (DOCX) [file pone.0330364.s002.docx]

**S2 Table. Search Strategies**

| Database | Search number | Search terms |
| --- | --- | --- |
| Pubmed | #1 | Virtual reality [MeSH Terms] |
|  | #2 | Virtual reality exposure therapy[MeSH Terms] |
|  | #3 | (Virtual reality immersion therapy OR Virtual reality therapy OR VR)[Title/Abstract] |
|  | #4 | #1 OR #2 OR #3 |
|  | #5 | Biopsy, Needle[MeSH Terms] |
|  | #6 | (Puncture Biopsy OR Puncture OR Biopsy)[Title/Abstract] |
|  | #7 | #5 OR #6 |
|  | #8 | #4 AND #7 |
| Web of Science | #1 | TS=(virtual reality) OR TS=(VR) OR TS=(Virtual reality exposure therapy) OR TS=(Virtual reality immersion therapy) OR TS=(Virtual reality therapy) |
|  | #2 | (((TS=(Biopsy, Needle)) OR TS=(Puncture Biopsy)) OR TS=(Puncture)) OR TS=(Biopsy ) |
|  | #3 | #1 AND #2 |
| EMBASE | #1 | 'virtual reality'/exp |
|  | #2 | 'virtual reality exposure therapy'/exp |
|  | #3 | #1 OR #2 |
|  | #4 | 'needle biopsy'/exp |
|  | #5 | 'puncture'/exp |
|  | #6 | 'biopsy'/exp |
|  | #7 | #4 OR #5 OR #6 |
|  | #8 | #3 AND #7 |
| SCOPUS | #1 | (TITLE-ABS-KEY("virtual reality") OR TITLE-ABS-KEY("virtual reality exposure therapy") OR TITLE-ABS-KEY("virtual reality immersion therapy") OR TITLE-ABS-KEY ("virtual reality therapy") OR TITLE-ABS-KEY(VR)) |
|  | #2 | ( TITLE-ABS-KEY ( "biopsy, needle" ) OR  TITLE-ABS-KEY ( "puncture biopsy" ) OR TITLE-ABS-KEY ( puncture ) OR TITLE-ABS-KEY ( biopsy ) ) |
|  | #3 | #1 AND #2 |
| Cochrane Library | #1 | (virtual reality) OR (VR) OR (virtual reality exposure therapy) OR (virtual reality immersion therapy) OR (virtual reality therapy) (Word variations have been searched) |
|  | #2 | (puncture biopsy) OR (puncture) OR (biopsy) OR (biopsy, needle) (Word variations have been searched) |
|  | #3 | #1 AND #2 |
| Chinese National Knowledge Infrastructure (CNKI) | #1 | Topic: Virtual Reality OR Virtual Reality Technology OR VR |
|  | #2 | Topic: Puncture OR Biopsy OR Puncture Biopsy OR Puncture Biopsy Procedure OR Needle Puncture |
|  | #3 | #1 AND #2 |
| Wan-fang Data | #1 | Topic: Virtual Reality OR Virtual Reality Technology OR VR |
|  | #2 | Topic: Puncture OR Biopsy OR Puncture Biopsy OR Puncture Biopsy Procedure OR Needle Puncture |
|  | #3 | #1 AND #2 |
| Chinese Biomedical Database (CBM) | #1 | (“Virtual Reality” [Title: Smart] OR “Virtual Reality Technology” [Title: Smart] OR “VR” [Title: Smart]) AND (“Puncture” [Title: Smart] OR ‘Biopsy’ [Title: Smart] OR “Puncture Biopsy” [Title: Smart] OR “Puncture Biopsy Technique” [Title: Smart] OR “Needle Puncture” [Title: Smart]) |
