# Supplementary material for: The efficacy of virtual reality in adults during puncture biopsy: A systematic review and meta-analysis of randomized controlled trials
Source: PLoS One. 2025 Aug 26;20(8):e0330364. doi: 10.1371/journal.pone.0330364 (PMC12380292; doi:10.1371/journal.pone.0330364)
Supplement: S3 Table — (DOCX) [file pone.0330364.s003.docx]

**S3 Table. Table of all studies**

|  | Author | Year | Study | Reason for exclusion |
| --- | --- | --- | --- | --- |
| 1 | \ | 2000 | Paper presented at the Proceedings of SPIE | Title-abstract exclusion |
| 2 | \ | 2001 | Proceedings of SPIE: Biomarkers and Biological Spectral Imaging | Title-abstract exclusion |
| 3 | \ | 2001 | 27th Biennial mechanisms and robotics conference: Part B | Title-abstract exclusion |
| 4 | \ | 2002 | 2013 ICME International Conference on Complex Medical Engineering, CME 2013 | Title-abstract exclusion |
| 5 | \ | 2014 | 6th International Symposium on Biomedical Simulation, ISBMS 2014 | Title-abstract exclusion |
| 6 | \ | 2013 | 2013 2nd International Conference on Sensors, Measurement and Intelligent Materials, ICSMIM 2013 | Title-abstract exclusion |
| 7 | \ | 2015 | Science & Technology Information | Title-abstract exclusion |
| 8 | \ | 2017 | Proceedings - IEEE Symposium on Computer-Based Medical Systems | Title-abstract exclusion |
| 9 | \ | 2017 | 7th International Workshop on Computational Kinematics, CK 2017 | Title-abstract exclusion |
| 10 | \ | 2018 | High-tech medicine comes to life | Title-abstract exclusion |
| 11 | \ | 2019 | Virtual reality reduces pain from needles | Title-abstract exclusion |
| 12 | \ | 2020 | International Conference on Computer Vision and Graphics, ICCVG 2020 | Title-abstract exclusion |
| 13 | \ | 2019 | Annual International Conference on 3D Imaging Technology, IC3DIT 2019 | Title-abstract exclusion |
| 14 | \ | 2020 | Application of virtual reality in patient explanation of magnetic resonance imaging–ultrasound fusion prostate biopsy | Title-abstract exclusion |
| 15 | \ | 2022 | Pig-to-human transplants take a leap toward reality | Title-abstract exclusion |
| 16 | \ | 2022 | 2022 8th International Conference on Virtual Reality, ICVR 2022 | Title-abstract exclusion |
| 17 | \ | 2022 | Abstracts of the 32nd Annual Meeting of the Italian Society of Uro-Oncology, SIUrO | Title-abstract exclusion |
| 18 | \ | 2024 | International Workshop on New Approaches for Multidimensional Signal Processing, NAMSP 2023 | Title-abstract exclusion |
| 19 | Aadam, A. A.Wani, S.  Amick, A.Shah, J. N.  Bhat, Y. M.Hamerski, C. M.Klapman, J. B.  Muthusamy, V. R.Watson, R. R.Rademaker, A. W.et al., | 2016 | A randomized controlled cross-over trial and cost analysis comparing endoscopic ultrasound fine needle aspiration and fine needle biopsy | Title-abstract exclusion |
| 20 | Abdelkhalik, M.  Boueri, M.  Nasr, L.  Khater, C. | 2024 | A Narrative Review: Transforming Pediatric Oncology Care Through Virtual Reality-Pain Management and Enhanced Patient Experience | Title-abstract exclusion |
| 21 | Abebe, B. T.  Weiss, M.  Modess, C.  Roustom, T.  Tadken, T.  Wegner, D.  Schwantes, U.  Neumeister, C.  Schulz, H.  Scheuch, E.  et al., | 2019 | Effects of the P‐Glycoprotein Inhibitor Clarithromycin on the Pharmacokinetics of Intravenous and Oral Trospium Chloride: a 4‐Way Crossover Drug‐Drug Interaction Study in Healthy Subjects | Title-abstract exclusion |
| 22 | Actrn, | 2020 | Integration of Virtual Reality as analgesia for Trans-rectal Ultrasound prostate biopsy (TRUS): a randomized prospective study | Trial registry record |
| 23 | Adachi, T.  Machida, H.  Nishikawa, M.  Arai, T.  Kariyasu, T.  Koyanagi, M.  Yokoyama, K. | 2020 | Improved delineation of CT virtual bronchoscopy by ultrahigh-resolution CT: comparison among different reconstruction parameters | Title-abstract exclusion |
| 24 | Aggarwal, R. | 2017 | Just-in-time simulation-based training | Title-abstract exclusion |
| 25 | Aghayev, E.  Thali, M. J.  Sonnenschein, M.  Jackowski, C.  Dirnhofer, R.  Vock, P. | 2007 | Post-mortem tissue sampling using computed tomography guidance | Title-abstract exclusion |
| 26 | Ahad, Md A. R.  Hossain, S. | 2004 | Augmented reality and its challenges | Title-abstract exclusion |
| 27 | Ahn, S. Y.  Park, C. M.  Yoon, S. H.  Kim, H.  Goo, J. M. | 2019 | Learning curve of C-arm cone-beam computed tomography virtual navigation-guided percutaneous transthoracic needle biopsy | Title-abstract exclusion |
| 28 | Alaraj, Ali  Charbel, Fady T.  Birk, Daniel  Tobin, Mathew  Luciano, Cristian  Banerjee, Pat P.  Rizzi, Silvio  Sorenson, Jeff  Foley, Kevin  Slavin, Konstantin  Roitberg, Ben | 2013 | Role of Cranial and Spinal Virtual and Augmented Reality Simulation Using Immersive Touch Modules in Neurosurgical Training | Title-abstract exclusion |
| 29 | Albuquerque, J. V. D.  Andriolo, B. N. G.  Vasconcellos, M. R. A.  Civile, V. T.  Lyddiatt, A.  Trevisani, V. F. M. | 2019 | Interventions for morphea | Title-abstract exclusion |
| 30 | Ali, Saad  Qandeel, Monther  Ramakrishna, Rishi  Yang, Carina W. | 2018 | Virtual Simulation in Enhancing Procedural Training for Fluoroscopy- guided Lumbar Puncture: A Pilot Study | Title-abstract exclusion |
| 31 | Allison, S. L.  Fagan, A. M.  Morris, J. C.  Head, D. | 2016 | Spatial Navigation in Preclinical Alzheimer's Disease | Title-abstract exclusion |
| 32 | al-Nahhas, A. M.  Kedar, R.  Morgan, S. H.  Landells, W. N.  al-Murrani, B.  Heary, T.  Wright, A.  Cosgrove, D. O.  Bending, M. R.  McCready, V. R. | **1993** | Cellular versus vascular rejection in transplant kidneys. Correlation of radionuclide and Doppler studies with histology | Title-abstract exclusion |
| 33 | Alonso-Felipe, Miguel  Aguiar-Perez, Javier Manuel  Perez-Juarez, Maria Angeles  Baladron, Carlos  Peral-Oliveira, Julio  Amat-Santos, Ignacio J. | 2023 | Application of Mixed Reality to Ultrasound-guided Femoral Arterial Cannulation During Real-time Practice in Cardiac Interventions | Title-abstract exclusion |
| 34 | Alpsoy, Anil  Adanir, Haydar  Bayramoglu, Zeynep  Elpek, Gulsum Ozlem | 2022 | Correlation of hepatitis B surface antigen expression with clinicopathological and biochemical parameters in liver biopsies: A comprehensive study | Title-abstract exclusion |
| 35 | Álvarez-Mancha, A. I.  Mancha-Doblas, I.  Molina-Vega, M.  Fernández-García, D.  Gómez-Pérez, A. M.  Gallego-Domínguez, E.  Ortega-Jiménez, M. V.  Hierro-Martín, I.  Tinahones, F. J. | 2024 | Evolutionary analysis of indeterminate cytology and risk of malignancy in a thyroid nodule unit | Title-abstract exclusion |
| 36 | Amiras, D.  Hurkxkens, T. J.  Figueroa, D.  Pratt, P. J.  Pitrola, B.  Watura, C.  Rostampour, S.  Shimshon, G. J.  Hamady, M. | 2021 | Augmented reality simulator for CT-guided interventions | Title-abstract exclusion |
| 37 | Andersen, N. L.  Jensen, R. O.  Posth, S.  Laursen, C. B.  Jørgensen, R.  Graumann, O. | 2021 | Teaching ultrasound-guided peripheral venous catheter placement through immersive virtual reality An explorative pilot study | Title-abstract exclusion |
| 38 | Anderson, N. | 2024 | Commentary: The effect of virtual reality distraction on haemodialysis patients’ pain and anxiety during arteriovenous fistula puncture: a randomised controlled trial | Title-abstract exclusion |
| 39 | Angulo, P.  Larson, D. R.  Therneau, T. M.  LaRusso, N. F.  Batts, K. P.  Lindor, K. D. | 1999 | Time course of histological progression in primary sclerosing cholangitis | Title-abstract exclusion |
| 40 | Aragao, M. F. V. V.  Oliveira, A. D. P.  Lima, A. R. M. C.  Leal, M. C.  Valenca, M. M. | 2021 | Virtual Biopsy: A Reality Thanks to Advances in Radiology | Title-abstract exclusion |
| 41 | Arase, Y.  Ikeda, K.  Tsubota, A.  Suzuki, Y.  Saitoh, S.  Kobayashi, M.  Akuta, N.  Someya, T.  Kobayashi, M.  Suzuki, F.  Kumada, H. | 2003 | Randomized trial of prolonged interferon retreatment for chronic hepatitis C patients with HCV-genotype 1b and high virus load | Title-abstract exclusion |
| 42 | Arikatla, V. S.  Sankaranarayanan, G.  De, S. | 2011 | Cost-efficient suturing simulation with pre-computed models | Title-abstract exclusion |
| 43 | Arjomandi Rad, A.  Subbiah Ponniah, H.  Shah, V.  Nanchahal, S.  Vardanyan, R.  Miller, G.  Malawana, J. | 2023 | Leading Transformation in Medical Education Through Extended Reality | Title-abstract exclusion |
| 44 | Arora, D.  Tewari, P.  Shamshery, C.  Chandra, B.  Gupta, A.  Pal, L. | 2024 | 3D Virtual Bronchoscopy as an Aid to Airway Management in a Patient with Anterior Mediastinal Mass | Title-abstract exclusion |
| 45 | Artico, Simara  Amaral, Karine Medeiros  Treter Goncalves, Candice Beatriz  Picon, Paulo Dornelles | 2012 | The effectiveness of retreatment with peginterferon alfa and ribavirin in patients with chronic viral hepatitis C genotype 2 and 3: a prospective cohort study in Brazil | Title-abstract exclusion |
| 46 | Arulesan, V.  Srimathveeravalli, G.  Kesavadas, T.  Nagathan, P.  Baier, R. E.  Arya, S.  Dwivedi, A.  Mulla, Z. D.  Plavsic, S. K. | 2007 | Data acquisition and development of a trocar insertion simulator using synthetic tissue models | Title-abstract exclusion |
| 47 | Arya, S.  Dwivedi, A.  Mulla, Z. D.  Plavsic, S. K. | 2017 | Effectiveness of ultrasound simulation in obstetrics and gynecology education: A state-of-the-Art review | Title-abstract exclusion |
| 48 | Asahina, H.  Yamazaki, K.  Onodera, Y.  Kikuchi, E.  Shinagawa, N.  Asano, F.  Nishimura, M. | 2005 | Transbronchial biopsy using endobronchial ultrasonography with a guide sheath and virtual bronchoscopic navigation | Title-abstract exclusion |
| 49 | Asano, F. | 2010 | Virtual Bronchoscopic Navigation | Title-abstract exclusion |
| 50 | Asano, F. | 2016 | Advanced bronchoscopy for the diagnosis of peripheral pulmonary lesions | Title-abstract exclusion |
| 51 | Asano, F. | 2016 | Recommendation of Combination of Radial Endobronchial Ultrasound with Virtual Bronchoscopic Navigation | Title-abstract exclusion |
| 52 | Asano, F. | 2018 | Does virtual bronchoscopic navigation improve the diagnostic yield of transbronchial biopsy? | Title-abstract exclusion |
| 53 | Asano, F.  Matsuno, Y.  Shinagawa, N.  Yamazaki, K.  Suzuki, T.  Ishida, T.  Moriya, H. | 2006 | A virtual bronchoscopic navigation system for pulmonary peripheral lesions | Title-abstract exclusion |
| 54 | Asgar-Deen, David  Carriere, Jay  Wiebe, Ericka  Peiris, Lashan  Duha, Aalo  Tavakoli, Mahdi | 2020 | Augmented Reality Guided Needle Biopsy of Soft Tissue: A Pilot Study | Title-abstract exclusion |
| 55 | Ashraf, S. F.  Lau, K. K. W. | 2019 | Navigation bronchoscopy: A new tool for pulmonary infections | Title-abstract exclusion |
| 56 | Atzori, B.  Vagnoli, L.  Graziani, D.  Hoffman, H. G.  Sampaio, M.  Alhalabi, W.  Messeri, A.  Lauro-Grotto, R. | 2022 | An Exploratory Study on the Effectiveness of Virtual Reality Analgesia for Children and Adolescents with Kidney Diseases Undergoing Venipuncture | Title-abstract exclusion |
| 57 | Atallah, Sam B.  DuBose, Arielle C.  Burke, John P.  Nassif, George  deBeche-Adams, Teresa  Frering, Taylor  Albert, Matthew R.  Monson, John R. T. | 2017 | Uptake of Transanal Total Mesorectal Excision in North America: Initial Assessment of a Structured Training Program and the Experience of Delegate Surgeons | Title-abstract exclusion |
| 58 | Awan, O. A. | 2023 | Simulation in Medical Education: Why We Need It | Title-abstract exclusion |
| 59 | Azagury, D. E.  Dua, M. M.  Barrese, J. C.  Henderson, J. M.  Buchs, N. C.  Ris, F. | 2015 | Image-guided surgery | Title-abstract exclusion |
| 60 | Azar, F. S.  Metaxas, D. N.  Schnall, M. D. | 2001 | A deformable finite element model of the breast for predicting mechanical deformations under external perturbations | Title-abstract exclusion |
| 61 | Azargoshasb, Samaneh  Molenaar, Lennert  Rosiello, Giuseppe | 2022 | Advancing intraoperative magnetic tracing using 3D freehand magnetic particle imaging | Title-abstract exclusion |
| 62 | Azzam, Nahla  Khamis, Nehal  Almadi, Majid  Batwa, Faisal  Alsohaibani, Fahad | 2020 | Development and validation of metric-based-training to proficiency simulation curriculum for upper gastrointestinal endoscopy using a novel assessment checklist | Title-abstract exclusion |
| 63 | Bainbridge, D. | 2010 | The use of ultrasound to guide interventions: From bench to bedside and back again | Title-abstract exclusion |
| 64 | Baker, S. D.  Heideman, R. L.  Crom, W. R.  Kuttesch, J. F.  Gajjar, A.  Stewart, C. F. | **1996** | Cerebrospinal fluid pharmacokinetics and penetration of continuous infusion topotecan in children with central nervous system tumors | Title-abstract exclusion |
| 65 | Ball, E.  Rivas, C.  Khan, R. | 2022 | If virtual gynecology clinics are here to stay, we need to include everyone | Title-abstract exclusion |
| 66 | Barba Teba, R.  López Arsuaga, L.  Firket, L.  Ferreira, F.  Moest, W.  Stoneman, S.  Georgopoulou, G. A.  Bratsiakou, A.  Gallieni, M. | 2024 | Vascular access hands-on training for young nephrologists: The fellows’ experience of the N-PATH project REVAC module | Title-abstract exclusion |
| 67 | Barisoni, L.  Hodgin, J. B. | 2017 | Digital pathology in nephrology clinical trials, research, and pathology practice | Title-abstract exclusion |
| 68 | Barone, Biagio  De Luca, Luigi  Napolitano, Luigi  Reccia, Pasquale  Crocetto, Felice  Caputo, Vincenzo Francesco  Del Biondo, Dario  Napodano, Giorgio | 2022 | COMPARISON BETWEEN THE EFFECTS OF MUSIC AND VIRTUAL REALITY ON PERCEIVED PAIN DURING TRANSRECTAL BIOPSY | Meeting Abstract |
| 69 | Barouk, J. | 2001 | Tomographie par cohérence optique et endobrachyœsophage: La réalité virtuelle? | Title-abstract exclusion |
| 70 | Battista, G.  Sassi, C.  Schiavina, R.  Franceschelli, A.  Baglivo, E.  Martorana, G.  Canini, R. | 2009 | Computerized tomography virtual endoscopy in evaluation of upper urinary tract tumors: Initial experience | Title-abstract exclusion |
| 71 | Bauer, J. J.  Zeng, J.  Zhang, W.  McLeod, D. G.  Sesterhenn, I. A.  Connelly, R. R.  Mun, S. K.  Moul, J. W. | 2000 | 3-D computer visualization and interactive prostate biopsy simulation leads to an improved systematic technique for the detection of prostate cancer: Clinical correlation | Title-abstract exclusion |
| 72 | Bebic, Zvonimir  Thomas, James Joseph | 2020 | Use of virtual reality for epidural placement in an adolescent with ischemic priapism | Title-abstract exclusion |
| 73 | Beck, S.  Honan, B.  Mallows, J. L.  Ting, J. | 2022 | From Other Journals | Title-abstract exclusion |
| 74 | Becker, H. D. | 2010 | Bronchoscopy: The Past, the Present, and the Future | Title-abstract exclusion |
| 75 | Bělohlávek, J.  Kuchynka, P.  Machala, L.  Dytrych, V.  Vítková, I.  Schramlová, J.  Šmíd, O.  Gandalovičová, J.  Linhart, A. | 2009 | Successfully resuscitated sudden cardiac death in a young homosexual male with HIV myocarditis | Title-abstract exclusion |
| 76 | Benyahia, S.  Van Nguyen, D.  Chellali, A.  Otmane, S. | 2015 | Designing the user interface of a virtual needle insertion trainer | Title-abstract exclusion |
| 77 | Beqari, J.  Hurd, J.  Masaki, F.  Tfayli, B.  Kharroubi, H.  Naito, M.  King, F.  Colson, Y. | 2024 | Assessing the accuracy of a multisection robotic bronchoscope prototype in localization and targeting of small pulmonary lesions | Title-abstract exclusion |
| 78 | Berger-Richardson, D.  Kurashima, Y.  Kaneva, P.  Feldman, L. S.  Fried, G. M.  Vassiliou, M. C. | 2012 | Models for training and evaluation of flexible endoscopic skills using the step program | Title-abstract exclusion |
| 79 | Berliere, M.  Piette, N.  Gerday, A.  Roelants, F.  Docquier, M. A.  Piette, P.  Duhoux, F.  Watremez, C. | 2019 | Mechanisms involved in hypnosis analgesia explaining potential benefits observed among breast cancer patients undergoing breast surgery | Title-abstract exclusion |
| 80 | Berni, Martina | 2018 | Haptic Surgical Guidance for Prostate Biopsy | Title-abstract exclusion |
| 81 | Best, L. M. J.  Rawji, V.  Pereira, S. P.  Davidson, B. R.  Gurusamy, K. S. | 2017 | Imaging modalities for characterising focal pancreatic lesions | Title-abstract exclusion |
| 82 | Bettati, Patric  Chalian, Majid  Huang, James  Dormer, James D.  Shahedi, Maysam  Fei, Baowei | 2020 | Augmented Reality-Assisted Biopsy of Soft Tissue Lesions | Title-abstract exclusion |
| 83 | Bettati, Patric  Dormer, James D.  Shahedi, Maysam  Fei, Baowei | 2022 | An Augmented Reality-Assisted Visualization System for Potential Applications in Prostate Biopsy | Title-abstract exclusion |
| 84 | Bettati, P.  Fei, B. | 2023 | An Augmented Reality System with Advanced User Interfaces for Image-Guided Intervention Applications | Title-abstract exclusion |
| 85 | Bettati, Patric  Young, Jeff  Rathgeb, Armand  Nawawithan, Nati  Gahan, Jeffrey  Johnson, Brett  Aspenleiter, Ryan  Browne, Fintan  Chaudhari, Aditi  Guin, Aditya  Sikand, Varin  Webb, Grant  Sherey, Jeremy  Shammet, Alsadiq  Fei, Baowei | 2024 | An augmented reality-guided biopsy system using a high-speed motion tracking and real-time registration platform | Title-abstract exclusion |
| 86 | Beulque, Emilie  Cortese, Sophie  Mastronicola, Romina  Dolivet, Gilles | 2021 | Surgical de-escalation for head and neck cancer surgery | Title-abstract exclusion |
| 87 | Bexson, C.  Oldham, G.  Wray, J. | 2024 | Safety of virtual reality use in children: a systematic review | Title-abstract exclusion |
| 88 | Bhagat, Y. A.  Rajapakse, C. S.  Magland, J. F.  Love, J. H.  Wright, A. C.  Wald, M. J.  Song, H. K.  Wehrli, F. W. | 2011 | Performance of μmRI-Based virtual bone biopsy for structural and mechanical analysis at the distal tibia at 7T field strength | Title-abstract exclusion |
| 89 | Biglari, E.  Feng, M.  Quarles, J.  Sako, E.  Calhoon, J.  Rodriguez, R.  Feng, Y. | 2015 | Haptics-enabled surgical training system with guidance using deep learning | Title-abstract exclusion |
| 90 | Bin Shahzad, Umer  Aiman, Ume  Ahmed, Muhammad | 2024 | Pioneering comfort in kidney biopsies: the role of hypnosis, virtual reality, and artificial intelligence | Title-abstract exclusion |
| 91 | Birnie, K. A.  Noel, M.  Chambers, C. T.  Uman, L. S.  Parker, J. A. | 2018 | Psychological interventions for needle‐related procedural pain and distress in children and adolescents | Title-abstract exclusion |
| 92 | Birth, M.  Iblher, P.  Hildebrand, P.  Nolde, J.  Bruch, H. P. | 2003 | Ultrasound-guided interventions using magnetic field navigation. First experiences with ultra-guide 2000™ under operative conditions | Title-abstract exclusion |
| 93 | Biscaro, Helton H.  Nunes, Fatima L. S.  Oliveira, Jessica dos Santos  Pereira, Gustavo R. | 2016 | Comparing efficient data structures to represent geometric models for three-dimensional virtual medical training | Title-abstract exclusion |
| 94 | Bisschops, R.  East, J. E.  Hassan, C.  Hazewinkel, Y.  Kamiński, M. F.  Neumann, H.  Pellisé, M.  Antonelli, G.  Bustamante Balen, M.  Coron, E.  Cortas, G.  Iacucci, M.  Yuichi, M.  Longcroft-Wheaton, G.  Pilonis, N.  Puig, I.  Van Hooft, J. E.  Dekker, E. | 2019 | Advanced imaging for detection and differentiation of colorectal neoplasia: European Society of Gastrointestinal Endoscopy (ESGE) Guideline - Update 2019 | Title-abstract exclusion |
| 95 | Bittner, J.  Tiemann, D.  Declue, A.  Awad, M. M. | 2012 | Reality vs. virtual reality-a comparison of colonoscopy simulators | Title-abstract exclusion |
| 96 | Blanco, P. J.  Holliman, R. P.  Ceballos, P. L.  Farnam, J. L. | 2019 | Exploring the Impact of Child-Centered Play Therapy on Academic Achievement of At-Risk Kindergarten Students | Title-abstract exclusion |
| 97 | Blatt, J. E.  Breese, R. O. | 2020 | In Reply to the Letter to the Editor Regarding “Tactile Skill-Based Neurosurgical Simulators Are Effective and Inexpensive” | Title-abstract exclusion |
| 98 | Blezek, D. J.  Robb, R. A. | **1997** | Evaluating virtual endoscopy for clinical use | Title-abstract exclusion |
| 99 | Blomqvist, G.  Ullman, K.  Segall, T.  Hauzenberger, E.  Renström, L.  Persson-Waller, K.  Leijon, M.  Valarcher, J. F. | 2018 | An unusual presentation of pseudocowpox associated with an outbreak of pustular ulcerative vulvovaginitis in a Swedish dairy herd | Title-abstract exclusion |
| 100 | Bloom, M. B.  Rawn, C. L.  Salzberg, A. D.  Krummel, T. M. | 2003 | Virtual reality applied to procedural testing: The next era | Title-abstract exclusion |
| 101 | Blößer, S.  May, A.  Welsch, L.  Ast, M.  Braun, S.  Velten, T.  Biehl, M.  Tschammer, J.  Roeb, E.  Knabe, M. | 2022 | Virtual Biopsy by Electrical Impedance Spectroscopy in Barrett’s Carcinoma | Title-abstract exclusion |
| 102 | Bluemel, C.  Matthies, P.  Herrmann, K.  Povoski, S. P. | 2016 | 3D scintigraphic imaging and navigation in radioguided surgery: Freehand SPECT technology and its clinical applications | Title-abstract exclusion |
| 103 | Boisvert, J.  Poirier, G.  Borgeat, L.  Godin, G. | 2013 | Real-time blood circulation and bleeding model for surgical training | Title-abstract exclusion |
| 104 | Boonstra, J. G.  Van Der Pijl, J. W.  Smets, Y. F. C.  Lemkes, H. H. P. J.  Ringers, J.  Van Es, L. A.  Van Der Woude, F. J.  Bruijn, J. A. | **1997** | Interstitial and vascular pancreas rejection in relation to graft survival | Title-abstract exclusion |
| 105 | Bordoni, V.  Bibas, M.  Viola, D.  Sacchi, A.  Cimini, E.  Tumino, N.  Casetti, R.  Amendola, A.  Ammassari, A.  Agrati, C.  Martini, F. | 2017 | Bone Marrow CD34+ Progenitor Cells from HIV-Infected Patients Show an Impaired T Cell Differentiation Potential Related to Proinflammatory Cytokines | Title-abstract exclusion |
| 106 | Branch, K. R.  Caldwell, J. H.  Soine, L.  O'Brien, K. D. | 2005 | Vascular (humoral) cardiac allograft rejection manifesting as inducible myocardial ischemia on nuclear perfusion imaging | Title-abstract exclusion |
| 107 | Brasington, R.  Miller, J.  Spitzer, V.  Holers, V. M.  Sherzinger, A.  Lawson, A.  Mahowald, M. L. | 2009 | Training, practice and assessment of arthrocentesis procedure skills without risk to a patient | Title-abstract exclusion |
| 108 | Brazil, A. L.  Conci, A.  Clua, E.  Rodriguez-Hernandez, N.  Bittencourt, L. K.  Ramos, R. R. | 2016 | Force modeling and gamification for Epidural Anesthesia training | Title-abstract exclusion |
| 109 | Breunissen, Esther Helena Wouda  Groenveld, Tjitske Diederike  Garms, Linda  Bonnes, Judith L.  van Goor, Harry  Damman, Peter | 2024 | Virtual reality to reduce periprocedural anxiety during invasive coronary angiography: rationale and design of the VR InCard trial | Title-abstract exclusion |
| 110 | Bricault, I.  Ferretti, G.  Cinquin, P. | **1998** | Registration of real and CT-derived virtual bronchoscopic images to assist transbronchial biopsy | Title-abstract exclusion |
| 111 | Bro-Nielsen, M.  Tasto, J. L.  Cunningham, R.  Merril, G. L. | **1999** | Preop™ endoscopic simulator: A PC-based immersive training system for bronchoscopy | Title-abstract exclusion |
| 112 | Brouwer, O. R.  Wendler, T.  Van De Hage, J.  Van Leeuwen, F.  Valdés Olmos, R. A. | 2011 | First in-vivo experience with fused SPECT/CT and real-time video visualization for intraoperative guidance in sentinel lymph node biopsies | Title-abstract exclusion |
| 113 | Brown, M.  Krishnananthan, N.  Paul, V. | 2022 | Right heart catherisation - a virtual reality | Title-abstract exclusion |
| 114 | Brown, M.  Krishnananthan, N.  Paul, V. | 2022 | Virtual Reality Training in Right Heart Catheterisation | Title-abstract exclusion |
| 115 | Brunzini, Agnese  Peruzzini, Margherita  Barbadoro, Pamela | 2023 | Human-centred data-driven redesign of simulation-based training: a qualitative study applied on two use cases of the healthcare and industrial domains | Title-abstract exclusion |
| 116 | Bryan, E. R.  McLachlan, R. I.  Rombauts, L.  Katz, D. J.  Yazdani, A.  Bogoevski, K.  Chang, C.  Giles, M. L.  Carey, A. J.  Armitage, C. W.  Trim, L. K.  McLaughlin, E. A.  Beagley, K. W. | 2019 | Detection of chlamydia infection within human testicular biopsies | Title-abstract exclusion |
| 117 | Brzozowski, Tomasz  Konturek, Peter C.  Pajdo, Robert  Kwiecien, Slawomir  Bielanski, Wladyslaw  Kuwahara, Atsukazu  Kato, Jin  Konturek, Stanislaw J.  Pawlik, Wieslaw W. | 2006 | Orexin-a, an. appetite hormone, attenuates gastric ischemia-reperfusion injury via of prostaglandin/cyclooxygenase pathway, vagal and afferent sensory nerves | Title-abstract exclusion |
| 118 | Buchbender, C.  Treffert, J.  Lehnerdt, G.  Mattheis, S.  Geiger, B.  Bockisch, A.  Forsting, M.  Antoch, G.  Heusner, T. A. | 2012 | Virtual 3-D 18F-FDG PET/CT panendoscopy for assessment of the upper airways of head and neck cancer patients: A feasibility study | Title-abstract exclusion |
| 119 | Burtscher, J.  Bale, R.  Dessl, A.  Eisner, W.  Twerdy, K.  Sweeney, R. A.  Felber, S. | 2002 | Virtual endoscopy for planning neuro-endoscopic intraventricular surgery | Title-abstract exclusion |
| 120 | Busque, S.  Leventhal, J.  Brennan, D. C.  Steinberg, S.  Klintmalm, G.  Shah, T.  Mulgaonkar, S.  Bromberg, J. S.  Vincenti, F.  Hariharan, S.  et al., | 2009 | Calcineurin-inhibitor-free immunosuppression based on the JAK inhibitor CP-690,550: a pilot study in de novo kidney allograft recipients | Title-abstract exclusion |
| 121 | Buthiau, D.  Rixe, O.  Spano, J. P.  Nizri, D.  Delgado, M.  Gutierrez, M.  Bloch, J.  Bloch, P.  Rocher, M. A.  Khayat, D. | 2003 | New imaging techniques in oncology | Title-abstract exclusion |
| 122 | Caballero, R.  Pasten, A.  Giménez, C.  Rodríguez, R.  Carmona, R. M.  Mora, J.  Valls-Esteve, A.  Lustig, P.  Lombardini, F.  Balsells, S.  Krauel, L. | 2024 | Beyond Needles: Pioneering Pediatric Care with Virtual Reality (VR) for TIVAD Access in Oncology | Title-abstract exclusion |
| 123 | Cadeddu, J. A.  Stoianovici, D.  Kavoussi, L. R. | **1997** | Robotics in urologic surgery | Title-abstract exclusion |
| 124 | Cadeddu, J. A.  Stoianovici, D.  Kavoussi, L. R. | **1998** | Robotic surgery in urology | Title-abstract exclusion |
| 125 | Cakir, O. O.  Castiglione, F.  Tandogdu, Z.  Collins, J.  Alnajjar, H. M.  Akers, C.  Albersen, M.  Alifrangis, C.  Ayres, B.  et al., | 2021 | Management of penile cancer patients during the COVID-19 pandemic: An eUROGEN accelerated Delphi consensus study | Title-abstract exclusion |
| 126 | Caltagirone, R.  Raghavan, V. R.  Adelgais, K.  Roosevelt, G. E. | 2018 | A Randomized Double Blind Trial of Needle-free Injected Lidocaine Versus Topical Anesthesia for Infant Lumbar Puncture | Title-abstract exclusion |
| 127 | Camacho-Cruz, J.  Palacios-Ariza, M. A.  Orrego-Celestino, L.  Valbuena-Velandia, N.  Paez-Castellanos, L.  Bolaños, J. M.  Pradilla, I. | 2023 | Effectiveness of non-immersive virtual reality in the management of procedure-related pain in preschool children: a randomized clinical trial | Title-abstract exclusion |
| 128 | Camparo, P.  Ramirez, A.  Claude, V.  Bertocchi, C.  Masse, S.  Blancard, P.  Branquet, D. | 2008 | Application of telepathology for diagnosis in interhospital network: Experience of the Paris Military Hospital | Title-abstract exclusion |
| 129 | Campone, M.  Marschner, N.  Villanueva, C.  Sonke, G. S.  Alba, E.  Jakobsen, E.  Su, F.  He, W.  Germa, C.  Andre, F. | 2017 | First-line ribociclib 1 letrozole in HR1, HER2-ABC: efficacy by baseline tumor markers | Title-abstract exclusion |
| 130 | Campo-Ruiz, V.  Patel, D.  Anderson, R. R.  Delgado-Baeza, E.  González, S. | 2006 | Virtual biopsy of the joint tissues using near-infrared, reflectance confocal microscopy. A pilot study | Title-abstract exclusion |
| 131 | Canares, T.  Parrish, C.  Santos, C.  Badawi, A.  Kleinman, K.  Stewart, A.  Biddle, A.  Brylske, P.  McGuire, J. | 2021 | Optimizing coping during venipuncture procedures with virtualreality in the pediatric emergency department: a randomizedcontrolled trial | Title-abstract exclusion |
| 132 | Canares, T. L.  Parrish, C.  Santos, C.  Badawi, A.  Stewart, A.  Kleinman, K.  Psoter, K. J.  McGuire, J. F. | 2021 | Resource use during pediatric venipuncture with virtual reality: Secondary analysis of a randomized controlled pilot trial | Title-abstract exclusion |
| 133 | Canda, Abdullah Erdem  Aksoy, Sertac Fatih  Altinmakas, Emre  Koseoglu, Ersin  Falay, Okan  Kordan, Yakup  Cil, Barbaros  Balbay, Mevlana Derya  Esen, Tarik | 2020 | Virtual reality tumor navigated robotic radical prostatectomy by using three-dimensional reconstructed multiparametric prostate MRI and 68Ga-PSMA PET/CT images: A useful tool to guide the robotic surgery? | Title-abstract exclusion |
| 134 | Cao, Z.  Xiu, Y.  Yu, D.  Li, X.  Yang, C.  Li, Z. | 2023 | Clinical Value of Mixed Reality-Assisted Puncture Navigation for Percutaneous Nephrolithotripsy | Title-abstract exclusion |
| 135 | Carnt, Nicole Ann  Connor, Sophie  Parayoganathan, Varshini  Keay, Lisa | 2019 | Predictors of quality of life for <i>Acanthamoeba</i> keratitis patients | Title-abstract exclusion |
| 136 | Carrascosa, P.  Capuñay, C.  Ulla, M.  López, E. M.  Corti, R.  Carrascosa, J. | 2006 | Elevated gastric lesions: Virtual gastroscopy | Title-abstract exclusion |
| 137 | Carvalho, A. D. D.  Souza, F. D.  Silva, R. B.  Bavaresco, A. Z.  Voll, J.  Contesini, E. A.  Reckziegel, S. H. | 2012 | Anatomical techniques as a complementary method for learning nephrotomy | Title-abstract exclusion |
| 138 | Casal, R. F. | 2015 | Reply | Title-abstract exclusion |
| 139 | Castrejón-Castrejón, S.  Pérez-Silva, A.  González-Villegas, E.  Al-Razzo, O.  Silvestre, J.  Doiny, D.  Estrada-Mucci, A.  Filgueiras-Rama, D.  Ortega-Molina, M.  López-Sendón, J. L.  Merino, J. L. | 2013 | Implantation of cardioverter defibrillators with minimal fluoroscopy using a three-dimensional navigation system: A feasibility study | Title-abstract exclusion |
| 140 | Cattari, Nadia  Condino, Sara  Cutolo, Fabrizio  Ferrari, Mauro  Ferrari, Vincenzo | 2021 | In Situ Visualization for 3D Ultrasound-Guided Interventions with Augmented Reality Headset | Title-abstract exclusion |
| 141 | Cattari, Nadia  Condino, Sara  Cutolo, Fabrizio  Ghilli, Matteo  Ferrari, Mauro  Ferrari, Vincenzo | 2021 | Wearable AR and 3D Ultrasound: Towards a Novel Way to Guide Surgical Dissections | Title-abstract exclusion |
| 142 | Cattari, Nadia  Cutolo, Fabrizio  Placa, Luciana La  Ferrari, Vincenzo | 2024 | Visualization modality for augmented reality guidance of in-depth tumour enucleation procedures | Title-abstract exclusion |
| 143 | César de Almeida, J.  Dellaretti, M.  Piaulino Benvindo Ferreira, P. H.  Ferreira, T. A.  Marques do Nascimento, L.  Faria, M. P. | 2020 | Evaluation of Accuracy and Reliability of a Smartphone Stereotactic Coordinates Checking Application | Title-abstract exclusion |
| 144 | Chae, S.  Jung, S. W.  Park, H. S. | 2018 | In vivo biomechanical measurement and haptic simulation of portal placement procedure in shoulder arthroscopic surgery | Title-abstract exclusion |
| 145 | Chae, Sanghoon  Jung, Sung-Weon  Park, Hyung-Soon | 2019 | <i>In vivo</i> biomechanical measurement and haptic simulation of portal placement procedure in shoulder arthroscopic surgery | Title-abstract exclusion |
| 146 | Chakraborty, Arjun  Sarwal, Minnie | 2018 | Protein biomarkers in renal transplantation | Title-abstract exclusion |
| 147 | Chalasani, Venu  Cool, Derek W.  Sherebrin, Shi  Fenster, Aaron  Chin, Joseph  Izawa, Jonathan I. | 2011 | Development and validation of a virtual reality transrectal ultrasound guided prostatic biopsy simulator | Title-abstract exclusion |
| 148 | Chalasani, Venu  Cool, Derek W.  Sherebrin, Shi  Fenster, Aaron  Chin, Joseph L.  Izawa, Jonathan I. | 2009 | VIRTUAL REALITY TRANSRECTAL ULTRASOUND GUIDED PROSTATIC BIOPSY SIMULATOR | Title-abstract exclusion |
| 149 | Chamberlain, Ronald S.  Patil, Sachin  Minja, Emmanuel J.  Kordears, Kristen | 2012 | Does residents' involvement in mastectomy cases increase operative cost? If so, who should bear the cost? | Title-abstract exclusion |
| 150 | Chan, E.  Hovenden, M.  Ramage, E.  Ling, N.  Pham, J. H.  Rahim, A.  Lam, C.  Liu, L.  **et al.** | 2019 | Virtual Reality for Pediatric Needle Procedural Pain: Two Randomized Clinical Trials | Title-abstract exclusion |
| 151 | Chan, L.  Andres, A.  Bunnapradist, S.  Gugliuzza, K.  Parasuraman, R.  Peddi, V. R.  Cassuto, E.  Hart, M. | 2012 | Renal function and NODM in de novo renal transplant recipients treated with standard and reduced levels of tacrolimus in combination with EC-MPS | Title-abstract exclusion |
| 152 | Chan, W. Y.  Ni, D.  Pang, W. M.  Qin, J.  Chui, Y. P.  Yu, S. C. H.  Heng, P. A. | 2010 | Learning ultrasound-guided needle insertion skills through an edutainment game | Title-abstract exclusion |
| 153 | Chang, Joseph  Ninan, Sen  Liu, Katherine  Iloreta, Alfred Marc  Kirke, Diana  Courey, Mark | 2021 | Enhancing Patient Experience in Office-Based Laryngology Procedures With Passive Virtual Reality | Title-abstract exclusion |
| 154 | Chartier, S.  Arif-Tiwari, H. | 2023 | MR Virtual Biopsy of Solid Renal Masses: An Algorithmic Approach | Title-abstract exclusion |
| 155 | Chavanon, O.  Barbe, C.  Troccaz, J.  Carrat, L.  Ribuot, C.  Blin, D. | **1997** | Computer Assisted PERicardial punctures: Animal feasability study | Title-abstract exclusion |
| 156 | Checcucci, E.  Amparore, D.  Volpi, G.  Piramide, F.  De Cillis, S.  Piana, A.  Alessio, P.  Verri, P.  **et al.** | 2022 | Percutaneous puncture during PCNL: new perspective for the future with virtual imaging guidance | Title-abstract exclusion |
| 157 | Checcucci, Enrico  De Cillis, Sabrina  Granato, Stefano  Chang, Peter  Afyouni, Andrew Shea  Okhunov, Zhamshid  European Association, Urology | 2020 | Applications of neural networks in urology: a systematic review | Title-abstract exclusion |
| 158 | Checcucci, E.  De Cillis, S. T.  Amparore, D.  Volpi, G.  Gatti, C.  Paolo, A.  Piramide, F.  Piana, A.  Cisero, E.  Ortenzi, M.  et al., | 2024 | Artificial intelligence 3D augmented reality guided RARP vs. Cognitive MRI intervention: ad interim analysis of RIDERS Trial | Title-abstract exclusion |
| 159 | Checcucci, Enrico  Piana, Alberto  Volpi, Gabriele  Piazzolla, Pietro  Amparore, Daniele  De Cillis, Sabrina  Piramide, Federico  **et al.** | 2023 | Three-dimensional automatic artificial intelligence driven augmented-reality selective biopsy during nerve-sparing robot-assisted radical prostatectomy: A feasibility and accuracy study | Title-abstract exclusion |
| 160 | Checcucci, Enrico  Piana, Alberto  Volpi, Gabriele  Piazzolla, Pietro  Amparore, Daniele  De Cillis, Sabrina  Piramide, Federico  **et al.** | 2024 | Visual extended reality tools in image-guided surgery in urology: a systematic review | Title-abstract exclusion |
| 161 | Chellali, Amine  Dumas, Cedric  Milleville-Pennel, Isabelle | 2012 | Haptic Communication to Support Biopsy Procedures Learning in Virtual Environments | Title-abstract exclusion |
| 162 | Chellali, A.  Dumas, C.  Milleville-Pennel, I. | 2013 | Haptic communication to support biopsy procedures learning in virtual environments | Title-abstract exclusion |
| 163 | Chen, Chi-Ya  Elarbi, Mustafa  Ragle, Claude A.  Fransson, Boel A. | 2019 | Development and evaluation of a high-fidelity canine laparoscopic ovariectomy model for surgical simulation training and testing | Title-abstract exclusion |
| 164 | Chen, E. C. S.  Ameri, G.  Li, H.  Sondekoppam, R. V.  Ganapathy, S.  Peters, T. M. | 2014 | Navigated simulator for spinal needle interventions | Title-abstract exclusion |
| 165 | Chen, J.  Ren, L. N.  Han, Q. | 2023 | Application and Study of Virtual Reality in The Diagnosis System of Peritoneal Effusion | Title-abstract exclusion |
| 166 | Chen, Long  Zhang, Fengfeng  Zhan, Wei  Gan, Minfeng  Sun, Lining | 2020 | Optimization of virtual and real registration technology based on augmented reality in a surgical navigation system | Title-abstract exclusion |
| 167 | Chen, N.  Yang, Z.  Ye, B.  Guo, H.  Wang, F.  Zhang, C.  Wang, D. | 2024 | The Application Value of Virtual Reality Navigation Combined with Rapid On-Site Evaluation in CT-Guided Lung Biopsy | Title-abstract exclusion |
| 168 | Chen, S. Y.  Chen, S. U.  Wu, H. Y.  Lee, W. J.  Liao, Y. H.  Sun, C. K. | 2010 | In vivo virtual biopsy of human skin by using noninvasive higher harmonic generation microscopy | Title-abstract exclusion |
| 169 | Chen, S. Y.  Wu, H. Y.  Sun, C. K. | 2009 | In vivo harmonic generation biopsy of human skin | Title-abstract exclusion |
| 170 | Chen, W. J.  Wang, X. T.  Yang, Y. J.  Tan, X. X.  Zhong, M.  Duan, Y. X.  Wang, Y.  Zhuge, Q. C. | 2008 | An experimental study of cerebral basilar artery vasospasm on CT angiography in a rabbit model | Title-abstract exclusion |
| 171 | Chen, X.  Cheng, J.  Gu, X.  Sun, Y.  Politis, C. | 2016 | Development of preoperative planning software for transforaminal endoscopic surgery and the guidance for clinical applications | Title-abstract exclusion |
| 172 | Chessa, F.  Schiavina, R.  Bianchi, L.  Marcelli, E.  Diciotti, S.  Lodi, S.  Gaudiano, C.  Giunchi, F.  Bortolani, B.  Cercenelli, L.  et al., | 2020 | Three dimensional model of the prostate and augmented reality robot assisted radical prostatectomy: a randomized controlled study to evaluate intraoperative and pathologic outcomes | Title-abstract exclusion |
| 173 | Cheung, J. J. H.  Chen, E. W.  Al-Allaq, Y.  Nikravan, N.  McCartney, C. J. L.  Dubrowski, A.  Awad, I. T. | 2011 | Acquisition of technical skills in ultrasound-guided regional anesthesia using a high-fidelity simulator | Title-abstract exclusion |
| 174 | Chew, S. C.  Beh, Z. Y.  Hakumat Rai, V. R.  Jamaluddin, M. F.  Ng, C. C.  Chinna, K.  Hasan, M. S. | 2020 | Ultrasound-guided central venous vascular access-novel needle navigation technology compared with conventional method: a randomized study | Title-abstract exclusion |
| 175 | ChiCtr, | 2021 | Research and Application of a Teaching Platform for Intracaspinal Puncture based on Virtual Reality and Haptic Feedback Technology | Title-abstract exclusion |
| 176 | ChiCtr, | 2022 | Effect of virtual reality intervention mode based on meditation trip on anxiety, fear and pain in breast biopsy patients | Trial registry record |
| 177 | ChiCtr, | 2024 | A study of the efficacy of immersive virtual reality to improve the experience of maternal intraspinal anesthesia operations | Title-abstract exclusion |
| 178 | ChiCtr, | 2024 | Exploring the transfer of ultrasound-guided central venous puncture placement to clinical skills in infants and children based on adult model simulation training | Title-abstract exclusion |
| 179 | Chollangi, Ravikiran | 2014 | Design and development of a haptic device for a trocar insertion minimum invasive procedure simulator | Title-abstract exclusion |
| 180 | Choueib, S.  McGarry, C.  Jaeger, M.  Ungi, T.  Janssen, N.  Fichtinger, G.  Patterson, L. | 2020 | Assessment of skill translation of intrathecal needle insertion using real-time needle tracking with an augmented reality display | Title-abstract exclusion |
| 181 | Chow, H.  Hon, J.  Chua, W.  Chuan, A. | 2021 | Effect of Virtual Reality Therapy in Reducing Pain and Anxiety for Cancer-Related Medical Procedures: A Systematic Narrative Review | Title-abstract exclusion |
| 182 | Christian, Eisha A.  Melamed, Edward F.  Peck, Edwin  Krieger, Mark D.  McComb, J. Gordon | 2016 | Surgical management of hydrocephalus secondary to intraventricular hemorrhage in the preterm infant | Title-abstract exclusion |
| 183 | Chuaypen, Natthaya  Posuwan, Nawarat  Payungporn, Sunchai  Tanaka, Yasuhito  Shinkai, Noboru  Poovorawan, Yong  Tangkijvanich, Pisit | 2016 | Serum hepatitis B core-related antigen as a treatment predictor of pegylated interferon in patients with HBeAg-positive chronic hepatitis B | Title-abstract exclusion |
| 184 | Chuaypen, Natthaya  Sriprapun, Methee  Praianantathavorn, Kesmanee  Payungporn, Sunchai  Wisedopas, Naruemon  Poovorawan, Yong  Tangkijvanich, Pisit | 2017 | Kinetics of serum HBsAg and intrahepatic cccDNA during pegylated interferon therapy in patients with HBeAg-positive and HBeAg-negative chronic hepatitis B | Title-abstract exclusion |
| 185 | Chuchu, N.  Dinnes, J.  Takwoingi, Y.  Matin, R. N.  et al., | 2018 | Teledermatology for diagnosing skin cancer in adults | Title-abstract exclusion |
| 186 | Chuchu, N.  Takwoingi, Y.  Dinnes, J.  Matin, R. N.  Bassett, O.  Moreau, J. F.  et al., | 2018 | Smartphone applications for triaging adults with skin lesions that are suspicious for melanoma | Title-abstract exclusion |
| 187 | Chung, C.  Bryant, A.  Brown, P. D. | 2018 | Interventions for the treatment of brain radionecrosis after radiotherapy or radiosurgery | Title-abstract exclusion |
| 188 | Ciobirca, C.  Gruionu, G.  Lango, T.  Leira, H. O.  Gruionu, L. G.  Amundsen, T.  Nutu, E.  Pastrama, S. D. | 2017 | An algorithm to obtain a theoretical model of the bronchial tree | Title-abstract exclusion |
| 189 | Codrington, J.  Qureshi, F.  Rahman, F.  Saltzman, R.  Deebel, N.  Cartaya, S.  Leong, P.  White, J.  Muthigi, A.  Ramasamy, R. | 2024 | VIRTUAL REALITY FOR IMPROVED VASECTOMY EXPERIENCE: INSIGHTS FROM A SINGLE-CENTER CLINICAL TRIAL | Title-abstract exclusion |
| 190 | Coles, T. R.  John, N. W.  Sofia, G.  Gould, D. A.  Caldwell, D. G. | 2011 | Modification of commercial force feedback hardware for needle insertion simulation | Title-abstract exclusion |
| 191 | Conci, A.  Brazil, A. L.  Popovici, D.  Jiga, G.  Lebon, F. | 2018 | Modeling the behavior of human body tissues on penetration | Title-abstract exclusion |
| 192 | Cooke, C. M.  Flaxman, T. E.  La Russa, D. J.  Duigenan, S.  Singh, S. S. | 2023 | Endometriosis Imaging: Enter the Metaverse of Possibilities | Title-abstract exclusion |
| 193 | Correa, Cleber G.  Nunes, Fatima L. S.  Ranzini, Edith  Nakamura, Ricardo  Tori, Romero | 2019 | Haptic interaction for needle insertion training in medical applications: The state-of-the-art | Title-abstract exclusion |
| 194 | Corrêa, E. S.  Oliveira, T. E. A. D.  Trenhago, P. R.  Oliveira, J. C. D. | 2013 | Performance analisys of the xSight 3120 HMD in a pleural puncture medical simulation | Title-abstract exclusion |
| 195 | Corrêa, V. P. S.  Melo, M. T. D.  Nogueira, V. F.  Gonçalves, V. H. L.  Costa, H. D. R.  Melo, J. S. S.  Rodrigues, B. A.  Brasil, L. M. | 2015 | Deformation method and 3D modeling of the female body to simulate core biopsy procedure | Title-abstract exclusion |
| 196 | Costa, Jose N.  Gomes-Fonseca, Joao  Valente, Simao  Ferreira, Luis  Oliveira, Bruno  Torres, Helena R.  Morais, Pedro  Alves, Victor  Vilaca, Joao L. | 2022 | Ultrasound training simulator using augmented reality glasses: an accuracy and precision assessment study | Title-abstract exclusion |
| 197 | Costa, Nuno  Ferreira, Luis  de Araujo, Augusto R. V. F.  Oliveira, Bruno  Torres, Helena R. R.  Morais, Pedro  Alves, Victor  Vilaca, Joao L. | 2023 | Augmented Reality-Assisted Ultrasound Breast Biopsy | Title-abstract exclusion |
| 198 | Costello, Sean S. P.  Johnston, Daniel J.  Dervan, Peter A.  O'Shea, Daniel G. | 2003 | Development and Evaluation of the Virtual Pathology Slide: A New Tool in Telepathology | Title-abstract exclusion |
| 199 | Cousins, J. N.  Wong, K. F.  Chee, M. W. L. | 2019 | Multi-Night Sleep Restriction Impairs Long-Term Retention of Factual Knowledge in Adolescents | Title-abstract exclusion |
| 200 | Cox, T. C.  Pearl, J. P.  Matthew Ritter, E. | 2011 | S.C.O.P.E.: Simulated colonoscopy objective performance evaluation | Title-abstract exclusion |
| 201 | Cremaschi, F.  Fausti, R.  Núñez, V.  Duca, E.  Glantz, M.  Casas, S. | 2022 | PO003 / #249 DEVELOPMENT AND VALIDATION OF AN IMMERSIVE SIMULATION IN VIRTUAL REALITY AND WITH HAPTIC TECHNOLOGY OF THE STEREOTACTIC TECHNIQUE FOR TUMOR BIOPSY: E-POSTER VIEWING | Title-abstract exclusion |
| 202 | Criner, G. J.  Eberhardt, R.  Fernandez-Bussy, S.  Gompelmann, D.  Maldonado, F.  Patel, N.  Shah, P. L.  et al | 2020 | Interventional bronchoscopy | Title-abstract exclusion |
| 203 | Cristiano, E.  Abad, P.  Becker, J.  Carrá, A.  Correale, J.  Flores, J.  Fruns, M.  Garcea, O.  et al | 2021 | Multiple sclerosis care units in Latin America: Consensus recommendations about its objectives and functioning implementation | Title-abstract exclusion |
| 204 | Cserni, T.  Cervellione, R. M.  Hajnal, D.  Varga, G.  Kubiak, R.  et al | 2015 | Alternative ileal flap for bladder augmentation if mesentery is short | Title-abstract exclusion |
| 205 | Ctri, | 2024 | Efficacy of smartphone based virtual reality in reducing anxiety during dermatosurgical procedures | Title-abstract exclusion |
| 206 | Ctri, | 2024 | To study the effect of virtual reality during various procedures like venipuncture in children with hematological malignancies | Title-abstract exclusion |
| 207 | Dahmke, T.  Färber, M.  Bohn, C. A.  Handels, H. | 2009 | VR training simulator for lumbar and ascites punctures with elastic needle deflection | Title-abstract exclusion |
| 208 | Dai, Junlong  Qi, Weili  Qiu, Zhancheng  Li, Chuan | 2023 | The application and prospection of augmented reality in hepato-pancreato-biliary surgery | Title-abstract exclusion |
| 209 | Dall'Orto, Clarissa Campo  Lapa, Guilherme Alves  Carnieto, Nádia de Mendonça  Siqueira, Breno de  Oliveira Neto, João Batista de  Mauro, Maria Fernanda Zuliani  Cristóvão, Salvador André Bavaresco  Salman, Adnan Ali  Mangione, José Armando | 2009 | Experiência inicial utilizando a via radial no tratamento percutâneo de doença coronária | Title-abstract exclusion |
| 210 | Dar, P.  Gebb, J.  Reimers, L.  Bernstein, P. S.  Chazotte, C.  Merkatz, I. R. | 2010 | First-trimester 3-dimensional power Doppler of the uteroplacental circulation space: A potential screening method for preeclampsia | Title-abstract exclusion |
| 211 | Das, D. K.  Gupta, A. K.  Chowdhury, V.  Satsangi, D. K.  Tyagi, S.  Mohan, J. C.  Khan, V. A.  Malhotra, V. | **1997** | Fine-needle aspiration diagnosis of carotid body tumor: report of a case and review of experience with cytologic features in four cases | Title-abstract exclusion |
| 212 | Das, M.  Sauer, F.  Schoepf, U. J.  Khamene, A.  Vogt, S. K.  Schaller, S.  Kikinis, R.  VanSonnenberg, E.  Silverman, S. G. | 2006 | Augmented reality visualization for CT-guided interventions: System description, feasibility, and initial evaluation in an abdominal phantom | Title-abstract exclusion |
| 213 | Davenport, C.  Rai, N.  Sharma, P.  Deeks, J. J.  Berhane, S.  Mallett, S.  Saha, P.  Champaneria, R.  Bayliss, S. E.  Snell, K. I. E.  et al., | 2022 | Menopausal status, ultrasound and biomarker tests in combination for the diagnosis of ovarian cancer in symptomatic women | Title-abstract exclusion |
| 214 | Davids, M. S.  Rogers, K. A.  Tyekucheva, S.  Wang, Z.  Pazienza, S.  Renner, S. K.  et al., | 2022 | Venetoclax plus dose-adjusted R-EPOCH for Richter syndrome | Title-abstract exclusion |
| 215 | Davoudi, Mohsen  Wahidi, Momen M.  Rohani, Nazanin Zamanian  Colt, Henri G. | 2010 | Comparative Effectiveness of Low- and High-Fidelity Bronchoscopy Simulation for Training in Conventional Transbronchial Needle Aspiration and User Preferences | Title-abstract exclusion |
| 216 | Davrieux, Carlos F.  Gimenez, Mariano E.  Gonzalez, Cristians A.  Ancel, Alexandre  Guinin, Maxime  Fahrer, Benedicte  Serra, Edgardo  Kwak, Jung-Myun  Marescaux, Jacques  Hostettler, Alexandre | 2020 | Mixed reality navigation system for ultrasound-guided percutaneous punctures: a pre-clinical evaluation | Title-abstract exclusion |
| 217 | Daw, Z.  Cleaveland, R.  Vetter, M. | 2014 | Formal verification of software-based medical devices considering medical guidelines | Title-abstract exclusion |
| 218 | De Almeida Souza, I.  Sanches Jr, C.  Kondo, M. N. S.  Zuffo, M. K. | 2008 | Development and evaluation of a virtual reality simulator for training of thyroid gland nodules needle biopsy | Title-abstract exclusion |
| 219 | De Carvalho, F. G.  Brandao, C. F. C.  Batitucci, G.  Souza, A. O.  Ferrari, G. D.  Alberici, L. C.  Muñoz, V. R.  Pauli, J. R.  De Moura, L. P.  Ropelle, E. R.  et al., | 2021 | Taurine supplementation associated with exercise increases mitochondrial activity and fatty acid oxidation gene expression in the subcutaneous white adipose tissue of obese women | Title-abstract exclusion |
| 220 | De Carvalho, F. G.  Brandao, C. F. C.  Muñoz, V. R.  Batitucci, G.  Tavares, M. E. A.  Teixeira, G. R.  Pauli, J. R.  De Moura, L. P.  Ropelle, E. R.  Cintra, D. E.  et al., | 2021 | Taurine supplementation in conjunction with exercise modulated cytokines and improved subcutaneous white adipose tissue plasticity in obese women | Title-abstract exclusion |
| 221 | De Keersmaecker, E.  Beckwée, D.  Denissen, S.  Nagels, G.  Swinnen, E. | 2021 | Virtual reality for multiple sclerosis rehabilitation | Title-abstract exclusion |
| 222 | De la Cruz-Ku, G.  Mallouh, M. P.  Torres Roman, J. S.  Linshaw, D. | 2023 | Three-dimensional virtual reality in surgical planning for breast cancer with reconstruction | Title-abstract exclusion |
| 223 | De Luca, L.  Barone, B.  Napolitano, L.  Crocetto, F.  Caputo, V. F.  Creta, M.  Fusco, F. | 2021 | Effects of virtual reality on perceived pain during transrectal biopsy | Conference Abstract |
| 224 | De Melo, R. H. C.  Conci, A. | 2021 | Modeling the basic behaviors of Anesthesia Training in Relation to Puncture and Penetration Feedback | Title-abstract exclusion |
| 225 | De Oliveira, A. C. M. T. G.  Dos Santos Nunes, F. D. L. | 2010 | Building a open source framework for virtual medical training | Title-abstract exclusion |
| 226 | De Oliveira, A. C. M. T. G.  Tori, R.  Bernardes, J. L.  Torres, R. S.  Nunes, F. L. S. | 2024 | Simulation of deformation in models of human organs using physical parameters | Title-abstract exclusion |
| 227 | De Souza-Junior, V. D.  Mendes, I. A. C.  Marchi-Alves, L. M.  Jackman, D.  Wilson-Keates, B.  De Godoy, S. | 2020 | Peripheral Venipuncture Education Strategies for Nursing Students: An Integrative Literature Review | Title-abstract exclusion |
| 228 | De Wever, W.  Bogaert, J.  Verschakelen, J. A. | 2005 | Virtual bronchoscopy: Accuracy and usefulness - An overview | Title-abstract exclusion |
| 229 | De Wever, W.  Vandecaveye, V.  Lanciotti, S.  Verschakelen, J. A. | 2004 | Multidetector CT-generated virtual bronchoscopy: An illustrated review of the potential clinical indications | Title-abstract exclusion |
| 230 | Decampli, William M. | **1998** | Video-assisted thoracic surgical procedures in children | Title-abstract exclusion |
| 231 | Deguchi, D.  Feuerstein, M.  Kitasaka, T.  Suenaga, Y.  Ide, I.  Murase, H.  Imaizumi, K.  Hasegawa, Y.  Mori, K. | 2012 | Real-time marker-free patient registration for electromagnetic navigated bronchoscopy: A phantom study | Title-abstract exclusion |
| 232 | Deguchi, D.  Mori, K.  Mekada, Y.  Hasegawa, J. I.  Toriwaki, J. I.  Noguchi, M. | 2006 | Development of a virtual needle biopsy simulation system for the virtual prostate | Title-abstract exclusion |
| 233 | Dekker, E.  Fockens, P. | 2004 | New endoscopic tools for the IBD physician | Title-abstract exclusion |
| 234 | Delbos, A. | 2014 | Learning regional anesthesia by simulation: Possible? | Title-abstract exclusion |
| 235 | Delengowski, A.  Lasota, M.  Galanis, P.  Vavala, V.  Kearns, L. | 2021 | Improving Patient Experience during Bone Marrow Biopsies | Title-abstract exclusion |
| 236 | Della Corte, Marcello  Quara, Alberto  De Cillis, Sabrina  Volpi, Gabriele  Amparore, Daniele  Piramide, Federico  Piana, Alberto  Sica, Michele  **et al** | 2024 | 3D virtual models and augmented reality for radical prostatectomy: a narrative review | Title-abstract exclusion |
| 237 | Delpero, Jean Robert  Sauvanet, Alain | 2020 | Vascular Resection for Pancreatic Cancer: 2019 French Recommendations Based on a Literature Review From 2008 to 6-2019 | Title-abstract exclusion |
| 238 | Demartines, N.  Battegay, E.  Liebermann, J.  Oberholzer, M.  Rufli, T.  Harder, F. | 2000 | Telemedicine: outlook and multidisciplinary approach | Title-abstract exclusion |
| 239 | Denkert, C.  Jank, P.  Link, T. | 2019 | Expression of ER, PR, HER2 and Ki67 in the neoadjuvant GeparX trial: comparison of central immunohistochemistry with an automated cartridge-based system for mRNA assessment (on behalf of the GBG neoadjuvant and translational subboard) | Title-abstract exclusion |
| 240 | deSouza, N. M. | 2021 | Imaging to assist fertility-sparing surgery | Title-abstract exclusion |
| 241 | Di Vece, Chiara | 2019 | Improvement of Psychomotor Skill Development for Veress Needle Insertion with Haptics and Virtual Reality | Title-abstract exclusion |
| 242 | Di Vece, Chiara  Luciano, Cristian  De Momi, Elena | 2021 | Psychomotor skills development for Veress needle placement using a virtual reality and haptics-based simulator | Title-abstract exclusion |
| 243 | Diana, M.  Agnus, V.  Halvax, P.  Liu, Y. Y.  Dallemagne, B.  Schlagowski, A. I.  Geny, B.  Diemunsch, P.  Lindner, V.  Marescaux, J. | 2015 | Intraoperative fluorescence-based enhanced reality laparoscopic real-time imaging to assess bowel perfusion at the anastomotic site in an experimental model | Title-abstract exclusion |
| 244 | Diana, M.  Noll, E.  Diemunsch, P.  Dallemagne, B.  Benahmed, M. A.  Agnus, V.  Soler, L.  **et al** | 2014 | Enhanced-reality video fluorescence: A real-time assessment of intestinal viability | Title-abstract exclusion |
| 245 | Diaz, Gabriela M.  Webb, Lindsey T.  Rabil, Maximilian J.  Lokeshwar, Soum D.  Choksi, Ankur U.  Leapman, Michael S.  Sprenkle, Preston C. | 2024 | Risk Factors and Contemporary Management Options for Pain and Discomfort Experienced During a Prostate Biopsy | Title-abstract exclusion |
| 246 | Diez-Ferrer, M.  Morales, A.  Tebé, C.  Cubero, N.  López-Lisbona, R.  Padrones, S.  Aso, S.  Dorca, J.  Gil, D.  Rosell, A. | 2019 | Ultrathin Bronchoscopy with and without Virtual Bronchoscopic Navigation: Influence of Segmentation on Diagnostic Yield | Title-abstract exclusion |
| 247 | DiMaio, S. P.  Pieper, S.  Chinzei, K.  Hata, N.  Balogh, E.  Fichtinger, G.  Tempany, C. M.  Kikinis, R. | 2006 | Robot-assisted needle placement in open-MRI: System architecture, integration and validation | Title-abstract exclusion |
| 248 | DiMaio, S. P.  Salcudean, S. E. | 2005 | Interactive simulation of needle insertion models | Title-abstract exclusion |
| 249 | Ding, W.  Nayak, J.  Swapnarekha, H.  Abraham, A.  Naik, B.  Pelusi, D. | 2021 | Fusion of intelligent learning for COVID-19: A state-of-the-art review and analysis on real medical data | Title-abstract exclusion |
| 250 | Dinnes, J.  Bamber, J.  Chuchu, N.  Bayliss, S. E.  Takwoingi, Y.  Davenport, C.  et al., | 2018 | High‐frequency ultrasound for diagnosing skin cancer in adults | Title-abstract exclusion |
| 251 | Dinnes, J.  Deeks, J. J.  Chuchu, N.  Ferrante di Ruffano, L.  et al., | 2018 | Dermoscopy, with and without visual inspection, for diagnosing melanoma in adults | Title-abstract exclusion |
| 252 | Dinnes, J.  Deeks, J. J.  Chuchu, N.  Ferrante di Ruffano, L.  et al., | 2018 | Reflectance confocal microscopy for diagnosing keratinocyte skin cancers in adults | Title-abstract exclusion |
| 253 | Dinnes, J.  Deeks, J. J.  Grainge, M. J.  Chuchu, N.  et al., | 2018 | Visual inspection for diagnosing cutaneous melanoma in adults | Title-abstract exclusion |
| 254 | Dinnes, J.  Deeks, J. J.  Saleh, D.  Chuchu, N.  Bayliss, S. E.  et al., | 2018 | Reflectance confocal microscopy for diagnosing cutaneous melanoma in adults | Title-abstract exclusion |
| 255 | Dinnes, J.  Matin, R. N.  Webster, A. C.  Lawton, P.  Chuchu, N.  Bayliss, S. E.  Takwoingi, Y.  Davenport, C.  Godfrey, K.  O'Sullivan, C.  et al., | 2017 | Tests to assist in the staging of cutaneous squamous cell carcinoma: a generic protocol | Title-abstract exclusion |
| 256 | Dlugosz, Aldona  Zakikhany, Katherina  Muschiol, Sandra  Hultenby, Kjell  Lindberg, Greger | 2011 | Infection of Human Enteroendocrine Cells With C. Trachomatis as a Model for Irritable Bowel Syndrome | Title-abstract exclusion |
| 257 | Doan, H. Q.  Nguyen, H. P.  Rady, P.  Tyring, S. K. | 2015 | Expression patterns of immune-associated genes in external genital and perianal warts treated with sinecatechins | Title-abstract exclusion |
| 258 | Dolina, Marina Y.  Cornish, Duane C.  Merritt, Scott A.  Rai, Lav  Mahraj, Rickhesvar  Higgins, William E.  Bascom, Rebecca | 2008 | Interbronchoscopist variability in endobronchial path selection - A simulation study | Title-abstract exclusion |
| 259 | Dolmans, Valerie E. M. G.  Schout, Barbara M. A.  de Beer, Nicole A. M.  Bemelmans, Bart L. H.  Scherpbier, Albert J. J. A.  Hendrikx, Ad J. M. | 2009 | The Virtual Reality Endourologic Simulator Is Realistic and Useful for Educational Purposes | Title-abstract exclusion |
| 260 | Dominguez-Velasco, Cesar F.  Tello-Mata, Isaac E.  Guinto-Nishimura, Gerardo  Martinez-Hernandez, Adriana  Alcocer-Barradas, Victor  Perez-Lomeli, Juan S.  Padilla-Castaneda, Miguel A. | 2023 | Augmented reality simulation as training model of ventricular puncture: Evidence in the improvement of the quality of punctures | Title-abstract exclusion |
| 261 | Dong, Ni  Wing Yin, Chan  Jing, Qin  Yim-Pan, Chui  Ingrid, Qu  Ho, S. S.  Pheng-Ann, Heng | 2011 | A virtual reality simulator for ultrasound-guided biopsy training | Title-abstract exclusion |
| 262 | Donofrio, M.  Gallotti, A.  Pozzi Mucelli, R. | 2010 | Imaging techniques in pancreatic tumors | Title-abstract exclusion |
| 263 | Drahota, A.  Ward, D.  Mackenzie, H.  Stores, R.  Higgins, B.  Gal, D.  Dean, T. P. | 2012 | Sensory environment on health‐related outcomes of hospital patients | Title-abstract exclusion |
| 264 | Driver, V. R.  Yao, M.  Kantarci, A.  Gu, G.  Park, N.  Hasturk, H. | 2013 | A prospective, randomized clinical study evaluating the effect of transdermal continuous oxygen therapy on biological processes and foot ulcer healing in persons with diabetes mellitus | Title-abstract exclusion |
| 265 | Drks, | 2024 | Evaluation of a playful VR intervention for children and their caregivers to reduce pain, anxiety, and stress during painful procedures | Title-abstract exclusion |
| 266 | Du, K. L.  Septans, A. L.  Maloisel, F.  Vanquaethem, H.  Schmitt, A.  Goff, M. L.  Moles-Moreau, M. P.  Zinger, M.  Bourgeois, H.  Peron, M.  et al., | 20 | A new option in pain prevention with blissc, a therapeutic virtual reality solution in bone marrow context: results of a french open-label multicenter randomized phase ii/iii study (reveh trial) | Conference proceeding |
| 267 | Dubois, E.  Nigay, L.  Troccaz, J.  Carrat, L.  Chavanon, O. | 2001 | A methodological tool for computer-assisted surgery interface design: Its application to computer-assisted pericardial puncture | Title-abstract exclusion |
| 268 | Duraes, Martha  Crochet, Patrice  Pages, Emmanuelle  Grauby, Elsa  Lasch, Lidia  Rebel, Lucie  Van Meer, Frederick  Rathat, Gauthier | 2019 | Surgery of nonpalpable breast cancer: First step to a virtual per-operative localization? First step to virtual breast cancer localization | Title-abstract exclusion |
| 269 | Duriez, C.  Lamy, D.  Chaillou, C. | 2001 | A parallel manipulator as a haptic interface solution for amniocentesis simulation | Title-abstract exclusion |
| 270 | Ebert, L. C.  Flach, P.  Schweitzer, W.  Leipner, A.  Kottner, S.  Gascho, D.  Thali, M. J.  Breitbeck, R. | 2016 | Forensic 3D surface documentation at the Institute of Forensic Medicine in Zurich - Workflow and communication pipeline | Title-abstract exclusion |
| 271 | Ebert, L. C.  Ptacek, W.  Naether, S.  Fürst, M.  Ross, S.  Buck, U.  Weber, S.  Thali, M. | 2010 | Virtobot - A multi-functional robotic system for 3D surface scanning and automatic post mortem biopsy | Title-abstract exclusion |
| 272 | Edery, E. G.  Scase, T.  Kisielewicz, C.  Dhumeaux, M. P. | 2018 | Comparison of standard single-bite with multiple-bite biopsy forceps for collection of gastrointestinal biopsies in dogs: a prospective study | Title-abstract exclusion |
| 273 | Eggers, G.  Sudra, G.  Ghanai, S.  Salb, T.  Dillmann, R.  Marmulla, R.  Hassfeld, S. | 2005 | Augmented Reality Guided Biopsy of a Tumour near the Skull Base - The Surgeons Experience | Title-abstract exclusion |
| 274 | Ekman, M.  Girnyi, S.  Marano, L.  Roviello, F.  Chand, M.  Diana, M.  Polom, K. | 2022 | Near-Infrared Fluorescence Image-Guided Surgery in Esophageal and Gastric Cancer Operations | Title-abstract exclusion |
| 275 | El-Domyati, M.  Hosam, W.  Nasef, G.  Abdel-Aziz, R. T. A.  Raouf, Y. | 2019 | Treatment of nevus of Ota by Q switched Nd: YAG laser: A histologic and histometric study | Title-abstract exclusion |
| 276 | Elefsiniotis, I.  Buti, M.  Jardi, R.  Vezali, E.  Esteban, R. | 2009 | Clinical outcome of lamivudine-resistant chronic hepatitis B patients with compensated cirrhosis under adefovir salvage treatment. Importance of HCC surveillance | Title-abstract exclusion |
| 277 | Elefsiniotis, I. S.  Pantazis, K. D.  Mavrogiannis, C.  Magaziotou, I.  Bogris, P. | 2004 | Virological response in post-partum treated chronic hepatitis C women with pegylated interferon-alpha plus ribavirin - A case-control study | Title-abstract exclusion |
| 278 | El-Haddad, Mohamed T.  Tao, Yuankai K. | 2017 | Advances in intraoperative optical coherence tomography for surgical guidance | Title-abstract exclusion |
| 279 | El-Monajjed, Khaled  Driscoll, Mark | 2021 | Analysis of Surgical Forces Required to Gain Access Using a Probe for Minimally Invasive Spine Surgery via Cadaveric-Based Experiments Towards Use in Training Simulators | Title-abstract exclusion |
| 280 | Elwagdy, S. | 2010 | Editorial comment to real-time Virtual Sonography for navigation of prostate-targeted biopsy by using magnetic resonance imaging data | Title-abstract exclusion |
| 281 | Elzeky, M. E. H.  Salameh, B.  Reshia, F. A. A.  Sabry, A. A.  Shahine, N. F. M.  Mohamed, E. A. | 2024 | The effect of virtual reality distraction on haemodialysis patients’ pain and anxiety during arteriovenous fistula puncture: a randomised controlled trial | Title-abstract exclusion |
| 282 | Engelen, Thijs  Winkel, Beatrice Mf  Rietbergen, Daphne Dd  KleinJan, Gijs H.  Vidal-Sicart, Sergi  Olmos, Renato A. Valdes  van den Berg, Nynke S.  van Leeuwen, Fijs Wb | 2015 | The next evolution in radioguided surgery: breast cancer related sentinel node localization using a freehandSPECT-mobile gamma camera combination | Title-abstract exclusion |
| 283 | Engelhorn, A. L. D. V.  Engelhorn, C. A.  Salles-Cunha, S. X. | 2015 | Initial evaluation of virtual histology ultrasonographic techniques applied to a case of renal transplant | Title-abstract exclusion |
| 284 | Engum, S. A.  Jeffries, P.  Fisher, L. | 2003 | Intravenous catheter training system: Computer-based education versus traditional learning methods | Title-abstract exclusion |
| 285 | Enislidis, G.  Wagner, A.  Ploder, O.  Ewers, R | 1997 | Computed intraoperative navigation guidance - A preliminary report on a new technique | Title-abstract exclusion |
| 286 | Enns, R.  Romagnuolo, J.  Ponich, T.  Springer, J.  Armstrong, D.  Barkun, A. N. | 2008 | Canadian credentialing guidelines for flexible sigmoidoscopy | Title-abstract exclusion |
| 287 | Enquobahrie, A.  Horvath, S.  Arikatla, S.  Rosenberg, A.  Cleary, K.  Sharma, K. | 2019 | Development and face validation of ultrasound-guided renal biopsy virtual trainer | Title-abstract exclusion |
| 288 | Erdogan, B.  Aytekin Ozdemir, A. | 2021 | The Effect of Three Different Methods on Venipuncture Pain and Anxiety in Children: distraction Cards, Virtual Reality, and Buzzy® (Randomized Controlled Trial) | Title-abstract exclusion |
| 289 | Essig, H.  Rana, M.  Kokemueller, H.  Von See, C.  Ruecker, M.  Tavassol, F.  Gellrich, N. C. | 2011 | Pre-operative planning for mandibular reconstruction - A full digital planning workflow resulting in a patient specific reconstruction | Title-abstract exclusion |
| 290 | Etlinger, Péter | 2021 | The Importance of Simulation Training in Pediatric Surgery | Title-abstract exclusion |
| 291 | Eto, M.  Tsukihara, H.  Kobayashi, E.  Kiguchi, K.  Sakuma, I.  Hashizume, M. | 2019 | In-vivo and in-vitro mechanical properties of pig liver during needle puncture for needle insertion | Title-abstract exclusion |
| 292 | Euctr, H. U. | 2008 | A Randomised, Open-Label, Multicentre Phase 3 Study of the Combination of Rituximab, Cyclophosphamide, Doxorubicin, VELCADE, and Prednisone (VcR-CAP) or Rituximab, Cyclophosphamide, Doxorubicin, Vincristine, and Prednisone (R-CHOP) in Patients With Newly Diagnosed Mantle Cell Lymphoma who are not Eligible for a Bone Marrow Transplant - NA | Title-abstract exclusion |
| 293 | Euctr, I. T. | 2022 | VEMURAFENIB + RITUXIMAB (VR) AS A CHEMOTHERAPY-FREE ALTERNATIVE TO CLADRIBINE + RITUXIMAB (CDAR) IN FRONT-LINE HAIRY CELL LEUKEMIA (HCL): a PHASE-2 RANDOMIZED MULTICENTER TRIAL | Title-abstract exclusion |
| 294 | Eyrich, N. W.  Andino, J. J.  Ukavwe, R. E.  Farha, M. W.  Patel, A. K.  Triner, D.  Ellimoottil, C. | 2022 | The Lack of a Physical Exam During New Patient Telehealth Visits Does Not Impact Plans for Office and Operating Room Procedures | Title-abstract exclusion |
| 295 | Fadlelseed, H.  Rhatigan, M.  Treacy, M.  Murphy, C.  O'Neill, J.  Kilmartin, D.  Kennedy, S. | 2024 | Vitreoretinal large B- cell lymphoma (VR- LBCL): Clinical and pathological features and treatment outcomes | Title-abstract exclusion |
| 296 | Faerber, M.  Hummel, F.  Gerloff, C.  Handels, H. | 2009 | Virtual Reality Simulator for the Training of Lumbar Punctures | Title-abstract exclusion |
| 297 | Faiella, E.  Frauenfelder, G.  Santucci, D.  Luppi, G.  Schena, E.  Beomonte Zobel, B.  Grasso, R. F. | 2018 | Percutaneous low-dose CT-guided lung biopsy with an augmented reality navigation system: validation of the technique on 496 suspected lesions | Title-abstract exclusion |
| 298 | Falavigna, A.  Guiroy, A.  Taboada, N. | 2020 | Teaching Training and Surgical Education in Minimally Invasive Surgery (MIS) of the Spine: What Are the Best Teaching and Learning Strategies for MIS? Do We Have Any Experience and Data? | Title-abstract exclusion |
| 299 | Fallahi, B.  Saghari, M.  Fard Esfahani, A.  Eftekhari, M.  Iravani, M.  Beiki, D.  Dabbagh Kakhki, V. R.  Sadeghi, R. | 2005 | The value of 99mTc-MIBI whole body scintigraphy in active and in remission multiple myeloma | Title-abstract exclusion |
| 300 | Färber, M.  Dahmke, T.  Bohn, C. A.  Handels, H. | 2009 | Needle bending in a VR-puncture training system using a 6DOF haptic device | Title-abstract exclusion |
| 301 | Färber, M.  Dalek, D.  Habermann, C. R.  Hummel, F.  Schöps, C.  Handels, H. | 2009 | A framework for visuo-haptic simulation of puncture interventions | Title-abstract exclusion |
| 302 | Färber, M.  Heller, J.  Handels, H. | 2007 | Simulation and training of lumbar punctures using haptic volume rendering and a 6DOF haptic device | Title-abstract exclusion |
| 303 | Färber, M.  Hoeborn, E.  Dalek, D.  Hummel, F.  Gerloff, C.  Bohn, C. A.  Handels, H. | 2008 | Training and evaluation of lumbar punctures in a VR-environment using a 6DOF haptic device | Title-abstract exclusion |
| 304 | Färber, M.  Hummel, F.  Gerloff, C.  Handels, H. | 2009 | Virtual reality simulator for the training of lumbar punctures | Title-abstract exclusion |
| 305 | Faruqz, F.  Kachrilas, S.  Bach, C.  Masood, J.  Buchholz, N.  Junaid, I. | 2011 | Enhancing trainee's skills by the virtual reality (VR) PERC-Mentor trainer | Title-abstract exclusion |
| 306 | Faso, Andrea | 2017 | Haptic and Virtual Reality Surgical Simulator for Training in Percutaneous Renal Access | Title-abstract exclusion |
| 307 | Fastner, C.  Behnes, M.  Kuschyk, J.  Akin, I. | 2015 | A plea for the single-lead ICD with atrial sensing due to anatomical considerations | Title-abstract exclusion |
| 308 | Feinmesser, Gilad  Yogev, David  Goldberg, Tomer  Parmet, Yisrael  Illouz, Shay  Vazgovsky, Oliana  Eshet, Yael  Tejman-Yarden, Shai  Alon, Eran | 2023 | Virtual reality-based training and pre-operative planning for head and neck sentinel lymph node biopsy | Title-abstract exclusion |
| 309 | Feng, Lei  Li, Wei  Lai, Jianming  Yang, Weihao  Wu, Shangxing  Liu, Jiajia  Ma, Ruixin  Lee, Soomin  Tian, Jing | 2024 | Validity of A Novel Simulator for Percutaneous Transforaminal Endoscopic Discectomy | Title-abstract exclusion |
| 310 | Feng, S. T.  Li, Z. P.  Cui, M. Y.  Meng, Q. F.  Zhou, X. H.  Peng, Z. P.  Sun, C. H.  Fan, M. | 2006 | The clinical application of 64-slice spiral CT angiography in abdominal tumors | Title-abstract exclusion |
| 311 | Fenici, R.  Brisinda, D. | 2007 | Percutaneous method for multiple epicardial monophasic action potential recordings during magnetocardiographic mapping in intact rats | Title-abstract exclusion |
| 312 | Ferraguti, F.  Farsoni, S.  Bonfè, M. | 2022 | Augmented Reality and Robotic Systems for Assistance in Percutaneous Nephrolithotomy Procedures: Recent Advances and Future Perspectives | Title-abstract exclusion |
| 313 | Ferraguti, Federica  Minelli, Marco  Farsoni, Saverio  Bazzani, Stefano  Bonfe, Marcello  Vandanjon, Alexandre  Puliatti, Stefano  Bianchi, Giampaolo  Secchi, Cristian | 2020 | Augmented Reality and Robotic-Assistance for Percutaneous Nephrolithotomy | Title-abstract exclusion |
| 314 | Ferrante di Ruffano, L.  Dinnes, J.  Chuchu, N.  Bayliss, S. E.  Takwoingi, Y.  et al., | 2018 | Exfoliative cytology for diagnosing basal cell carcinoma and other skin cancers in adults | Title-abstract exclusion |
| 315 | Ferrante di Ruffano, L.  Takwoingi, Y.  Dinnes, J.  Chuchu, N.  Bayliss, S. E.  et al., | 2018 | Computer‐assisted diagnosis techniques (dermoscopy and spectroscopy‐based) for diagnosing skin cancer in adults | Title-abstract exclusion |
| 316 | Ferrari, V.  Ferrari, M.  Mosca, F. | 2011 | Video see-through in the clinical practice | Title-abstract exclusion |
| 317 | Ferrari, Vincenzo  Viglialoro, Rosanna Maria  Nicoli, Paola  Cutolo, Fabrizio  Condino, Sara  Carbone, Marina  Siesto, Mentore  Ferrari, Mauro | 2016 | Augmented reality visualization of deformable tubular structures for surgical simulation | Title-abstract exclusion |
| 318 | Ferreira, Elaine Barros  Cruz, Flávia Oliveira de Almeida Marques da  Silveira, Renata Cristina de Campos Pereira  Reis, Paula Elaine Diniz dos | 2015 | Distraction methods for pain relief of cancer children submitted to painful procedures: systematic review | Title-abstract exclusion |
| 319 | Ferrell, A. | 2022 | 25th IFSO World Congress Silver Anniversary | Title-abstract exclusion |
| 320 | Ferretti, G.  Coulomb, M. | 2000 | 3D virtual imaging of the upper airways | Title-abstract exclusion |
| 321 | Fiard, G.  Selmi, S. Y.  Promayon, E.  Descotes, J. L.  Troccaz, J. | 2020 | Simulation-based training for prostate biopsies: towards the validation of the Biopsym simulator | Title-abstract exclusion |
| 322 | Fiard, G.  Selmi, S. Y.  Promayon, E.  Vadcard, L.  Descotes, J. L.  Troccaz, J. | 2014 | Initial validation of a virtual-reality learning environment for prostate biopsies: Realism matters! | Title-abstract exclusion |
| 323 | Fiard, Gaelle  Selmi, Sonia-Yuki  Promayon, Emmanuel  Descotes, Jean-Luc  Troccaz, Jocelyne | 2020 | Simulation-based training for prostate biopsies: towards the validation of the Biopsym simulator | Title-abstract exclusion |
| 324 | Fiard, Gaelle  Selmi, Sonia-Yuki  Promayon, Emmanuel  Vadcard, Lucile  Descotes, Jean-Luc  Troccaz, Jocelyne | 2014 | Initial Validation of a Virtual-Reality Learning Environment for Prostate Biopsies: Realism Matters! | Title-abstract exclusion |
| 325 | Fichtinger, G.  Deguet, A.  Masamune, K.  Balogh, E.  Fischer, G. S.  Mathieu, H.  Taylor, R. H.  Zinreich, S. J.  Fayad, L. M. | 2005 | Image overlay guidance for needle insertion in CT scanner | Title-abstract exclusion |
| 326 | Fichtner, J.  Raabe, C.  Beck, J.  Gralla, J.  Raabe, A. | 2017 | Revisiting the rules for freehand ventriculostomy: A virtual reality analysis | Title-abstract exclusion |
| 327 | Figueiredo, R. J.  Backman, V.  Liu, Y.  Paladugula, J. | 2007 | Architecture and performance of a grid-enabled lookup-based biomedical optimization application: Light scattering spectroscopy | Title-abstract exclusion |
| 328 | Filippou, Pauline  Odisho, Anobel  Ramaswamy, Krishna  Usawachintachit, Manint  Hu, Weiguo  Li, Jianxing  Chi, Thomas | 2016 | Using an abdominal phantom to teach urology residents ultrasound-guided percutaneous needle placement | Title-abstract exclusion |
| 329 | Filograna, L.  Pugliese, L.  Muto, M.  Tatulli, D.  Guglielmi, G.  Thali, M. J.  Floris, R. | 2019 | A Practical Guide to Virtual Autopsy: Why, When and How | Title-abstract exclusion |
| 330 | Finger, T.  Schaumann, A.  Schulz, M.  Thomale, U. W. | 2017 | Augmented reality in intraventricular neuroendoscopy | Title-abstract exclusion |
| 331 | Finos, K.  Datta, S.  Sedrakyan, A.  Milsom, J. W.  Pua, B. B. | 2024 | Mixed reality in interventional radiology: a focus on first clinical use of XR90 augmented reality-based visualization and navigation platform | Title-abstract exclusion |
| 332 | Fior, D.  Vacirca, F.  Leni, D.  Pagni, F.  Ippolito, D.  Riva, L.  Sironi, S.  Corso, R. | 2019 | Virtual Guidance of Percutaneous Transthoracic Needle Biopsy with C-Arm Cone-Beam CT: Diagnostic Accuracy, Risk Factors and Effective Radiation Dose | Title-abstract exclusion |
| 333 | Fischer, H.  Selig, M.  Vagner, J.  Vogel, B.  Hempel, E.  Kaiser, M.  Brhel, K.  et al | 2000 | The medical engineering program of Forschungszentrum Karlsruhe | Title-abstract exclusion |
| 334 | Fischer, S.  MacLean, A. A.  Liu, M.  Kalirai, B.  Keshavjee, S. | 2000 | Inhibition of angiotensin-converting enzyme by captopril: A novel approach to reduce ischemia-reperfusion injury after lung transplantation | Title-abstract exclusion |
| 335 | Fitzpatrick, J. M. | 2011 | Virtual issue on urological oncology available online | Title-abstract exclusion |
| 336 | Forest, C.  Comas, O.  Vaysière, C.  Soler, L.  Marescaux, J. | 2007 | Ultrasound and needle insertion simulators built on real patient-based data | Title-abstract exclusion |
| 337 | Foroughi, Pezhman  Demir, Alican  Hossbach, Martin  Rajan, Purnima  Yarmolenko, Pavel  Vellody, Ranjith  Cleary, Kevin  Sharma, Karun | 2023 | In situ guidance for MRI interventions using projected feedback | Title-abstract exclusion |
| 338 | Fortmeier, Dirk  Mastmeyer, Andre  Handels, Heinz | 2013 | Optimized image-based soft tissue deformation algorithms for visualization of haptic needle insertion | Title-abstract exclusion |
| 339 | Fortmeier, Dirk  Mastmeyer, Andre  Handels, Heinz | 2013 | Image-based Soft Tissue Deformation Algorithms for Real-time Simulation of Liver Puncture | Title-abstract exclusion |
| 340 | Fortmeier, D.  Mastmeyer, A.  Handels, H. | 2013 | Image-based palpation simulation with soft tissue deformations using chainmail on the GPU | Title-abstract exclusion |
| 341 | Fortmeier, D.  Mastmeyer, A.  Handels, H. | 2013 | Optimized image-based soft tissue deformation algorithms for visualization of haptic needle insertion | Title-abstract exclusion |
| 342 | Fortmeier, Dirk  Wilms, Matthias  Mastmeyer, Andre  Handels, Heinz | 2015 | Direct Visuo-Haptic 4D Volume Rendering Using Respiratory Motion Models | Title-abstract exclusion |
| 343 | Foss, G. L.  Tindall, V. R.  Birkett, J. P. | 1973 | The treatment of subfertile men with clomiphene citrate | Title-abstract exclusion |
| 344 | Fournier, Jean-Yves | 2018 | Endoscope in Cranial Neurosurgery | Title-abstract exclusion |
| 345 | Frauchiger, B.  Bock, A.  Eichlisberger, R.  Landmann, J.  Thiel, G.  Mihatsch, M. J.  Jäger, K. | 1995 | The value of different resistance parameters in distinguishing biopsy-proved dysfunction of renal allografts | Title-abstract exclusion |
| 346 | Freschi, C.  Troia, E.  Ferrari, V.  Megali, G.  Pietrabissa, A.  Mosca, F. | 2009 | Ultrasound guided robotic biopsy using augmented reality and human-robot cooperative control | Title-abstract exclusion |
| 347 | Frick, T. B.  Marucci, D. D.  Cartmill, J. A.  Martin, C. J.  Walsh, W. R. | 2001 | Resistance forces acting on suture needles | Title-abstract exclusion |
| 348 | Friedlander, J. A.  Fleischer, D. M.  Black, J. O.  Levy, M.  Rothenberg, M. E.  Smith, C.  Nguyen, N.  Pan, Z.  Furuta, G. T. | 2021 | Unsedated transnasal esophagoscopy with virtual reality distraction enables earlier monitoring of dietary therapy in eosinophilic esophagitis | Title-abstract exclusion |
| 349 | Fritz, Jan  U-Thainual, Paweena  Ungi, Tamas  Flammang, Aaron J.  McCarthy, Edward F.  Fichtinger, Gabor  Iordachita, Iulian I.  Carrino, John A. | 2013 | Augmented Reality Visualization Using Image Overlay Technology for MR-Guided Interventions <i>Cadaveric Bone Biopsy at 1</i>.<i>5 T</i> | Title-abstract exclusion |
| 350 | Frohn, J.  Pinkert-Leetsch, D.  Missbach-Guntner, J.  Reichardt, M.  Osterhoff, M.  Alves, F.  Salditt, T. | 2020 | 3D virtual histology of human pancreatic tissue by multiscale phase-contrast X-ray tomography | Title-abstract exclusion |
| 351 | Frommer, J. P.  Laski, M. E.  Wesson, D. E.  Kurtzman, N. A. | 1984 | Internephron heterogeneity for carbonic anhydrase-independent bicarbonate reabsorption in the rat | Title-abstract exclusion |
| 352 | Fu, Junling  Pecorella, Matteo  Iovene, Elisa  Palumbo, Maria Chiara  Rota, Alberto  Redaelli, Alberto  Ferrigno, Giancarlo  De Momi, Elena | 2024 | Augmented Reality and Human-Robot Collaboration Framework for Percutaneous Nephrolithotomy: System Design, Implementation, and Performance Metrics | Title-abstract exclusion |
| 353 | Fuchs, H. | 1996 | Toward virtual teleconferencing for telemedical applications | Title-abstract exclusion |
| 354 | Fuchs, H.  State, A.  Livingston, M.  Garrett, W.  Hirota, G.  Whitton, M.  Pisano, E. | 1996 | Virtual environments technology to aid needle biopsies of the breast. An example of real-time data fusion | Title-abstract exclusion |
| 355 | Fuerst, Bernhard  Sprung, Julian  Pinto, Francisco  Frisch, Benjamin  Wendler, Thomas  Simon, Herve  Mengus, Laurent  van den Berg, Nynke S.  van der Poel, Henk G.  van Leeuwen, Fijs W. B.  Navab, Nassir | 2016 | First Robotic SPECT for Minimally Invasive Sentinel Lymph Node Mapping | Title-abstract exclusion |
| 356 | Fujihara, Atsuko  Ukimura, Osamu | 2022 | Virtual reality of three-dimensional surgical field for surgical planning and intraoperative management | Title-abstract exclusion |
| 357 | Gabr, A. H.  Elbadry, M.  Elsherief, A.  Tawfiek, E. R. | 2013 | Computed tomography-virtual cystoscopy in the evaluation of a bladder mass: Could it replace standard conventional cystoscopy? | Title-abstract exclusion |
| 358 | Gadjiev, N. K.  Gorelov, D. S.  Mishchenko, A. A.  Britov, V. P.  Kharchilava, R. R.  Sharafutdinov, E. F.  Petrov, S. B.  Shkarupa, D. D. | 2023 | Comparative evaluation of simulators for practising fluoroscopy-guided renal pelvic puncture | Title-abstract exclusion |
| 359 | Gadzhiev, N. K.  Mishchenko, A. A.  Britov, V. P.  Khrenov, A. M.  Gorelov, D. S.  Obidnyak, V. M.  Grigoriev, V. E.  Semenyakin, I. V.  Petrov, S. B. | 2021 | Creation of a training simulator model for practising puncture of the kidney calyceal system under ultrasound control | Title-abstract exclusion |
| 360 | Ganpule, A. P.  Mishra, S.  Sabnis, R. B.  Muthu, V.  Desai, M. R. | 2009 | Evaluation and validation of virtual reality (VR) based simulation to develop endourological percutaneous renal access technique for urological trainees | Title-abstract exclusion |
| 361 | Gao, Lei  Xu, Yidi  Zhang, Xixue  Jiang, Zhaoshun  Wu, Jiajun  Dong, Yanjun  Li, Ming  Jin, Liang  Qiu, Jianjian  You, Lijue  Qin, Chunhui  Gu, Weidong | 2024 | Comparison of Mixed Reality-Assisted Spinal Puncture with Landmark-Guided Spinal Puncture by Novice Practitioners: A Pilot Study | Title-abstract exclusion |
| 362 | Gao, Yan  Xu, Yiwei  Liu, Naiquan  Fan, Ling | 2023 | Full title: Effectiveness of virtual reality intervention on reducing the pain, anxiety and fear of needle-related procedures in paediatric patients: A systematic review and meta-analysis | Title-abstract exclusion |
| 363 | Gao, Y.  Xu, Y.  Liu, N.  Fan, L. | 2023 | Effectiveness of virtual reality intervention on reducing the pain, anxiety and fear of needle-related procedures in paediatric patients: A systematic review and meta-analysis | Title-abstract exclusion |
| 364 | Gao, Z.  Pan, F.  Wang, J.  Pan, H.  Jiang, Z. | 2015 | Virtual needle insertion and haptic interaction | Title-abstract exclusion |
| 365 | García-Carpintero, E.  Naredo, E.  Vélez-Vélez, E.  Fuensalida, G.  Ortiz-Miluy, G.  Gómez-Moreno, C. | 2023 | Phantoms for ultrasound-guided vascular access cannulation training: a narrative review | Title-abstract exclusion |
| 366 | Garcia-Esteban, J. A.  Curto, B.  Moreno, V.  Hernandez, F.  Alonso, P.  Serrano, F. J.  Blanco, F. J. | 2024 | Real needle for minimal invasive procedures training using motion sensors and optical flow | Title-abstract exclusion |
| 367 | Garnon, J.  De Marini, P.  Meylheuc, L.  Dalili, D.  Cazzato, R. L.  Bayle, B.  Gangi, A. | 2020 | Percutaneous image-guided double oblique anterior approach to the acetabulum for cementoplasty | Title-abstract exclusion |
| 368 | Garrison, M. A.  Bailey, J. K.  Pollack, M. S.  Elston, D. M.  Libow, L.  Sheffler, R. L. | 2000 | Transfusion associated graft-versus-host disease in an apparently immunocompetent patient initially identified as toxic epidermal necrolysis | Title-abstract exclusion |
| 369 | Gärtner, H. V.  Rösch, C. H.  Wehrmann, M.  Fischer, S. | 1998 | Morphology and pathogenesis of chronic transplant nephropathy with special regard to transplant glomerulopathy | Title-abstract exclusion |
| 370 | Gärtner, V.  Eigentler, T. K.  Viebahn, R. | 2006 | Plasma cell-rich rejection processes in renal transplantation:: Morphology and prognostic relevance | Title-abstract exclusion |
| 371 | Gates, M.  Hartling, L.  Shulhan-Kilroy, J.  MacGregor, T.  Guitard, S.  et al | 2020 | Digital technology distraction for acute pain in children: A Meta-analysis | Title-abstract exclusion |
| 372 | Gauhar, Vineet  Giulioni, Carlo  Gadzhiev, Nariman  De Stefano, Virgilio  Teoh, Jeremy Yuen-Chun  Tiong, Ho Yee  Taguchi, Kazumi  Milanese, Giulio  Galosi, Andrea Benedetto  Somani, Bhaskar Kumar  Castellani, Daniele | 2023 | An Update of In Vivo Application of Artificial Intelligence and Robotics for Percutaneous Nephrolithotripsy: Results from a Systematic Review | Title-abstract exclusion |
| 373 | Geagea, Dali  Tyack, Zephanie  Kimble, Roy  Polito, Vince  Ayoub, Bassel  Terhune, Devin B.  Griffin, Bronwyn | 2023 | Clinical Hypnosis for Procedural Pain and Distress in Children: A Scoping Review | Title-abstract exclusion |
| 374 | Geiger, B.  Kikinis, R. | 1995 | Simulation of endoscopy | Title-abstract exclusion |
| 375 | Genç, H.  Korkmaz, M.  Akkurt, A. | 2022 | The Effect of Virtual Reality Glasses and Stress Balls on Pain and Vital Findings During Transrectal Prostate Biopsy: A Randomized Controlled Trial | Included |
| 376 | George, Sandeep  Kesavadas, Thenkurussi | 2008 | Low cost augmented reality for training of MRI-guided needle biopsy of the spine | Title-abstract exclusion |
| 377 | Ghaednia, Hamid  Fourman, Mitchell S.  Lans, Amanda  Detels, Kelsey  Dijkstra, Hidde  Lloyd, Sophie  Sweeney, Allison  Oosterhoff, Jacobien H. F.  Schwab, Joseph H. | 2021 | Augmented and virtual reality in spine surgery, current applications and future potentials | Title-abstract exclusion |
| 378 | Gibbs, J. D.  Graham, M. W.  Bascom, R.  Cornish, D. C.  Khare, R.  Higgins, W. E. | 2014 | Optimal procedure planning and guidance system for peripheral bronchoscopy | Title-abstract exclusion |
| 379 | Gibby, Jacob T.  Swenson, Samuel A.  Cvetko, Steve  Rao, Raj  Javan, Ramin | 2019 | Head-mounted display augmented reality to guide pedicle screw placement utilizing computed tomography | Title-abstract exclusion |
| 380 | Gibby, Wendell  Cvetko, Steve  Gibby, Andrew  Gibby, Conrad  Sorensen, Kiel  Andrews, Edward G.  Maroon, Joseph  Parr, Ryan | 2022 | The application of augmented reality-based navigation for accurate target acquisition of deep brain sites: advances in neurosurgical guidance | Title-abstract exclusion |
| 381 | Gil Piquer, R.  Mañes Jiménez, Y.  España Marí, M.  Peris Peris, A.  Solanes Donet, P.  García Lledó, N.  Pons Fernández, N. | 2024 | Usefulness of virtual reality in the management of pain associated with venepuncture: A multicentre randomized clinical trial | Title-abstract exclusion |
| 382 | Gilboa Pras, Y.  Indursky, A.  Gilboa Pras, S.  Schaffer, O.  Niazov, E.  Zmora, O. | 2024 | Infectious Diseases-Approved Virtual Reality Goggles for Common Procedures in Pediatric Surgical Patients | Title-abstract exclusion |
| 383 | Giovannini, M.  Bories, E.  Monges, G.  Pesenti, C.  Caillol, F.  Delpero, J. R. | 2011 | Results of a phase I-II study on intraductal confocal microscopy (IDCM) in patients with common bile duct (CBD) stenosis | Title-abstract exclusion |
| 384 | Giovannini, M.  Hookey, L. C.  Bories, E.  Pesenti, C.  Monges, G.  Delpero, J. R. | 2006 | Endoscopic ultrasound elastography: The first step towards virtual biopsy? Preliminary results in 49 patients | Title-abstract exclusion |
| 385 | Gkali, C. A.  Chalazonitis, A. N.  Feida, E.  Sotiropoulou, M.  Giannos, A.  Tsigginou, A.  Dimitrakakis, C. | 2015 | Breast elastography: How we do it | Title-abstract exclusion |
| 386 | Glennon, C.  McElroy, S. F.  Connelly, L. M.  Mische Lawson, L.  Bretches, A. M.  Gard, A. R.  Newcomer, L. R. | 2018 | Use of Virtual Reality to Distract From Pain and Anxiety | Quasi-experimental study |
| 387 | Glielmo, Pierluigi  Fusco, Stefano  Gitto, Salvatore  Zantonelli, Giulia  Albano, Domenico  Messina, Carmelo  Sconfienza, Luca Maria  Mauri, Giovanni | 2024 | Artificial intelligence in interventional radiology: state of the art | Title-abstract exclusion |
| 388 | Goergen, D. I.  Freitas, D. M. O. | 2022 | Virtual Reality as a distraction therapy during cystoscopy: a clinical trial | Title-abstract exclusion |
| 389 | Gold, J. I.  Akbar, K. M.  Avila, S.  Ngo, N. H.  Klein, M. J. | 2024 | Exploring Relations Between Unique Patient Characteristics and Virtual Reality Immersion Level on Anxiety and Pain in Patients Undergoing Venipuncture: Secondary Analysis of a Randomized Control Trial | Title-abstract exclusion |
| 390 | Gold, J. I.  Kant, A. J.  Kim, S. H.  Rizzo, A. | 2005 | Virtual anesthesia: The use of virtual reality for pain distraction during acute medical interventions | Title-abstract exclusion |
| 391 | Gong, Juan Wen | 2007 | Solving collision detection using algebraic methods | Title-abstract exclusion |
| 392 | González-Gómez, R.  Pazo-Cid, R. A.  Sarría, L.  Morcillo, M. Á  Schuhmacher, A. J. | 2021 | Diagnosis of pancreatic ductal adenocarcinoma by immuno-positron emission tomography | Title-abstract exclusion |
| 393 | Goodsitt, M. M.  Chan, H. P.  Hadjiiski, L. | 2000 | Stereomammography: Evaluation of depth perception using a virtual 3D cursor | Title-abstract exclusion |
| 394 | Gorman, P.  Krummel, T.  Webster, R.  Smith, M.  Hutchens, D. | 2000 | A prototype haptic lumbar puncture simulator | Title-abstract exclusion |
| 395 | Goud, A.  Dahagam, C.  Breen, D. P.  Sarkar, S. | 2016 | Role of electromagnetic navigational bronchoscopy in pulmonary nodule management | Title-abstract exclusion |
| 396 | Graber, M. A.  Pierre, J.  Charlton, M. | 2003 | Patient opinions and attitudes toward medical student procedures in the emergency department | Title-abstract exclusion |
| 397 | Grasso, R. F.  Faiella, E.  Luppi, G.  Schena, E.  Giurazza, F.  Del Vescovo, R.  D'Agostino, F.  Cazzato, R. L.  Zobel, B. Beomonte | 2013 | Percutaneous lung biopsy: comparison between an augmented reality CT navigation system and standard CT-guided technique | Title-abstract exclusion |
| 398 | Grasso, Rosario Francesco  Luppi, Giacomo  Cazzato, Roberto Luigi  Faiella, Eliodoro  D'Agostino, Francesco  Zobel, Daniela Beomonte  De Lena, Mario | 2012 | Percutaneous computed tomography-guided lung biopsies: preliminary results using an augmented reality navigation system | Title-abstract exclusion |
| 399 | Grober, Ethan D.  Blankstein, Udi | 2024 | Testosterone therapy at the time of vasectomy reversal: Impact on intraoperative decision-making and interpretation of postoperative outcomes | Title-abstract exclusion |
| 400 | Grosch, Anne Sophie  Schroeder, Timo  Schroeder, Torsten  Onken, Julia  Picht, Thomas | 2020 | Development and initial evaluation of a novel simulation model for comprehensive brain tumor surgery training | Title-abstract exclusion |
| 401 | Groves, Leah  Li, Natalie  Peters, Terry M.  Chen, Elvis C. S. | 2022 | Towards a First-Person Perspective Mixed Reality Guidance System for Needle Interventions | Title-abstract exclusion |
| 402 | Guo, J.  Li, H.  Chen, Y.  Chen, P.  Li, X.  Sun, S. | 2019 | Robotic ultrasound and ultrasonic robot | Title-abstract exclusion |
| 403 | Guo, Zhaoxiang  Tai, Yonghang  Du, Junzhen  Chen, Zaiqing  Li, Qiong  Shi, Junsheng | 2021 | Automatically Addressing System for Ultrasound-Guided Renal Biopsy Training Based on Augmented Reality | Title-abstract exclusion |
| 404 | Guo, Zhaoxiang  Tai, Yonghang  Qin, Zhibao  Huang, Xiaoqiao  Li, Qiong  Peng, Jun  Shi, Junsheng | 2020 | Development and assessment of a haptic-enabled holographic surgical simulator for renal biopsy training | Title-abstract exclusion |
| 405 | Gupta, Sandeep K.  Lewis, Guy  Rogers, Kerry M.  Attia, John  Rostron, Kirk  O'Neill, Leanne  Skillen, Annah  Viswanathan, Suresh | 2014 | Quantitative (99m)Tc DTPA renal transplant scintigraphic parameters: assessment of interobserver agreement and correlation with graft pathologies | Title-abstract exclusion |
| 406 | Gutmann, Sarah  Heiderhoff, Miriam  Moebius, Robert  Siegel, Tanja  Flegel, Thomas | 2023 | Application accuracy of a frameless optical neuronavigation system as a guide for craniotomies in dogs | Title-abstract exclusion |
| 407 | Habeck, C.  Whitwell, J. L. | 2013 | Structural brain imaging and multivariate analysis enable virtual lumbar punctures | Title-abstract exclusion |
| 408 | Haddad, R.  Mattei, A.  Giovanni, A. | 2023 | Local anesthesia with blue-dyed lidocaine for a better patient’s tolerance during office-based laryngology procedures: how I do it | Title-abstract exclusion |
| 409 | Hagmann, E.  Rouiller, P.  Helmer, P.  Grange, S.  Baur, C. | 2004 | A haptic guidance tool for CT-directed percutaneous interventions | Title-abstract exclusion |
| 410 | Hall, A. R.  Tsochatzis, E.  Morris, R.  Burroughs, A. K.  Dhillon, A. P. | 2013 | Sample size requirement for digital image analysis of collagen proportionate area in cirrhotic livers | Title-abstract exclusion |
| 411 | Hammarstedt, A.  Sopasakis, V. R.  Gogg, S.  Jansson, P. A.  Smith, U. | 2005 | Improved insulin sensitivity and adipose tissue dysregulation after short-term treatment with pioglitazone in non-diabetic, insulin-resistant subjects | Title-abstract exclusion |
| 412 | Hampel, H.  Shaw, L. M.  Aisen, P.  Chen, C.  Lleó, A.  Iwatsubo, T.  Iwata, A.  et al | 2022 | State-of-the-art of lumbar puncture and its place in the journey of patients with Alzheimer's disease | Title-abstract exclusion |
| 413 | Han, D.  Yu, Y.  He, T.  Yu, N.  Dang, S.  Wu, H.  Ren, J.  Duan, X. | 2021 | Effect of radiomics from different virtual monochromatic images in dual-energy spectral CT on the WHO/ISUP classification of clear cell renal cell carcinoma | Title-abstract exclusion |
| 414 | Hanaway, M. J.  Woodle, E. S.  Mulgaonkar, S.  Peddi, V. R.  Kaufman, D. B.  First, M. R.  Croy, R.  Holman, J. | 2011 | Alemtuzumab induction in renal transplantation | Title-abstract exclusion |
| 415 | Hann, A.  Walter, B.  Schmidt, S.  Meining, A. | 20 | Application of virtual reality in endoscopy | Title-abstract exclusion |
| 416 | Hanna, W. C. | 2019 | The Dawn of the Age of Virtual Biopsies | Title-abstract exclusion |
| 417 | Hao, J.  Li, T.  Heinzelmann, M.  Moussaud-Lamodière, E.  Lebre, F.  Krjutškov, K.  Damdimopoulos, A.  Arnelo, C.  Pettersson, K.  Alfaro-Moreno, E.  Lindskog, C.  Van Duursen, M.  Damdimopoulou, P. | 2024 | Effects of chemical in vitro activation versus fragmentation on human ovarian tissue and follicle growth in culture | Title-abstract exclusion |
| 418 | Harder, C.  Pryalukhin, A.  Quaas, A.  Eich, M. L.  Tretiakova, M.  Klein, S.  Seper, A.  Heidenreich, A.  Netto, G. J.  Hulla, W.  Büttner, R.  Bozek, K.  Tolkach, Y. | 2022 | Enhancing Prostate Cancer Diagnosis: Artificial intelligence-Driven Virtual Biopsy for Optimal Magnetic Resonance Imaging-Targeted Biopsy Approach and Gleason Grading Strategy | Title-abstract exclusion |
| 419 | Harper, H. E.  Hirt, P. A.  Lev-Tov, H. | 2022 | The use of virtual reality in non-burn dermatological care–a review of the literature | Title-abstract exclusion |
| 420 | Hasan, D. I.  Almassry, H. N. | 2010 | Magnetic resonance cholangiopancreatography in conjunction with 3D for assessment of different biliary obstruction causes | Title-abstract exclusion |
| 421 | Hatzl, J.  Henning, D.  Böckler, D.  Hartmann, N.  Meisenbacher, K.  Uhl, C. | 2024 | Comparing Different Registration and Visualization Methods for Navigated Common Femoral Arterial Access—A Phantom Model Study Using Mixed Reality | Title-abstract exclusion |
| 422 | Hatzl, Johannes  Henning, Daniel  Hartmann, Niklas  Boeckler, Dittmar  Uhl, Christian | 2023 | A New Method for Common Femoral Arterial Access Using a Mixed Reality-Assisted Technique on a Phantom Model | Title-abstract exclusion |
| 423 | Hawks, J. A.  Rentschler, M. E.  Farritor, S.  Oleynikov, D.  Platt, S. R. | 2009 | A modular wireless in vivo surgical robot with multiple surgical applications | Title-abstract exclusion |
| 424 | Hawks, J. A.  Rentschler, M. E.  Redden, L.  Infanger, R.  Dumpert, J.  Farritor, S.  Oleynikov, D.  Platt, S. R. | 2008 | Towards an in vivo wireless mobile robot for surgical assistance | Title-abstract exclusion |
| 425 | Hayashi, Sanae  Isogawa, Masanori  Kawashima, Keigo  Ito, Kyoko  Chuaypen, Natthaya  Morine, Yuji  Shimada, Mitsuo  Higashi-Kuwata, Nobuyo  Watanabe, Takehisa  Tangkijvanich, Pisit  Mitsuya, Hiroaki  Tanaka, Yasuhito | 2022 | Droplet digital PCR assay provides intrahepatic HBV cccDNA quantification tool for clinical application | Title-abstract exclusion |
| 426 | He, T.  Xue, Z.  Xie, W.  Wong, S.  Wong, K.  Alvarado, M. V. Y.  Wong, S. T. C. | 2010 | A motion correction algorithm for microendoscope video computing in image-guided intervention | Title-abstract exclusion |
| 427 | Healey, A. E.  Evans, J. C.  Murphy, M. G.  Powell, S.  How, T. V.  Groves, D.  Hatfield, F.  Diaz, B. M.  Gould, D. A. | 2005 | In vivo force during arterial interventional radiology needle puncture procedures | Title-abstract exclusion |
| 428 | Healy, D. A.  Murphy, S. P.  Burke, J. P.  Coffey, J. C. | 2013 | Artificial interfaces ("AI") in surgery: Historic development, current status and program implementation in the public health sector | Title-abstract exclusion |
| 429 | Hecken, Julia M. M.  Halagiera, Pia  Rehman, Sadia  Tempfer, Clemens B. B.  Rezniczek, Gunther A. | 2023 | Virtual Reality for Anxiety Reduction in Women Undergoing Colposcopy: A Randomized Controlled Trial | Title-abstract exclusion |
| 430 | Heidari, Houtan  Kanschik, Dominika  Maier, Oliver  Wolff, Georg  Brockmeyer, Maximilian  Masyuk, Maryna  et al | 2024 | A comparison of conventional and advanced 3D imaging techniques for percutaneous left atrial appendage closure | Title-abstract exclusion |
| 431 | Heinrich, Florian  Joeres, Fabian  Lawonn, Kai  Hansen, Christian | 2019 | Comparison of Projective Augmented Reality Concepts to Support Medical Needle Insertion | Title-abstract exclusion |
| 432 | Heinrich, Florian  Schwenderling, Luisa  Joeres, Fabian  Lawonn, Kai  Hansen, Christian | 2020 | Comparison of Augmented Reality Display Techniques to Support Medical Needle Insertion | Title-abstract exclusion |
| 433 | Heinzow, H. S.  Lenz, P.  Lallier, S.  Lenze, F.  Domagk, D.  Domschke, W.  Meister, T. | 2011 | Ampulla of Vater tumors: Impact of intraductal ultrasound and transpapillary endoscopic biopsies on diagnostic accuracy and therapy | Title-abstract exclusion |
| 434 | Helferty, J. P.  Sherbondy, A. J.  Kiraly, A. P.  Higgins, W. E. | 2005 | System for live virtual-endoscopic guidance of bronchoscopy | Title-abstract exclusion |
| 435 | Helin, H. O.  Lundin, M. E.  Laakso, M.  Lundin, J.  Helin, H. J.  Isola, J. | 2006 | Virtual microscopy in prostate histopathology: Simultaneous viewing of biopsies stained sequentially with hematoxylin and eosin, and α-methylacyl-coenzyme A racemase/p63 immunohistochemistry | Title-abstract exclusion |
| 436 | Hellmuth, C.  Demmelmair, H.  Schmitt, I.  Peissner, W.  Blüher, M.  Koletzko, B. | 2013 | Association between Plasma Nonesterified Fatty Acids Species and Adipose Tissue Fatty Acid Composition | Title-abstract exclusion |
| 437 | Hendricks, J. B. | 2001 | Virtual reality comes to pathology | Title-abstract exclusion |
| 438 | Heng, Pheng-Ann  Xie, Yongming  Wang, Xinghe  Chui, Yim-Pan  Wong, Tien-Tsin | 2006 | Virtual acupuncture human based on chinese visible human dataset | Title-abstract exclusion |
| 439 | Henna, N.  Fakhar, S. A.  Akhter, N.  Afzal, M. M.  Khan, K. A. A.  Rashid, T.  Aziz, F. | 2017 | Relationship of cytological with histopathological examination of palpable thyroid nodule | Title-abstract exclusion |
| 440 | Henning, D.  Hatzl, J.  Barb, A.  Ebner, J.  Uhl, C.  Böckler, D. | 2024 | Mixed reality in vascular surgery operating rooms | Title-abstract exclusion |
| 441 | Henricks, W. H.  Roumina, K.  Skilton, B. E.  Ozan, D. J.  Goss, G. R. | 2002 | The utility and cost effectiveness of voice recognition technology in surgical pathology | Title-abstract exclusion |
| 442 | Henry-Stanley, M. J.  Stanley, M. W. | 1992 | Processing of needle rinse material from fine-needle aspirations rarely detects malignancy not identified in smears | Title-abstract exclusion |
| 443 | Henshall, G.  Pop, S. R.  Edwards, M. R.  Ap Cenydd, L.  John, N. W. | 2015 | Towards a high fidelity simulation of the kidney biopsy procedure | Title-abstract exclusion |
| 444 | Heo, S.  Lee, S. S.  Kim, S. Y.  Lim, Y. S.  Park, H. J.  Yoon, J. S.  Suk, H. I.  Sung, Y. S.  Park, B.  Lee, J. S. | 2022 | Prediction of Decompensation and Death in Advanced Chronic Liver Disease Using Deep Learning Analysis of Gadoxetic Acid-Enhanced MRI | Title-abstract exclusion |
| 445 | Hernandez-Rodriguez, Jose  Font, Carme  Garcia-Martinez, Ana  Espigol-Frigole, Georgina  Sanmarti, Raimon  Canete, Juan D.  Grau, Josep M.  Cid, Maria C. | 2007 | Development of ischemic complications in patients with giant cell arteritis presenting with apparently isolated Polymyalgia Rheumatica -: Study of a series of 100 patients | Title-abstract exclusion |
| 446 | Hetherington, Jorden  Lessoway, Victoria  Gunka, Vit  Abolmaesumi, Purang  Rohling, Robert | 2017 | SLIDE: automatic spine level identification system using a deep convolutional neural network | Title-abstract exclusion |
| 447 | Hiemenz Holton, Leslie Lynne | 2000 | Development of a haptic feedback model for computer simulation of the epidural anesthesia needle insertion procedure | Title-abstract exclusion |
| 448 | Hiemenz, L.  Stredney, D.  Schmalbrock, P. | 1998 | Development of the force-feedback model for an epidural needle insertion simulator | Title-abstract exclusion |
| 449 | Higgins, W. E.  Helferty, J. P.  Lu, K.  Merritt, S. A.  Rai, L.  Yu, K. C. | 2008 | 3D CT-Video Fusion for Image-Guided Bronchoscopy | Title-abstract exclusion |
| 450 | Higgins, W. E.  Helferty, J. P.  Padfield, D. R. | 2003 | Integrated bronchoscopic video tracking and 3D CT registration for virtual bronchoscopy | Title-abstract exclusion |
| 451 | Hill, Katie  Brown, Chris  Gibbs, Austin  Mitchell, Andrew Robert John | 2022 | Virtual reality device to improve the tolerability of lumbar puncture | Case report |
| 452 | Hitching, R.  Hoffman, H. G.  Garcia-Palacios, A.  Adamson, M. M.  Madrigal, E.  Alhalabi, W.  Alhudali, A.  Sampaio, M.  Peterson, B.  Fontenot, M. R.  Mason, K. P. | 2023 | The Emerging Role of Virtual Reality as an Adjunct to Procedural Sedation and Anesthesia: A Narrative Review | Title-abstract exclusion |
| 453 | Högberg, J.  Rizell, M.  Hultborn, R.  Svensson, J.  Henrikson, O.  Mölne, J.  Gjertsson, P.  Bernhardt, P. | 2016 | Simulation Model of Microsphere Distribution for Selective Internal Radiation Therapy Agrees With Observations | Title-abstract exclusion |
| 454 | Holden, A. V. | 2010 | Development and application of human virtual excitable tissues and organs: From premature birth to sudden cardiac death | Title-abstract exclusion |
| 455 | Holden, M. S.  Ungi, T.  Sargent, D.  McGraw, R. C.  Chen, E. C. S.  Ganapathy, S.  Peters, T. M.  Fichtinger, G. | 2014 | Feasibility of real-time workflow segmentation for tracked needle interventions | Title-abstract exclusion |
| 456 | Holmes Iii, D. R.  Robb, R. | 2000 | Trans-urethral ultrasound (TUUS) imaging for visualization and analysis of the prostate and associated tissues | Title-abstract exclusion |
| 457 | Holtel, M. R.  Burgess, L. P. A. | 2002 | Telemedicine in otolaryngology | Title-abstract exclusion |
| 458 | Holton, L. L. H. | 2001 | Force models for needle insertion created from measured needle puncture data | Title-abstract exclusion |
| 459 | Holzem, K. M.  Sanchez, L. A. | 2023 | Technical tips and clinical experience with the Terumo Relay®Branch aortic endovascular graft | Title-abstract exclusion |
| 460 | Hong, Wei  Qiu, Feng  Kaufman, Arie | 2006 | A pipeline for computer aided polyp detection | Title-abstract exclusion |
| 461 | Hopper, K. D.  Lucas, T. A.  Gleeson, K.  Stauffer, J. L.  Bascom, R.  Mauger, D. T.  Mahraj, R. | 2001 | Transbronchial biopsy with virtual CT bronchoscopy and nodal highlighting | Title-abstract exclusion |
| 462 | Horvath, Samantha  Arikatla, Sreekanth  Cleary, Kevin  Sharma, Karun  Rosenberg, Avi  Enquobahrie, Andinet | 2019 | Towards an Advanced Virtual Ultrasound-guided Renal Biopsy Trainer | Title-abstract exclusion |
| 463 | Hsu, L. H.  Ko, J. S.  You, D. L.  Chu, N. M. | 2005 | Transbronchial needle aspiration guided by integrated positron emission tomography and computed tomography: A preliminary report | Title-abstract exclusion |
| 464 | Hu, B.  Li, G.  Brown, J. Q. | 2019 | Enhanced resolution 3D digital cytology and pathology with dual-view inverted selective plane illumination microscopy | Title-abstract exclusion |
| 465 | Hu, Z.  Luo, J.  Wei, H.  Ou, W.  Xiao, S.  et al | 2015 | Correlation of virtual touch tissue quantification and liver biopsy in a rat liver fibrosis model | Title-abstract exclusion |
| 466 | Huang, C. H.  Veillard, A.  Roux, L.  Loménie, N.  Racoceanu, D. | 2011 | Time-efficient sparse analysis of histopathological whole slide images | Title-abstract exclusion |
| 467 | Huang, Junfeng  Lin, Jinsheng  Chen, Chongxiang  Liang, Weiquan  Chen, Yumei  Li, Hongjia  Zhong, Changhao  Li, Shiyue | 2023 | 5G-Based Remote Virtual Bronchoscopic Navigation-Guided Transbronchial Lung Biopsy for Diagnosis of Lung Cancer: Description of 2 Cases | Title-abstract exclusion |
| 468 | Huang, Mingxing  Liu, Jian  Chow, Monica  Zhou, Xuan  Han, Zongping  He, Zhenjian  Xue, Jinfang  Zhu, Zhe  Li, Xinhua  Xia, Jinyu | 2018 | Negative HBcAg in immunohistochemistry assay of liver biopsy is a predictive factor for the treatment of patients with nucleos(t)ide analogue therapy | Title-abstract exclusion |
| 469 | Huang, Xin  Liu, Xiaoguang  Zhu, Bin  Hou, Xiangyu  Hai, Bao  Li, Shuiqing  et al | 2023 | Evaluation of Augmented Reality Surgical Navigation in Percutaneous Endoscopic Lumbar Discectomy: Clinical Study | Title-abstract exclusion |
| 470 | Huang, Xin  Liu, Xiaoguang  Zhu, Bin  Hou, Xiangyu  Hai, Bao  Yu, Dongfang | 2023 | Augmented Reality Surgical Navigation in Minimally Invasive Spine Surgery: A Preclinical Study | Title-abstract exclusion |
| 471 | Hufnagel, G.  Pankuweit, S.  Maisch, B. | 1998 | Treatment of dilated cardiomyopathy with or without inflammation | Title-abstract exclusion |
| 472 | Hunt, L.  Hahn, R.  LaTour, D.  Tamares, S.  Crawley, B.  Baldwin, D. | 2021 | PAIN ASSESSMENT FOR OFFICE BASED UROLOGIC PROCEDURES | Title-abstract exclusion |
| 473 | Hussain, Raabid  Lalande, Alain  Marroquin, Roberto  Guigou, Caroline  Grayeli, Alexis Bozorg | 2020 | Video-based augmented reality combining CT-scan and instrument position data to microscope view in middle ear surgery | Title-abstract exclusion |
| 474 | Hwang, E. J.  Kim, H.  Park, C. M.  Yoon, S. H.  Lim, H. J.  Goo, J. M. | 2018 | Cone beam computed tomography virtual navigationguided transthoracic biopsy of small (≥1 cm) pulmonary nodules: Impact of nodule visibility during real-Time fluoroscopy | Title-abstract exclusion |
| 475 | Ianculescu, V.  Ciolovan, L. M.  Dunant, A.  Vielh, P.  Mazouni, C.  Delaloge, S.  Dromain, C.  Blidaru, A.  Balleyguier, C. | 2014 | Added value of Virtual Touch IQ shear wave elastography in the ultrasound assessment of breast lesions | Title-abstract exclusion |
| 476 | Iftikhar, M.  Masood, K.  Song, T. T. | 2009 | A model proposal for tele-pathology labs (TelePol) | Title-abstract exclusion |
| 477 | Igarashi, T. | 2020 | Editorial Comment to Application of virtual reality in patient explanation of magnetic resonance imaging–ultrasound fusion prostate biopsy | Title-abstract exclusion |
| 478 | Ikegami, T.  Taketomi, A.  Soejima, Y.  Yoshizumi, T.  Fukuhara, T.  Kotoh, K.  Shimoda, S.  Kato, M.  Maehara, Y. | 2009 | The Benefits of Interferon Treatment in Patients Without Sustained Viral Response After Living Donor Liver Transplantation for Hepatitis C | Title-abstract exclusion |
| 479 | Imlay, H.  Gnann, J. W.  Rooney, J.  Peddi, V. R.  Wiseman, A. C.  Josephson, M. A.  Kew, C.  Young, J. Ah  Adey, D. B.  Samaniego-Picota, M.  et al., | 2024 | A randomized, placebo-controlled, dose-escalation phase I/II multicenter trial of low-dose cidofovir for BK polyomavirus nephropathy | Title-abstract exclusion |
| 480 | İncetan, K.  Celik, I. O.  Obeid, A.  Gokceler, G. I.  Ozyoruk, K. B.  Almalioglu, Y.  Chen, R. J.  Mahmood, F.  Gilbert, H.  Durr, N. J.  Turan, M. | 2021 | VR-Caps: A Virtual Environment for Capsule Endoscopy | Title-abstract exclusion |
| 481 | Inoue, H.  Cho, J. Y.  Satodate, H.  Sakashita, M.  Hidaka, E.  Fukami, S.  Kazawa, T.  Yoshida, T.  Shiokawa, A.  Kudo, S. | 2003 | Development of virtual histology and virtual biopsy using laser-scanning confocal microscopy | Title-abstract exclusion |
| 482 | Irct | 2020 | The effect of virtual reality on catheter insertion anxiety | Title-abstract exclusion |
| 483 | Irct | 2024 | Comparing the effect of virtual reality and aromatherapy with lavender on anxiety caused by bone marrow biopsy | Registration record |
| 484 | Ishida, T.  Asano, F.  Yamazaki, K.  Shinagawa, N.  Oizumi, S.  Moriya, H.  Munakata, M.  Nishimura, M. | 2011 | Virtual bronchoscopic navigation combined with endobronchial ultrasound to diagnose small peripheral pulmonary lesions: A randomised trial | Title-abstract exclusion |
| 485 | Isiyel, E.  Yurttas, M.  Perktas, E.  Ozmert, E. N.  Teksam, O. | 2023 | Effect of an Active Distraction Method for Pediatric Venipuncture-Related Pain and Anxiety | Title-abstract exclusion |
| 486 | Isrctn, | 2013 | Autologous Stem cells, Chondrocytes Or the Two? | Title-abstract exclusion |
| 487 | Itano, H.  Hirokawa, Y.  Takauchi, K. | 2010 | Clinical utility of three-dimensional integrated 18F-fluorodeoxyglucose positron-emission tomography/computed tomography virtual mediastinoscopy | Title-abstract exclusion |
| 488 | Ivan, M. E.  Yarlagadda, J.  Saxena, A. P.  Martin, A. J.  Starr, P. A.  Sootsman, W. K.  Larson, P. S. | 2014 | Brain shift during bur hole-based procedures using interventional MRI: Clinical article | Title-abstract exclusion |
| 489 | Jacques, J.  Legros, R.  Sautereau, D. | 2019 | Mechanical model of sphincterotomy: A step forward for trainees | Title-abstract exclusion |
| 490 | Jagtap, Jitendra | 2010 | SURGICAL SKILLS LAB FOR PERCUTANEOUS RENAL ACCESS TRAINING: CONTENT VALIDATION COMPARISON BETWEEN LIVE PORCINE AND VR SIMULATION MODEL | Title-abstract exclusion |
| 491 | James, Ryan | 2019 | A New Perspective on Minimally Invasive Procedures: Exploring the Utility of a Novel Virtual Reality Endovascular Navigation System | Title-abstract exclusion |
| 492 | James, Ryan  Monsky, Wayne L.  Seslar, Stephen | 2019 | INITIAL EXPERIENCE PERFORMING A SIMULATED TRANSSEPTAL PUNCTURE WITH A VIRTUAL REALITY HEAD MOUNTED DISPLAY CATHETER NAVIGATION SYSTEM | Title-abstract exclusion |
| 493 | James, Ryan C.  Monsky, Wayne L.  Jorgensen, Neal W.  Seslar, Stephen P. | 2020 | Virtual-Reality Guided Versus Fluoroscopy-Guided Transseptal Puncture in a Cardiac Phantom | Title-abstract exclusion |
| 494 | Jang, C.  Inder, W. J.  Obeyesekere, V. R.  Alford, F. P. | 2008 | Adiponectin, skeletal muscle adiponectin receptor expression and insulin resistance following dexamethasone | Title-abstract exclusion |
| 495 | Janonytė, Viktorija | 2022 | Duchenne Muscular Dystrophy: The Role of Rehabilitation in Maintaining the Functional State of Patients | Title-abstract exclusion |
| 496 | Janssoone, T.  Chevreau, G.  Vadcard, L.  Mozerb, P.  Troccaz, J. | 2011 | Biopsym: A learning environment for trans-rectal ultrasound guided prostate biopsies | Title-abstract exclusion |
| 497 | Jaquez, S. D.  Haller, C. N.  England, M. E.  Bruinsma, R. L.  Arbet, G.  Croce, E. A.  Ruth, J.  Levy, M. L.  Diaz, L. Z. | 2023 | Virtual reality and noise canceling headphone distraction during pediatric dermatologic procedures | Title-abstract exclusion |
| 498 | Javier, G.  Ortega, J. J.  Saenz, A.  Tusell, J. | 1976 | Neonatal leukemia. Report of seven cases (author's transl) | Title-abstract exclusion |
| 499 | Jean, Walter C.  Tai, Alexander X.  Hogan, Elizabeth  Herur-Raman, Aalap  Felbaum, Daniel R.  Leonardo, Jody  Syed, Hasan R. | 2019 | An anatomical study of the foramen of Monro: implications in management of pineal tumors presenting with hydrocephalus | Title-abstract exclusion |
| 500 | Jeremiasen, M.  Walther, C.  Rissler, P.  Johansson, J.  Larsson, M.  Walther, B. | 2016 | A new endoscopic drill biopsy tool for diagnostics of submucosal lesions in the upper gi tract | Title-abstract exclusion |
| 501 | Jerman, Anze  Umek, Nejc  Cvetko, Erika  Snoj, Ziga | 2023 | Comparison of the feasibility and safety of infrazygomatic and suprazygomatic approaches to pterygopalatine fossa using virtual reality | Title-abstract exclusion |
| 502 | Jiang, Baichuan  Wang, Liam  Xu, Keshuai  Hossbach, Martin  Demir, Alican  Rajan, Purnima  Taylor, Russell H.  Moghekar, Abhay  Foroughi, Pezhman  Kazanzides, Peter  Boctor, Emad M. | 2023 | Wearable Mechatronic Ultrasound-integrated AR Navigation System for Lumbar Puncture Guidance | Title-abstract exclusion |
| 503 | Jiang, Y.  Guan, T. M.  Ci, Y.  Zhu, Y.  Zhao, P.  Zheng, J. F.  Yang, T.  Zhang, G. Y. | 2024 | Application of mixed reality technology in vertebroplasty | Title-abstract exclusion |
| 504 | Jiang, Z.  Gao, Z.  Chen, X.  Sun, W. | 2013 | Remote haptic collaboration for virtual training of lumbar puncture | Title-abstract exclusion |
| 505 | Jin, Faguang  Wang, Xiaofang  Qi, Maomao  Zhang, Wenhua  Zhang, Yongfeng | 2024 | Effectiveness and safety of Buzzy device in needle-related procedures for children under twelve years of age: A systematic review and meta-analysis | Title-abstract exclusion |
| 506 | R. B. R. jmy | 2022 | The effect of Virtual Reality to aid Peripheral Venipuncture in children | Title-abstract exclusion |
| 507 | John, N. W.  Luboz, V.  Bello, F.  Hughe, C.  Vidal, F.  Lim, I. S.  How, T. V.  Zhai, J.  et al | 2008 | Physics-based virtual environment for training core skills in vascular interventional radiological procedures | Title-abstract exclusion |
| 508 | John, N. W.  Phillips, N. | 2000 | Surgical simulators using the WWW | Title-abstract exclusion |
| 509 | Johnson, J. P.  Krupinski, E. A.  Yan, M.  Roehrig, H. | 2010 | Use of a visual discrimination model to detect compression artifacts in virtual pathology images | Title-abstract exclusion |
| 510 | Johnson, J. P.  Krupinski, E. A.  Yan, M.  Roehrig, H.  Graham, A. R.  Weinstein, R. S. | 2011 | Using a visual discrimination model for the detection of compression artifacts in virtual pathology images | Title-abstract exclusion |
| 511 | Johnson, S.  Hunt, C.  Woolnough, H.  Crawshaw, M.  Kilkenny, C.  Gould, D.  England, A.  Sinha, A. | 2010 | Assessing performance on a virtual reality simulated liver biopsy procedure: Validating Imagine-S | Title-abstract exclusion |
| 512 | Johnson, S. J.  Hunt, C. M.  Woolnough, H. M.  Crawshaw, M.  Kilkenny, C.  Gould, D. A.  England, A.  Sinha, A.  Villard, P. F. | 2012 | Virtual reality, ultrasound-guided liver biopsy simulator: development and performance discrimination | Title-abstract exclusion |
| 513 | Jones, N. W.  Hutchinson, E. S.  Brownbill, P.  Crocker, I. P.  Eccles, D.  Bugg, G. J.  Raine-Fenning, N. J. | 2009 | In Vitro Dual Perfusion of Human Placental Lobules as a Flow Phantom to Investigate the Relationship between Fetoplacental Flow and Quantitative 3D Power Doppler Angiography | Title-abstract exclusion |
| 514 | Jose, J.  Bhavani, R. R. | 2018 | Bimanual haptic simulator for training hand palpation and lumbar puncture | Title-abstract exclusion |
| 515 | Joshi, A. R.  Lawande, M. A.  Khanna, P. C. | 2002 | Virtual bronchoscopy using multislice CT scanner: Early clinical experience | Title-abstract exclusion |
| 516 | Juanes, J. A.  Alonso, P.  Hernández, F.  Ruisoto, P.  Santos, J. A.  Muriel, C. | 2013 | Digital viewer for learning regional anaesthesia | Title-abstract exclusion |
| 517 | Jung, E. M.  Friedrich, C.  Hoffstetter, P.  Dendl, L. M.  Klebl, F.  Agha, A.  Wiggermann, P.  Stroszcynski, C.  Schreyer, A. G. | 2012 | Volume navigation with contrast enhanced ultrasound and image fusion for percutaneous interventions: First results | Title-abstract exclusion |
| 518 | Jureidini, S.  Chase, N. A.  Alpert, B. S.  Vanderzalm, T.  Sheneflet, R. E. | 1986 | Soft-tissue swelling in two neonates during prostaglandin E1 therapy | Title-abstract exclusion |
| 519 | Kadioglu, O.  Bahramimehr, F.  Dawood, M.  Mahmoud, N.  Elbadawi, M.  Lu, X.  Bülbül, Y.  Schulz, J. A.  et al | 2023 | A drug repurposing approach for individualized cancer therapy based on transcriptome sequencing and virtual drug screening | Title-abstract exclusion |
| 520 | Kagadis, G. C.  Siablis, D.  Liatsikos, E. N.  Petsas, T.  Nikiforidis, G. C. | 2006 | Virtual endoscopy of the urinary tract | Title-abstract exclusion |
| 521 | Kaiser, M. F.  Hall, A.  Walker, K.  Sherborne, A.  De Tute, R. M.  Newnham, N.  Roberts, S.  et al | 2023 | Daratumumab, Cyclophosphamide, Bortezomib, Lenalidomide, and Dexamethasone as Induction and Extended Consolidation Improves Outcome in Ultra-High-Risk Multiple Myeloma | Title-abstract exclusion |
| 522 | Kalanjeri, S.  Gildea, T. R. | 2016 | Electromagnetic Navigational Bronchoscopy for Peripheral Pulmonary Nodules | Title-abstract exclusion |
| 523 | Kalinski, T.  Zwönitzer, R.  Grabellus, F.  Sheu, S. Y.  Sel, S.  Hofmann, H.  Bernarding, J.  Roessner, A. | 2009 | Lossy compression in diagnostic virtual 3-dimensional microscopy-where is the limit? | Title-abstract exclusion |
| 524 | Kalinski, T.  Zwönitzer, R.  Grabellus, F.  Sheu, S. Y.  Sel, S.  Hofmann, H.  Roessner, A. | 2011 | Lossless compression of JPEG2000 whole slide images is not required for diagnostic virtual microscopy | Title-abstract exclusion |
| 525 | Kane, R. A.  Kruskal, J. B. | 2007 | Intraoperative ultrasonography of the brain and spine | Title-abstract exclusion |
| 526 | Kaneko, S.  Usui, J.  Takahashi, K.  Oda, T.  Yamagata, K. | 2022 | Increased intrarenal post-glomerular blood flow is a key condition for the development of calcineurin inhibitor-induced renal tubular acidosis in kidney transplant recipients | Title-abstract exclusion |
| 527 | Kanetsuna, Y.  Horita, S.  Tanabe, K.  Teraoka, S.  Hattori, M.  Toki, D.  Yamaguchi, Y. | 2008 | Is patchy tubular injury a histopathological marker of acute rejection? | Title-abstract exclusion |
| 528 | Kankam, H. K. N.  Lenti, L.  Razai, M. S.  Hourston, G. J. M.  Khatib, M. | 2021 | The role of simulation in training breast surgeons: a systematic review | Title-abstract exclusion |
| 529 | Kanschik, Dominika  Bruno, Raphael Romano  Wolff, Georg  Kelm, Malte  Jung, Christian | 2023 | Virtual and augmented reality in intensive care medicine: a systematic review | Title-abstract exclusion |
| 530 | Kanzira, A.  Chen, X. | 2022 | Virtual Reality-Based Simulator of Needle Interventions for Liver Biopsy | Title-abstract exclusion |
| 531 | Kapogiannis, F.  Karydakis, P.  Antoniou, C.  Aggelopoulos, A. | 2024 | Transrectal prostate biopsy with a virtual reality device: Less pain, anxiety, and discomfort | Conference Abstract |
| 532 | Karaman, D.  Taşdemir, N. | 2021 | The Effect of Using Virtual Reality During Breast Biopsy on Pain and Anxiety: A Randomized Controlled Trial | Included |
| 533 | Karim, Shah M. R.  Ong, Chin T.  Miah, Mizanur R.  Sleep, Tamsin  Hanifudin, Abdul | 2011 | A Novel Technique of Rescuing Capsulorhexis Radial Tear-out using a Cystotome | Title-abstract exclusion |
| 534 | Karppa, E.  Puura, K.  Jyskä, I.  Turunen, M.  Palmu, S. | 2024 | Case Report: Virtual natural environment solution helped a child cope with a painful procedure | Title-abstract exclusion |
| 535 | Kashiwagi, S.  Asano, Y.  Goto, W.  Morisaki, T.  Shibutani, M.  Tanaka, H.  Hirakawa, K.  Ohira, M. | 2022 | Optical See-through Head-mounted Display (OST-HMD)–assisted Needle Biopsy for Breast Tumor: A Technical Innovation | Title-abstract exclusion |
| 536 | Kassim, M. I. M.  Wu, R.  Shao, F.  Ng, W. S.  Wee, S. B. | 2004 | Tracked arm manipulator for lumpectomy | Title-abstract exclusion |
| 537 | Kath, N.  Handels, H.  Mastmeyer, A. | 2019 | Simulation of radiofrequency ablations for liver puncture in 4D VR simulations | Title-abstract exclusion |
| 538 | Kaufman, A. E.  Lakare, S.  Kreeger, K.  Bitter, I. | 2005 | Virtual colonoscopy | Title-abstract exclusion |
| 539 | Kaufman, L.  Goldhaber, D. M.  Kramer, D. M.  Hawryszko, C.  Georgian-Smith, D.  Haynor, D. | 2001 | Ghost imaging in MRI | Title-abstract exclusion |
| 540 | Kaushik, Abhishek  Dwarakanath, T. A.  Bhutani, Gaurav  Moiyadi, Aliasgar  Chaudhari, Pradip | 2020 | Validation of High Precision Robot-Assisted Methods for Intracranial Applications: Preliminary Study | Title-abstract exclusion |
| 541 | Kehler, U.  Furrer, S.  Parfenov, N.  El-Allawy, A. | 2019 | Facilitated ventricle catheter placement during shunt surgery with mixed reality | Title-abstract exclusion |
| 542 | Keller, J. J.  Johnson, J. P.  Latour, E. | 2020 | Inpatient teledermatology: Diagnostic and therapeutic concordance among a hospitalist, dermatologist, and teledermatologist using store-and-forward teledermatology | Title-abstract exclusion |
| 543 | Kesavan, A.  Gao, Y.  Ng, K. W.  Tay, E. Y. Z.  Guo, Q.  Tay, H. K. L.  Tay, A. H. K.  Ngiam, K. Y.  Chiong, E.  Yeo, Z. | 2024 | Holomedicine: The use of a mixed reality device to aid in transperineal prostate biopsies | Title-abstract exclusion |
| 544 | Keunen, O.  Taxt, T.  Grüner, R.  Lund-Johansen, M.  Tonn, J. C.  Pavlin, T.  Bjerkvig, R.  Niclou, S. P.  Thorsen, F. | 2014 | Multimodal imaging of gliomas in the context of evolving cellular and molecular therapies | Title-abstract exclusion |
| 545 | Khaled, W.  Reichling, S.  Bruhns, O. T.  Boese, H.  Baumann, M.  Monkman, G.  et al | 2004 | Palpation imaging using a haptic system for virtual reality applications in medicine | Title-abstract exclusion |
| 546 | Khamene, A.  Acker, F. W.  Vogt, S.  Azar, F.  Wendt, M.  Sauer, F.  Lewin, J. | 2003 | An augmented reality system for MRI-guided needle biopsies | Title-abstract exclusion |
| 547 | Khamene, A.  Vogt, S.  Azar, F.  Sielhorst, T.  Sauer, F.  Niemann, H. | 2003 | Local 3D reconstruction and augmented reality visualization of free-hand ultrasound for needle biopsy procedures | Title-abstract exclusion |
| 548 | Khan, Nausheen  Hiesgen, Juliane | 2017 | Computerised tomography findings in HIV-associated cryptococcal meningoencephalitis at a tertiary hospital in Pretoria | Title-abstract exclusion |
| 549 | Khan, R.  Plahouras, J.  Johnston, B. C.  Scaffidi, M. A.  Grover, S. C.  Walsh, C. M. | 2018 | Virtual reality simulation training for health professions trainees in gastrointestinal endoscopy | Title-abstract exclusion |
| 550 | Khanna, A.  Alam, M. | 2017 | Shaping and optimization of the non-small cell lung cancer (NSCLC) diagnostic landscape in australia and New Zealand (ANZ) | Title-abstract exclusion |
| 551 | Khanna, P. C.  Joshi, R. A.  Pruthi, S.  Merchant, S. A. | 2003 | Multislice CT virtual colonoscopy in the evaluation of large bowel lesions: A pictorial essay and review of literature | Title-abstract exclusion |
| 552 | Khanna, P. C.  Kukreja, K. U.  Merchant, S. A.  Farooq, M. | 2006 | Virtual cystoscopy: Reality in imaging of bladder tuberculosis | Title-abstract exclusion |
| 553 | Khare, Rahul  Bascom, Rebecca  Higgins, William E. | 2015 | Hands-Free System for Bronchoscopy Planning and Guidance | Title-abstract exclusion |
| 554 | Kikuchi, A.  Nakaguchi, T.  Tanabe, M.  Haneishi, H. | 2012 | Development of a VR-based injection training system using a standardized patient | Title-abstract exclusion |
| 555 | Kim, H. W.  Greenburg, A. G. | 2002 | Nitric oxide scavenging, alone or with nitric oxide synthesis inhibition, modulates vascular hyporeactivity in rats with intraperitoneal sepsis | Title-abstract exclusion |
| 556 | Kim, H. W.  Suh, I. H.  Yi, B. J. | 2004 | Haptic rendering of a puncture task with 4-legged 6 DOF parallel haptic device | Title-abstract exclusion |
| 557 | Kim, J. W.  Carroll, B. T.  Jeong, H.  Kim, K.  Demeo, D. P. | 2022 | Immersive Haptic Interface Simulating Skin Biopsy for Dermatological Skill Training | Title-abstract exclusion |
| 558 | Kim, Phillip  Dall'era, Marc | 2023 | VIRTUAL REALITY IMMERSION FOR REDUCING ANXIETY AND PAIN DURING TRANSPERINEAL PROSTATE BIOPSY: A PROSPECTIVE, RANDOMIZED CLINICAL TRIAL | Meeting Abstract |
| 559 | Kin, T.  Shin, M.  Oyama, H.  Kamada, K.  Kunimatsu, A.  Momose, T.  Saito, N. | 2011 | Impact of multiorgan fusion imaging and interactive 3-dimensional visualization for intraventricular neuroendoscopic surgery | Title-abstract exclusion |
| 560 | Kini, V. R.  White, J. R.  Horwitz, E. M.  Dmuchowski, C. F.  Martinez, A. A.  Vicini, F. A. | 1998 | Long term results with breast-conserving therapy for patients with early stage breast carcinoma in a community hospital setting | Title-abstract exclusion |
| 561 | Kinner, Sonja  Reeder, Scott B.  Yokoo, Takeshi | 2016 | Quantitative Imaging Biomarkers of NAFLD | Title-abstract exclusion |
| 562 | Klaastad, O.  Lilleås, F. G.  Rotnes, J. S.  Breivik, H.  Fosse, E. | 2000 | A magnetic resonance imaging study of modifications to the infraclavicular brachial plexus block | Title-abstract exclusion |
| 563 | Klavdianos, P. B. L.  Parente, M.  Brasil, L. M.  Lamas, J. M. | 2012 | ONTO-MAMA: An unified ontology and 3D graphic model of the female breast anatomy | Title-abstract exclusion |
| 564 | Klavdianos, P. B. L.  Souza, E. K. F.  Brasil, L. M.  Lamas, J. M. | 2011 | Onto-mama: An ontology of the female breast anatomy applicable to a virtual learning environment | Title-abstract exclusion |
| 565 | KleinJan, Gijs H.  Karakullukcu, Baris  Klop, W. Martin C.  Engelen, Thijs  van den Berg, Nynke S.  van Leeuwen, Fijs W. B. | 2017 | Introducing navigation during melanoma-related sentinel lymph node procedures in the head-and-neck region | Title-abstract exclusion |
| 566 | Knight, K.  McClenaghan, C. E.  Singh, B. | 2019 | Virtual reality distraction from painful procedures in the paediatric emergency department | Title-abstract exclusion |
| 567 | Knipfer, Thorsten  Steudle, Ernst | 2008 | Root hydraulic conductivity measured by pressure clamp is substantially affected by internal unstirred layers | Title-abstract exclusion |
| 568 | Knitschke, M.  Bäcker, C.  Schmermund, D.  Böttger, S.  Streckbein, P.  Howaldt, H. P.  Attia, S. | 2021 | Impact of planning method (Conventional versus virtual) on time to therapy initiation and resection margins: A retrospective analysis of 104 immediate jaw reconstructions | Title-abstract exclusion |
| 569 | Knoll, B. M.  Hellmann, M.  Kotton, C. N. | 2013 | Vancomycin-resistant Enterococcus faecium meningitis in adults: Case series and review of the literature | Title-abstract exclusion |
| 570 | Knudsen, Bodo E.  Matsumoto, Edward D.  Chew, Ben H.  Johnson, Brooke  Margulis, Vitaly  Cadeddu, Jeffrey A.  Pearle, Margaret S.  Pautler, Stephen E.  Denstedt, John D. | 2006 | A randomized, controlled, prospective study validating the acquisition of percutaneous renal collecting system access skills using a computer based hybrid virtual reality surgical simulator: Phase I | Title-abstract exclusion |
| 571 | Koç Özkan, T.  Polat, F. | 2020 | The Effect of Virtual Reality and Kaleidoscope on Pain and Anxiety Levels During Venipuncture in Children | Title-abstract exclusion |
| 572 | Kocev, B.  Georgii, J.  Linsen, L.  Hahn, H. K. | 2014 | Information Fusion for Real-Time Motion Estimation in Image-guided Breast Biopsy Navigation | Title-abstract exclusion |
| 573 | Koizumi, N.  Sumiyama, K.  Suzuki, N.  Hattori, A.  Tajiri, H.  Uchiyama, A. | 2003 | Development of a new three-dimensional endoscopic ultrasound system through endoscope shape monitoring | Title-abstract exclusion |
| 574 | Koletsis, Efstratios N.  Kalogeropoulou, Christine  Prodromaki, Eleni  Kagadis, George C.  Katsanos, Konstantinos  Spiropoulos, Konstantinos  Petsas, Theodore  Nikiforidis, George C.  Dougenis, Dimitris | 2007 | Tumoral and non-tumoral trachea stenoses: evaluation with three-dimensional CT and virtual bronchoscopy | Title-abstract exclusion |
| 575 | Kong, L. | 2007 | Full field algebras, operads and tensor categories | Title-abstract exclusion |
| 576 | Konge, Lars  Annema, Jouke  Clementsen, Paul  Minddal, Valentina  Vilmann, Peter  Ringsted, Charlotte | 2013 | Using Virtual-Reality Simulation to Assess Performance in Endobronchial Ultrasound | Title-abstract exclusion |
| 577 | Konge, Lars  Arendrup, Henrik  von Buchwald, Christian  Ringsted, Charlotte | 2011 | Virtual reality simulation of basic pulmonary procedures | Title-abstract exclusion |
| 578 | Konge, L.  Clementsen, P. F.  Annema, J. | 2018 | Efficacy of Endobronchial Ultrasound-Transbronchial Needle Aspiration Virtual-Reality Simulator Training | Title-abstract exclusion |
| 579 | Konge, L.  Clementsen, P. F.  Annema, J. T. | 2018 | Diagnostic Yield of EBUS-TBNA during the Learning Curve | Title-abstract exclusion |
| 580 | Konge, L.  Clementsen, P. F.  Ringsted, C.  Minddal, V.  Larsen, K. R.  Annema, J. T. | 2015 | Simulator training for endobronchial ultrasound: A randomised controlled trial | Title-abstract exclusion |
| 581 | Konge, L.  Savran, M.  Clementsen, P.  Minddal, V.  Annema, J. | 2014 | Exploring competency in endobronchial ultrasonography (EBUS): Correlation between experience, self-assessment, theoretical knowledge, and technical performance | Title-abstract exclusion |
| 582 | Konh, B.  Honarvar, M.  Darvish, K.  Hutapea, P. | 2017 | Simulation and experimental studies in needle–tissue interactions | Title-abstract exclusion |
| 583 | Konishi, Kozo  Nakamoto, Masahiko  Kakeji, Yoshihiro  Tanoue, Kazuo  Kawanaka, Hirofumi  Yamaguchi, Shohei  Ieiri, Satoshi  Sato, Yoshinobu  Maehara, Yoshihiko  Tamura, Shinichi  Hashizume, Makoto | 2007 | A real-time navigation system for laparoscopic surgery based on three-dimensional ultrasound using magneto-optic hybrid tracking configuration | Title-abstract exclusion |
| 584 | Konturek, P. C.  Duda, A.  Brzozowski, T.  Konturek, S. J.  Kwiecien, S.  Drozdowicz, D.  Pajdo, R.  Meixner, H.  Hahn, E. G. | 2000 | Activation of genes for superoxide dismutase, interleukin-1β, tumor necrosis factor-α, and intercellular adhesion molecule-1 during healing of ischemia-reperfusion-induced gastric injury | Title-abstract exclusion |
| 585 | Korkmaz, Emine  Guler, Sevil | 2023 | The Effect of Video Streaming With Virtual Reality on Anxiety and Pain During Bone Marrow Aspiration and Biopsy Procedure | Included |
| 586 | Koti, V. R.  Sirajuddin, M. D.  Ali, M.  Qadri, S. S. A. | 2024 | A COMPARATIVE STUDY OF NSAIDS AND TRANSNASAL SPHENOPALATINE GANGLION BLOCK FOR THE TREATMENT OF POST DURAL PUNCTURE HEADACHE(PDPH) IN LOWER SEGMENT CAESAREAN SECTIONS(LSCS) | Title-abstract exclusion |
| 587 | Koyyalamudi, Veerandra  Sidhu, Gurleen  Cornett, Elyse M.  Viet, Nguyen  Labrie-Brown, Carmen  Fox, Charles J.  Kaye, Alan D. | 2016 | New Labor Pain Treatment Options | Title-abstract exclusion |
| 588 | Krupinski, E. A.  Johnson, J. P.  Jaw, S.  Graham, A. R.  Weinstein, R. S. | 2012 | Compressing virtual pathology slides: Human & model observer evaluation | Title-abstract exclusion |
| 589 | Kshatriya, R. M.  Khara, N. V.  Paliwal, R. P.  Patel, S. N. | 2016 | Role of virtual and flexible bronchoscopy in the management of a case of unnoticed foreign body aspiration presented as nonresolving pneumonia in an adult female | Title-abstract exclusion |
| 590 | Ku, H.  Meng, Z.  Zhang, Y.  Gao, H.  Zhang, W.  Sun, X.  Ku, H. | 2021 | Value of simplified augmented reality technique in location of puncture point before drainage of supratentorial hemorrhage in the elderly | Title-abstract exclusion |
| 591 | Kudo, K.  Moriyasu, F.  Mine, Y.  Miyata, Y.  Sugimoto, K.  Metoki, R.  et al | 2007 | Preoperative RFA simulation for liver cancer using a CT virtual ultrasound system | Title-abstract exclusion |
| 592 | Kudo, M.  Shiina, T.  Moriyasu, F.  Iijima, H.  Tateishi, R.  Yada, N.  Fujimoto, K.  Morikawa, H.  Hirooka, M.  Sumino, Y.  Kumada, T. | 2013 | JSUM ultrasound elastography practice guidelines: Liver | Title-abstract exclusion |
| 593 | Kulcsár, Z.  O'Mahony, E.  Lövquist, E.  Aboulafia, A.  Šabova, D.  Ghori, K.  Iohom, G.  Shorten, G. | 2013 | Preliminary evaluation of a virtual reality-based simulator for learning spinal anesthesia | Title-abstract exclusion |
| 594 | Kuloglu, Figen  Rolain, Jean Marc  Akata, Filiz  Eroglu, Cafer  Celik, Aygul Dogan  Parola, Philippe | 2012 | Mediterranean spotted fever in the Trakya region of Turkey | Title-abstract exclusion |
| 595 | Kumar, A.  Kilkenny, C.  Johnson, S.  Villard, P. F.  Gould, D. | 2013 | Validation of ImaGINe-S; a novel, virtual, interventional radiology simulation | Title-abstract exclusion |
| 596 | Kumar, R.  Thiagarajan, K.  Jagannathan, L.  Liu, L.  Mayawala, K.  de Alwis, D.  Topp, B. | 2021 | Beyond the single average tumor: Understanding IO combinations using a clinical QSP model that incorporates heterogeneity in patient response | Title-abstract exclusion |
| 597 | Kurdi, Madhuri S.  Rajagopal, Vennila  Sangineni, Kalyani Sdl  Thalaiappan, Murugan  Grewal, Anju  Gupta, Sunanda | 2023 | Recent advances in obstetric anaesthesia and critical care | Title-abstract exclusion |
| 598 | Kurdziel, K. A.  Ravizzini, G.  Croft, B. Y.  Tatum, J. L.  Choyke, P. L.  Kobayashi, H. | 2008 | The evolving role of nuclear molecular imaging in cancer | Title-abstract exclusion |
| 599 | Kurian, Matthew  Tomlinson, Benjamin  Martin, James M.  Wish-Baratz, Susan | 2022 | Incorporation of Virtual Reality in Bone Marrow Biopsy Training: A New Frontier of Learning | Title-abstract exclusion |
| 600 | Kuznetsov, K.  Lambert, R.  Rey, J. F. | 2005 | Real-time endoscopic diagnosis of <i>H</i>. <i>pylori</i> infection with the hemoglobin index | Title-abstract exclusion |
| 601 | Kwak, Jin Tae  Sankineni, Sandeep  Xu, Sheng  Turkbey, Baris  Choyke, Peter L.  Pinto, Peter A.  Moreno, Vanessa  Merino, Maria  Wood, Bradford J. | 2017 | Prostate Cancer: A Correlative Study of Vultiparametric VR Imaging and Digital Histopathology | Title-abstract exclusion |
| 602 | Kwon, R. S.  Davila, R. E.  Mullady, D. K.  Al-Haddad, M. A.  Bang, J. Y.  Bingener-Casey, J.  Bosworth, B. P.  et al | 2017 | EGD core curriculum | Title-abstract exclusion |
| 603 | Laas, E.  El Beheiry, M.  Masson, J. B.  Malhaire, C. | 2021 | Partial breast resection for multifocal lower quadrant breast tumour using virtual reality | Title-abstract exclusion |
| 604 | Lachkar, S.  Salaün, M.  Perrot, L.  Gervereau, D.  De Marchi, M.  Le Bouar, G.  Morisse-Pradier, H.  Dominique, S.  Piton, N.  Guisier, F.  Thiberville, L. | 2022 | Virtual bronchoscopy planner and radial-ebus guided biopsy for organizing pneumonia diagnosis | Title-abstract exclusion |
| 605 | Ladetto, M.  Tavarozzi, R.  Evangelista, A.  Zanni, M.  Tucci, A.  et al., | 2022 | RADIOIMMUNOTHERAPY (RIT) VERSUS AUTOLOGOUS HEMATOPOIETIC STEM-CELL TRANSPLANTATION (ASCT) IN RELAPSED/REFRACTORY (R/R) FOLLICULAR LYMPHOMA: a FONDAZIONE ITALIANA LINFOMI (FIL) PHASE III TRIAL | Title-abstract exclusion |
| 606 | Ladjal, H.  Hanus, J. L.  Ferreira, A. | 2008 | Interactive cell injection simulation based on 3D biomechanical tensegrity model | Title-abstract exclusion |
| 607 | Ladjal, H.  Hanus, J. L.  Ferreira, A. | 2013 | Micro-to-nano biomechanical modeling for assisted biological cell injection | Title-abstract exclusion |
| 608 | Ladnier, E.  Sheth, A.  Shupak, R. P. | 2023 | Surgical management of mandibular cherubism in an adolescent: integration of virtual surgical planning | Title-abstract exclusion |
| 609 | Lai, M.  Skyrman, S.  Kor, F.  Homan, R.  Babic, D.  Edström, E.  Persson, O.  Burström, G.  Elmi-Terander, A.  Hendriks, B. H. W.  De With, P. H. N. | 2021 | Development of a CT-compatible anthropomorphic skull phantom for surgical planning, training, and simulation | Title-abstract exclusion |
| 610 | Lambert, V.  Boylan, P.  Boran, L.  Hicks, P.  Kirubakaran, R.  Devane, D.  Matthews, A. | 2020 | Virtual reality distraction for acute pain in children | Title-abstract exclusion |
| 611 | Lampotang, Samsun  Bigos, Andre K.  Avari, Kaizad  Johnson, William T.  Mei, Vincent  Lizdas, David E. | 2021 | SMMARTS An Open Architecture Development Platform for Modular, Mixed, and Augmented Reality Procedural and Interventional Simulators | Title-abstract exclusion |
| 612 | Lampotang, Samsun  Lizdas, David E.  Johnson, William T.  Mei, Vincent  Wakim, Jonathan  Lou, XiangYang  DeStephens, Anthony  Acar, Yahya  Moy, Louis  Ahmad, Ardalanejaz  Brisbane, Wayne  Stringer, Thomas | 2024 | Development and Validation of a Mixed-Reality Simulator for Reducing Biopsy Core Deviation During Simulated Freehand Systematic Prostate Biopsy | Title-abstract exclusion |
| 613 | Landry, Vivianne  Christopoulos, Apostolos  Guertin, Louis  Bissada, Eric  Tabet, Paul  Berania, Ilyes  Royal-Lajeunesse, Emilie  Olivier, Marie-Jo  Ayad, Tareck | 2023 | Patterns of alaryngeal voice adoption and predictive factors of vocal rehabilitation failure following total laryngectomy | Title-abstract exclusion |
| 614 | Larnpotang, S.  Lizdas, D.  Rajon, D.  Luria, I.  Gravenstein, N.  Bisht, Y.  Schwab, W.  Friedman, W.  Bova, F.  Robinson, A. | 2013 | Mixed simulators: Augmented physical simulators with virtual underlays | Title-abstract exclusion |
| 615 | Lathan, C.  Cleary, K.  Greco, R. | 1998 | Development and evaluation of a spine biopsy simulator | Title-abstract exclusion |
| 616 | Lau, S. H.  Wang, G.  Chandrasekeran, M.  Fan, V.  Nazrul, M.  Changa, H.  Fonga, T.  Gelb, J.  Fesera, M.  Yuna, W. | 2009 | Multiscale 3D bioimaging: From cell, tissue to whole organism | Title-abstract exclusion |
| 617 | Laufer, S.  Kempton, S. J.  MaCiolek, K.  Terry, A.  Ray, R. D.  Pugh, C. M.  Afifi, A. M. | 2016 | A multi-layered needle injection simulator | Title-abstract exclusion |
| 618 | Lavie, David  Ozcan, Muhit  Chaudhry, Arvind  Zhou, Xuan  Paydar, Ima  Farooqui, Mohammed Z. H.  Ghia, Paolo | 2023 | Bellwave-010: Phase 3, Open-Label, Randomized Study of Nemtabrutinib Plus Venetoclax Versus Venetoclax Plus Rituximab in Patients with Relapsed/Refractory Chronic Lymphocytic Leukemia/Small Lymphocytic Lymphoma Following at Least One Prior Therapy | Title-abstract exclusion |
| 619 | Le Du, K.  Septans, A. L.  Maloisel, F.  Vanquaethem, H.  Schmitt, A.  Le Goff, M.  Clavert, A.  Zinger, M.  Bourgeois, H.  Dupuis, O.  et al., | 2023 | A New Option for Pain Prevention Using a Therapeutic Virtual Reality Solution for Bone Marrow Biopsy (REVEH Trial): open-Label, Randomized, Multicenter, Phase 3 Study | Included |
| 620 | Le Du, K.  Septans, A. L.  Maloisel, F.  Vanquaethem, H.  Schmitt, A.  Le Goff, M.  et al | 2021 | A new option in pain prevention with bliss, a therapeutic virtual reality solution in bone marrow biopsy context: Results of a French open-label multicenter randomized phase II/III study (REVEH Trial) | Conference Abstract |
| 621 | Lee, H. N.  Bae, W.  Park, J. W.  Jung, J. Y.  Hwang, S.  Kim, D. K.  Kwak, Y. H. | 2021 | Virtual reality environment using a dome screen for procedural pain in young children during intravenous placement: a pilot randomized controlled trial | Title-abstract exclusion |
| 622 | Lee, H. N.  Park, J. W.  Hwang, S.  Jung, J. Y.  Kim, D. K.  Kwak, Y. H.  Lee, E. J. | 2023 | Effect of a Virtual Reality Environment Using a Domed Ceiling Screen on Procedural Pain during Intravenous Placement in Young Children: A Randomized Clinical Trial | Title-abstract exclusion |
| 623 | Lee, J.  Ryu, J. H.  Seo, S. H.  Han, S.  Park, J. W. | 2024 | Virtual reality vs. Tablet video for venipuncture education in children: A randomized clinical trial | Title-abstract exclusion |
| 624 | Lee, Jaeyeon  Zhang, Xiao  Park, Chung Hyuk  Kim, Min Jun | 2021 | Real-Time Teleoperation of Magnetic Force-Driven Microrobots With 3D Haptic Force Feedback for Micro-Navigation and Micro-Transportation | Title-abstract exclusion |
| 625 | Lee, L. S.  Nieto, J.  Watson, R. R.  Hwang, A. L.  Muthusamy, V. R.  Walter, L.  Jajoo, K.  Ryou, M. K.  Saltzman, J. R.  Saunders, M. D.  et al., | 2016 | Randomized non-inferiority trial comparing diagnostic yield of cytopathologist-guided versus seven passes for endoscopic ultrasound-guided fine-needle aspiration of pancreatic masses | Title-abstract exclusion |
| 626 | Lee, R.  Vaidy, K.  Telega, G.  Lerner, D.  Chugh, A. | 2024 | FIRST YEAR EXPERIENCE WITH DISPOSABLE TRANSNASAL ENDOSCOPY AT A TERTIARY, PEDIATRIC ACADEMIC CENTER | Title-abstract exclusion |
| 627 | Lee, S.  Lee, J.  Lee, A.  Park, N.  Lee, S.  Song, S.  Seo, A.  Lee, H.  Kim, J. I.  Eom, K. | 2013 | Augmented reality intravenous injection simulator based 3D medical imaging for veterinary medicine | Title-abstract exclusion |
| 628 | Lee, S. K.  Yang, S. C.  Chung, P. C.  Lo, C. S.  Yang, C. W.  Lee, T.  Chang, C. I. | 2001 | Three-dimensional localization of microcalcifications on X-ray mammograms | Title-abstract exclusion |
| 629 | Leenders, B.  Bruijnen, S.  Elshof, J. W. | 2019 | Ultrasound Guided Central Line Placement: Is a Gelatine Phantom a Good and Affordable Alternative? | Title-abstract exclusion |
| 630 | Leendertse, M.  Willems, R. J. L.  Oei, G. A.  Florquin, S.  Bonten, M. J. M.  Van Der Poll, T. | 2009 | Intestinal Enterococcus faecium colonization improves host defense during polymicrobial peritonitis | Title-abstract exclusion |
| 631 | Leopold, I.  Denson, K.  Cutler, E.  Schaake, R.  Zenk, B.  Shafer, L.  Maresky, H.  Cohen, G. | 2022 | Abstract No. 14 Virtual reality and its effect on reduction of pain during interventional radiology procedures | Title-abstract exclusion |
| 632 | Levy, M. L.  Day, J. D.  Albuquerque, F.  Schumaker, G.  Giannotta, S. L.  McComb, J. G. | 1997 | Heads-up intraoperative endoscopic imaging: a prospective evaluation of techniques and limitations | Title-abstract exclusion |
| 633 | Lezrek, M.  Tazi, H.  Alaoui, A. S.  Bazine, K.  Alami, M. | 2013 | Percutaneous calyx puncture simulation in a glove model | Title-abstract exclusion |
| 634 | Lezrek, M.  Tazi, H.  Ammani, A.  Slimani, A.  Bazine, K.  Asseban, M.  Kasmaoui, E.  Qarro, A.  Alami, M.  Beddouch, A.  Rabii, R. | 2013 | Learning percutaneous calyx access in a glove model | Title-abstract exclusion |
| 635 | Lezrek, M.  Tazi, H.  Slimani, A.  Bazine, K.  Ammani, A.  Kasmaoui, E. H.  Alami, M. | 2013 | A glove model for learning of calyx puncture in percutaneous renal surgery | Title-abstract exclusion |
| 636 | Li, Chengyang  Zhang, Xiao  Zhuang, Xueting  Zhang, Kun  Huang, Qiyuan  Ge, Song  Wu, Yong  Hu, Rong | 2024 | The effectiveness of non-pharmacological interventions on reducing pain in patients undergoing bone marrow aspiration and biopsy: A systematic review and meta-analysis of randomized controlled trials | Title-abstract exclusion |
| 637 | Li, Feiyan  Tai, Yonghang  Li, Qiong  Peng, Jun  Huang, Xiaoqiao  Chen, Zaiqing  Shi, Junsheng | 2019 | Real-Time Needle Force Modeling for VR-Based Renal Biopsy Training with Respiratory Motion Using Direct Clinical Data | Title-abstract exclusion |
| 638 | Li, Haowei  Yan, Wenqing  Zhao, Jiasheng  Ji, Yuqi  Qian, Long  Ding, Hui  Zhao, Zhe  Wang, Guangzhi | 2024 | Navigate biopsy with ultrasound under augmented reality device: Towards higher system performance | Title-abstract exclusion |
| 639 | Li, J. F.  Fang, S. M.  He, H. W.  Wen, J. W. | 2014 | Realization of the car tire puncture in virtual environment | Title-abstract exclusion |
| 640 | Li, Ming  Mehralivand, Sherif  Xu, Sheng  Varble, Nicole  Bakhutashvili, Ivane  Gurram, Sandeep  Pinto, Peter A.  Choyke, Peter L.  Wood, Bradford J.  Turkbey, Baris | 2023 | HoloLens augmented reality system for transperineal free-hand prostate procedures | Title-abstract exclusion |
| 641 | Li, Ming  Seifabadi, Reza  Long, Dilara  De Ruiter, Quirina  Varble, Nicole  Hecht, Rachel  Negussie, Ayele H.  Krishnasamy, Venkatesh  Xu, Sheng  Wood, Bradford J. | 2020 | Smartphone- versus smartglasses-based augmented reality (AR) for percutaneous needle interventions: system accuracy and feasibility study | Title-abstract exclusion |
| 642 | Li, M.  Zuo, X. L.  Li, Y. Q. | 2014 | Virtual gastroscopy for the evaluation of stomach malignancy | Title-abstract exclusion |
| 643 | Li, Q.  Duan, Y.  Baikpour, M.  Pierce, T. T.  McCarthy, C. J.  Thabet, A.  Chan, S. T.  Samir, A. E. | 2020 | Magnetic resonance imaging/transrectal ultrasonography fusion guided seed placement in a phantom: Accuracy between 2-seed versus 1-seed strategies | Title-abstract exclusion |
| 644 | Li, Ruotong  Si, Weixin  Liao, Xiangyun  Wang, Qiong  Klein, Reinhard  Heng, Pheng-Ann | 2019 | Mixed reality based respiratory liver tumor puncture navigation | Title-abstract exclusion |
| 645 | Li, Ruotong  Tong, Yuqi  Yang, Tianpei  Guo, Jianxi  Si, Weixin  Zhang, Yanfang  Klein, Reinhard  Heng, Pheng-Ann | 2021 | Towards quantitative and intuitive percutaneous tumor puncture via augmented virtual reality | Title-abstract exclusion |
| 646 | Li, Y.  Brodlie, K.  Phillips, N. | 2000 | Web-based VR training simulator for percutaneous rhizotomy | Title-abstract exclusion |
| 647 | Liang, Huiqing  Liu, Yaoyu  Jiang, Xiaoqian  Zheng, Xiaoting  Tang, Jinmo  Yang, Jiaen  et al | 2020 | Impact of Hepatic Steatosis on the Antiviral Effects of PEG-IFNα-2a in Patients with Chronic Hepatitis B and the Associated Mechanism | Title-abstract exclusion |
| 648 | Lilamand, M.  Vrillon, A.  Gonzales-Marabal, L.  Sindzingre, L.  Götze, K.  Boddaert, J.  Pautas, E.  François-Fasille, V.  Dumurgier, J.  Paquet, C. | 2023 | Lumbar puncture training with healthcare simulation improves self-confidence and practical skills of French medical residents in geriatrics | Title-abstract exclusion |
| 649 | Lim, L. G.  Yeoh, K. G.  Srivastava, S.  Chan, Y. H.  Teh, M.  Ho, K. Y. | 2013 | Comparison of probe-based confocal endomicroscopy with virtual chromoendoscopy and white-light endoscopy for diagnosis of gastric intestinal metaplasia | Title-abstract exclusion |
| 650 | Lin, G. S.  Oppenheimer, R. G.  Mobbs, L.  Garra, B. S. | 2000 | Differentiation of breast masses by vibrational resonance ultrasonic Doppler contrast spectra: Preliminary experience in 20 patients | Title-abstract exclusion |
| 651 | Lin, Hui-Chen  Hwang, Gwo-Jen  Chou, Kuei-Ru  Tsai, Chia-Kuang | 2023 | Fostering complex professional skills with interactive simulation technology: A virtual reality-based flipped learning approach | Title-abstract exclusion |
| 652 | Lin, Julie  Ost, David E. | 2021 | Robotic bronchoscopy for peripheral pulmonary lesions: a convergence of technologies | Title-abstract exclusion |
| 653 | Lin, Michael A.  Siu, Alexa F.  Bae, Jung Hwa  Cutkosky, Mark R.  Daniel, Bruce L. | 2018 | HoloNeedle: Augmented Reality Guidance System for Needle Placement Investigating the Advantages of Three-Dimensional Needle Shape Reconstruction | Title-abstract exclusion |
| 654 | Lin, Wei  Zhu, Zhaoju  He, Bingwei  Liu, Yuqing  Hong, Wenyao  Liao, Zhengjian | 2022 | A novel virtual reality simulation training system with haptic feedback for improving lateral ventricle puncture skill | Title-abstract exclusion |
| 655 | Little, E. L.  Artemiou, E.  Pereira, M. M.  Hunt, J. A. | 2024 | The Impact of Self-Directed Arthrocentesis Model Practice on Student Stress and Procedural Performance of Live Donkey Arthrocentesis | Title-abstract exclusion |
| 656 | Liu, K.  Ninan, S.  Chang, J.  Iloreta, A. M. C.  Kirke, D. N.  Courey, M. S. | 2020 | Integrating virtual reality as distraction analgesia for office-based laryngology procedures | Title-abstract exclusion |
| 657 | Liu, Ruoheng  Luo, Jianghong  Spasojevic, Predrag | 2007 | Adaptive transmission with variable-rate turbo bit-interleaved coded modulation | Title-abstract exclusion |
| 658 | Lohre, Ryan  Wang, Jeffrey C.  Lewandrowski, Kai-Uwe  Goel, Danny P. | 2020 | Virtual reality in spinal endoscopy: a paradigm shift in education to support spine surgeons | Title-abstract exclusion |
| 659 | Lopez-Ceron, M.  Sanabria, E.  Pellise, M. | 2014 | Colonic polyps: Is it useful to characterize them with advanced endoscopy? | Title-abstract exclusion |
| 660 | Lu, Mingqin  Song, Yuting  Niu, Yushuo  Liu, Ting  Ge, Song  Sun, Yaru  Wang, Xin  Luo, Ying  Li, Kuinan  Yang, Xiuling | 2024 | Effectiveness of Virtual Reality in the Management of Anxiety and Pain Peri-Treatment for Breast Cancer: A Systematic Review and Meta-Analysis | Title-abstract exclusion |
| 661 | Lu, WenhuiSun, BaoliMo, Jianyue  Zeng, Xiduo  Zhang, Guanqun  Wang, Lianxiang  et al | 2014 | Attenuation and Immunogenicity of a Live High Pathogenic PRRSV Vaccine Candidate with a 32-Amino Acid Deletion in the nsp2 Protein | Title-abstract exclusion |
| 662 | Luboz, V.  Hughes, C.  Gould, D.  John, N.  Bello, F. | 2009 | Real-time seldinger technique simulation in complex vascular models | Title-abstract exclusion |
| 663 | Luboz, V.  Zhang, Y.  Johnson, S.  Song, Y.  Kilkenny, C.  Hunt, C.  et al | 2013 | ImaGiNe Seldinger: First simulator for Seldinger technique and angiography training | Title-abstract exclusion |
| 664 | Lui, A. J.  Hahn, M. E.  Hussain, T.  Conlin, C. C.  Zhong, A. Y.  Digma, L. A.  et al | 2022 | ReIGNITE RT Boost: An International Study Testing the Feasibility, Accuracy and Reliability of Using Restriction Spectrum Imaging (RSI) MRI to Guide Radiotherapy Target Volume Delineation for Prostate Cancer Tumor Boost | Title-abstract exclusion |
| 665 | Luo, D.  Zhang, Y.  Zhao, R. | 2018 | Study on Deformation Technology of Virtual Surgery Simulator Based on Liver Puncture | Title-abstract exclusion |
| 666 | Ma, X. J.  Lin, Y. P.  Huang, Y. L.  Sun, W.  Shen, J. K.  Sun, M. X.  Zuo, D. Q.  Fu, Z. Z.  Wang, L.  Fu, Q.  Cai, Z. D. | 2019 | Application and exploration of mixed reality technology in accurate operation of percutaneous vertebroplasty of spine | Title-abstract exclusion |
| 667 | Magee, D.  Zhu, Y.  Ratnalingam, R.  Gardner, P.  Kessel, D. | 2007 | An augmented reality simulator for ultrasound guided needle placement training | Title-abstract exclusion |
| 668 | Magierowski, M.  Jasnos, K.  Kwiecien, S.  Drozdowicz, D.  Surmiak, M.  Strzalka, M.  Ptak-Belowska, A.  Wallace, J. L.  Brzozowski, T. | 2015 | Endogenous prostaglandins and afferent sensory nerves in gastroprotective effect of hydrogen sulfide against stress-induced gastric lesions | Title-abstract exclusion |
| 669 | Magnano, M.  Bongioannini, G.  Cirillo, S.  Regge, D.  Martinich, L.  Canale, G.  Lerda, W.  Galvagno, M. B.  Taranto, F. | 2005 | Virtual endoscopy of laryngeal carcinoma: Is it useful? | Title-abstract exclusion |
| 670 | Mahmoud, M. A.  Daboos, M.  Gouda, S.  Othman, A.  Abdelmaboud, M.  Hussein, M. E.  Akl, M. | 2022 | Telemedicine (virtual clinic) effectively delivers the required healthcare service for pediatric ambulatory surgical patients during the current era of COVID-19 pandemic: A mixed descriptive study | Title-abstract exclusion |
| 671 | Maiñez, R.  Kelly, R. H.  Kobayashi, M.  Takaya, S.  Bronsther, O.  Kramer, D.  Duquesnoy, R. J.  Iwaki, Y.  Fung, J. J.  Starzl, T. E.  Demetris, A. J. | 1995 | Immunoglobulin G lymphocytotoxic antibodies in clinical liver transplantation: Studies toward further defining their significance | Title-abstract exclusion |
| 672 | Maisons, V.  Lanot, A.  Luque, Y.  Sautenet, B.  Esteve, E.  Guillouet, E.  François, H.  Bobot, M. | 2024 | Simulation-based learning in nephrology | Title-abstract exclusion |
| 673 | Majak, M.  Zuk, M.  Swiatek-Najwer, E.  Popek, M.  Pietruski, P. | 2018 | Biopsy procedure applied in MentorEye molecular surgical navigation system | Title-abstract exclusion |
| 674 | Majak, Marcin  Zuk, Magdalena  Swiatek-Najwer, Ewelina  Popek, Michal  Pietruski, Piotr | 2021 | Augmented reality visualization for aiding biopsy procedure according to computed tomography based virtual plan | Title-abstract exclusion |
| 675 | Majewicz, Ann | 2014 | Robotic Needle Steering: Design and Evaluation for Clinical Application | Title-abstract exclusion |
| 676 | Majumder, S.  Shivaji, U. N.  Kasturi, R.  Sigamani, A.  Ghosh, S.  Iacucci, M. | 2022 | Inflammatory bowel disease-related colorectal cancer: Past, present and future perspectives | Title-abstract exclusion |
| 677 | Makary, Joshua  van Diepen, Danielle C.  Arianayagam, Ranjan  McClintock, George  Fallot, Jeremy  Leslie, Scott  Thanigasalam, Ruban | 2022 | The evolution of image guidance in robotic-assisted laparoscopic prostatectomy (RALP): a glimpse into the future | Title-abstract exclusion |
| 678 | Malavazos, Alexis Elias  Di Vincenzo, Angelica  Iacobellis, Gianluca  Basilico, Sara  Dubini, Carola  Morricone, Lelio  et al | 2022 | The density of crown-like structures in epicardial adipose tissue could play a role in cardiovascular diseases | Title-abstract exclusion |
| 679 | Maliha, Samantha G.  Diaz-Siso, J. Rodrigo  Plana, Natalie M.  Torroni, Andrea  Flores, Roberto L. | 2018 | Haptic, Physical, and Web-Based Simulators: Are They Underused in Maxillofacial Surgery Training? | Title-abstract exclusion |
| 680 | Malloy, K. M.  Milling, L. S. | 2010 | The effectiveness of virtual reality distraction for pain reduction: A systematic review | Title-abstract exclusion |
| 681 | Maluf, D. G.  Dumur, C. I.  Suh, J. L.  Lee, J. K.  Cathro, E. P.  King, A. L.  Gallon, L.  Brayman, K. L.  Mas, V. R. | 2014 | Evaluation of molecular profiles in calcineurin inhibitor toxicity post-kidney transplant: input to chronic allograft dysfunction | Title-abstract exclusion |
| 682 | Malukhin, K.  Ehmann, K. | 2018 | Mathematical Modeling and Virtual Reality Simulation of Surgical Tool Interactions with Soft Tissue: A Review and Prospective | Title-abstract exclusion |
| 683 | Mangalote, Iffa Afsa Changaai  Aboumarzouk, Omar  Al-Ansari, Abdulla A.  Dakua, Sarada Prasad | 2024 | A comprehensive study to learn the impact of augmented reality and haptic interaction in ultrasound-guided percutaneous liver biopsy training and education | Title-abstract exclusion |
| 684 | Manning, P. J.  Dixit, P.  Satthenapalli, V. R.  Katare, R.  Sutherland, W. H. F. | 2019 | WITHDRAWN: GLP-1 Has an Anti-Inflammatory Effect on Adipose Tissue Expression of Cytokines, Chemokines, and Receptors in Obese Women | Title-abstract exclusion |
| 685 | Mansoux, B.  Nigay, L.  Troccaz, J. | 2005 | The mini-screen: An innovative device for Computer Assisted Surgery systems | Title-abstract exclusion |
| 686 | Mantegazza, C.  Silvera, V.  Maestri, L.  Meroni, M.  Destro, F.  La Pergola, E.  Cococcioni, L.  Pelizzo, G.  Zuccotti, G. V. | 2021 | PIPEND: pain in paediatric endoscopy, a pilot study | Title-abstract exclusion |
| 687 | Marchal, M.  Promayon, E.  Troccaz, J. | 2006 | Simulating prostate surgical procedures with a discrete soft tissue model | Title-abstract exclusion |
| 688 | Marcos, Ricardo  Fonte-Oliveira, Luisa  Santos, Marta  Caniatti, Mario | 2023 | An immersive simulation strategy to teach cytology sample collection methods and basic diagnosis skills: A two academic center study | Title-abstract exclusion |
| 689 | Marie-Danielle, V. D.  Christine, J.  Aurélie, C.  Christophe, M.  Olga, F.  Alain, D.  Solène, M.  Gilles, P. | 2014 | Improving the human skin microanatomy understanding and skin aging observation with the SkinExplorer™ platform | Title-abstract exclusion |
| 690 | Marien, A.  De Luis Abreu, A. C.  Desai, M.  Azhar, R. A.  Chopra, S.  Shoji, S.  Matsugasumi, T.  Nakamoto, M.  Gill, I. S.  Ukimura, O. | 2015 | Three-dimensional navigation system integrating position-tracking technology with a movable tablet display for percutaneous targeting | Title-abstract exclusion |
| 691 | Márquez, P. L.  Liuzzi, J. F.  Silva, C.  Espinoza, M. J.  Vuolo, Y.  Alemán, L. | 2016 | Metastasis in a brachial cyst from thyroid papillary carcinoma. A case report | Title-abstract exclusion |
| 692 | Marshall, Lindsey  Atkinson, Andrew  Aminu, Abimbola Juliet  Morehead, Michael  Spirou, George  Iles, Tinen  Iaizzo, Paul A.  Dobrzynski, Halina | 2021 | New observations in a journey through the human heart using immersive virtual reality | Title-abstract exclusion |
| 693 | Martellucci, Jacopo  Naldini, Gabriele  Colosimo, Caterina  Cionini, Luca  Rossi, Mauro | 2009 | Accuracy of endoanal ultrasound in the follow-up assessment for squamous cell carcinoma of the anal canal treated with radiochemotherapy | Title-abstract exclusion |
| 694 | Marti, G.  Rouiller, P.  Grange, S.  Baur, C. | 2003 | Biopsy navigator: A smart haptic interface for interventional radiological gestures | Title-abstract exclusion |
| 695 | Martsopoulos, Athanasios  Hill, Thomas L. L.  Persad, Rajendra  Bolomytis, Stefanos  Tzemanaki, Antonia | 2023 | Modelling and real-time dynamic simulation of flexible needles for prostate biopsy and brachytherapy | Title-abstract exclusion |
| 696 | Mastmeyer, A.  Fortmeier, D.  Handels, H. | 2012 | Direct haptic volume rendering in lumbar puncture simulation | Title-abstract exclusion |
| 697 | Mastmeyer, Andre  Fortmeier, Dirk  Handels, Heinz | 2016 | Efficient patient modeling for visuo-haptic VR simulation using a generic patient atlas | Title-abstract exclusion |
| 698 | Mastmeyer, Andre  Hecht, Tobias  Fortmeier, Dirk  Handels, Heinz | 2014 | Ray-casting based evaluation framework for haptic force feedback during percutaneous transhepatic catheter drainage punctures | Title-abstract exclusion |
| 699 | Mastmeyer, A.  Wilms, M.  Fortmeier, D.  Schroder, J.  Handels, H. | 2016 | Real-time ultrasound simulation for training of us-guided needle insertion in breathing virtual patients | Title-abstract exclusion |
| 700 | Mastmeyer, A.  Wilms, M.  Handels, H. | 2017 | Inter-patient transfer of breathing models for virtual reality training of puncture procedures | Title-abstract exclusion |
| 701 | Mastmeyer, A.  Wilms, M.  Handels, H. | 2017 | Interpatient respiratory motion model transfer for virtual reality simulations of liver punctures | Title-abstract exclusion |
| 702 | Mastmeyer, A.  Wilms, M.  Handels, H. | 2018 | Population-based respiratory 4D motion atlas construction and its application for VR simulations of liver punctures | Title-abstract exclusion |
| 703 | Mastracci, L.  Bruno, S.  Spaggiari, P.  Ceppa, P.  Fiocca, R. | 2008 | The impact of biopsy number and site on the accuracy of intestinal metaplasia detection in the stomach. A morphometric study based on virtual biopsies | Title-abstract exclusion |
| 704 | Matsui, K.  Kitagawa, M. | 1993 | Biopsy study of polyps in the duodenal bulb | Title-abstract exclusion |
| 705 | Matsuki, M.  Kanazawa, S.  Kanamoto, T.  Inada, Y.  Kani, H.  Tanikake, M.  et al | 2006 | Virtual CT gastrectomy by three-dimensional imaging using multidetector-row CT for laparoscopic gastrectomy | Title-abstract exclusion |
| 706 | Matsumoto, Edward D. | 2011 | Development and validation of a virtual reality transrectal ultrasound guided prostatic biopsy simulator | Title-abstract exclusion |
| 707 | Mattei, T. A.  Rodriguez, A. H.  Sambhara, D.  Mendel, E. | 2014 | Current state-of-the-art and future perspectives of robotic technology in neurosurgery | Title-abstract exclusion |
| 708 | Matthews, D. E.  Schwarz, H. P.  Yang, R. D.  Motil, K. J.  Young, V. R.  Bier, D. M. | 1982 | Relationship of plasma leucine and alpha-ketoisocaproate during a L-[1-13C]leucine infusion in man: a method for measuring human intracellular leucine tracer enrichment | Title-abstract exclusion |
| 709 | Mauri, G | 2015 | Expanding role of virtual navigation and fusion imaging in percutaneous biopsies and ablation | Title-abstract exclusion |
| 710 | Mauro, M. A. | 2009 | Interventional radiology: An ever-changing landscape | Title-abstract exclusion |
| 711 | Mazilu, Dumitru  Patriciu, Alexandru  Gruionu, Lucian  McAllister, Marc  Ong, Albert  Ellison, Lars  Frimberger, Dominic  Fugita, Oscar  Kavoussi, Louis  Stoianovici, Dan | 20 | Synthetic torso for training in and evaluation of urologic laparoscopic skills | Title-abstract exclusion |
| 712 | Mazzon, Ivan  Etrusco, Andrea  Lagana, Antonio Simone  Chiantera, Vito  Antonio, Silvia Di Angelo  Tosto, Valentina  Gerli, Sandro  Favilli, Alessandro | 2023 | Training in Diagnostic Hysteroscopy: The "Arbor Vitae" Method | Title-abstract exclusion |
| 713 | McAdams, H. P.  Goodman, P. C.  Kussin, P. | 1998 | Original report. Virtual bronchoscopy for directing transbronchial needle aspiration of hilar and mediastinal lymph nodes: A pilot study | Title-abstract exclusion |
| 714 | McCloskey, Kyle  Turlip, Ryan  Ahmad, Hasan S.  Ghenbot, Yohannes G.  Chauhan, Daksh  Yoon, Jang W. | 2023 | Virtual and Augmented Reality in Spine Surgery: A Systematic Review | Title-abstract exclusion |
| 715 | McConnell, R. A.  Kim, S.  Ahmad, N. A.  Falk, G. W.  Forde, K. A.  Ginsberg, G. G.  Jaffe, D. L.  Makar, G. A.  Long, W. B.  Panganamamula, K. V.  Kochman, M. L. | 2012 | Poor discriminatory function for endoscopic skills on a computer-based simulator | Title-abstract exclusion |
| 716 | McDonald, J. S. | 2000 | Computer driven needle probe enables therapy for painful neuropathies | Title-abstract exclusion |
| 717 | McGill, S.  Gainey, C.  Commins, S.  Friedlander, J. | 2024 | SEDATION-FREE TRANSNASAL UPPER ENDOSCOPY WITH BIOPSIES USING VIRTUAL REALITY DISTRACTION CAN EVALUATE THE UPPER GASTROINTESTINAL TRACT IN ADULT RESEARCH SUBJECTS | Title-abstract exclusion |
| 718 | McGill, S.  Soetikno, R.  Kaltenbach, T. | 2009 | Image-enhanced endoscopy in practice | Title-abstract exclusion |
| 719 | McLennan, Geoffrey  Ferguson, J. Scott  Thomas, Karl  Delsing, Angela S.  Cook-Granroth, Janice  Hoffman, Eric A. | 2007 | The use of MDCT-based computer-aided pathway finding for mediastinal and perihilar lymph node biopsy: A randomized controlled prospective trial | Title-abstract exclusion |
| 720 | McLeod, Ronald Murdock | 1999 | Object and work space modeling in 3-dimensional virtual reality with application to haptic interfaces | Title-abstract exclusion |
| 721 | McNeil, C.  Wong, P. F.  Sridhar, N.  Wang, Y.  Santori, C.  et al | 2024 | An End-to-End Platform for Digital Pathology Using Hyperspectral Autofluorescence Microscopy and Deep Learning-Based Virtual Histology | Title-abstract exclusion |
| 722 | Meliagros, Pete  Diener-Brazelle, Jayson  Garber, Adam | 2024 | Using Mixed Reality to Teach Medical Students Lumbar Punctures | Title-abstract exclusion |
| 723 | Melo, J. S. S.  Brasil, L. M.  Cerqueira, J. P. D. S.  Ramos, M. D. S.  Leitee, A. R. M.  Lima, J. G. M.  Lamas, J. M.  Nunes, F. D. L. S. | 2012 | JCHAI3D: Multiplatform framework for healthcare applications - Multiplatform framework for graphic and haptic processing develop in Java | Title-abstract exclusion |
| 724 | Meshi, A.  Chiaravalloti, T. | 2022 | InVisible - Movement on the Edge of Two Realities | Title-abstract exclusion |
| 725 | Mettler, L.  Vanhecke, K.  Kühnapfel, U.  Cakmak, H. | 2004 | Experience with a new virtual reality laparoscopic trainer for reproductive surgery | Title-abstract exclusion |
| 726 | Mewes, Andre  Heinrich, Florian  Hensen, Bennet  Wacker, Frank  Lawonn, Kai  Hansen, Christian | 2018 | Concepts for augmented reality visualisation to support needle guidance inside the MRI | Title-abstract exclusion |
| 727 | Meyer, B. C.  Peter, O.  Nagel, M.  Hoheisel, M.  Frericks, B. B.  Wolf, K. J.  Wacker, F. K. | 2008 | Electromagnetic field-based navigation for percutaneous punctures on C-arm CT: Experimental evaluation and clinical application | Title-abstract exclusion |
| 728 | Michel, M. S.  Knoll, T.  Köhrmann, K. U.  Alken, P. | 2002 | The URO Mentor:: development and evaluation of a new computer-based interactive training system for virtual life-like simulation of diagnostic and therapeutic endourological procedures | Title-abstract exclusion |
| 729 | Mirbagheri, A.  Owlia, M.  Khabbazan, M.  Moradi, M.  Mohandesi, F. | 2020 | Introducing a robotic lumbar puncture simulator with force feedback: LP sim | Title-abstract exclusion |
| 730 | Mirhosseini, Seyedkoosha  Gutenko, Ievgeniia  Ojal, Sushant  Marino, Joseph  Kaufman, Arie | 2019 | Immersive Virtual Colonoscopy | Title-abstract exclusion |
| 731 | Mirza, E.  Hanif, M.  Khan, M. A.  Jaleel, S. | 2021 | Re: Virtual reality for acute pain in outpatient hysteroscopy: a randomised controlled trial | Title-abstract exclusion |
| 732 | Mirza, S.  Athreya, S. | 2018 | Review of Simulation Training in Interventional Radiology | Title-abstract exclusion |
| 733 | Mishra, Shashikant  Kurien, Abraham  Patel, Rajesh  Patil, Pradip  Ganpule, Arvind  Muthu, Veeramani  Sabnis, Ravindra B.  Desai, Mahesh | 2010 | Validation of Virtual Reality Simulation for Percutaneous Renal Access Training | Title-abstract exclusion |
| 734 | Mitri, F. G.  Urban, M. W.  Fatemi, M.  Greenleaf, J. F. | 2011 | Shear wave dispersion ultrasonic vibrometry for measuring prostate shear stiffness and viscosity: An in vitro pilot study | Title-abstract exclusion |
| 735 | Mittal, Ajay  Wakim, Jonathan  Huq, Suhaiba  Wynn, Tung | 2024 | Effectiveness of Virtual Reality in Reducing Perceived Pain and Anxiety Among Patients Within a Hospital System: Protocol for a Mixed Methods Study | Title-abstract exclusion |
| 736 | Miyagawa, T.  Ishikawa, S.  Kimura, T.  Suetomi, T.  Tsutsumi, M.  Irie, T.  Kondoh, M.  Mitake, T. | 2010 | Real-time virtual sonography for navigation during targeted prostate biopsy using magnetic resonance imaging data | Title-abstract exclusion |
| 737 | Miyake, Y. | 2007 | Development of laparoscopic surgery training system using VR technology | Title-abstract exclusion |
| 738 | Miyata, Akinori  Arita, Junichi  Shirata, Chikara  Abe, Satoru  Akamatsu, Nobuhisa  Kaneko, Junichi  Kokudo, Norihiro  Hasegawa, Kiyoshi | 2020 | Quantitative Assessment of the Accuracy of Real-Time Virtual Sonography for Liver Surgery | Title-abstract exclusion |
| 739 | Miyoshi, Shion  Isobe, Kazutoshi  Shimizu, Hiroshige  Sunakawa, Motoko  Suzuki, Aika  Sugino, Keish  et al | 2019 | The Utility of Virtual Bronchoscopy Using a Computed Tomography Workstation for Conducting Conventional Bronchoscopy: A Retrospective Analysis of Clinical Practice | Title-abstract exclusion |
| 740 | Mohamadnejad, M.  Mullady, D.  Early, D. S.  Collins, B.  Marshall, C.  et al., | 2017 | Increasing Number of Passes Beyond 4 Does Not Increase Sensitivity of Detection of Pancreatic Malignancy by Endoscopic Ultrasound-Guided Fine-Needle Aspiration | Title-abstract exclusion |
| 741 | Mohamadnejad, M.  Mullady, D.  Early, D. S.  Rastogi, A.  Collins, B.  et al., | 2016 | Per-pass analysis on the diagnostic yield of EUS-guided FNA in solid pancreatic mass lesions: analysis from a multicenter randomized controlled trial | Title-abstract exclusion |
| 742 | Mohan, Anant  Madan, Karan  Hadda, Vijay  Tiwari, Pawan  Mittal, Saurabh  Guleria, Randeep  Khilnani, G. C.  et al., | 2019 | Guidelines for diagnostic flexible bronchoscopy in adults: Joint Indian Chest Society/National College of chest physicians (I)/Indian association for bronchology recommendations | Title-abstract exclusion |
| 743 | Mohan, P.  Ho, H.  Yuen, J.  Ng, W. S.  Cheng, W. S. | 2007 | A 3D computer simulation to study the efficacy of transperineal versus transrectal biopsy of the prostate | Title-abstract exclusion |
| 744 | Monteiro, J.  Tanday, A.  Ashley, P. F.  Parekh, S.  Alamri, H. | 2020 | Interventions for increasing acceptance of local anaesthetic in children and adolescents having dental treatment | Title-abstract exclusion |
| 745 | Moorthy, K.  Jiwanji, M.  Shah, J.  Bello, F.  Munz, Y.  Darzi, A. | 2003 | Validation of a web-based training tool for lumbar puncture | Title-abstract exclusion |
| 746 | Moorthy, K.  Mansoori, M.  Bello, F.  Hance, J.  Undre, S.  Munz, Y.  Darzi, A. | 2004 | Evaluation of the benefit of VR simulation in a multi-media web-based educational tool | Title-abstract exclusion |
| 747 | Morales, J. M.  Tedesco-Silva, H.  Peddi, V. R.  Russ, G. R.  Marder, B. A.  Hahn, C. M.  Li, H.  Flynn, A.  Schulman, S. L. | 2013 | Planned transition from tacrolimus to sirolimus versus continued tacrolimus in renal allograft patients | Title-abstract exclusion |
| 748 | Moranne, O.  Cariou, S.  Messikh, Z.  Pambrun, E.  Garo, F.  Schultz, C.  Prouvot, J.  Renaud, S.  Reboul, P. | 2021 | Randomized control trial of intermittent hemodialysis with regional citrate vs priming heparin with predilution in patients at risk of bleeding | Title-abstract exclusion |
| 749 | Morente, M. M.  Mager, R.  Alonso, S.  Pezzella, F.  Spatz, A.  Knox, K.  et al | 2006 | TuBaFrost 2: Standardising tissue collection and quality control procedures for a European virtual frozen tissue bank network | Title-abstract exclusion |
| 750 | Moreta-Martinez, Rafael  Rubio-Perez, Ines  Garcia-Sevilla, Monica  Garcia-Elcano, Laura  Pascau, Javier | 2022 | Evaluation of optical tracking and augmented reality for needle navigation in sacral nerve stimulation | Title-abstract exclusion |
| 751 | Morimoto, Tania | 2017 | Patient-Specific Design of Concentric Tube Robots | Title-abstract exclusion |
| 752 | Morimoto, T.  Greer, J.  Hawkes, E.  Okamura, A.  Hsieh, M. | 2017 | Design, fabrication, and testing of patientspecific concentric tube robots for nonlinear renal access and mass ablation | Title-abstract exclusion |
| 753 | Moscardi, Paulo R. Marcelo  Braun, Ruben Blachman  Ruiz, Nicholas  Masterson, Thomas A. | 2024 | THE IMPACT OF VIRTUAL REALITY(VR) ON PATIENT EXPERIENCE UNDERGOING PROSTATE BIOPSY | Meeting Abstract |
| 754 | Mosgaard, B. J.  Skovlund, V. R.  Hendel, H. W. | 2013 | Promising results using sentinel node biopsy as a substitute for radical lymphadenectomy in endometrial cancer staging | Title-abstract exclusion |
| 755 | Mouzaki, M.  Trout, A. T. | 2019 | Virtual Reality: New Insights Regarding the Prevalence of Nonalcoholic Fatty Liver Disease in Children and Adolescents with Obesity Using Magnetic Resonance Imaging | Title-abstract exclusion |
| 756 | Mu, S.  Hu, J.  Liu, F.  Zhao, C.  Sun, Y. | 2023 | New innovations and educational process in undergraduate neurology education in blended learning | Title-abstract exclusion |
| 757 | Mu, Yanyu | 2020 | Development and Validation of Augmented Reality Training Simulator for Ultrasound Guided Percutaneous Renal Access | Title-abstract exclusion |
| 758 | Mueller, Michael  Rassweiler, Marie-Claire  Klein, Jan  Seitel, Alexander  Gondan, Matthias  Baumhauer, Matthias  Teber, Dogu  Rassweiler, Jens J.  Meinzer, Hans-Peter  Maier-Hein, Lena | 2013 | Mobile augmented reality for computer-assisted percutaneous nephrolithotomy | Title-abstract exclusion |
| 759 | Mühlbauer, M.  Pfisterer, W.  Haberler, C.  Knosp, E. | 2002 | Penetration failure and misdiagnosis of stereotactic biopsy caused by the uncommonly firm tissue of a gliomyosarcoma | Title-abstract exclusion |
| 760 | Mulero Aniorte, F. | 2022 | ImmunoPET in oncology | Title-abstract exclusion |
| 761 | Müller, M.  Rassweiler, M. C.  Klein, J.  Seitel, A.  Gondan, M.  Baumhauer, M.  Teber, D.  Rassweiler, J. J.  Meinzer, H. P.  Maier-Hein, L. | 2013 | Mobile augmented reality for computer-assisted percutaneous nephrolithotomy | Title-abstract exclusion |
| 762 | Muñoz Pandiella, I.  Monclús, E.  Brunet, P.  Conesa, G. | 2012 | Ventricular puncture trainer | Title-abstract exclusion |
| 763 | Muraglia, L.  Mattana, F.  Zuccotti, G.  Collamati, F.  Luzzago, S.  Travaini, L. L.  Musi, G.  Ceci, F. | 2023 | Prostate-specific membrane antigen (PSMA) radioguided surgery in prostate cancer: An overview of current application and future perspectives | Title-abstract exclusion |
| 764 | Murali, A.  Chandran, S.  Thomas, A. | 2024 | Clinicopathological Evaluation of Benign Vulvar Tumors: A Descriptive Study in a South Asian Population | Title-abstract exclusion |
| 765 | Murphy, Declan Ciaran  Felembam, Majed  Hunt, Nicola  Baker, Stuart N.  Lako, Majlinda  Steel, David | 2017 | Does intravitreal Ocriplasmin degrade intraretinal extracellular matrix molecules? | Title-abstract exclusion |
| 766 | Myrga, John M.  Natesan, Divya  Staniorski, Christopher J.  Stencel, Michael  Jacobs, Bruce L.  Davies, Benjamin J. | 2024 | VIRTUAL REALITY WITH BIOFEEDBACK ENHANCES TOLERABILITY OF OFFICE BASED TRANSPERINEAL PROSTATE BIOPSY | Title-abstract exclusion |
| 767 | Na, S. Y.  Moon, W. | 2022 | Recent advances in surveillance colonoscopy for dysplasia in inflammatory bowel disease | Title-abstract exclusion |
| 768 | Nadrous, H. F.  Allen, M. S.  Bartholmai, B. J.  Aughenbaugh, G. L.  Lewis, J. T.  Jett, J. R. | 2004 | Glomus Tumor of the Trachea: Value of Multidetector Computed Tomographic Virtual Bronchoscopy | Title-abstract exclusion |
| 769 | Nakai, T.  Izumo, T.  Matsumoto, Y.  Tsuchida, T. | 2017 | Virtual fluoroscopy during transbronchial biopsy for locating ground-glass nodules not visible on X-ray fluoroscopy | Title-abstract exclusion |
| 770 | Nakajima, Y.  Oyama, H.  Sawada, A.  Muroi, K. | 2000 | Enhanced video image guidance for biopsy using the safety map | Title-abstract exclusion |
| 771 | Nakamoto, Ryusuke  Zhuo, Jialin  Guja, Kip E.  Duan, Heying  Perkins, Stephanie L.  Leuze, Christoph  Daniel, Bruce L.  Franc, Benjamin Lewis | 2022 | Phantom study of SPECT/CT augmented reality for intraoperative localization of sentinel lymph nodes in head and neck melanoma | Title-abstract exclusion |
| 772 | Nanda, A.  Hu, J.  Hodgkinson, S.  Ali, S.  Rainsbury, R.  Roy, P. G. | 2021 | Oncoplastic breast‐conserving surgery for women with primary breast cancer | Title-abstract exclusion |
| 773 | Nankivell, Brian J.  Shingde, Meena  P'Ng, Chow H. | 2022 | The Pathological and Clinical Diversity of Acute Vascular Rejection in Kidney Transplantation | Title-abstract exclusion |
| 774 | Nankivell, Brian J.  Shingde, Meena  P'Ng, Chow H.  Sharma, Ankit | 2022 | The Clinical and Pathologic Phenotype of Antibody-Mediated Vascular Rejection Diagnosed Using Arterial C4d Immunoperoxidase | Title-abstract exclusion |
| 775 | Natesan, D.  Myrga, J. M.  Stencel, M. G.  Staniorski, C. J.  Alcorn, M.  Erpenbeck, S. P.  Jacobs, B. L.  Davies, B. J. | 2023 | Single-center, pilot study evaluating feasibility of virtual reality to alleviate pain during transperineal prostate biopsy | Conference Abstract |
| 776 | Naur, T. M. H.  Nilsson, P. M.  Pietersen, P. I.  Clementsen, P. F.  Konge, L. | 2017 | Simulation-Based Training in Flexible Bronchoscopy and Endobronchial Ultrasound-Guided Transbronchial Needle Aspiration (EBUS-TBNA): A Systematic Review | Title-abstract exclusion |
| 777 | Nava, Alessandro  Mazza, Edoardo  Kleinermann, Frederic  Avis, Nick J.  McClure, John  Bajka, Michael | 2004 | Evaluation of the mechanical properties of human liver and kidney through aspiration experiments | Title-abstract exclusion |
| 778 | Nayahangan, Leizl Joy  Clementsen, Paul Frost  Paltved, Charlotte  Lindorff-Larsen, Karen Gilboe  Nielsen, Bjorn Ulrik  Konge, Lars | 2016 | Identifying Technical Procedures in Pulmonary Medicine That Should Be Integrated in a Simulation-Based Curriculum: A National General Needs Assessment | Title-abstract exclusion |
| 779 | Nayahangan, Leizl Joy  Hansen, Rikke Bolling  Lindorff-Larsen, Karen Gilboe  Paltved, Charlotte  Nielsen, Bjorn Ulrik  Konge, Lars | 2017 | Identifying content for simulation-based curricula in urology: a national needs assessment | Title-abstract exclusion |
| 780 | Naymagon, S.  Ullman, T. A. | 2015 | Chromoendoscopy and dysplasia surveillance in inflammatory bowel disease: Past, present, and future | Title-abstract exclusion |
| 781 | Nct, | 2008 | Video/Audio Distraction Analgesia for Simulated Oncology Procedure Pain | Title-abstract exclusion |
| 782 | Nct, | 2010 | Multi-Port Versus Single-port Cholecystectomy | Title-abstract exclusion |
| 783 | Nct, | 2013 | Virtual Reality With Ultrasound Versus Ultrasound For Central Line Insertion | Title-abstract exclusion |
| 784 | Nct, | 2014 | Mechanistic Approach to Preventing Atrophy and Restoring Function in Older Adults | Title-abstract exclusion |
| 785 | Nct, | 2016 | Impact of a Simulation-based Training Curriculum of Non-technical Skills on Colonoscopy Performance | Title-abstract exclusion |
| 786 | Nct, | 2018 | Virtual Reality in Reducing Pain and Anxiety in Cancer Participants Undergoing Painful Procedures | Trial registry record |
| 787 | Nct, | 2018 | Virtual Reality and Outpatient Hysteroscopy | Title-abstract exclusion |
| 788 | Nct, | 2018 | Virtual Reality for Needle Procedures in the Pediatric Emergency Department | Title-abstract exclusion |
| 789 | Nct, | 2018 | Trial Evaluating Hypnosis Using Virtural Reality vs Standard Pain Management During Musculoskelettal Biopsy | Title-abstract exclusion |
| 790 | Nct, | 2018 | Therapeutic Virtual Reality : impact on the Management of Pain and Anxiety Related to Hematology Care (REVEH) | Trial registry record |
| 791 | Nct, | 2018 | HoloLens: an Objective Alternative to the Operator's Memory | Title-abstract exclusion |
| 792 | Nct, | 2019 | e-Nature VR: evaluation of the Impact of Virtual Reality During Breast Biopsy | Title-abstract exclusion |
| 793 | Nct, | 2019 | Virtual Reality for the Treatment of Pain in Pediatric Vaccination | Title-abstract exclusion |
| 794 | Nct, | 2019 | Virtual Reality on Perception of Pain and Anxiety by Hysteroscopy | Title-abstract exclusion |
| 795 | Nct, | 2019 | Effect of Virtual Reality Distraction on Procedural Pain for Children and Adolescents in Onco-Hematology Unit | Title-abstract exclusion |
| 796 | Nct, | 2020 | VR Ultrasound Guided Breast Localization | Title-abstract exclusion |
| 797 | Nct, | 2020 | Impact of Relaxing Visual Immersion on Anxiety During Anti-cancer Treatment | Title-abstract exclusion |
| 798 | Nct, | 2020 | Virtual Reality in MVA for Miscarriage | Title-abstract exclusion |
| 799 | Nct, | 2020 | Clinical Efficacy of Virtual Reality During Office Hysteroscopy and Endometrial Biopsy in Subfertility | Trial registry record |
| 800 | Nct, | 2020 | Mixed Reality Technique Combined With 3D Printing Navigational Template for Localizing Pulmonary Nodules | Title-abstract exclusion |
| 801 | Nct, | 2020 | Virtual Reality: influence on Satisfaction, Pain, and Anxiety in Patients Undergoing Colposcopy | Title-abstract exclusion |
| 802 | Nct, | 2021 | The Use of Virtual Reality During Breast Ultrasound-Guided Biopsy Procedures | Trial registry record |
| 803 | Nct, | 2021 | Testing the Combination of Venetoclax and Rituximab, in Comparison to the Usual Treatment (Ibrutinib and Rituximab) for Waldenstrom's Macroglobulinemia/Lymphoplasmacytic Lymphoma | Title-abstract exclusion |
| 804 | Nct, | 2021 | Mixed-reality REBOA Simulator With Virtual Coaching | Title-abstract exclusion |
| 805 | Nct, | 2022 | Effect of Virtual Reality and Music Therapy on Pain Relief in Outpatient Hysteroscopy | Title-abstract exclusion |
| 806 | Nct, | 2022 | Effect of Virtual Reality Glasses in The Arteriovenous Fistule Cannulation Processon Paın and Patient Satisfaction | Title-abstract exclusion |
| 807 | Nct, | 2022 | Improving Patient Experience: BMBA | Trial registry record |
| 808 | Nct, | 2022 | Impact of Virtual Reality in Pediatric Hematology and Oncology | Title-abstract exclusion |
| 809 | Nct, | 2022 | Decreasing Patient Anxiety During Revascularization of Chronic Total Coronary Occlusions Using Virtual Reality Glasses | Title-abstract exclusion |
| 810 | Nct, | 2022 | The Effect of Virtual Reality Glasses Application on Pain, Anxiety, and Patient Satisfaction | Trial registry record |
| 811 | Nct, | 2022 | Use of Virtual Reality in Pain Management in Pediatric Oncology | Title-abstract exclusion |
| 812 | Nct, | 2022 | Virtual Reality Distraction During Arteriovenous Fistula Puncture | Title-abstract exclusion |
| 813 | Nct, | 2022 | The Effect of Virtual Reality on Psychological Parameters in Patients Receiving Chemotherapy | Title-abstract exclusion |
| 814 | Nct, | 2022 | Assessment of the Influence of the Virtual Reality Headset on Pain and Anxiety During Oocyte Retrieval Under Local Anesthesia | Trial registry record |
| 815 | Nct, | 2022 | Interest of Hypnosis in Virtual Reality on Nociception During Oocyte Retrieval in a Medically Assisted Reproduction Procedure | Title-abstract exclusion |
| 816 | Nct, | 2022 | Three Distraction Methods for Pain Reduction During Colonoscopy | Title-abstract exclusion |
| 817 | Nct, | 2022 | Leap Motion Controller for Pain During Venipuncture in Pediatrics | Title-abstract exclusion |
| 818 | Nct, | 2023 | Effect of Virtual Reality and Music Therapy on Pain Relief in OPH Operations | Title-abstract exclusion |
| 819 | Nct, | 2023 | The Effect of Virtual Reality on Anxiety and Pain During Endometrial Biopsy | Trial registry record |
| 820 | Nct, | 2023 | Virtual Reality for the Management of Pain and Anxiety for Outpatient Interventional Radiology Procedures | Trial registry record |
| 821 | Nct, | 2023 | Impact of Virtual Reality on Peri-interventional Pain, Anxiety and Distress in a Pediatric Oncology Outpatient Clinic | Title-abstract exclusion |
| 822 | Nct, | 2023 | The Effect of Virtual Reality Application on Pain Due to Fistula Puncture in Hemodialysis Patients | Title-abstract exclusion |
| 823 | Nct, | 2023 | Application of Mixed Reality Technique for Percutaneous Lung Nodule Localization: a Prospective, Randomized, Controlled Trial | Title-abstract exclusion |
| 824 | Nct, | 2024 | Virtual Reality in Invasive Techniques in Physiotherapy | Title-abstract exclusion |
| 825 | Nct, | 2024 | The Main Content of This Study is to Use Virtual Reality Technology Combined With Local Anesthesia With Lidocaine Injection at Different Times to Reduce the Pain of PICC in School-age Children | Title-abstract exclusion |
| 826 | Nct, | 2024 | Distraction-Based Interventions During Implanted Venous Port Catheter Needle Entry: virtual Reality Application | Title-abstract exclusion |
| 827 | Nct, | 2024 | Feasibility of Virtual Reality Assistance in Prostate Biopsy Under Local Anesthesia | Trial registry record |
| 828 | Nct, | 2024 | Virtual Reality Headset and/or Aromatherapy for Patients Undergoing a Transperineal Prostate Biopsy | Trial registry record |
| 829 | Nct, | 2024 | Augmented Reality Assisted Lung Nodule Localization: a Multicentered, Prospective, Randomly Controlled, Non-inferiority Trial | Title-abstract exclusion |
| 830 | Nct, | 2024 | Bliss DTx Assessment During Course Care Including Port-A-Catheter Setting | Title-abstract exclusion |
| 831 | Ndhaief, N.  Rezg, N.  Dziri, C.  Pitekelabou, E. H. | 2022 | Towards the Digitalization of the Healthcare Learning Process using 3D experience | Title-abstract exclusion |
| 832 | Nedbal, C.  Jahrreiss, V.  Cerrato, C.  Castellani, D.  Pietropaolo, A.  Galosi, A. B.  Somani, B. K. | 2023 | The Role of 'Artificial Intelligence, Machine Learning, Virtual Reality and Radiomics' in PCNL: A Review of Publication Trends Over the Last 30 Years | Title-abstract exclusion |
| 833 | Nelson, James E.  Mugford, Virginia R.  Kilcourse, Ellen  Wang, Richard S.  Kowdley, Kris V. | 2010 | Relationship between gene expression of duodenal iron transporters and iron stores in hemochromatosis subjects | Title-abstract exclusion |
| 834 | Nesbitt, Craig  Tingle, Samuel James  Williams, Robin  McCaslin, James  Searle, Roger  Mafeld, Sebastian  Stansby, Gerard | 2018 | A Pulsatile Fresh Frozen Human Cadaver Circulation Model for Endovascular Training: A Trial of Face Validity | Title-abstract exclusion |
| 835 | Nesbitt, Craig I.  Tingle, Samual J.  Williams, Robin  McCaslin, James E.  Searle, Roger  Mafeld, Sebastian  Stansby, Gerard P. | 2019 | Educational Impact of a Pulsatile Human Cadaver Circulation Model for Endovascular Training | Title-abstract exclusion |
| 836 | Nestel, D.  Kneebone, R.  Black, S.  Jones, N.  Horrocks, E.  Harrison, R.  Wetzel, C. | 2006 | Simulated patients and the development of procedural and operative skills [2] | Title-abstract exclusion |
| 837 | Neto, J. S.  Fonseca, E. A.  Cândido, H. L.  Pugliese, R.  Feier, F. H.  Kondo, M.  Azambuja, R. L.  Chapchap, P. | 2016 | Alternatives for vascular reconstruction in pediatric living donor liver transplantation | Title-abstract exclusion |
| 838 | Neubauer, H.  Li, M.  Müller, V. R.  Pabst, T.  Beer, M. | 2017 | Diagnostic Value of Diffusion-Weighted MRI for Tumor Characterization, Differentiation and Monitoring in Pediatric Patients with Neuroblastic Tumors | Title-abstract exclusion |
| 839 | Neumann, H.  Vieth, M.  Günther, C.  Neufert, C.  Kiesslich, R.  Grauer, M.  Atreya, R.  Neurath, M. F. | 2013 | Virtual chromoendoscopy for prediction of severity and disease extent in patients with inflammatory bowel disease: A randomized controlled study | Title-abstract exclusion |
| 840 | Ng, C. F. | 2014 | Training in percutaneous nephrolithotomy: The learning curve and options | Title-abstract exclusion |
| 841 | Ng, S. Y.  Lin, C. L. | 2022 | A Multilayered, Lesion-Embedded Ultrasound Breast Phantom with Realistic Visual and Haptic Feedback for Needle Biopsy | Title-abstract exclusion |
| 842 | Nguyen, N.  Lavery, W. J.  Capocelli, K. E.  Smith, C.  DeBoer, E. M.  Deterding, R.  Prager, J. D.  et al | 2019 | Transnasal Endoscopy in Unsedated Children With Eosinophilic Esophagitis Using Virtual Reality Video Goggles | Title-abstract exclusion |
| 843 | Nguyen, N.  Pan, Z.  Smith, C.  Friedlander, J. A. | 2024 | Transnasal endoscopy ease score “TNEase score” to evaluate patient tolerance of unsedated transnasal endoscopy | Title-abstract exclusion |
| 844 | Ni, Dong  Chan, Wing Yin  Qin, Jing  Chui, Yim Pan  Qu, Yingge  Ho, Simon S. M.  Heng, Pheng Ann | 2011 | A Virtual Reality Simulator for Ultrasound-Guided Biopsy Training | Title-abstract exclusion |
| 845 | Ni, D.  Chan, W. Y.  Qin, J.  Qu, Y.  Chui, Y. P.  Ho, S. S. M.  Heng, P. A. | 2008 | An ultrasound-guided organ biopsy simulation with 6DOF haptic feedback | Title-abstract exclusion |
| 846 | Nickolas, T. L.  Shirazian, S.  Shane, E. | 2010 | High-resolution computed tomography imaging: A virtual bone biopsy | Title-abstract exclusion |
| 847 | Nicolau, S.  Schmid, J.  Pennec, X.  Soler, L.  Ayache, N. | 2004 | An augmented reality & virtuality interface for a puncture guidance system: Design and validation on an abdominal phantom | Title-abstract exclusion |
| 848 | Nicolau, S. A.  Pennec, X.  Soler, L.  Ayache, N. | 2005 | A complete augmented reality guidance system for liver punctures: First clinical evaluation | Title-abstract exclusion |
| 849 | Nicolau, S. A.  Pennec, X.  Soler, L.  Buy, X.  Gangi, A.  Ayache, N.  Marescaux, J. | 2009 | An augmented reality system for liver thermal ablation: Design and evaluation on clinical cases | Title-abstract exclusion |
| 850 | Nillahoot, N.  Suthakorn, J. | 2013 | Development of Veress Needle Insertion Robotic System and its experimental study for force acquisition in soft tissue | Title-abstract exclusion |
| 851 | Nilsson, Stefan  Finnstrom, Berit  Kokinsky, Eva  Enskar, Karin | 2009 | The use of Virtual Reality for needle-related procedural pain and distress in children and adolescents in a paediatric oncology unit | Title-abstract exclusion |
| 852 | Nishida, S.  Ito, A.  Tsuchiya, A.  Urabe, K.  Yamamoto, K.  Amakawa, R.  Nagao, M.  Takizawa, A.  Ito, T. | 1985 | Acute idiopathic myocarditis having myocardial inflammatory swelling demonstrated by echocardiography: a case report | Title-abstract exclusion |
| 853 | Nishio, Kohei  Nakaguchi, Toshiyia | 2016 | Development of Haptic Needle for VR Based Injection Training System Using Simulated Patient | Title-abstract exclusion |
| 854 | Nl, Omon | 2021 | Virtual Reality hypnosis during needle related procedures in children | Title-abstract exclusion |
| 855 | Nl, Omon | 2022 | The effect of Virtual Reality on pain in patients undergoing lumbar puncture. A randomized controlled trial | Trial registry record |
| 856 | Noda, Y.  Goshima, S.  Kaga, T.  Ando, T.  Miyoshi, T.  Kawai, N.  Kawada, H.  Tanahashi, Y.  Matsuo, M. | 2020 | Virtual monochromatic image at lower energy level for assessing pancreatic ductal adenocarcinoma in fast kV-switching dual-energy CT | Title-abstract exclusion |
| 857 | Noguchi, M.  Deguchi, D.  Toriwaki, J.  Mori, K.  Mekada, Y.  Matsuoka, K. | 2006 | Evaluation of a prostate biopsy strategy for cancer detection using a computer simulation system with virtual needle biopsy for three-dimensional prostate models | Title-abstract exclusion |
| 858 | Nougaret, S.  Tibermacine, H.  Tardieu, M.  Sala, E. | 2019 | Radiomics: an Introductory Guide to What It May Foretell | Title-abstract exclusion |
| 859 | Noureldin, Y. A.  Elkoushy, M. A.  Andonian, S. | 2015 | Assessment of percutaneous renal access skills during urology objective structured clinical examinations (OSCE) | Title-abstract exclusion |
| 860 | Novak, Z.  Nadvornik, P.  Chrastina, J.  Riha, I. | 2005 | Neuroendoscopic management of haematocephalus | Title-abstract exclusion |
| 861 | Ock, Junhyeok  Moon, Sojin  Kim, Minkyeong  Ko, Beom Seok  Kim, Namkug | 2024 | Evaluation of the accuracy of an augmented reality-based tumor-targeting guide for breast-conserving surgery | Title-abstract exclusion |
| 862 | Oger, M.  Belhomme, P.  Gurcan, M. N. | 2012 | A general framework for the segmentation of follicular lymphoma virtual slides | Title-abstract exclusion |
| 863 | O'Hagan, J. J.  Samani, A. | 2009 | Measurement of the hyperelastic properties of 44 pathological ex vivo breast tissue samples | Title-abstract exclusion |
| 864 | Ohnsorge, J. A. K.  Siebert, C. H.  Schkommodau, E.  Mahnken, A. H.  Prescher, A.  Weisskopf, M. | 2005 | Minimally-invasive computer-assisted fluoroscopic navigation for kyphoplasty | Title-abstract exclusion |
| 865 | Okada, Atsushi  Noda, Yusuke  Ohashi, Kazuya  Okada, Tomoki  Chaya, Ryosuke  Kawase, Kengo  et al | 2020 | USEFULNESS OF DETERMINATION OF RENAL PUNCTURE LINE IN ECIRS USING VIRTUAL REALITY | Title-abstract exclusion |
| 866 | Okada, Atsushi  Ohashi, Kazuya  Noda, Yusuke  Okada, Tomoki  et al | 2022 | EFFECTIVENESS OF VIRTUAL REALITY SIMULATION OF RENAL PUNCTURE FOR ENDOSCOPIC-COMBINED INTRARENAL SURGERY | Title-abstract exclusion |
| 867 | Okada, D. H.  Binder, S. W.  Felten, C. L.  Strauss, J. S.  Marchevsky, A. M. | 1999 | 'virtual microscopy' and the internet as telepathology consultation tools: Diagnostic accuracy in evaluating melanocytic skin lesions | Title-abstract exclusion |
| 868 | Okamoto, E.  Sato, S.  Sanchez-Siles, A. A.  Ishine, J.  Miyake, T.  Amano, Y.  Kinoshita, Y. | 2010 | Evaluation of virtual CT sonography for enhanced detection of small hepatic nodules: A prospective pilot study | Title-abstract exclusion |
| 869 | Okimasa, Seiji  Shibata, Satoshi  Awaya, Yoshikazu  Nagao, Yukiyasu  Murakami, Isao  Shigeto, Eriko | 2007 | Virtual bronchoscopy aids management of lung cancer | Title-abstract exclusion |
| 870 | Okimasa, Seiji  Yoshioka, Shinkichiro  Shibata, Satoshi  Awaya, Yoshikazu  Nagao, Yukiyasu  Murakami, Isao  Shigeto, Eriko  Asahara, Toshimasa | 2007 | Endobronchial ultrasonography with a guide-sheath and virtual bronchoscopy navigation aids management of peripheral pulmonary nodules | Title-abstract exclusion |
| 871 | Okrainec, A.  Farcas, M.  Henao, O.  Choy, I.  Green, J.  Fotoohi, M.  Leslie, R.  Wight, D.  Karam, P.  Gonzalez, N.  Apkarian, J. | 2009 | Development of a virtual reality haptic veress needle insertion simulator for surgical skills training | Title-abstract exclusion |
| 872 | Okur, A.  Ahmadi, S. A.  Bigdelou, A.  Wendler, T.  Navab, N. | 2011 | MR in OR: First analysis of AR/VR visualization in 100 intra-operative Freehand SPECT acquisitions | Title-abstract exclusion |
| 873 | O'Leary, M. F.  Jackman, S. R.  Sabou, V. R.  Campbell, M. I.  Tang, J. C. Y.  Dutton, J.  Bowtell, J. L. | 2021 | Shatavari Supplementation in Postmenopausal Women Improves Handgrip Strength and Increases Vastus lateralis Myosin Regulatory Light Chain Phosphorylation but Does Not Alter Markers of Bone Turnover | Title-abstract exclusion |
| 874 | Oliver Jofre, Maria Isabel | 1993 | Evaluation of autonomic nervous system in non-alcoholic chronic liver disease | Title-abstract exclusion |
| 875 | Olmos, R. A. Valdes  Vidal-Sicart, S.  Giammarile, F.  Zaknun, J. J.  Van Leeuwen, F. W. B.  Mariani, G. | 2014 | The GOSTT concept and hybrid mixed/virtual/augmented reality environment radioguided surgery | Title-abstract exclusion |
| 876 | Onodera, Y.  Omatsu, T.  Takeuchi, S.  Shinagawa, N.  Yamazaki, K.  Nishioka, T.  Miyasaka, K. | 2004 | Enhanced virtual bronchoscopy using the pulmonary artery: Improvement in route mapping for ultraselective transbronchial lung biopsy | Title-abstract exclusion |
| 877 | Orlandi, D.  Viglino, U.  Dedone, G.  Leale, G.  Caruso, P.  Mauri, G.  Turtulici, G. | 20222 | US-CT fusion-guided percutaneous radiofrequency ablation of large substernal benign thyroid nodules | Title-abstract exclusion |
| 878 | Orr, M. E.  Gonzales, M. M.  Garbarino, V. R.  Zilli, E. M.  Peterson, R. C.  Kirkland, J. L.  Tchkonia, T.  Musi, N.  Seshadri, S.  Craft, S. | 2021 | Senolytic therapy to modulate the progression of alzheimer's disease (stomp-AD): methodology for a randomized, double-blind, placebo-controlled phase ii trial | Title-abstract exclusion |
| 879 | Orringer, Daniel A.  Golby, Alexandra  Jolesz, Ferenc | 2012 | Neuronavigation in the surgical management of brain tumors: current and future trends | Title-abstract exclusion |
| 880 | Orzechowski Westholm, J.  Espes, D.  Carlsson, P. O.  Benedict, C.  Cedernaes, J. | 2019 | Adverse insulin sensitivity profile associated with the observed increase in circulating hormone FGF-21 levels and altered peripheral tissue promoter dna methylation following acute sleep loss in humans | Title-abstract exclusion |
| 881 | O'Toole, R. V.  Playter, R. R.  Krummel, T. M.  Blank, W. C.  Cornelius, N. H.  Roberts, W. R.  Bell, W. J.  Raibert, M. | 20 | Measuring and developing suturing technique with a virtual reality surgical simulator | Title-abstract exclusion |
| 882 | Ow, A.  Tan, W.  Pienkowski, L. | 2015 | Mandibular reconstruction using a custom-made titanium prosthesis: A case report on the use of virtual surgical planning and computer-aided design/computer-aided manufacturing | Title-abstract exclusion |
| 883 | Paepke, S. Zacherl M. Schwarz-Boeger U. Humbert A. Kiechle M. Jacobs V. R. | 2004 | ADAM (axillary dissection with access minimized) - technique reduces significantly postoperative lymph seroma in breast conserving surgery | Title-abstract exclusion |
| 884 | Pagador, J. B.  Sanchez-Margallo, F. M.  Sanchez-Peralta, L. F.  Sanchez-Margallo, J. A.  Moyano-Cuevas, J. L.  Enciso-Sanz, S.  Uson-Gargallo, J.  Moreno, J. | 2012 | Decomposition and analysis of laparoscopic suturing task using tool-motion analysis (TMA): improving the objective assessment | Title-abstract exclusion |
| 885 | Pan, J.  Dogan, B. E.  Carkaci, S.  Santiago, L.  Arribas, E.  Cantor, S. B.  Wei, W.  Stafford, R. J.  Whitman, G. J. | 2013 | Comparing performance of the CADstream and the DynaCAD breast MRI CAD systems: CADstream vs. DynaCAD in breast MRI | Title-abstract exclusion |
| 886 | Pan, J.  Li, R.  Yu, D.  Wang, X.  Zheng, W.  Huang, X.  Zhu, B.  Zeng, H.  Liu, X. | 2021 | Augmented reality based surgical navigation for percutaneous endoscopic transforaminal discectomy | Title-abstract exclusion |
| 887 | Pan, Junjun  Yu, Dongfang  Li, Ranyang  Huang, Xin  Wang, Xinliang  Zheng, Wenhao  Zhu, Bin  Liu, Xiaoguang | 2021 | Multi-Modality guidance based surgical navigation for percutaneous endoscopic transforaminal discectomy | Title-abstract exclusion |
| 888 | Panebianco, V.  Pecoraro, M.  Novelli, S.  Catalano, C. | 2024 | Bridging the gap between human beings and digital twins in radiology | Title-abstract exclusion |
| 889 | Papanastasiou, S.  Estdale, S. E.  Homer-Vanniasinkam, S.  Mathie, R. T. | 1999 | Protective effect of preconditioning and adenosine pretreatment in experimental skeletal muscle reperfusion injury | Title-abstract exclusion |
| 890 | Papatsoris, A. G.  Shaikh, T.  Patel, D.  Bourdoumis, A.  Bach, C.  Buchholz, N.  Masood, J.  Junaid, I. | 2012 | Use of a Virtual Reality Simulator to Improve Percutaneous Renal Access Skills: A Prospective Study in Urology Trainees | Title-abstract exclusion |
| 891 | Park, C. H.  Jung, S. W.  Shin, J. W.  Bae, M. A.  Lee, Y. I.  Park, Y. T.  Chung, H. S.  Park, N. H. | 2016 | Comparison of tenofovir plus lamivudine versus tenofovir monotherapy in patients with lamivudine-resistant chronic hepatitis B | Title-abstract exclusion |
| 892 | Park, Jin Seo  Jung, Yong-Wook  Lee, Jun Won  Shin, Dong Sun  Chung, Min Suk  Riemer, Martin  Handels, Heinz | 2008 | Generating useful images for medical applications from the Visible Korean Human | Title-abstract exclusion |
| 893 | Park, S. J.  Oh, S. H.  Kang, M. S.  Kim, T. H.  Kang, S. W.  Yoon, Y. C.  Kim, Y. H. | 2014 | Reuse of a previously transplanted kidney from a deceased donor using luminex virtual crossmatching: A case report | Title-abstract exclusion |
| 894 | Parks, E. T. | 2001 | Basic principles of computed tomography | Title-abstract exclusion |
| 895 | Parwani, A. V.  Amin, M. B. | 2020 | Convergence of Digital Pathology and Artificial Intelligence Tools in Anatomic Pathology Practice: Current Landscape and Future Directions | Title-abstract exclusion |
| 896 | Pascual, E. A.  Herrero, C. G.  Navallas, M.  Robinot, D. C.  Vilalta, M. P. | 2023 | Virtual reality glasses: distraction technique to reduce sedation rates in children undergoing interventional radiology procedures | Title-abstract exclusion |
| 897 | Pasha, F.  Abazari, S.  Bikarannejad, P.  Zabolian, A. | 2019 | Systemic sclerosis with focus on scleroderma renal crisis | Title-abstract exclusion |
| 898 | Patel, P.  El Husseiny, T.  Moraitis, K.  Shaikh, T.  Buchholz, N.  Masood, J.  Junaid, I. | 2010 | Assessing and developing percutaneous renal access skills of trainees using the state of the art PERC Mentor™ simulation trainer | Title-abstract exclusion |
| 899 | Paterson, A. H.  Taylor, D. M.  McCready, V. R. | 1975 | A clinical comparison of the tumour-imaging radiopharmaceuticals 67Gallium-citrate and 111Indium-labelled bleomycin | Title-abstract exclusion |
| 900 | Pawlik, Michal  Brzozowski, Tomasz  Konturek, Peter C.  Targosz, Anetta  Drozdowicz, Danuta  Burnat, Greg  Konturek, Stanislaw J.  Pawlik, Wieslaw W.  Hahn, Eckhart G. | 2006 | Role of endocannabinoids in gastroprotection against acute gastric lesions | Title-abstract exclusion |
| 901 | Peck, M.  Yiasemides, E.  Badrick, T. | 2018 | An Australian Mohs external quality assurance program | Title-abstract exclusion |
| 902 | Peddi, V. R.  Marder, B.  Gaite, L.  Oberholzer, J.  Goldberg, R.  et al., | 2020 | Treatment of De Novo Renal Transplant Recipients with Calcineurin Inhibitor (CNI)-Free, Belatacept+Everolimus-Based Immunosuppression | Title-abstract exclusion |
| 903 | Pellicori, P.  Doolub, G.  Wong, C. M.  Lee, K. S.  Mangion, K.  et al., | 2021 | COVID‐19 and its cardiovascular effects: a systematic review of prevalence studies | Title-abstract exclusion |
| 904 | Pellisé, M.  Llach, J.  Bordas, J. M. | 2005 | Emerging endoscopic techniques. The arrival of virtual histology | Title-abstract exclusion |
| 905 | Peng, Yanjun  Ma, Yingran  Wang, Yuanhong  Shan, Junliang | 2017 | The application of interactive dynamic virtual surgical simulation visualization method | Title-abstract exclusion |
| 906 | Peng, Yilong  Xie, Zhengyuan  Chen, Shaoai  Wu, Yi  Dong, Jiajun  Li, Jinhong  He, Jinlang  Chen, Xiaolei  Gao, Hongzhi | 2023 | Application effect of head-mounted mixed reality device combined with 3D printing model in neurosurgery ventricular and hematoma puncture training | Title-abstract exclusion |
| 907 | Peper, Jordan M. J.  Kalivas, John H. | 2024 | Redefining Spectral Data Analysis with Immersive Analytics: Exploring Domain-Shifted Model Spaces for Optimal Model Selection | Title-abstract exclusion |
| 908 | Pepley, D. F.  Gordon, A. B.  Yovanoff, M. A.  Mirkin, K. A.  Miller, S. R.  Han, D. C.  Moore, J. Z. | 2017 | Training Surgical Residents With a Haptic Robotic Central Venous Catheterization Simulator | Title-abstract exclusion |
| 909 | Perenic, Emma  Grember, Emilie  Bassard, Sebastien  Koutlidis, Nicolas | 2023 | Impact of virtual reality on pain management in transrectal MRI-guided prostate biopsy | Meeting abstract |
| 910 | Perez-Moneo, B.  Bellido, M. G.  Mena, E. B.  Agapito, Map- M.  Garcia, L. C.  Fuerte, R. B. | 2024 | Virtual reality in the outpatient: reducing anxiety and fear in venous puncture | Title-abstract exclusion |
| 911 | Pérez-Pachón, L.  Poyade, M.  Brown, J.  Fallon, V. | 2017 | Towards the development of a virtual reality simulator with haptic force feedback for training in stereotactic brain biopsies | Title-abstract exclusion |
| 912 | Perrakis, A.  Yedibela, S.  Schuhmann, S.  Croner, R.  Schellerer, V.  Demir, R.  Hohenberger, W.  Müller, V. | 2011 | The effect and safety of the treatment of recurrent hepatitis C infection after orthotopic liver transplantation with pegylated interferon α2b and ribavirin | Title-abstract exclusion |
| 913 | Perrotti, M.  Pantuck, A.  Rabbani, F.  Israeli, R. S.  Weiss, R. E. | 1999 | Review of staging modalities in clinically localized prostate cancer | Title-abstract exclusion |
| 914 | Peterlík, I.  Golembiovský, T.  Duriez, C.  Cotin, S. | 2014 | Complete Real-Time Liver Model Including Glisson's Capsule, Vascularization and Parenchyma | Title-abstract exclusion |
| 915 | Petersen, K. L.  Rice, F. L.  Suess, F.  Berro, M.  Rowbotham, M. C. | 2002 | Relief of post-herpetic neuralgia by surgical removal of painful skin | Title-abstract exclusion |
| 916 | Petronilho, F.  Vuolo, F.  Galant, L. S.  Constantino, L.  Tomasi, C. D.  et al., | 2012 | Gastrin-releasing peptide receptor antagonism induces protection from lethal sepsis: involvement of toll-like receptor 4 signaling | Title-abstract exclusion |
| 917 | Pfandler, Michael  Lazarovici, Marc  Stefan, Philipp  Wucherer, Patrick  Weigl, Matthias | 2017 | Virtual reality-based simulators for spine surgery: a systematic review | Title-abstract exclusion |
| 918 | Pieler, A.  Ipsen, H. K. | 2022 | Using Virtual Reality (VR) to help children cope with joint puncture related to JIA | Title-abstract exclusion |
| 919 | Pilcher, T. A. | 2019 | The Role of Atrial Arrhythmia Ablation in Adolescent and Adult Congenital Heart Disease | Title-abstract exclusion |
| 920 | Pinski, Jacek K.  Mohacsi, Savannah  Nelson, Genevieve Viva  Ghani, Umair  Roy, Varun Yadav  Pai, Jonathan  Robles, Mars  Groshen, Susan G.  Xiong, Shigang | 2023 | Effects of virtual reality on pain and anxiety in patients with cancer undergoing bone marrow biopsy | Meeting Abstract |
| 921 | Piramide, Federico  Amparore, Daniele  Checcucci, Enrico  De Cillis, Sabrina T.  Piana, Alberto  Volpi, Gabriele  et al | 2024 | HOW TO MAXIMIZE ROBOTIC RADICAL PROSTATECTOMY OUTCOMES: SURGICAL AND NEW GENERATION IMAGING TIPS AND TRICKS | Title-abstract exclusion |
| 922 | Pirotte, Benoit J. M.  Levivier, Marc  Goldman, Serge  Massager, Nicolas  Wikler, David  Dewitte, Olivier  Bruneau, Michael  Rorive, Sandrine  David, Philippe  Brotchi, Jacques | 2009 | POSITRON EMISSION TOMOGRAPHY-GUIDED VOLUMETRIC RESECTION OF SUPRATENTORIAL HIGH-GRADE GLIOMAS: A SURVIVAL ANALYSIS IN 66 CONSECUTIVE PATIENTS | Title-abstract exclusion |
| 923 | Pisano, G.  Wendler, T.  Valdés Olmos, R. A.  Garganese, G.  Rietbergen, D. D. D.  Giammarile, F.  Vidal-Sicart, S.  Oonk, M. H. M.  Frumovitz, M.  Abu-Rustum, N. R.  Scambia, G.  Rufini, V.  Collarino, A. | 2024 | Molecular image–guided surgery in gynaecological cancer: where do we stand? | Title-abstract exclusion |
| 924 | Pîslă, D.  Gherman, B.  Gîrbacia, F.  Vaida, C.  Butnariu, S.  Gîrbacia, T.  Plitea, N. | 2016 | Optimal planning of needle insertion for robotic-assisted prostate biopsy | Title-abstract exclusion |
| 925 | Planz, V.  Franzen, L.  Windbergs, M. | 2015 | Novel in vitro approaches for the simulation and analysis of human skin wounds | Title-abstract exclusion |
| 926 | Polasek, Thomas M.  Rostami-Hodjegan, Amin | 2020 | Virtual Twins: Understanding the Data Required for Model-Informed Precision Dosin | Title-abstract exclusion |
| 927 | Pomykala, Kelsey L.  Herrmann, Ken  Emmett, Louise  Lalumera, Elisabetta  Fanti, Stefano | 2022 | Virtual Prostate Biopsy with Prostate-specific Membrane Antigen and Magnetic Resonance Imaging: Closer to Reality in a Subgroup of Prostate Cancer Patients? | Title-abstract exclusion |
| 928 | Popa, D. O.  Singh, S. K. | 1998 | Creating realistic force sensations in a virtual environment: Experimental system, fundamental issues and result | Title-abstract exclusion |
| 929 | Porpiglia, Francesco  Bertolo, Riccardo  Amparore, Daniele  Checcucci, Enrico  et al | 2018 | Augmented reality during robot-assisted radical prostatectomy: expert robotic surgeons' on-the-spot insights after live surgery | Title-abstract exclusion |
| 930 | Porpiglia, Francesco  Checcucci, Enrico  Amparore, Daniele  Autorino, Riccardo  Piana, Alberto  et al | 2019 | Augmented-reality robot-assisted radical prostatectomy using hyper-accuracy three-dimensional reconstruction (HA3D™) technology: a radiological and pathological study | Title-abstract exclusion |
| 931 | Porpiglia, F.  Checcucci, E.  Amparore, D.  De Cillis, S.  Volpi, G.  et al | 2021 | Artificial intelligence 3D augmented reality guided biopsy during robotic prostatectomy: A new way to reduce the positive surgical margin rate | Title-abstract exclusion |
| 932 | Porpiglia, Francesco  Checcucci, Enrico  Amparore, Daniele  Peretti, Dario  Piramide, Federico  et al | 2022 | Percutaneous Kidney Puncture with Three-dimensional Mixed-reality Hologram Guidance: From Preoperative Planning to Intraoperative Navigation | Title-abstract exclusion |
| 933 | Porpiglia, Francesco  Checcucci, Enrico  Amparore, Daniele  Peretti, Dario  Piramide, Federico  De Cillis, Sabrina  Verri, Paolo  Niculescu, Razvan Gabriel  Poggio, Massimiliano  Cossu, Marco  Fiori, Cristian | 2020 | KIDNEY STONES SURGICAL TREATMENT WITH 3 D MIXED REALITY ASSISTANCE FOR PERCUTANEOUS PUNCTURE | Title-abstract exclusion |
| 934 | Porpiglia, Francesco  Checcucci, Enrico  Amparore, Daniele  Piana, Alberto  Volpi, Gabriele  Piazzolla, Pietro  Manfrin, Diego  Fiori, Cristian | 2019 | 3D ELASTIC AUGMENTED REALITY ROBOT-ASSITED RADICAL PROSTATECTOMY: FURTHER EVOLUTION OF THE STANDARD PROCEDURE | Title-abstract exclusion |
| 935 | Porpiglia, Francesco  Fiori, Cristian  Checcucci, Enrico  Amparore, Daniele  Bertolo, Riccardo | 2018 | Augmented Reality Robot-assisted Radical Prostatectomy: Preliminary Experience | Title-abstract exclusion |
| 936 | Poté, N.  Cauchy, F.  Albuquerque, M.  Cros, J.  Soubrane, O.  Bedossa, P.  Paradis, V. | 2018 | Contribution of virtual biopsy to the screening of microvascular invasion in hepatocellular carcinoma: A pilot study | Title-abstract exclusion |
| 937 | Prabhu, V. G.  Sprouse, H. A.  Brignull, C. G.  Snider, R.  Tanner, S.  Adams, K. J.  Nisonson, A. B.  Hand, W. R.  Epling, J. A. | 2024 | The Impact of Virtual Reality on Anxiety and Pain During US-Guided Breast Biopsies: A Randomized Controlled Clinical Trial | Included |
| 938 | Pritchett, Michael A.  Bhadra, Krish  Calcutt, Mike  Folch, Erik | 2020 | Virtual or reality: divergence between preprocedural computed tomography scans and lung anatomy during guided bronchoscopy | Title-abstract exclusion |
| 939 | Priya, S.  Nagpal, P. | 2021 | Virtual modeling and interactive virtual reality display of unusual high-riding cervical aortic arch | Title-abstract exclusion |
| 940 | Profeta, Andrea Corrado  Schilling, Clare  McGurk, Mark | 2016 | Augmented reality visualization in head and neck surgery: an overview of recent findings in sentinel node biopsy and future perspectives | Title-abstract exclusion |
| 941 | Puliatti, S.  Sighinolfi, M. C.  Rocco, B.  Patel, V.  Francesco, P.  Micali, S.  Eissa, A.  Torricelli, P.  Bianchi, G. | 2019 | First live case of augmented reality robot-assisted radical prostatectomy from 3D magnetic resonance imaging reconstruction integrated with PRECE model (Predicting Extracapsular extension of prostate cancer) | Title-abstract exclusion |
| 942 | Purkable, T. L.  Bauer, J. J. | 1999 | A telementored trans-rectal ultrasound guided prostate biopsy | Title-abstract exclusion |
| 943 | Qazi, Y.  Shaffer, D.  Kaplan, B.  Kim, D. Y.  Luan, F. L.  Peddi, V. R.  Shihab, F.  Tomlanovich, S.  Yilmaz, S.  McCague, K.  et al., | 2017 | Efficacy and Safety of Everolimus Plus Low-Dose Tacrolimus Versus Mycophenolate Mofetil Plus Standard-Dose Tacrolimus in De Novo Renal Transplant Recipients: 12-Month Data | Title-abstract exclusion |
| 944 | Qian, Jiandan  Zhang, Chi  Liu, Huaie  Wang, Guiqiang  Zhao, Hong | 2022 | Serum HBV RNA as a predictor of virological response in treatment-naive chronic HBeAg-positive HBV-infected patients with normal alanine aminotransferase | Title-abstract exclusion |
| 945 | Qian, K.  Krimsky, W. S.  Sarkar, S. A.  Deng, Y. | 2020 | Efficiency of Electromagnetic Navigation Bronchoscopy and Virtual Bronchoscopic Navigation | Title-abstract exclusion |
| 946 | Qidwai, U.  Akbar, M. A. | 2016 | Image stitching system with scanning microscopy for histopathological applications | Title-abstract exclusion |
| 947 | Qin, J.  Li, S.  Wu, H. S.  Wang, L. S.  Wang, X. X.  Wong, T. T.  Yu, C. M.  Heng, P. A. | 2012 | Towards an interactive simulation system for percutaneous coronary intervention training | Title-abstract exclusion |
| 948 | Qin, Shuchao  Jiang, Rui  Dai, Luomengjia  Miao, Yi  Sha, Yeqin  et al | 2024 | Venetoclax plus dose-adjusted R-EPOCH (VR-DA-EPOCH) or G-EPOCH bridging to subsequent cellular therapy for the patients with transformed lymphoma a single center clinical experience | Title-abstract exclusion |
| 949 | Raabe, Clemens  Fichtner, Jens  Beck, Jurgen  Gralla, Jan  Raabe, Andreas | 2018 | Revisiting the rules for freehand ventriculostomy: a virtual reality analysis | Title-abstract exclusion |
| 950 | Raghunath, S.  Rajagopalan, S.  Karwoski, R. A.  Larson, A. G.  Bartholmai, B. J.  Robb, R. A. | 2012 | Detail-on-demand visualization for lean understanding of lung abnormalities | Title-abstract exclusion |
| 951 | Rahman, Rafa  Wood, Matthew E.  Qian, Long  Price, Carrie L.  Johnson, Alex A.  Osgood, Greg M. | 2020 | Head-Mounted Display Use in Surgery: A Systematic Review | Title-abstract exclusion |
| 952 | Rajakariar, R.  Jivanji, N.  Varagunam, M.  Rafiq, M.  Gupta, A.  Sheaff, M.  Sinnott, P.  Yaqoob, M. M. | 2005 | High pre-transplant soluble CD30 levels are predictive of the grade of rejection | Title-abstract exclusion |
| 953 | Ralph, H. K. | 20 | 2020 European Society of Human Genetics, June 6–June 9th: Live in your living room! | Title-abstract exclusion |
| 954 | Rapetti, L.  Crivellaro, S.  De Momi, E.  Ferrigno, G.  Niederberger, C.  Luciano, C. | 2017 | Virtual reality navigation system for prostate biopsy | Title-abstract exclusion |
| 955 | Rassweiler, J.  Müller, M.  Fangerau, M.  Klein, J.  Meinzer, H. P.  Teber, D. | 2012 | Ipad-assisted percutaneous access to the kidney - Initial experience | Title-abstract exclusion |
| 956 | Rassweiler, J.  Rassweiler, M. C.  Klein, J. | 2016 | New technology in ureteroscopy and percutaneous nephrolithotomy | Title-abstract exclusion |
| 957 | Rassweiler, Jens  Rassweiler, Marie Claire  Mueller, Michael  Klein, Jan  Teber, Dogu  Pereira, Phillipe  Meinzer, Hans Peter | 2012 | IPAD-ASSISTED PUNCTURE OF THE KIDNEY - EXPERIMENTAL EVALUATION AND EARLY CLINICAL EXPERIENCE | Title-abstract exclusion |
| 958 | Rassweiler, J.  Rassweiler, M. C.  Müller, M.  Kenngott, H.  Meinzer, H. P.  et al | 2014 | Surgical navigation in urology: European perspective | Title-abstract exclusion |
| 959 | Rassweiler, J.  Rassweiler, M. C.  Müller, M.  Klein, J.  Teber, D.  Pereira, P.  Meinzer, H. P. | 2012 | IPAD-assisted puncture of the kidney-experimental evaluation and early clinical experience | Title-abstract exclusion |
| 960 | Rassweiler, Jens  Rassweiler, Marie-Claire  Klein, Jan | 2016 | New technology in ureteroscopy and percutaneous nephrolithotomy | Title-abstract exclusion |
| 961 | Rassweiler, Jens  Rassweiler, Marie-Claire  Mueller, Michael  Kenngott, Hannes  Meinzer, Hans-Peter  Teber, Dogu  Esut Expert Grp | 2014 | Surgical navigation in urology: European perspective | Title-abstract exclusion |
| 962 | Rassweiler-Seyfried, Marie-Claire  Rassweiler, J. J.  Weiss, C.  Mueller, M.  Meinzer, H. P.  Maier-Hein, L.  Klein, J. T. | 2020 | iPad-assisted percutaneous nephrolithotomy (PCNL): a matched pair analysis compared to standard PCNL | Title-abstract exclusion |
| 963 | Rath, Timo  Morgenstern, Nadine  Vitali, Francesco  Atreya, Raja  Neurath, Markus F. | 2020 | Advanced Endoscopic Imaging in Colonic Neoplasia | Title-abstract exclusion |
| 964 | Reddy, V. K.  Lavoie, M. C.  Verbeek, J. H.  Pahwa, M. | 2017 | Devices for preventing percutaneous exposure injuries caused by needles in healthcare personnel | Title-abstract exclusion |
| 965 | Reichel, J. L.  Peirson, R. P.  Berg, D. | 2004 | Teaching and evaluation of surgical skills in dermatology - Results of a survey | Title-abstract exclusion |
| 966 | Reichmann, J.  Verleden, S. E.  Kühnel, M.  Kamp, J. C.  et al | 2023 | Human lung virtual histology by multi-scale x-ray phase-contrast computed tomography | Title-abstract exclusion |
| 967 | Reitze, Alicia  Voigt, Marie  Klawonn, Frank  Dusch, Martin  Grigull, Lorenz  Muecke, Urs | 2024 | Impact of virtual reality on peri-interventional pain, anxiety and distress in a pediatric oncology outpatient clinic: a randomized controlled trial | Title-abstract exclusion |
| 968 | Rentschler, M. E.  Dumpert, J.  Platt, S. R.  Farritor, S. M.  Oleynikov, D. | 2006 | Mobile in vivo biopsy and camera robot | Title-abstract exclusion |
| 969 | Reus, A. D.  El-Harbachi, H.  Rousian, M.  Exalto, N.  Steegers-Theunissen, R. P. M.  Steegers, E. P. | 2011 | An Innovative way of 3D placental volume measurements in early pregnancy | Title-abstract exclusion |
| 970 | Reynolds, K.  Zablah, J. | 2022 | Use of Virtual Reality in Case Preparation for Catheter Intervention in Congenitally Corrected Transposition of the Great Arteries: A Case Series | Title-abstract exclusion |
| 971 | Ribeiro De Oliveira, T. M.  Henriques Pereira, S.  Osório, L.  Dragos, L.  et al | 2024 | Structured technical skills training with the Transurethral Training (TUT) Curriculum: development process of one of the core curricula of the European Association of Urology (EAU) Standardization in Surgical Education (SISE) program | Title-abstract exclusion |
| 972 | Ricca, A.  Chellali, A. | 2016 | Interaction fidelity in virtual simulators: Two navigation techniques for a virtual biopsy trainer | Title-abstract exclusion |
| 973 | Ricca, A.  Chellali, A.  Otmane, S. | 2017 | Study of interaction fidelity for two viewpoint changing techniques in a virtual biopsy trainer | Title-abstract exclusion |
| 974 | Ricca, Aylen  Chellali, Amine  Otmane, Samir | 2021 | Comparing touch-based and head-tracking navigation techniques in a virtual reality biopsy simulator | Title-abstract exclusion |
| 975 | Ridout, B.  Kelson, J.  Campbell, A.  Steinbeck, K. | 2021 | Effectiveness of virtual reality interventions for adolescent patients in hospital settings: Systematic review | Title-abstract exclusion |
| 976 | Rietbergen, D. D. D.  Meershoek, P.  van Oosterom, M. N.  Roestenberg, M.  van Erkel, A. R.  Smit, F.  van der Hage, J. A.  Valdés Olmos, R. A.  van Leeuwen, F. W. B. | 2019 | Freehand-SPECT with 99mTc-HDP as tool to guide percutaneous biopsy of skeletal lesions detected on bone scintigraphy | Title-abstract exclusion |
| 977 | Rios-Hernandez, M.  Jacinto-Villegas, J. M.  Vilchis-Gonzalez, A. H.  Zemiti, N.  Padilla-Castaneda, M. A. | 2022 | Virtual lumbar puncture simulators: Where are we today? | Title-abstract exclusion |
| 978 | Ríos-Hernández, M.  Jacinto-Villegas, J. M.  Zemiti, N.  Vilchis-González, A. H.  Padilla-Castañeda, M. A.  Debien, B. | 2023 | Development of a lumbar puncture virtual simulator for medical students training: A preliminary evaluation | Title-abstract exclusion |
| 979 | Ritacco, Lucas E.  Milano, Federico  Aponte Tinao, Luis  Risk, Marcelo  et al | 2011 | Realidad virtual: Su aplicación en cirugía reconstructiva oncológica esquelética Presentación de un caso de osteosarcoma tibial | Title-abstract exclusion |
| 980 | Ritter, E. M.  Cox, T. C.  Trinca, K. D.  Pearl, J. P. | 2013 | Simulated Colonoscopy Objective Performance Evaluation (SCOPE): a non-computer-based tool for assessment of endoscopic skills | Title-abstract exclusion |
| 981 | Robb, R. A. | 1997 | Virtual endoscopy: evaluation using the visible human datasets and comparison with real endoscopy in patients | Title-abstract exclusion |
| 982 | Robb, R. A. | 2000 | Virtual endoscopy: development and evaluation using the Visible Human Datasets | Title-abstract exclusion |
| 983 | Robb, R. A. | 2002 | Three-dimensional visualization and analysis in prostate cancer | Title-abstract exclusion |
| 984 | Robb, R. A.  Aharon, S.  Cameron, B. M. | 1997 | Patient-specific anatomic models from three dimensional medical image data for clinical applications in surgery and endoscopy | Title-abstract exclusion |
| 985 | Robinson, Christian  Clarkson, Thomas  Zarrabi, Amir | 2022 | Effect of virtual reality (VR) as analgesia for trans-rectal ultrasound prostate biopsy (TRUS Bx) on pain severity: a prospectively randomized study | Meeting Abstract |
| 986 | Robiony, Massimo | 2010 | Distraction Osteogenesis: A Method to Improve Facial Balance in Asymmetric Patients | Title-abstract exclusion |
| 987 | Rochlen, Lauryn R.  Levine, Robert  Tait, Alan R. | 2017 | First-Person Point-of-View-Augmented Reality for Central Line Insertion Training | Title-abstract exclusion |
| 988 | Rodd, C. D.  Velchuru, V. R.  Holly-Archer, F.  Clark, A.  Pereira, J. H. | 2007 | Randomized clinical trial comparing two mastectomy techniques | Title-abstract exclusion |
| 989 | Rodríguez-Vila, B.  Gutiérrez, A.  Peral-Boiza, M.  Ying, H.  Gómez-Fernández, T.  Gómez, E. J.  Sánchez-González, P. | 2018 | A low-cost pedagogical environment for training on technologies for image-guided robotic surgery | Title-abstract exclusion |
| 990 | Roehr, Mark  Wu, Teresa  Maykowski, Philip  Munter, Bryce  Hoebee, Shelby  Daas, Eshaan  Kang, Paul | 2021 | The Feasibility of Virtual Reality and Student-Led Simulation Training as Methods of Lumbar Puncture Instruction | Title-abstract exclusion |
| 991 | Roggeri, A.  Agostini, L.  Vezzani, G.  Sabattini, E.  Serra, L. | 1993 | Primary malignant non-Hodgkin's lymphoma of the lung arising in mucosa-associated lymphoid tissue (MALT) | Title-abstract exclusion |
| 992 | Roignot, Patrick  Donzel, Jean-Paul  Brunaud, Marie-Dominique | 2011 | The use of virtual slides in the daily practice of a pathology laboratory | Title-abstract exclusion |
| 993 | Rolland, J. P.  Lee, K. S.  Khoudeir, L.  Meemon, P.  Thompson, K. P.  Huang, J.  Yao, J.  Ibrahim, S. F. | 2012 | Virtual skin biopsy with Gabor Domain optical coherence microscopy | Title-abstract exclusion |
| 994 | Ros, Maxime  Neuwirth, Lorenz S.  Ng, Sam  Debien, Blaise  Molinari, Nicolas  Gatto, Franck  Lonjon, Nicolas | 2021 | The Effects of an Immersive Virtual Reality Application in First Person Point-of-View (IVRA-FPV) on The Learning and Generalized Performance of a Lumbar Puncture Medical Procedure | Title-abstract exclusion |
| 995 | Rose, S. C.  Nelson, T. R.  Deutsch, R. | 2004 | Display of 3-dimensional ultrasonographic images for interventional procedures - Volume-rendered versus multiplanar display | Title-abstract exclusion |
| 996 | Rosenthal, Annika  Ebrahimi, Claudia  Wedemeyer, Friederike  Romanczuk-Seiferth, Nina  Beck, Anne | 2022 | The Treatment of Substance Use Disorders: Recent Developments and New Perspectives | Title-abstract exclusion |
| 997 | Rosenthal, M.  State, A.  Lee, J.  Hirota, G.  Ackerman, J.  Keller, K.  Pisano, E. D.  Jiroutek, M.  Muller, K.  Fuchs, H. | 2002 | Augmented reality guidance for needle biopsies: An initial randomized, controlled trial in phantoms | Title-abstract exclusion |
| 998 | Ross, S. G.  Thali, M. J.  Bolliger, S.  Germerott, T.  Ruder, T. D.  Flach, P. M. | 2012 | Sudden death after chest pain: Feasibility of virtual autopsy with postmortem CT angiography and biopsy | Title-abstract exclusion |
| 999 | Roth, C. G.  Marzio, D. H. D.  Guglielmo, F. F. | 2018 | Contributions of Magnetic Resonance Imaging to Gastroenterological Practice: MRIs for GIs | Title-abstract exclusion |
| 1000 | Rovetta, A. | 1999 | Tests on reliability of a prostate biopsy telerobotic system | Title-abstract exclusion |
| 1001 | Rovetta, A.  Bejczy, A. K.  Sala, R. | 1997 | Telerobotic surgery: Applications on human patients and training with virtual reality | Title-abstract exclusion |
| 1002 | Rovetta, A.  Sala, R.  Bressanelli, M.  Lorini, F.  Pegoraro, R.  Canina, M. | 1998 | Demonstration of surgical telerobotics and virtual telepresence by internet+isdn from monterey (USA) to milan (Italy) | Title-abstract exclusion |
| 1003 | Rowley, Keri J.  Liss, Michael A. | 2020 | Systematic Review of Current Ultrasound Use in Education and Simulation in the Field of Urology | Title-abstract exclusion |
| 1004 | Ruan, P.  Zhou, B.  Dai, X.  Sun, Z.  Guo, X.  Huang, J.  Gong, Z. | 2014 | Predictive value of intrahepatic hepatitis B virus covalently closed circular DNA and total DNA in patients with acute hepatitis B and patients with chronic hepatitis B receiving anti-viral treatment | Title-abstract exclusion |
| 1005 | Rueger, Christoph  Feufel, Markus A.  Moosburner, Simon  Oezbek, Christopher  Pratschke, Johann  Sauer, Igor M. | 2020 | Ultrasound in augmented reality: a mixed-methods evaluation of head-mounted displays in image-guided interventions | Title-abstract exclusion |
| 1006 | Rust, G. F.  Sackmann, M.  Eisele, O.  Reiser, M. | 2001 | Virtual colonoscopy with multi-slice computed tomography - An alternative to conventional colonoscopy? | Title-abstract exclusion |
| 1007 | Rustina, Y.  Effendi, D.  Nurlaela, S. | 2023 | Cope children's anxious behavior and pain with virtual reality during venipuncture procedure in emergency department | Title-abstract exclusion |
| 1008 | Rutkove, S. B.  Sanchez, B. | 2019 | Electrical impedance methods in neuromuscular assessment: An overview | Title-abstract exclusion |
| 1009 | Ryu, J. H.  Han, S. H.  Hwang, S. M.  Lee, J.  Do, S. H.  Kim, J. H.  Park, J. W. | 2022 | Effects of Virtual Reality Education on Procedural Pain and Anxiety During Venipuncture in Children: A Randomized Clinical Trial | Title-abstract exclusion |
| 1010 | Saba, P.  Shepard, L.  Nithipalan, V.  Holler, T.  Rashid, H.  Quarrier, S.  Ghazi, A. | 2022 | Design and development of a high-fidelity transrectal ultrasound (TRUS) simulation model for remote education and training | Title-abstract exclusion |
| 1011 | Saccenti, Laetitia  Bessy, Hugo  Ben Jedidia, Bilel  Longere, Benjamin  Tortolano, Lionel  Derbel, Haytham  Luciani, Alain  Kobeiter, Hicham  Grandpierre, Thierry  Tacher, Vania | 2024 | Performance Comparison of Augmented Reality Versus Ultrasound Guidance for Puncture: A Phantom Study | Title-abstract exclusion |
| 1012 | Sadeghi, R.  Zakavi, S. R.  Forghani, M. N.  Aryana, K.  Kakhki, V. R.  Ayati, N. K.  Shandiz, F. H.  Ghavamnasiri, M. R.  Keshtgar, M. | 2010 | The efficacy of Tc-99m sestamibi for sentinel node mapping in breast carcinomas: comparison with Tc-99m antimony sulphide colloid | Title-abstract exclusion |
| 1013 | Sáez-Rodríguez, D. J.  Chico-Sánchez, P. | 2021 | Application of virtual reality in hospitalized oncology children subjected to invasive procedures: A case study | Title-abstract exclusion |
| 1014 | Safi, A.  Castaneda, V.  Lasser, T.  Navab, N. | 20 | Skin lesions classification with optical spectroscopy | Title-abstract exclusion |
| 1015 | Sainsbury, Ben  Wilz, Olivia  Ren, Jing  Green, Mark  Fergie, Martin  Rossa, Carlos | 2022 | Preoperative Virtual Reality Surgical Rehearsal of Renal Access during Percutaneous Nephrolithotomy: A Pilot Study | Title-abstract exclusion |
| 1016 | Saitou, Y.  Shiraki, K.  Kawakita, T.  Inoue, H.  Okano, H.  Yamamoto, N.  Sugimoto, K.  Muraki, K.  Nakano, T. | 2003 | Oninvasive prediction of fibrosis by a novel serum marker in patients with chronic hepatitis C | Title-abstract exclusion |
| 1017 | Sakakibara, J.  Nagashima, T.  Fujimoto, H.  Takada, M.  Ohtsuka, M. | 2023 | A review of MRI (CT)/US fusion imaging in treatment of breast cancer | Title-abstract exclusion |
| 1018 | Salgueero, R.  Borman, A. M.  Herrtage, M.  Benchekroun, G.  Abbondati, E.  Piola, V.  Vanhaesebrouck, A. | 2013 | Rasamsonia argillacea mycosis in a dog: first case in Europe | Title-abstract exclusion |
| 1019 | Salleh, Rosli | 2001 | Minimally invasive surgery training and tele-surgery system using vr and haptic techniques | Title-abstract exclusion |
| 1020 | Samani, Abbas  Zubovits, Judit  Plewes, Donald | 2007 | Elastic moduli of normal and pathological human breast tissues: an inversion-technique-based investigation of 169 samples | Title-abstract exclusion |
| 1021 | Samei, Golnoosh  Tsang, Keith  Kesch, Claudia  Lobo, Julio  Hor, Soheil  Mohareri, Omid  Chang, Silvia  Goldenberg, S. Larry  Black, Peter C.  Salcudean, Septimiu | 2020 | A partial augmented reality system with live ultrasound and registered preoperative MRI for guiding robot-assisted radical prostatectomy | Title-abstract exclusion |
| 1022 | Sánchez-Caballero, E.  Ortega-Donaire, L.  Sanz-Martos, S. | 2024 | Immersive Virtual Reality for Pain and Anxiety Management Associated with Medical Procedures in Children and Adolescents: A Systematic Review | Title-abstract exclusion |
| 1023 | Sander Wint, Suzanne  Eshelman, Debra  Steele, Jill  Guzzetta, Cathie E. | 2002 | Effects of distraction using virtual reality glasses during lumbar punctures in adolescents with cancer | Title-abstract exclusion |
| 1024 | Sandholzer, M. A.  Errickson, D.  Walter, B. S. | 2013 | AAFS 2013: Current issues and future trends in forensic radiology and imaging | Title-abstract exclusion |
| 1025 | Sanicola, Henry  Stewart, Caleb  Fletcher, Stephen  Brooks, Morgan  Ware, Marcus  Sarkar, Korak | 2019 | Planning a Glioma Partial Resection with DTI Tractography and Virtual Reality | Title-abstract exclusion |
| 1026 | Sankaranarayanan, R.  Nene, B. M.  Shastri, S. S.  Jayant, K.  Muwonge, R.  Budukh, A. M.  Hingmire, S.  Malvi, S. G.  Thorat, R.  Kothari, A.  et al., | 2009 | HPV screening for cervical cancer in rural India | Title-abstract exclusion |
| 1027 | Sanna, G.  Camporesi, A.  Diotto, V.  Abbiati, G.  Torri, A.  Gemma, M. | 2024 | Virtual sedation as a substitute to pharmacological sedation during PICC placement in pediatric patients: A feasibility study | Title-abstract exclusion |
| 1028 | Sano, A.  Tsuchiya, T. | 2014 | Virtual bronchoscopy using OsiriX | Title-abstract exclusion |
| 1029 | Santamaria, A.  Lóbez, T.  García-Barcenilla, S.  García-Pérez, L.  Sánchez-Ruiz, M.  Farriol, J.  García, D. | 2023 | 360° VIRTUAL REALITY INCREASES TREATMENT ADHERENCE AND EMPOWERMENT IN PEOPLE WITH HEMOPHILIA: PRELIMINARY RESULTS | Title-abstract exclusion |
| 1030 | Sappenfield, Joshua Warren  Smith, William Brit  Cooper, Lou Ann  Lizdas, David  Gonsalves, Drew B.  Gravenstein, Nikolaus  Lampotang, Samsun  Robinson, Albert R., III | 2018 | Visualization Improves Supraclavicular Access to the Subclavian Vein in a Mixed Reality Simulator | Title-abstract exclusion |
| 1031 | Sapuk, A.  Steinhoff, L.  Huenninghaus, K.  Willuweit, K.  Rashidi Alavijeh, J.  Hild, B.  Asar, L.  Schmidt, H. H.  Schramm, C. | 2024 | Long-Term Treatment with Bulevirtide in Patients with Chronic Hepatitis D and Advanced Chronic Liver Disease | Title-abstract exclusion |
| 1032 | Sara Sixtos-Alonso, Maria  Sanchez-Munoz, Fausto  Francisco Sanchez-Avila, Juan  Avalos Martinez, Rosalba  Dominguez Lopez, Aaron  Vargas Vorackova, Florencia  Uribe, Misael | 2011 | IFN-stimulated Gene Expression Is a Useful Potential Molecular Marker of Response to Antiviral Treatment with Peg-IFNα 2b and Ribavirin in Patients with Hepatitis C Virus Genotype 1 | Title-abstract exclusion |
| 1033 | Sasidhar, M. | 2010 | Navigation guidance for bronchoscopy "are we there yet?" | Title-abstract exclusion |
| 1034 | Satava, R. M. | 1997 | Virtual reality and telepresence surgery | Title-abstract exclusion |
| 1035 | Sathia Narayanan, Madusudanan | 2014 | Quantitative Evaluation of User Performance in Minimally Invasive Surgical Procedures | Title-abstract exclusion |
| 1036 | Sato, M.  Chen, F.  Aoyama, A.  Yamada, T.  Ikeda, M.  Bando, T.  Date, H. | 2013 | Virtual endobronchial ultrasound for transbronchial needle aspiration | Title-abstract exclusion |
| 1037 | Satoh, Makoto  Nakajima, Takeshi  Yamaguchi, Takashi  Watanabe, Eiju  Kawai, Kensuke | 2019 | Application of Augmented Reality to Stereotactic Biopsy | Title-abstract exclusion |
| 1038 | Sauer, F.  Khamene, A.  Bascle, B.  Vogt, S. | 2002 | An augmented reality system for ultrasound guided needle biopsies | Title-abstract exclusion |
| 1039 | Sauer, F.  Schoepf, U. J.  Khamene, A.  Vogt, S.  Das, M.  Silverman, S. G. | 2003 | Augmented reality system for CT-guided interventions: System description and initial phantom trials | Title-abstract exclusion |
| 1040 | Sauerbronn, A. V.  Fonseca, A. M.  Bagnoli, V. R.  Saldiva, P. H.  Pinotti, J. A. | 2000 | The effects of systemic hormonal replacement therapy on the skin of postmenopausal women | Title-abstract exclusion |
| 1041 | Savage, N. | 2017 | Machine learning: Calculating disease | Title-abstract exclusion |
| 1042 | Sawaya, R.  Rambo Jr, W. M.  Hammoud, M. A.  Ligon, B. L. | 1995 | Advances in surgery for brain tumors | Title-abstract exclusion |
| 1043 | Scarlata, S.  Palermo, P.  Candoli, P.  Tofani, A.  Petitti, T.  Corbetta, L. | 2017 | EBUS-STAT Subscore Analysis to Predict the Efficacy and Assess the Validity of Virtual Reality Simulation for EBUS-TBNA Training among Experienced Bronchoscopists | Title-abstract exclusion |
| 1044 | Schaake, Ryan  Leopold, Ian  Sandberg, Alexander  Zenk, Brianna  Shafer, London  Yu, Daohai  Lu, Xiaoning  Theingi, Shunn  Udongwo, Angela  Cohen, Gary S.  Maresky, Hillel S. | 204 | Virtual Reality for the Management of Pain and Anxiety for IR Procedures: A Prospective, Randomized, Pilot Study on Digital Sedation | Woring outcomes |
| 1045 | Schawkat, Khoschy  Krajewski, Katherine M. | 2023 | Insights into Renal Cell Carcinoma with Novel Imaging Approaches | Title-abstract exclusion |
| 1046 | Schmitz, R.  Krause, J.  Krech, T.  Rösch, T. | 2018 | Virtual Endoscopy Based on 3-Dimensional Reconstruction of Histopathology Features of Endoscopic Resection Specimens | Title-abstract exclusion |
| 1047 | Schnelldorfer, T.  Jenkins, R. L.  Birkett, D. H.  Wright, V. J.  Price, L. L.  Georgakoudi, I. | 2016 | Laparoscopic narrow band imaging for detection of occult cancer metastases: a randomized feasibility trial | Title-abstract exclusion |
| 1048 | Schniepp, Roman  Trabold, Raimund  Romagna, Alexander  Akrami, Farhoud  Hesselbarth, Kristin  Wuehr, Max  Peraud, Aurelia  Brandt, Thomas  Dieterich, Marianne  Jahn, Klaus | 2017 | Walking assessment after lumbar puncture in normal-pressure hydrocephalus: a delayed improvement over 3 days | Title-abstract exclusion |
| 1049 | Schoeb, D. S.  Rassweiler, J.  Sigle, A.  Miernik, A.  Engels, C.  Goezen, A. S.  Teber, D. | 2021 | Robotics and intraoperative navigation | Title-abstract exclusion |
| 1050 | Scholten, H. J.  Pourtaherian, A.  Mihajlovic, N.  Korsten, H. H. M.  Bouwman, R. A. | 2017 | Improving needle tip identification during ultrasound-guided procedures in anaesthetic practice | Title-abstract exclusion |
| 1051 | Scholz, B. | 2002 | Towards virtual electrical breast biopsy: Space-frequency MUSIC for trans-admittance data | Title-abstract exclusion |
| 1052 | Schreen, W.  Friederich, K.  John, A. | 2024 | Use of virtual reality to reduce perioperative anxiety and pain in urologic interventions – a randomized controlled prospective study | Conference Abstract |
| 1053 | Schuster, L.  Valor-Méndez, L.  Wacker, J.  Dannhardt-Thiem, V.  Schmidt, A.  Knitza, J.  Simon, D.  Manger, B.  Schett, G.  Kleyer, A. | 2024 | “Rheuma (be-)greifen”— A multimodal teaching concept to improve rheumatology education for medical students | Title-abstract exclusion |
| 1054 | Schutyser, V.  Buyl, R.  Vos, M. D.  Tournaye, H.  Blockeel, C. | 2021 | Clinical efficacy of virtual reality for acute pain and anxiety management during outpatient hysteroscopy and endometrial biopsy in subfertile patients | Title-abstract exclusion |
| 1055 | Schwarz, Y. | 2010 | Electromagnetic Navigation | Title-abstract exclusion |
| 1056 | Sclaverano, S.  Chevreau, G.  Vadcard, L.  Mozer, P.  Troccaz, J. | 2009 | BiopSym: A simulator for enhanced learning of ultrasound-guided prostate biopsy | Title-abstract exclusion |
| 1057 | Scott, A.  McDonnell, M. J.  Ahmed, M.  Flannery, A.  Mujammil, I.  Breen, D.  Rutherford, R. M. | 2018 | Survey of management of iatrogenic bleeding following bronchoscopic sampling | Title-abstract exclusion |
| 1058 | Scott, S. N.  Shepherd, S. O.  Hopkins, N.  Dawson, E. A.  Strauss, J. A.  Wright, D. J.  Cooper, R. G.  Kumar, P.  Wagenmakers, A. J. M.  Cocks, M. | 2019 | Home-hit improves muscle capillarisation and eNOS/NAD(P)Hoxidase protein ratio in obese individuals with elevated cardiovascular disease risk | Title-abstract exclusion |
| 1059 | Sebbag, Lionel  Harrington, Danielle M.  Mochel, Jonathan P. | 2018 | Tear fluid collection in dogs and cats using ophthalmic sponges | Title-abstract exclusion |
| 1060 | Selim, Mostafa  Dresscher, Douwe  Abayazid, Momen | 2024 | Virtual Needle Insertion with Enhanced Haptic Feedback for Guidance and Needle-Tissue Interaction Forces | Title-abstract exclusion |
| 1061 | Selmi, S. Y.  Fiard, G.  Promayon, E.  Vadcard, L.  Troccaz, J. | 2013 | A virtual reality simulator combining a learning environment and clinical case database for image-guided prostate biopsy | Title-abstract exclusion |
| 1062 | Şen, H.  Lafcı Bakar, D. | 2024 | The effect of virtual reality glasses on pain and patient satisfaction in arteriovenous fistula cannulation procedure | Title-abstract exclusion |
| 1063 | Sendi, Hemen | 2024 | Nigropterix cummingi sp. n., an umenocoleid (Insecta: Dictyoptera) from mid-Cretaceous northern Myanmar amber | Title-abstract exclusion |
| 1064 | Septans, A. L.  Le Dû, K.  Maloisel, F.  Vanquaethem, H.  Schmitt, A.  Le Goff, M.  Moles, M. P.  Zinger, M.  Bourgeois, H.  Peron, M.  Denis, F.  Bouchard, S. | 2021 | A new option in pain prevention with bliss©, a digital therapeutic solution leveraging virtual reality: Results of a french open-label multicenter randomized phase III study (Reveh trial) | Title-abstract exclusion |
| 1065 | Sessanna, D.  Stredney, D.  Hittle, B.  Lambert, D. | 2008 | Simulation of punch biopsies: A case study | Title-abstract exclusion |
| 1066 | Seung, S. L.  Kyoung, W. K.  Beom, J. P.  Yong, M. S.  Pyo, N. K.  Lee, M. G.  Sung, G. L. | 2007 | Effect of respiration on the spectral Doppler wave of the right hepatic vein in right lobe living donor liver transplant recipients | Title-abstract exclusion |
| 1067 | Sewry, C. A.  Sansome, A.  Clerk, A.  Sherratt, T. G.  Hasson, N.  Rodillo, E.  Heckmatt, J. Z.  Strong, P. N.  Dubowitz, V. | 1993 | Manifesting carriers of Xp21 muscular dystrophy; lack of correlation between dystrophin expression and clinical weakness | Title-abstract exclusion |
| 1068 | Shah, R.  Agarwal, A.  Kavoussi, P.  Rambhatla, A.  Saleh, R.  Cannarella, R.  Harraz, A. M.  et al | 2022 | Consensus and Diversity in the Management of Varicocele for Male Infertility: Results of a Global Practice Survey and Comparison with Guidelines and Recommendations | Title-abstract exclusion |
| 1069 | Shah, V. R.  Bhosale, G. P. | 2010 | Spinal anaesthesia in young patients: evaluation of needle gauge and design on technical problems and postdural puncture headache | Title-abstract exclusion |
| 1070 | Shahzad, U. B.  Aiman, U.  Ahmed, M. | 2024 | Pioneering comfort in kidney biopsies: the role of hypnosis, virtual reality, and artificial intelligence | Title-abstract exclusion |
| 1071 | Shaikh, A.  Patel, N.  Nair, D.  Campbell, K. N. | 2020 | Current Paradigms and Emerging Opportunities in Nephrology Training | Title-abstract exclusion |
| 1072 | Shallik, N.  Elarref, M.  Khamash, O.  Abdelaal, A.  Alkhafaji, M. R.  Makki, H.  Abusabeib, A.  Moustafa, A.  Menon, A. | 2021 | Management of critical tracheal stenosis with a straw sized tube (Tritube): Case report | Title-abstract exclusion |
| 1073 | Shao, Long  Yang, Shuo  Fu, Tianyu  Lin, Yucong  Geng, Haixiao  Ai, Danni  Fan, Jingfan  Song, Hong  Zhang, Tao  Yang, Jian | 2022 | Augmented reality calibration using feature triangulation iteration-based registration for surgical navigation | Title-abstract exclusion |
| 1074 | Shen, Y.  Fulgham, P.  Zhou, X.  Burke, D.  Sweet, R. | 2009 | The Wii™ transrectal ultrasonography simulator | Title-abstract exclusion |
| 1075 | Shenoy, V.  Khan, S. I.  Lee, E.  Aalami, O. | 2021 | Vascular1: Development and Evaluation of a Virtual Reality Ultrasound Guided Vascular Access Training Module | Title-abstract exclusion |
| 1076 | Shi, G.  Wang, Z.  Wang, X. | 2023 | STUDY ON DIGITAL TWIN ORGAN MAPPING PREDICTION METHOD OF TUMOR PUNCTURE TARGET DEVIATION CAUSED BY PATIENT RESPIRATION | Title-abstract exclusion |
| 1077 | Shi, Yangyang  Deng, Xuesong  Tong, Yuqi  Li, Ruotong  Zhang, Yanfang  Ren, Lijie  Si, Weixin | 2022 | Synergistic Digital Twin and Holographic Augmented-Reality-Guided Percutaneous Puncture of Respiratory Liver Tumor | Title-abstract exclusion |
| 1078 | Shiba-Ishii, Aya  Isagawa, Takayuki  Shiozawa, Toshihiro  Mato, Naoko  Nakagawa, Tomoki  Takada, Yurika  et al | 2024 | Novel therapeutic strategies targeting bypass pathways and mitochondrial dysfunction to combat resistance to RET inhibitors in NSCLC | Title-abstract exclusion |
| 1079 | Shiffman, M. L.  Hofmann, C. M.  Luketic, V. A.  Thompson, E. B.  Sanyal, A. J. | 1996 | A randomized controlled trial comparing interferon-2b alone to a combination of interferon (INF) plus granulocyte-macrophage colony stimulating factor (GMCSF) for treatment of chronic hepatitis C virus (HCV) | Title-abstract exclusion |
| 1080 | Shihab, F.  Qazi, Y.  Mulgaonkar, S.  McCague, K.  Patel, D.  Peddi, V. R.  Shaffer, D. | 2017 | Association of Clinical Events With Everolimus Exposure in Kidney Transplant Patients Receiving Low Doses of Tacrolimus | Title-abstract exclusion |
| 1081 | Shihab, F. S.  Qazi, Y.  Mulgaonkar, S. P.  McCague, K. M.  Patel, D.  Peddi, V. R.  Shaffer, D. | 2016 | Everolimus with Low-Dose Tacrolimus versus Standard Immunosuppressive Regimen: subgroup Analysis of Renal Function at 12 Months in De Novo Renal Transplant Patients | Title-abstract exclusion |
| 1082 | Shimoga, K. B.  Khosla, P. K. | 1994 | Visual and force feedback to aid neurosurgical probe insertion | Title-abstract exclusion |
| 1083 | Shimura, Tetsuro  Higami, Hirooki  Matsuo, Hitoshi  Yamamoto, Masanori | 2023 | Appropriate assessment using virtual reality simulation for a novel reshaped curve sheath during percutaneous left atrial appendage closure: a follow-up case report | Title-abstract exclusion |
| 1084 | Shin, D. S.  Chung, M. S.  Park, J. S.  Park, H. S.  Lee, S. B.  Lee, S. H.  Choi, H. N.  Riemer, M.  Handels, H.  Lee, J. E.  Jung, W. | 2011 | Three-dimensional surface models of detailed lumbosacral structures reconstructed from the Visible Korean | Title-abstract exclusion |
| 1085 | Shinagawa, N.  Yamazaki, K.  Onodera, Y.  Asahina, H.  Kikuchi, E.  Asano, F.  Miyasaka, K.  Nishimura, M. | 2007 | Factors related to diagnostic sensitivity using an ultrathin bronchoscope under CT guidance | Title-abstract exclusion |
| 1086 | Shirk, Joseph D.  Reiter, Robert  Wallen, Eric M.  Pak, Ray  Ahlering, Thomas  Badani, Ketan K.  Porter, James R. | 2022 | Effect of 3-Dimensional, Virtual Reality Models for Surgical Planning of Robotic Prostatectomy on Trifecta Outcomes: A Randomized Clinical Trial | Title-abstract exclusion |
| 1087 | Shkel, A.  Natarajan, S.  Schimpf, S.  Culjat, M. O.  et al | 20 | A transurethral catheter-based ultrasound system for multi-modal fusion | Title-abstract exclusion |
| 1088 | Shrestha, P.  Geffner, C.  Jaffey, M.  Wu, Z.  Iapichino, M.  Bacca, M.  Stoeber, B. | 2024 | Force decomposition and toughness estimation from puncture experiments in soft solids | Title-abstract exclusion |
| 1089 | Si, Weixin  Liao, Xiangyun  Qian, Yinling  Wang, Qiong | 2018 | Mixed Reality Guided Radiofrequency Needle Placement: A Pilot Study | Title-abstract exclusion |
| 1090 | Siew, K.  Li, Z.  Walsh, S. B. | 2022 | A Novel Pipeline for the Tissue Transformation, 3D Imaging, and Visualisation in Virtual Space of Optically Cleared Human Renal Tissue | Title-abstract exclusion |
| 1091 | Silvia, R. | 2009 | MDCT diagnostic evaluation of tracheal and primary bronchial pathology | Title-abstract exclusion |
| 1092 | Simpfendoerfer, T.  Hatiboglu, G.  Hadaschik, B. A.  Wild, E.  Maier-Hein, L.  Rassweiler, M. C.  Rassweiler, J.  Hohenfellner, M.  Teber, D. | 2015 | Navigation in urological surgery. Possibilities and limits of current techniques | Title-abstract exclusion |
| 1093 | Simpfendörfer, T.  Baumhauer, M.  Müller, M.  Gutt, C. N.  Meinzer, H. P.  Rassweiler, J. J.  Guven, S.  Teber, D. | 2011 | Augmented reality visualization during laparoscopic radical prostatectomy | Title-abstract exclusion |
| 1094 | Sinclair, S. B.  Greig, P. D.  Blendis, L. M.  Abecassis, M.  Roberts, E. A.  Phillips, M. J.  Cameron, R.  Levy, G. A. | 1989 | Biochemical and clinical response of fulminant viral hepatitis to administration of prostaglandin E. A preliminary report | Title-abstract exclusion |
| 1095 | Sindram, D.  Lau, K. N.  Martinie, J. B.  Iannitti, D. A. | 2010 | Human pilot trial of novel real-time 3d and virtual reality (VR) guided ultrasound (US) targeting system during open microwave ablation (MWA) of liver tumors | Title-abstract exclusion |
| 1096 | Singapogu, R.  Jagannathan, A.  Nagarajan, N.  Moody, C.  Zhang, G.  Cull, D. | 2016 | A capacitance-based sensor for hemodialysis cannulation training: A proof-of-concept study | Title-abstract exclusion |
| 1097 | Singleton, H.  Hodder, A.  Almilaji, O.  Ersser, S. J.  Heaslip, V.  O'Meara, S.  Boyers, D.  Roberts, A.  Scott, H.  Van Onselen, J.  et al., | 2024 | Educational and psychological interventions for managing atopic dermatitis (eczema) | Title-abstract exclusion |
| 1098 | Singson, R. P. C.  Natarajan, S.  Greenson, J. K.  Marchevsky, A. M. | 1999 | Virtual microscopy and the internet as telepathology consultation tools: A study of gastrointestinal biopsy specimens | Title-abstract exclusion |
| 1099 | Sinha, A.  Johnson, S.  Hunt, C.  Woolnough, H.  Vidal, F. P.  John, N. W.  Villard, P. F.  Holbray, R.  Bello, F.  Gould, D. A. | 2009 | Importance of virtual reality simulators in interventional radiology: The ImaGiNe-S CIRSE 2008 experience | Title-abstract exclusion |
| 1100 | Sinha, Maneesh  Krishnamoorthy, Venkatesh | 2015 | Use of a vegetable model as a training tool for PCNL puncture | Title-abstract exclusion |
| 1101 | Siongers, E.  Bellens, M.  Vandeneede, E.  Broekmans, S.  Vergote, V.  et al | 20 | Virtual reality in bone marrow biopsy and aspiration: safety and user experience | Meeting Abstract |
| 1102 | Sixtos-Alonso, M. S.  Sánchez-Muñoz, F.  Sánchez-Ávila, J. F.  Martínez, R. A.  Domínguez López, A.  Vargas Vorácková, F.  Uribe, M. | 2011 | IFN-stimulated gene expression is a useful potential molecular marker of response to antiviral treatment with peg-IFNα 2b and ribavirin in patients with hepatitis C virus genotype 1 | Title-abstract exclusion |
| 1103 | Slaton, J. W.  Karashima, T.  Perrotte, P.  Inoue, K.  Kim, S. J.  et al | 2001 | Treatment with low-dose interferon-α restores the balance between matrix metalloproteinase-9 and E-cadherin expression in human transitional cell carcinoma of the bladder | Title-abstract exclusion |
| 1104 | Smadi, Yamen  Thomas, Jessina  Bittar, Khaled  Norton, Hannah  Friedlander, Joel A.  Bornstein, Jeffrey | 2024 | Office-based sedation-free transnasal esophagogastroduodenoscopy with biopsies using single-use gastroscopes: A pediatric single-center experience | Title-abstract exclusion |
| 1105 | Soyer, O. M.  Baran, B.  Ormeci, A. C.  Gokturk, S.  Aydın, E.  Onel, D.  Gulluoglu, M.  Karaca, C.  Akyuz, F.  Demir, K.  Besisik, F.  Kaymakoglu, S. | 2016 | Comparison of the efficacy of 12 months and longer courses of interferon therapy for the treatment of chronic delta hepatitis: a retrospective cohort study | Title-abstract exclusion |
| 1106 | Sparwasser, P.  Haack, M.  Frey, L.  Boehm, K.  Boedecker, C.  Huber, T.  Stroh, K.  Brandt, M. P.  Mager, R.  Höfner, T.  Tsaur, I.  Haferkamp, A.  Borgmann, H. | 2022 | Assessment of a novel smartglass-based point-of-care fusion approach for mixed reality-assisted targeted prostate biopsy: A pilot proof-of-concept study | Title-abstract exclusion |
| 1107 | Sporea, I. | 2011 | Romanian ultrasonography in Vienna | Title-abstract exclusion |
| 1108 | Sporea, I. | 2012 | Is there a real future for liver elastography? | Title-abstract exclusion |
| 1109 | Squara, Fabien  Bateau, Jules  Scarlatti, Didier  Bun, Sok-Sithikun  Moceri, Pamela  Ferrari, Emile | 2024 | Virtual Reality for the Management of Pain and Anxiety in Patients Undergoing Implantation of Pacemaker or Implantable Cardioverter Defibrillator: A Randomized Study | Title-abstract exclusion |
| 1110 | Staffolani, S.  Buonfrate, D.  Angheben, A.  Gobbi, F.  Giorli, G.  Guerriero, M.  Bisoffi, Z.  Barchiesi, F. | 2018 | Acute histoplasmosis in immunocompetent travelers: A systematic review of literature | Title-abstract exclusion |
| 1111 | State, A.  Keller, K.  Rosenthal, M.  Yang, H.  Ackerman, J.  Fuchs, H. | 2003 | Stereo imagery from the UNC augmented reality system for breast biopsy guidance | Title-abstract exclusion |
| 1112 | State, Andrei  Livingston, Mark A.  Garrett, William F.  Hirota, Gentaro  Whitton, Mary C.  Pisano, Etta D.  Fuchs, Henry | 1996 | Technologies for augmented reality systems: Realizing ultrasound-guided needle biopsies | Title-abstract exclusion |
| 1113 | Stather, David R.  MacEachern, Paul  Chee, Alex  Dumoulin, Elaine  Tremblay, Alain | 2012 | Evaluation of clinical endobronchial ultrasound skills following clinical versus simulation training | Title-abstract exclusion |
| 1114 | Stather, David R.  MacEachern, Paul  Rimmer, Karen  Hergott, Christopher A.  Tremblay, Alain | 2011 | Validation of an Endobronchial Ultrasound Simulator: Differentiating Operator Skill Level | Title-abstract exclusion |
| 1115 | Stather, David R.  Maceachern, Paul  Rimmer, Karen  Hergott, Christopher A.  Tremblay, Alain | 2011 | Assessment and learning curve evaluation of endobronchial ultrasound skills following simulation and clinical training | Title-abstract exclusion |
| 1116 | Steers, W. D. | 2009 | This Month in Adult Urology | Title-abstract exclusion |
| 1117 | Stern, Joshua  Zeltser, Ilia S.  Pearle, Margaret S. | 2007 | Percutaneous renal access simulators | Title-abstract exclusion |
| 1118 | Stevens, P. E.  Ahmed, S. B.  Carrero, J. J.  Foster, B.  Francis, A.  et al | 2024 | KDIGO 2024 Clinical Practice Guideline for the Evaluation and Management of Chronic Kidney Disease | Title-abstract exclusion |
| 1119 | Stone, Nelson N.  Wilson, Michael P.  Griffith, Steven H.  Immerzeel, Jos  Debruyne, Frans  Gorin, Michael A.  Brisbane, Wayne  Orio, Peter F.  Kim, Laura S.  Stone, Jonathan J. | 2022 | Remote surgical education using synthetic models combined with an augmented reality headset | Title-abstract exclusion |
| 1120 | Su, H.  Camilo, A.  Cole, G. A.  Hata, N.  Tempany, C. M.  Fischer, G. S. | 2011 | High-field MRI-compatible needle placement robot for prostate interventions | Title-abstract exclusion |
| 1121 | Su, Xiao H.  Deng, Zhen  He, Bin W.  Liu, Yu Q. | 2020 | Haptic-based virtual reality simulator for lateral ventricle puncture operation | Title-abstract exclusion |
| 1122 | Suarez-Ahedo, C.  Lopez-Reyes, A.  Martinez-Armenta, C.  Martinez-Gomez, L. E.  Martinez-Nava, G. A.  Pineda, C.  Vanegas-Contla, D. R.  Domb, B. | 2023 | Revolutionizing orthopedics: a comprehensive review of robot-assisted surgery, clinical outcomes, and the future of patient care | Title-abstract exclusion |
| 1123 | Sugiura, R.  Kuwatani, M.  Nishida, M.  Hirata, K.  et al | 2019 | Correlation between Liver Elasticity by Ultrasound Elastography and Liver Functional Reserve | Title-abstract exclusion |
| 1124 | Sukumar, P.  Ananth, S.  Kessler, D.  Maniker, R.  Friberg, R.  Gopalakrishnan, R.  Schlager, B.  Feiner, S. | 2024 | Visual Guidance for Infant Lumbar Puncture Training in XR | Title-abstract exclusion |
| 1125 | Snorek, M.  Bulava, A.  Vonke, I. | 2017 | Chronic lymphocytic leukemia skin infiltration mimicking an ICD pocket infection: a case report | Title-abstract exclusion |
| 1126 | Soerge, P.  Makowski, L.  Schippert, C.  Staboulidou, I.  Hille, U.  Hillemanns, P. | 2012 | The cost efficiency of HPV vaccines is significantly underestimated due to omission of conization-associated prematurity with neonatal mortality and morbidity | Title-abstract exclusion |
| 1127 | Solbiati, Marco  Ierace, Tiziana  Muglia, Riccardo  Pedicini, Vittorio  Iezzi, Roberto  Passera, Katia M.  Rotilio, Alessandro C.  Goldberg, S. Nahum  Solbiati, Luigi A. | 2022 | Thermal Ablation of Liver Tumors Guided by Augmented Reality: An Initial Clinical Experience | Title-abstract exclusion |
| 1128 | Sonnemans, Lianne J. P.  Kubat, Bela  Prokop, Mathias  Klein, Willemijn M. | 2018 | Can virtual autopsy with postmortem CT improve clinical diagnosis of cause of death? A retrospective observational cohort study in a Dutch tertiary referral centre | Title-abstract exclusion |
| 1129 | Souza, I. A.  Sanches Jr, C.  Zuffo, M. K. | 2009 | A virtual reality simulator for training of needle biopsy of thyroid gland nodules | Title-abstract exclusion |
| 1130 | Souza, I. A.  Zuffo, M. K. | 2007 | Needle biopsy simulator of thyroid gland nodules | Title-abstract exclusion |
| 1131 | Sumdani, H.  Aguilar-Salinas, P.  Avila, M. J.  Barber, S. R.  Dumont, T. | 2022 | Utility of Augmented Reality and Virtual Reality in Spine Surgery: A Systematic Review of the Literature | Title-abstract exclusion |
| 1132 | Suzuki, Kazufumi  Morita, Satoru  Endo, Kenji  Yamamoto, Takahiro  Fujii, Shuhei  Ohya, Jun  Masamune, Ken  Sakai, Shuji | 2021 | Learning effectiveness of using augmented reality technology in central venous access procedure: an experiment using phantom and head-mounted display | Title-abstract exclusion |
| 1133 | Suzuki, Kazufumi  Morita, Satoru  Endo, Kenji  Yamamoto, Takahiro  Sakai, Shuji | 2022 | Noncontact measurement of puncture needle angle using augmented reality technology in computed tomography-guided biopsy: stereotactic coordinate design and accuracy evaluation | Title-abstract exclusion |
| 1134 | Suzuki, Kazufumi  Sakai, Shuji | 2023 | Agreement Between Augmented Reality and Computed Tomography Coordinate Systems: A New Approach to an Image-Guided Procedure | Title-abstract exclusion |
| 1135 | Swanson, K. R.  Rostomily, R. C.  Alvord Jr, E. C. | 2008 | A mathematical modelling tool for predicting survival of individual patients following resection of glioblastoma: A proof of principle | Title-abstract exclusion |
| 1136 | Świątek-Najwer, E.  Majak, M.  Żuk, M.  Popek, M.  Kulas, Z.  Jaworowski, J.  Pietruski, P. | 2017 | The new computer and fluorescence-guided system for planning and aiding oncological treatment | Title-abstract exclusion |
| 1137 | Tadrous, P. J.  Podoleanu, A. G.  Dobre, G.  Stamp, G. W. H. | 2003 | Application of VRML for 3-dimensional, interactive, real-time comparison of OCT structure with standard histology | Title-abstract exclusion |
| 1138 | Tai, Yonghang  Qian, Kai  Huang, Xiaoqiao  Zhang, Jun  Jan, Mian Ahmad  Yu, Zhengtao | 2021 | Intelligent Intraoperative Haptic-AR Navigation for COVID-19 Lung Biopsy Using Deep Hybrid Model | Title-abstract exclusion |
| 1139 | Tai, Y.  Shi, J.  Wei, L.  Huang, X.  Chen, Z.  Li, Q. | 2018 | Real-Time Visuo-Haptic Surgical Simulator for Medical Education – A Review | Title-abstract exclusion |
| 1140 | Tai, Yonghang  Wei, Lei  Xiao, Minhui  Zhou, Hailing  Li, Qiong  Shi, Junsheng  Nahavandi, Saeid | 2018 | A High-Immersive Medical Training Platform Using Direct lntraoperative Data | Title-abstract exclusion |
| 1141 | Tai, Y.  Wei, L.  Zhou, H.  Nahavandi, S.  Shi, J.  Li, Q. | 2017 | Integrating virtual reality and haptics for renal puncture surgical simulator | Title-abstract exclusion |
| 1142 | Tailor, V. K.  Abou-Rayyah, Y.  Brookes, J.  Khaw, P. T.  Papadopoulos, M.  Adams, G. G. W.  Bunce, C.  Dahlmann-Noor, A. | 2017 | Quality of life and functional vision in children treated for cataract-a cross-sectional study | Title-abstract exclusion |
| 1143 | Takano, T.  Asahi, S.  Matsuzuka, F.  Hidaka, Y.  Yoshida, H.  Miyauchi, A. | 2008 | Aspiration biopsy-nucleic acid diagnosis of thyroid malignant lymphoma by vectorette PCR: Experience of eight cases | Title-abstract exclusion |
| 1144 | Tan, H. N.  Yang, B. L.  Wang, S. P.  Peng, W. J.  Wu, J.  Gu, Y. J.  Wu, J.  Qian, M.  Hu, X. X. | 2010 | The feasibility of breast cancer sentinel lymph node mapping at CT lymphography | Title-abstract exclusion |
| 1145 | Tan, L.  Li, Y.  Hu, X.  Lu, M.  Zhang, Y.  Gan, Y.  Tu, C.  Min, L. | 2023 | Clinical evaluation of the three-dimensional printed strut-type prosthesis combined with autograft reconstruction for giant cell tumor of the distal femur | Title-abstract exclusion |
| 1146 | Tan, W.  Wang, J.  Huang, P.  Yang, G.  Zhou, Q. | 2023 | Multi-Needle Particle Implantation Computer Assisted Surgery Based on Virtual Reality | Title-abstract exclusion |
| 1147 | Tandon, S.  Devarajan, V.  Richer, E. | 2008 | Design and simulation of a visual and haptic assisted biopsy (ViHAB) system | Title-abstract exclusion |
| 1148 | Tang, Y.  Li, W.  Chu, Y.  Han, Q. | 2023 | Self-rescue technique and Yamane's technique in situ: Management of intraoperative haptic slippage | Title-abstract exclusion |
| 1149 | Tang, Z. N.  Hu, L. H.  Soh, H. Y.  Yu, Y.  Zhang, W. B.  Peng, X. | 2022 | Accuracy of Mixed Reality Combined With Surgical Navigation Assisted Oral and Maxillofacial Tumor Resection | Title-abstract exclusion |
| 1150 | Tangkijvanich, Pisit  Komolmit, Piyawat  Mahachai, Varocha  Sa-nguanmoo, Pattaratida  Theamboonlers, Apiradee  Poovorawan, Yong | 2010 | Comparison between quantitative hepatitis B surface antigen, hepatitis B e-antigen and hepatitis B virus DNA levels for predicting virological response to pegylated interferon-α-2b therapy in hepatitis B e-antigen-positive chronic hepatitis B | Title-abstract exclusion |
| 1151 | Tatanis, V.  Cracco, C. M.  Liatsikos, E. | 2023 | Advances in percutaneous renal puncture: A comprehensive review of the literature | Title-abstract exclusion |
| 1152 | Tawfik, Ahmad M.  El-Abd, Ahmed S.  El-Enen, Mohamed Abo  Farahat, Yasser A.  El-Bendary, Mohamed A.  El-Gamal, Osama M.  Soliman, Mohamed G.  El-Bahnasy, Abdelhameed M.  Rasheed, Mohamed | 2017 | Validity of a sponge trainer as a simple training model for percutaneous renal access | Title-abstract exclusion |
| 1153 | Teama, A. H.  Darweesh, A. E. M. N.  Abol-Enin, H. A.  Abouelkheir, R. T. | 2014 | Role of multidetector computed tomography virtual cystoscopy in evaluation of urinary bladder carcinoma | Title-abstract exclusion |
| 1154 | Teber, D.  Müller, M.  Fangerau, M.  Meinzer, P.  Klein, J.  Simpfendörfer, T.  Hohenfellner, M.  Rassweiler, J. | 2012 | iPad-assisted percutaneous access to the kidney: Initial experience | Title-abstract exclusion |
| 1155 | Tedesco-Silva, H.  Peddi, V. R.  Sanchez-Fructuoso, A.  Marder, B. A.  Russ, G. R.  Diekmann, F.  Flynn, A.  Hahn, C. M.  Li, H.  Tortorici, M. A.  et al., | 2016 | Open-Label, Randomized Study of Transition From Tacrolimus to Sirolimus Immunosuppression in Renal Allograft Recipients | Title-abstract exclusion |
| 1156 | Teh, J. J.  Pascoe, D. J.  Hafeji, S.  Parchure, R.  Koczoski, A.  Rimmer, M. P.  Khan, K. S.  Al Wattar, B. H. | 2024 | Efficacy of virtual reality for pain relief in medical procedures: a systematic review and meta-analysis | Title-abstract exclusion |
| 1157 | Teles, A. R.  Mattei, T. A. | 2017 | Robotic technology in neurosurgery: Past, present, and future perspectives | Title-abstract exclusion |
| 1158 | tenBerge, J.  Hoffman, B. J.  Hawes, R. H.  van Enckevort, C.  Giovannini, M.  Erickson, R. A.  et al | 2002 | EUS-guided fine needle aspiration of the liver: indications, yield, and safety based on an international survey of 167 cases | Title-abstract exclusion |
| 1159 | Tenzer, Yaroslav  Davies, Brian  Rodriguez y Baena, Ferdinando | 2008 | Investigation into the effectiveness of vibrotactile feedback to improve the haptic realism of an arthroscopy training simulator | Title-abstract exclusion |
| 1160 | Thali, M. J.  Yen, K.  Vock, P.  Ozdoba, C.  Kneubuehl, B. P.  Sonnenschein, M.  Dirnhofer, R. | 2003 | Image-guided virtual autopsy findings of gunshot victims performed with multi-slice computed tomography (MSCT) and magnetic resonance imaging (MRI) and subsequent correlation between radiology and autopsy findings | Title-abstract exclusion |
| 1161 | Thanh Trung, Tran  Van Khoa, Dang  Van Dong, Trinh | 2021 | The comparison of analgesic efficacy between ultrasound-guided continuous thoracic paravertebral block and continuous thoracic epidural block using bupivacaine - fentanyl in patients undergoing lung surgery: A prospective, randomized, controlled trial | Title-abstract exclusion |
| 1162 | Theivendrampillai, Shenthiuiyan  Yang, Bob  Little, Mark  Blick, Christopher | 2024 | Targeted augmented reality-guided transperineal prostate biopsies study: initial experience | Title-abstract exclusion |
| 1163 | Thieman Mankin, K. M.  Cornell, K.  Peycke, L.  Dickerson, V.  Scallan, E. | 2021 | Adaptation of a hands-on veterinary surgical training course from a traditionally taught laboratory to a remotely taught laboratory during a global pandemic | Title-abstract exclusion |
| 1164 | Thoeni, R. F | 1997 | Colorectal cancer. Radiologic staging | Title-abstract exclusion |
| 1165 | Thomas, James J.  Albietz, Joseph  Polaner, David | 2018 | Virtual reality for lumbar puncture in a morbidly obese patient with leukemia | Case report |
| 1166 | Thomas, M. Bijoy  Mariani, Andrea  Cliby, William A.  Keeney, Gary A.  Podratz, Karl C.  Dowdy, Sean C. | 2007 | Role of systematic lymphadenectomy and adjuvant therapy in stage I uterine papillary serous carcinoma | Title-abstract exclusion |
| 1167 | Thompson, G. H. | 2024 | CORR Insights®: Virtual Reality Distraction Is No Better Than Simple Distraction Techniques for Reducing Pain and Anxiety During Pediatric Orthopaedic Outpatient Procedures: A Randomized Controlled Trial | Title-abstract exclusion |
| 1168 | Thu Hang Tran, Thi  Mudiyanselage, Sriyani Padmalatha Konara  Huang, Mei-Chih | 2022 | Effects of Distraction on Reducing Pain During Invasive Procedures in Children with Cancer: A Systematic Review and Meta-Analysis | Title-abstract exclusion |
| 1169 | Tiessi Gomes de Oliveira, Ana Claudia Melo  dos Santos Nunes, Fatima de Lourdes | 2010 | Tiessi Gomes de Oliveira, Ana Claudia Melo  dos Santos Nunes, Fatima de Lourdes | Title-abstract exclusion |
| 1170 | Tirakotai, W.  Bozinov, O.  Sure, U.  Riegel, T.  Bertalanffy, H.  Hellwig, D. | 2004 | The evolution of stereotactic guidance in neuroendoscopy | Title-abstract exclusion |
| 1171 | Tjong, Fleur V. Y.  Perrotta, Laura  Goette, Andreas  Duncker, David  Vernooy, Kevin  Boveda, Serge  Chun, Kyoung-Ryul Julian  Svennberg, Emma | 2024 | Utilization of and perceived need for simulators in clinical electrophysiology: results from an EHRA physician survey | Title-abstract exclusion |
| 1172 | Toledo del Castillo, B.  Pérez Torres, J. A.  Morente Sánchez, L.  Escobar Castellanos, M.  Escobar Fernández, L.  González Sánchez, M. I.  Rodríguez Fernández, R. | 2019 | Reducing the pain in invasive procedures during paediatric hospital admissions: Fiction, reality or virtual reality? | Title-abstract exclusion |
| 1173 | Tomas, C.  Soyer, P.  Dohan, A.  Dray, X.  Boudiaf, M.  Hoeffel, C. | 2014 | Update on imaging of Peutz-Jeghers syndrome | Title-abstract exclusion |
| 1174 | Tomikawa, M.  Hong, J.  Akahoshi, T.  Tsutsumi, N.  Ohuchida, K.  Ieiri, S.  Ohdaira, T.  Hashizume, M. | 2011 | Usefulness of a real-time virtual reality navigation system using an open magnetic resonance imaging: Tumor ablation therapy for 50 liver cancers | Title-abstract exclusion |
| 1175 | Tomikawa, Morimasa  Hong, Jaesung  Shiotani, Satoko  Tokunaga, Eriko  Konishi, Kozo  Ieiri, Satoshi  Tanoue, Kazuo  Akahoshi, Tomohiko  Maehara, Yoshihiko  Hashizume, Makoto | 2010 | Real-Time 3-Dimensional Virtual Reality Navigation System with Open MRI for Breast-Conserving Surgery | Title-abstract exclusion |
| 1176 | Tong, J.  Chu, C.  Li, B. | 2023 | Vascular interventional surgery robot and its force feedback technology | Title-abstract exclusion |
| 1177 | Toraman, Rumeysa Lale  Eskici Ilgin, Vesile | 20 | Effect of Virtual Reality Glasses Application on Pain, Anxiety, and Patient Satisfaction During a Transrectal Prostate Biopsy: A Randomized Controlled Trial | Title-abstract exclusion |
| 1178 | Torres, R. S.  Nunes, F. L. S. | 2011 | Applying entertaining aspects of serious game in medical training: Systematic review and implementation | Title-abstract exclusion |
| 1179 | Tran Thi, T. H.  Konara Mudiyanselage, S. P.  Huang, M. C. | 2022 | Effects of Distraction on Reducing Pain During Invasive Procedures in Children with Cancer: A Systematic Review and Meta-Analysis | Title-abstract exclusion |
| 1180 | Treanor, D.  Lim, C. H.  Magee, D.  Bulpitt, A.  Quirke, P. | 2009 | Tracking with virtual slides: A tool to study diagnostic error in histopathology | Title-abstract exclusion |
| 1181 | Trivedi, H. L.  Vanikar, A. V.  Modi, P. R.  Shah, V. R.  Vakil, J. M.  Trivedi, V. B.  Khemchandani, S. I. | 2005 | Allogeneic hematopoietic stem-cell transplantation, mixed chimerism, and tolerance in living related donor renal allograft recipients | Title-abstract exclusion |
| 1182 | Trong, Nguyen  Plishker, William  Matisoff, Andrew  Sharma, Karun  Shekhar, Raj | 2022 | HoloUS: Augmented reality visualization of live ultrasound images using HoloLens for ultrasound-guided procedures | Title-abstract exclusion |
| 1183 | Troosters, T.  Casaburi, R. | 2020 | Interview with prof. Dr richard casaburi, presidential awardee 2020 | Title-abstract exclusion |
| 1184 | Trottier, E. D.  Osmanlliu, E.  Bailey, B.  Lagace, M.  Sanchez, M.  Certain, M.  Khadra, C.  Theriault, C.  Paquin, D.  Turpin, C. C.  et al., | 2020 | Distraction in the ed using virtual reality for intravenous needs in children to improve comfort-the devinci project: a pilot RCT | Title-abstract exclusion |
| 1185 | Trung, T. T.  Van Khoa, D.  Van Dong, T. | 2021 | The comparison of analgesic efficacy between ultrasound-guided continuous thoracic paravertebral block and continuous thoracic epidural block using bupivacaine - fentanyl in patients undergoing lung surgery: A prospective, randomized, controlled trial | Title-abstract exclusion |
| 1186 | Tsai, C. K.  Wang, T. D.  Lin, J. W.  Hsu, R. B.  Guo, L. Z.  Chen, S. T.  Liu, T. M. | 2013 | Virtual optical biopsy of human adipocytes with third harmonic generation microscopy | Title-abstract exclusion |
| 1187 | Tsai, M. R.  Chen, S. Y.  Shieh, D. B.  Lou, P. J.  Sun, C. K. | 2011 | In vivo optical virtual biopsy of human oral mucosa with harmonic generation microscopy | Title-abstract exclusion |
| 1188 | Tseng, C. S.  Chung, C. W.  Chen, H. H.  Wang, S. S.  Tseng, H. M. | 1999 | Development of a robotic navigation system for neurosurgery | Title-abstract exclusion |
| 1189 | Tsunoda, S.  Itoh, M.  Oshima, H. | 1994 | An evaluation of rectal mucosal hemoglobin index by electric endoscopic image processing —A comparative study with the histpathological findings— | Title-abstract exclusion |
| 1190 | Tsurumaru, D.  Takatsu, N.  Kai, S.  Oki, E.  Ishigami, K. | 2021 | Measurement of circumferential tumor extent of colorectal cancer on CT colonography: relation to clinicopathological features and patient prognosis after surgery | Title-abstract exclusion |
| 1191 | Tulman, David | 2018 | Quantitative Evaluation and Optimization of Video-Rate Structured Illumination Microscopy (VR-SIM) for Clinical Applications in Point-of-Procedure Tissue Assessment | Title-abstract exclusion |
| 1192 | Tytgat, G. N. J. | 2008 | Best Practice and Research: Clinical Gastroenterology | Title-abstract exclusion |
| 1193 | Ueda, K.  McCague, K. M.  Wiland, A.  Peddi, V. R. | 2014 | Early corticosteroid withdrawal in the real world: a long-term analysis of kidney transplant recipients from the mycophenolic acid observational renal transplant registry | Title-abstract exclusion |
| 1194 | Uhl, C.  Hatzl, J.  Meisenbacher, K.  Zimmer, L.  Hartmann, N.  Böckler, D. | 2022 | Mixed-Reality-Assisted Puncture of the Common Femoral Artery in a Phantom Model | Title-abstract exclusion |
| 1195 | Uhl, J. F. | 2012 | Three-dimensional modelling of the venous system by direct multislice helical computed tomography venography: Technique, indications and results | Title-abstract exclusion |
| 1196 | Uman, Lindsay S.  Birnie, Kathryn A.  Noel, Melanie  Parker, Jennifer A.  Chambers, Christine T.  McGrath, Patrick J.  Kisely, Steve R. | 2013 | Psychological interventions for needle-related procedural pain and distress in children and adolescents | Title-abstract exclusion |
| 1197 | Uno, L. H.  Sugimoto, O.  Carvalho, F. M.  Bagnoli, V. R.  Fonseca, A. M.  Pinotti, J. A. | 1995 | Morphologic hysteroscopic criteria suggestive of endometrial hyperplasia | Title-abstract exclusion |
| 1198 | Unroe, Mark A.  Shofer, Scott L.  Wahidi, Momen M. | 2010 | Training for endobronchial ultrasound: methods for proper training in new bronchoscopic techniques | Title-abstract exclusion |
| 1199 | Urban, M. W.  Greenleaf, J. F.  Mitri, F. G. | 2010 | Measurement of prostate viscoelasticity using Shearwave Dispersion Ultrasound Vibrometry (SDUV): An in vitro study | Title-abstract exclusion |
| 1200 | Ushida, T.  Ikemoto, T.  Tanaka, S.  Shinozaki, J.  Taniguchi, S.  Murata, Y.  McLaughlin, M.  Arai, Y. C. P.  Tamura, Y. | 2008 | Virtual needle pain stimuli activates cortical representation of emotions in normal volunteers | Title-abstract exclusion |
| 1201 | Vahora, F.  Ternkin, B.  Marcy, W.  Gorman, P. J.  Krummel, T. M.  Heinrichs, W. L. | 20 | Virtual reality and women's health: A breast biopsy system | Title-abstract exclusion |
| 1202 | Valdivia Y Alvarado, M.  Cheng He, T.  Xue, Z.  Wong, S.  Wong, K. | 2010 | Peripheral lung cancer detection by vascular tumor labeling using in-vivo microendoscopy under real time 3D CT image guided intervention | Title-abstract exclusion |
| 1203 | Valentine, R. J.  Rege, R. V. | 2004 | Integrating technical competency into the surgical curriculum: Doing more with less | Title-abstract exclusion |
| 1204 | Valentini, Ilaria  Lazzari Agli, Luigi  Michieletto, Lucio  Innocenti, Margherita  Savoia, Francesca  Del Prato, Bruno  Mancino, Laura  Maddau, Cristina  Romano, Annamaria  Puorto, Antonella  Corbetta, Lorenzo  Fois, Alessandro | 2019 | Competence in flexible bronchoscopy and basic biopsy technique | Title-abstract exclusion |
| 1205 | Van Den Berg, N. S.  Brouwer, O. R.  KleinJan, G. H.  Van Der Poel, H. G.  Wendler, T.  Valdés Olmos, R. A.  Van Leeuwen, F. | 2013 | Virtual-reality-based navigation during the robot-assisted laparoscopic sentinel node biopsy procedure in prostate cancer patients | Title-abstract exclusion |
| 1206 | Van Den Berg, N. S.  Brouwer, O. R.  Mathéron, H. M.  KleinJan, G. H.  Wendler, T.  Nieweg, O. E.  Horenblas, S.  Van Der Poel, H. G.  Valdés Olmos, R. A.  Van Leeuwen, F. | 2013 | Navigation towards the sentinel node in the groin | Title-abstract exclusion |
| 1207 | Van Den Berg, N. S.  Engelen, T.  Brouwer, O. R.  Mathéron, H. M.  Valdés-Olmos, R. A.  Nieweg, O. E.  Van Leeuwen, F. W. B. | 2016 | A pilot study of SPECT/CT-based mixed-reality navigation towards the sentinel node in patients with melanoma or Merkel cell carcinoma of a lower extremity | Title-abstract exclusion |
| 1208 | Van Nguyen, D.  Ben Lakhal, S.  Chellali, A. | 2015 | Preliminary evaluation of a virtual needle insertion training system | Title-abstract exclusion |
| 1209 | van Oosterom, Matthias Nathanal  Engelen, Myrthe Adriana  van den Berg, Nynke Sjoerdtje  KleinJan, Gijs Hendrik  van der Poel, Henk Gerrit  Wendler, Thomas  van de Velde, Cornelis Jan Hadde  Navab, Nassir  van Leeuwen, Fijs Willem Bernhard | 2016 | Navigation of a robot-integrated fluorescence laparoscope in preoperative SPECT/CT and intraoperative freehand SPECT imaging data: a phantom study | Title-abstract exclusion |
| 1210 | van Oosterom, M. N.  Meershoek, P.  KleinJan, G. H.  Hendricksen, K.  Navab, N.  van de Velde, C. J. H.  van der Poel, H. G.  van Leeuwen, F. W. B. | 2018 | Navigation of Fluorescence Cameras during Soft Tissue Surgery—Is it Possible to Use a Single Navigation Setup for Various Open and Laparoscopic Urological Surgery Applications? | Title-abstract exclusion |
| 1211 | van Oosterom, Matthias N.  Meershoek, Philippa  Welling, Mick M.  Pinto, Francisco  Matthies, Philipp  Simon, Herve  Wendler, Thomas  Navab, Nassir  van de Velde, Cornelis J. H.  van der Poel, Henk G.  van Leeuwen, Fijs W. B. | 2020 | Extending the Hybrid Surgical Guidance Concept With Freehand Fluorescence Tomography | Title-abstract exclusion |
| 1212 | Van Zundert, A. | 2013 | Epidural simulators | Title-abstract exclusion |
| 1213 | Vanoli, Sylvain  Grobet-Jeandin, Elisabeth  Windisch, Olivier  Valerio, Massimo  Benamran, Daniel | 2024 | Evolution of anxiety management in prostate biopsy under local anesthesia: a narrative review | Title-abstract exclusion |
| 1214 | Vaughan, Neil  Dubey, Venketesh N.  Wee, Michael Y. K.  Isaacs, Richard | 2013 | A review of epidural simulators: Where are we today? | Title-abstract exclusion |
| 1215 | Vaughan, N.  Dubey, V. N.  Wee, M. Y. K.  Isaacs, R. | 2014 | Development Of Epidural Simulators: Towards Hybrid Virtual Reality Training | Title-abstract exclusion |
| 1216 | Vavra, P.  Roman, J.  Zonca, P.  Ihnat, P.  Nemec, M.  Kumar, J.  Habib, N.  El-Gendi, A. | 2017 | Recent Development of Augmented Reality in Surgery: A Review | Title-abstract exclusion |
| 1217 | Vázques, J. L. M.  Golsong, J. B.  Wiederhold, M. D. | 2013 | Cybertherapy in medicine - Clinical applications to reduce pain and anxiety | Title-abstract exclusion |
| 1218 | Velasco-Hidalgo, Liliana  Gonzalez-Garay, Alejandro  Segura-Pacheco, Blanca Angelica  Esparza-Silva, Ana Luisa  Mendoza, Miguel Enrique Cuellar  Ochoa-Drucker, Cecilia  Campos-Ugalde, Sofia  Bernabe-Gaspar, Luis Eduardo  Zapata-Tarres, Marta | 2024 | Virtual reality as a non-medical tool in the treatment of anxiety, pain, and perception of time in children in the maintenance phase of acute lymphoblastic leukemia treatment | Title-abstract exclusion |
| 1219 | Velazco-Garcia, J. D.  Leiss, E. L.  Karkoub, M.  Tsiamyrtzis, P.  Tsekos, N. V.  Navkar, N. V.  et al | 2019 | Preliminary evaluation of robotic transrectal biopsy system on an interventional planning software | Title-abstract exclusion |
| 1220 | Veneziano, D.  Smith, A.  Reihsen, T.  Hananel, D.  Stubbs, J.  Speich, J.  Sweet, R. | 2015 | The simportal fluoro-less C-arm trainer (CAT): An innovative training device for percutaneous kidney access | Title-abstract exclusion |
| 1221 | Venkatesh, R. D.  Leinwand, K.  Nguyen, N. | 2023 | Pediatric Unsedated Transnasal Endoscopy | Title-abstract exclusion |
| 1222 | Vermorken, J. B.  Stöhlmacher-Williams, J.  Davidenko, I.  Licitra, L.  Winquist, E.  Villanueva, C.  Foa, P.  Rottey, S.  Skladowski, K.  Tahara, M.  et al., | 2013 | Cisplatin and fluorouracil with or without panitumumab in patients with recurrent or metastatic squamous-cell carcinoma of the head and neck (SPECTRUM): an open-label phase 3 randomised trial | Title-abstract exclusion |
| 1223 | Vicandi, B.  Jiménez-Heffernan, J. A.  López-Ferrer, P.  Patrón, M.  Gamallo, C.  Colmenero, C.  Viguer, J. M. | 1999 | HIV-1 (p24)-positive multinucleated giant cells in HIV-associated lymphoepithelial lesion of the parotid gland -: A report of two cases | Title-abstract exclusion |
| 1224 | Vidal, F. P.  John, N. W.  Guillemot, R. M. | 2007 | Interactive physically-based x-ray simulation: CPU or GPU? | Title-abstract exclusion |
| 1225 | Vidal, F. P.  John, N. W.  Healey, A. E.  Gould, D. A. | 2008 | Simulation of ultrasound guided needle puncture using patient specific data with 3D textures and volume haptics | Title-abstract exclusion |
| 1226 | Vidal, F. P.  Villard, P. F.  Holbrey, R.  John, N. W.  Bello, F.  Bulpitt, A.  Gould, D. A. | 2009 | Developing an immersive ultrasound guided needle puncture simulator | Title-abstract exclusion |
| 1227 | Vidal-Sicart, Sergi  Goni, Elena  Cebrecos, Isaac  Rioja, Ma Eugenia  Perissinotti, Andres  Sampol, Catalina  Vidal, Oscar  et al | 2024 | Continuous innovation in precision radio-guided surgery | Title-abstract exclusion |
| 1228 | Vijayakumar, Mohankumar  Balaji, Sudharsan  Singh, Abhishek  Ganpule, Arvind  Sabnis, Ravindra  Desai, Mahesh | 2019 | A novel biological model for training in percutaneous renal access | Title-abstract exclusion |
| 1229 | Vikal, S.  U-Thainual, P.  Carrino, J. A.  Iordachita, I.  Fischer, G. S.  Fichtinger, G. | 2010 | Perk Station-Percutaneous surgery training and performance measurement platform | Title-abstract exclusion |
| 1230 | Villard, P. F.  Jacob, M.  Gould, D.  Bello, F. | 2009 | Haptic simulation of the liver with respiratory motion | Title-abstract exclusion |
| 1231 | Villard, P. F.  Vidal, F. P.  Ap Cenydd, L.  Holbrey, R.  Pisharody, S.  Johnson, S.  Bulpitt, A.  John, N. W.  Bello, F.  Gould, D. | 2014 | Interventional radiology virtual simulator for liver biopsy | Title-abstract exclusion |
| 1232 | Villard, P. F.  Vidal, F. P.  Bello, F.  John, N. W. | 2012 | A method to compute respiration parameters for patient-based simulators | Title-abstract exclusion |
| 1233 | Villard, P. F.  Vidal, F. P.  Hunt, C.  Bello, F.  John, N. W.  Johnson, S.  Gould, D. A. | 2009 | A prototype percutaneous transhepatic cholangiography training simulator with real-time breathing motion | Title-abstract exclusion |
| 1234 | Villard, Pierre-Frederic  Jacob, Mathieu  Gould, Derek  Bello, Fernando | 2009 | Haptic simulation of the liver with respiratory motion | Title-abstract exclusion |
| 1235 | Villar-López, M. B.  Gómez-Cambronero, Á  Suarez, D.  Remolar, I. | 2023 | Development of an Immersive Virtual Reality System to Practice the Lumbar Puncture Manoeuvre | Title-abstract exclusion |
| 1236 | Villers, A.  Marliere, F.  Ouzzane, A.  Puech, P.  Lemaître, L. | 2012 | MRI in addition to or as a substitute for prostate biopsy: The clinician's point of view | Title-abstract exclusion |
| 1237 | Vincenti, F.  Shaffer, D.  Qazi, Y.  Shihab, F.  McCague, K.  Patel, D.  Peddi, V. R.  Yilmaz, S. | 2016 | The effect of everolimus and tacrolimus exposure levels on renal histology parameters 6 months post-transplantation | Title-abstract exclusion |
| 1238 | Voelker, W.  Maier, S.  Lengenfelder, B.  Schoebel, W.  Petersen, J.  Bonz, A.  Ertl, G. | 2011 | Improved quality of coronary diagnostics and interventions by virtual reality simulation | Title-abstract exclusion |
| 1239 | Voi, E. L.  Basile, G. C.  Bramanti, A.  Paladina, G.  Militi, A.  Bruschetta, D.  Alito, A.  Cavallaro, F.  Bertino, S.  Milardi, D. | 2021 | Cerebellar Atrophy Associated with Primary Sjögren’s Syndrome: Diagnosis, Therapy, and Virtual Reality Rehabilitation: A Case Report | Title-abstract exclusion |
| 1240 | Volpi, G.  Checcucci, E.  Amparore, D.  De Cillis, S.  Piramide, F.  Piana, A.  Sica, M.  et al | 2023 | The application of artificial intelligence guided 3D automatic augmented-reality biopsy allows to improve the oncological safety of the nerve sparing phase during robotic prostatectomy | Title-abstract exclusion |
| 1241 | von Haxthausen, Felix  Rueger, Christoph  Sieren, Malte Maria  Kloeckner, Roman  Ernst, Floris | 2023 | Augmenting Image-Guided Procedures through In Situ Visualization of 3D Ultrasound via a Head-Mounted Display | Title-abstract exclusion |
| 1242 | von Niederhaeusern, Peter A.  Seppi, Carlo  Sandkuehler, Robin  Nicolas, Guillaume  Haerle, Stephan K.  Cattin, Philippe C. | 2024 | International Journal of Computer Assisted Radiology and Surgery | Title-abstract exclusion |
| 1243 | Vosburgh, K. G.  San José Estépar, R. | 2007 | Natural orifice transluminal endoscopic surgery (NOTES): An opportunity for augmented reality guidance | Title-abstract exclusion |
| 1244 | Vosburgh, K. G.  Schenck, J. F. | 1998 | Experience with MR-guided therapy | Title-abstract exclusion |
| 1245 | Vrillon, Agathe  Gonzales-Marabal, Laurent  Ceccaldi, Pierre-Francois  Plaisance, Patrick  Desrentes, Eric  Paquet, Claire  Dumurgier, Julien | 2022 | Using virtual reality in lumbar puncture training improves students learning experience | Title-abstract exclusion |
| 1246 | Wacker, F. K.  Vogt, S.  Khamene, A.  Jesberger, J. A.  Nour, S. G.  Elgort, D. R.  Sauer, F.  Duerk, J. L.  Lewin, J. S. | 2006 | An augmented reality system for MR image-guided needle biopsy: Initial results in a swine model | Title-abstract exclusion |
| 1247 | Wagner, A.  Undt, G.  Watzinger, F.  Wanschitz, F.  Schicho, K.  Yerit, K.  Kermer, C.  Birkfellner, W.  Ewers, R. | 2001 | Principles of computer-assisted arthroscopy of the temporomandibular joint with optoelectronic tracking technology | Title-abstract exclusion |
| 1248 | Wahab, Ahsan  Kesari, Kavitha  Smith, Susan J.  Liu, Yang  Barta, Stefan K. | 2018 | Type B lactic acidosis, an uncommon paraneoplastic syndrome | Title-abstract exclusion |
| 1249 | Wake, Nicole  Rosenkrantz, Andrew B.  Sodickson, Daniel K.  Chandarana, Hersh  Wysock, James S. | 2020 | MRI guided procedure planning and 3D simulation for partial gland cryoablation of the prostate: a pilot study | Title-abstract exclusion |
| 1250 | Waldner, Douglas James | 2006 | Porcine reproductive and respiratory syndrome virus: Acute and persistent infections and genetic divergence of the virus during persistent infections | Title-abstract exclusion |
| 1251 | Wallace, M. B.  Keisslich, R. | 2010 | Advances in Endoscopic Imaging of Colorectal Neoplasia | Title-abstract exclusion |
| 1252 | Wan, M. | 2001 | Interactive electronic biopsy for 3D virtual colonoscopy | Title-abstract exclusion |
| 1253 | Wang, E. E.  Quinones, J.  Fitch, M. T.  Dooley-Hash, S.  Griswold-Theodorson, S.  Medzon, R.  Korley, F.  Laack, T.  Robinett, A.  Clay, L. | 2008 | Developing technical expertise in emergency medicine - The role of simulation in procedural skill acquisition | Title-abstract exclusion |
| 1254 | Wang, G.  Mercier, L.  Collins, D. L.  Cooperstock, J. R. | 2009 | A comparative study of monoscopic and stereoscopic display for a Probe-Positioning task | Title-abstract exclusion |
| 1255 | Wang, Jiayun  Walter, Peter  Baumgarten, Sabine | 2022 | Surgical anatomy of the small animal eye-3D reconstruction and ablation effect | Title-abstract exclusion |
| 1256 | Wang, Lifeng  Zhang, Yongde  Zuo, Sihao  Xu, Yong | 2021 | A review of the research progress of interventional medical equipment and methods for prostate cancer | Title-abstract exclusion |
| 1257 | Wang, Lei  Zhao, Zichen  Wang, Gang  Zhou, Jianfang  Zhu, He  et al | 2022 | Application of a three-dimensional visualization model in intraoperative guidance of percutaneous nephrolithotomy | Title-abstract exclusion |
| 1258 | Wang, Mei | 2017 | Video Rate Structured Illumination Microscopy for Rapid On-Site Pathology Evaluation | Title-abstract exclusion |
| 1259 | Wang, Mei  Kimbrell, Hillary Z.  Sholl, Andrew B.  Tulman, David B.  Elfer, Katherine N.  Schlichenmeyer, Tyler C.  Lee, Benjamin R.  Lacey, Michelle  Brown, J. Quincy | 2015 | High-Resolution Rapid Diagnostic Imaging of Whole Prostate Biopsies Using Video-Rate Fluorescence Structured Illumination Microscopy | Title-abstract exclusion |
| 1260 | Wang, M.  Sholl, A. B.  Kimbrell, H.  Tulman, D. B.  Elfer, K. N.  Brown, J. Q. | 2015 | Rapid diagnostic imaging and pathologic evaluation of whole core biopsies at the point-of-care using structured illumination microscopy | Title-abstract exclusion |
| 1261 | Wang, M.  Tulman, D.  Schlichenmeyer, T. C.  Kimbrell, H.  Brown, J. Q. | 2014 | Rapid diagnosis of whole prostate core-needle biopsies with video-rate structured illumination microscopy | Title-abstract exclusion |
| 1262 | Wang, Mei  Tulman, David B.  Sholl, Andrew B.  Kimbrell, Hillary Z.  Mandava, Sree H.  et al | 2016 | Gigapixel surface imaging of radical prostatectomy specimens for comprehensive detection of cancer-positive surgical margins using structured illumination microscopy | Title-abstract exclusion |
| 1263 | Wang, M.  Tulman, D. B.  Sholl, A. B.  Mandava, S. H.  Maddox, M. M.  Lee, B. R.  Quincy Brown, J. | 2018 | Partial nephrectomy margin imaging using structured illumination microscopy | Title-abstract exclusion |
| 1264 | Wang, Qi  Wang, Qiuyuan  Ding, Ran  Yao, Youjie  Pan, Junjun  Wang, Weiguo | 2022 | Augmented Reality Navigation-Guided Core Decompression for Osteonecrosis of Femoral Head | Title-abstract exclusion |
| 1265 | Wang, R.  Yao, J.  Wang, L.  Liu, X.  Wang, H.  Zheng, L. | 2017 | A surgical training system for four medical punctures based on virtual reality and haptic feedback | Title-abstract exclusion |
| 1266 | Wang, Shu  Frisbie, James  Keepers, Zachery  Bolten, Zachary  Hevaganinge, Anjana  Boctor, Emad  Leonard, Simon  Tokuda, Junichi  Krieger, Axel  Siddiqui, Mohummad Minhaj | 2021 | The Use of Three-dimensional Visualization Techniques for Prostate Procedures: A Systematic Review | Title-abstract exclusion |
| 1267 | Wang, Y.  Ding, H.  Xuan, J.  Sesterhenn, I. A.  Moul, J. W.  Mun, S. K. | 1998 | 3D model supported virtual environment for prostate disease interpretation and biopsy design | Title-abstract exclusion |
| 1268 | Wang, Yi  Feng, Yujin  Yang, Xiaoyun  Zhang, Liyan  Zhang, Tongdi  Wang, Wengang | 2016 | Clinical Values of Studying Kidney Elasticity with Virtual Touch Quantification in Gestational Hypertension Patients | Title-abstract exclusion |
| 1269 | Wang, Y. Y.  Liu, H. P.  Hsiao, F. L.  Kumar, A. | 2019 | Augmented reality for temporomandibular joint arthrocentesis: a cadaver study | Title-abstract exclusion |
| 1270 | Wang, Z.  Shi, G.  Wang, X. | 2023 | INNOVATIVE RESEARCH ON APPLICATION OF MULTIMODAL IMAGE FUSION NAVIGATION ROBOT TECHNOLOGY IN TUMOR ABLATION | Title-abstract exclusion |
| 1271 | Wani, Nisar A.  Mir, Farooq  Bhat, Irshad M.  Gojwari, Tariq  Bhat, Salma | 2011 | Giant Cystic Virchow-Robin Spaces with Adjacent White Matter Signal Alteration | Title-abstract exclusion |
| 1272 | Warren, H.  Neves, J. B. N.  Rode, N.  Cullen, D.  Santiapillai, J.  Pavlou, M. P.  et al., | 2023 | NEphron Sparing Treatment (NEST) for small renal masses: a feasibility cohort-embedded randomised controlled trial | Title-abstract exclusion |
| 1273 | Watanabe, Takuya  Fujiwara, Michitaka  Kodera, Yasuhiro  Sakaguchi, Masamichi  Hidaka, Hiroki  Fujimoto, Hideo  Nakao, Akimasa | 2011 | Measurement of Inserting Motion of Bladeless Trocar at Real Surgery for Development of a Virtual Training System for Initial Trocar Placement in Laparoscopic Surgery | Title-abstract exclusion |
| 1274 | Watson, CatherineHannah | 2021 | Reusable Surgical Phantoms With Self-Healing Nanocomposite Hydrogel | Title-abstract exclusion |
| 1275 | Weegink, C. J.  Sentjens, R. E.  Beld, M. G.  Dijkgraaf, M. G. W.  Reesink, H. W. | 2003 | Chronic hepatitis C patients with a post-treatment virological relapse re-treated with an induction dose of 18 MU interferon-α in combination with ribavirin and amantadine: A two-arm randomized pilot study | Title-abstract exclusion |
| 1276 | Wegelin, O.  Henken, K. R.  Somford, D. M.  Breuking, F. A. M.  Bosch, R. J.  Van Swol, C. F. P.  Van Melick, H. H. E. | 2016 | An Ex Vivo Phantom Validation Study of an MRI-Transrectal Ultrasound Fusion Device for Targeted Prostate Biopsy | Title-abstract exclusion |
| 1277 | Wegenkittl, R.  Medgraph, T.  Vilanova, A.  Hegedüs, B.  Wagner, D.  Freund, M. C.  Gröller, E. M. | 2000 | Mastering interactive virtual bronchioscopy on a low-end PC | Title-abstract exclusion |
| 1278 | Wegner, K.  Kanon, D. B. | 1998 | Audio-guided blind biopsy needle placement | Title-abstract exclusion |
| 1279 | Wei, Qin  Sun, Rong  Liang, Yan  Chen, Dan | 2024 | Virtual reality technology reduces the pain and anxiety of children undergoing vein puncture: a meta-analysis | Title-abstract exclusion |
| 1280 | Weinfurtner, R. J.  Raghunand, N.  Stringfield, O.  Abdalah, M.  Niell, B. L.  Ataya, D.  et al | 2022 | MRI Response to Pre-operative Stereotactic Ablative Body Radiotherapy (SABR) in Early Stage ER/PR+ HER2- Breast Cancer correlates with Surgical Pathology Tumor Bed Cellularity | Title-abstract exclusion |
| 1281 | Weiss, Clifford R.  Nour, Sherif Gamal  Lewin, Jonathan S. | 2008 | MR-guided biopsy: A review of current techniques and applications | Title-abstract exclusion |
| 1282 | Wells, P. N. T. | 2000 | Advances in ultrasound: From microscanning to telerobotics | Title-abstract exclusion |
| 1283 | Wen, L.  Fan, Y. H.  Wu, C. W.  Wong, H. G.  et al | 2023 | The effect of a virtual reality device (VRD, HypnoVR) on pain, anxiety scores and satisfaction during transperineal targeted and systemic prostate biopsies | Meeting Abstract |
| 1284 | Wendt, M.  Sauer, F.  Khamene, A.  Bascle, B.  Vogt, S.  Wacker, F. K. | 2003 | A head-mounted display system for augmented reality: Initial evaluation for interventional MRI | Title-abstract exclusion |
| 1285 | Weng, W. H.  Liao, Y. H.  Tsai, M. R.  Wei, M. L.  Huang, H. Y.  Sun, C. K. | 2016 | Differentiating intratumoral melanocytes from Langerhans cells in nonmelanocytic pigmented skin tumors in vivo by label-free third-harmonic generation microscopy | Title-abstract exclusion |
| 1286 | Whittaker, G.  Aydin, A.  Raison, N.  Kum, F.  Challacombe, B.  Khan, M. S.  Dasgupta, P.  Ahmed, K. | 2016 | Validation of the RobotiX Mentor Robotic Surgery Simulator | Title-abstract exclusion |
| 1287 | Wiegand, C.  Bittenger, K.  Galiano, R. D.  Driver, V. R.  Gibbons, G. W. | 2017 | Does noncontact low-frequency ultrasound therapy contribute to wound healing at the molecular level? | Title-abstract exclusion |
| 1288 | Wilhelm, D.  Vogel, T.  Ostler, D.  Marahrens, N.  Kohn, N.  Koller, S.  Friess, H.  Kranzfelder, M. | 2018 | Enhanced Visualization: From Intraoperative Tissue Differentiation to Augmented Reality | Title-abstract exclusion |
| 1289 | Wilke, P. I.  Biermann, D.  Grafmann, M.  Kozlik-Feldmann, R.  Papingi, D.  Sachweh, J. S.  Stute, F.  Olfe, J. | 2023 | Siblings with Gorlin–Goltz syndrome associated with cardiac tumors: a case report and review of literature | Title-abstract exclusion |
| 1290 | Wilken, R.  Li, C. S.  Sharon, V. R.  Kim, K.  Patel, F. B.  Patel, F.  Maverakis, E. | 2015 | Topical clobetasol for the treatment of toxic epidermal necrolysis: study protocol for a randomized controlled trial | Title-abstract exclusion |
| 1291 | Wilms, M.  Fortmeier, D.  Mastmeyer, A.  Handels, H. | 2015 | Modellbasierte simulation der Atembewegung für das virtual-reality-training von Punktionseingriffen | Title-abstract exclusion |
| 1292 | Winetraub, Y.  Vleck, A. V.  Yuan, E.  Terem, I.  Zhao, J.  et al | 2024 | Noninvasive virtual biopsy using micro-registered optical coherence tomography (OCT) in human subjects | Title-abstract exclusion |
| 1293 | Witt, Benjamin L.  Adler, Douglas G.  Hilden, Kristen  Layfield, Lester J. | 2013 | A Comparative Needle Study: EUS-FNA Procedures Using the HD ProCore™ and EchoTip® 22-Gauge Needle Types | Title-abstract exclusion |
| 1294 | Wong, C. L.  Choi, K. C. | 2023 | Effects of an Immersive Virtual Reality Intervention on Pain and Anxiety among Pediatric Patients Undergoing Venipuncture: A Randomized Clinical Trial | Title-abstract exclusion |
| 1295 | Wong, C. L.  Lui, M. M. W.  Choi, K. C. | 2019 | Effects of immersive virtual reality intervention on pain and anxiety among pediatric patients undergoing venipuncture: A study protocol for a randomized controlled trial | Title-abstract exclusion |
| 1296 | Wong, Jane  McGuffin, Merrylee  Smith, Mackenzie  Loblaw, Dr Andrew | 2023 | The use of virtual reality hypnosis for prostate cancer patients during transperineal biopsy/gold seed implantation: A needs assessment study | Woring outconmes |
| 1297 | Wong, K. H.  Gruionu, L. G.  Cheng, P.  Abshire, P.  Saveliev, V.  Mun, S. K.  Cleary, K.  Weinberg, I. N. | 2007 | PETglove™: A new technology for portable molecular imaging | Title-abstract exclusion |
| 1298 | Woodard, S.  Kleiman, K. | 2024 | Virtual Reality Simulation–Based Training in Image-Guided Breast Intervention in Low- and Middle-Income Countries | Title-abstract exclusion |
| 1299 | Wooding, E. L. | 2021 | Virtual reality reduced measured levels of pain, fear and anxiety scores during venepuncture for children aged 5-12 years compared to control | Title-abstract exclusion |
| 1300 | Woodle, E. S.  Peddi, V. R.  Tomlanovich, S.  Mulgaonkar, S.  Kuo, P. C. | 2010 | A prospective, randomized, multicenter study evaluating early corticosteroid withdrawal with Thymoglobulin in living-donor kidney transplantation | Title-abstract exclusion |
| 1301 | Wu, B.  Klatzky, R. L.  Shelton, D.  Stetten, G. D. | 2005 | Psychophysical evaluation of in-situ ultrasound visualization | Title-abstract exclusion |
| 1302 | Wu, Jiajun  Gao, Lei  Shi, Qiao  Qin, Chunhui  Xu, Kai  et al | 2023 | Accuracy Evaluation Trial of Mixed Reality-Guided Spinal Puncture Technology | Title-abstract exclusion |
| 1303 | Wu, Jianmei  Xu, Xinfen  Wang, Hua  Shi, Xieli  et al | 2024 | Exploring the effectiveness of virtual technology combined with lidocaine cream in pediatric puncture pain management | Title-abstract exclusion |
| 1304 | Wu, Leilei  Deng, Qinfang  Xu, Ze  Zhou, Songwen  Li, Chao  Li, Yi-Xue | 2020 | A novel virtual barcode strategy for accurate panel-wide variant calling in circulating tumor DNA | Title-abstract exclusion |
| 1305 | Wu, Qingming  Wang, Yubin  Lu, Lili  Chen, Yong  Long, Hui  Wang, Jun | 2022 | Virtual Simulation in Undergraduate Medical Education: A Scoping Review of Recent Practice | Title-abstract exclusion |
| 1306 | Wu, X.  Shi, L.  Xia, Y.  Wang, K. P.  Li, Q. | 2018 | Intrabronchial display of hilar-mediastinal lymph nodes by virtual bronchoscopic navigation system | Title-abstract exclusion |
| 1307 | Wu, Z.  Fu, J.  Wang, Z.  Li, X.  Li, J.  Pei, Y.  Pei, G.  Li, D.  Guo, Z.  Fan, H. | 2015 | Three-dimensional virtual bone bank system for selecting massive bone allograft in orthopaedic oncology | Title-abstract exclusion |
| 1308 | Xiang, Wenxin  Li, Deliang  Sun, Jiabing  Liu, Jiawei  Zhou, Guowei  Gao, Yuan  Cui, Xiaoyu | 2021 | FPGA-Based Two-Dimensional Matched Filter Design for Vein Imaging Systems | Title-abstract exclusion |
| 1309 | Xie, H.  Gao, F.  Zheng, X. | 2021 | Research and application of a teaching platform for combined spinal-epidural anesthesia based on virtual reality and haptic feedback technology | Title-abstract exclusion |
| 1310 | Xie, Sujun  Grimstrup, Soren  Nayahangan, Leizl Joy  Wang, Zheng  Wan, Xing  Konge, Lars | 2023 | Using a novel virtual-reality simulator to assess performance in lumbar puncture: a validation study | Title-abstract exclusion |
| 1311 | Xie, X.  Zhao, C.  Du, R.  Yu, X.  Zhang, Y. | 2011 | Virtual reality based auxiliary robot system for liver | Title-abstract exclusion |
| 1312 | Xie, Y.  Li, M.  Ou, X.  Zheng, S.  Gao, Y.  Xu, X.  et al | 2023 | IP10 and Anti-HBc can Predict Virological Relapse and HBsAg Loss in Chronic Hepatitis B Patients after Nucleos(t)ide Analog Discontinuation | Title-abstract exclusion |
| 1313 | Xu, L.  Zhao, C.  Sun, L. | 2020 | Virtual Haptic Simulation for the VR-Based Biopsy Surgical Navigation | Title-abstract exclusion |
| 1314 | Xuan, J. H.  Wang, Y.  Sesterhenn, I. A.  Moul, J. W.  Mun, S. K. | 1998 | 3-D model supported prostate biopsy simulation and evaluation | Title-abstract exclusion |
| 1315 | Xynopoulos, D.  Stasinopoulou, M.  Dimitroulopoulos, D.  Tsamakides, K.  Arhavlis, E.  Kontou, M.  Tavernaraki, A.  Paraskevas, E. | 2002 | Colorectal polyp detection with virtual colonoscopy (computed tomographic colonography); the reliability of the method | Title-abstract exclusion |
| 1316 | Yamamoto, S.  Maeda, N.  Yoshimura, K.  Oka, M. | 2013 | Intraoperative detection of sentinel lymph nodes in breast cancer patients using ultrasonography-guided direct indocyanine green dye-marking by real-time virtual sonography constructed with three-dimensional computed tomography-lymphography | Title-abstract exclusion |
| 1317 | Yang, C. H.  Chen, L. H.  Lin, Y. S.  Hsu, C. Y.  Tung, M. C.  Huang, S. W.  Wu, C. H.  Ou, Y. C. | 2023 | Incorporating VR-RENDER Fusion Software in Robot-Assisted Partial Prostatectomy: The First Case Report | Title-abstract exclusion |
| 1318 | Yang, J.  Guo, J.  Tang, Y.  Huang, L.  Wiley, J.  Zhou, Z.  Whittemore, R. | 2019 | The mediating effect of coping styles and self‐efficacy between perceived stress and satisfaction with QOL in Chinese adolescents with type 1 diabetes | Title-abstract exclusion |
| 1319 | Yang, J.  Yu, L.  Wang, L.  Li, H.  An, Q. | 2016 | Study on mechanical characterization of liver tissue based on haptic devices for virtual surgical simulation | Title-abstract exclusion |
| 1320 | Yang, S. C.  Hsu, H. H.  Hsu, G. C.  Chung, P. C.  Guo, S. M.  Lo, C. S.  Yang, C. W.  Lee, S. K.  Chang, C. I. | 2005 | 3D localization of clustered microcalcifications using cranio-caudal and medio-lateral oblique views | Title-abstract exclusion |
| 1321 | Yanof, J.  Bauer, C.  Wood, B. | 2004 | Tactile feedback and display system for CT-guided, robot-assisted percutaneous procedures | Title-abstract exclusion |
| 1322 | Yanof, J.  Haaga, J.  Klahr, P.  Bauer, C.  Nakamoto, D.  Chaturvedi, A.  Bruce, R. | 2001 | CT-integrated robot for interventional procedures: Preliminary experiment and computer-human interfaces | Title-abstract exclusion |
| 1323 | Yao, M.  Hasturk, H.  Kantarci, A.  Gu, G.  Garcia-Lavin, S.  Fabbi, M.  Park, N.  Hayashi, H.  Attala, K.  French, M. A.  et al., | 2014 | A pilot study evaluating non-contact low-frequency ultrasound and underlying molecular mechanism on diabetic foot ulcers | Title-abstract exclusion |
| 1324 | Yarmus, L.  Feller-Kopman, D. | 2010 | Bronchoscopes of the Twenty-First Century | Title-abstract exclusion |
| 1325 | Yeo, C. T.  Ungi, T.  Leung, R.  Moult, E.  Sargent, D.  McGraw, R.  Fichtinger, G. | 2018 | Augmented reality assistance in training needle insertions of different levels of difficulty | Title-abstract exclusion |
| 1326 | Yi, Z. Q.  Li, L.  Mo, D. P.  Zhang, J. Y.  Zhang, Y.  Bao, S. D. | 2008 | Preoperative surgical planning and simulation of complex cranial base tumors in virtual reality | Title-abstract exclusion |
| 1327 | Yoshida, H.  Masutani, Y.  MacEnaney, P.  Dachman, A. | 2001 | Computer-aided detection of polyps in CT colonography based on geometric features | Title-abstract exclusion |
| 1328 | Yoshida, S.  Taniguchi, N.  Moriyama, S.  Matsuoka, Y.  Saito, K.  Fujii, Y. | 2020 | Application of virtual reality in patient explanation of magnetic resonance imaging–ultrasound fusion prostate biopsy | Title-abstract exclusion |
| 1329 | Yoshii, N.  Yamada, K.  Niki, M.  Imoto, W.  Yamairi, K.  Shibata, W.  et al | 2021 | Invasive pulmonary aspergillosis caused by Aspergillus terreus diagnosed using virtual bronchoscopic navigation and endobronchial ultrasonography with guide sheath and successfully treated with liposomal amphotericin B | Title-abstract exclusion |
| 1330 | You, S.  Sun, Y.  Chaney, E. J.  Zhao, Y.  Chen, J.  Boppart, S. A.  Tu, H. | 2018 | Slide-free virtual histochemistry (Part II): Detection of field cancerization | Title-abstract exclusion |
| 1331 | Young, D.  Ng, P. Y.  Cheng, D.  Hong, L. C. | 2019 | Effects of Physical Activity Intervention for Chinese People With Severe Mental Illness | Title-abstract exclusion |
| 1332 | Yu, Haiyang  Zhou, Zhi  Lei, Xuefeng  Liu, Huaqing  Fan, Guoxin  He, Shisheng | 2019 | Mixed Reality-Based Preoperative Planning for Training of Percutaneous Transforaminal Endoscopic Discectomy: A Feasibility Study | Title-abstract exclusion |
| 1333 | Yu, P.  McClain, M.  Xuan, J.  Wang, Y.  Sesterhenn, I. A.  Moul, J. W.  Zhang, W.  Mun, S. K. | 1999 | Model supported virtual environment for prostate cancer pattern analysis | Title-abstract exclusion |
| 1334 | Yu, P.  Pan, J.  Qin, H.  Hao, A.  Wang, H. | 2020 | Real-time suturing simulation for virtual reality medical training | Title-abstract exclusion |
| 1335 | Yuan, J. C.  Rodriguez, S.  Caruso, T. J. | 2021 | Unique considerations of virtual reality utilization for perioperative pediatric patients | Title-abstract exclusion |
| 1336 | Yücel, I.  Çavuş, B.  Salhaoǧlu, M.  Güder, N. | 2023 | Determining the Effect of Using Virtual Reality Glasse and Music Therapy During Liver Biopsy on Treatment Satisfaction and Anxiety Level | Meeting abstract |
| 1337 | Yue, Wei  Yuan, Hong  Mao, Xiao-rong  Deng, Yong-dong  Chen, Lin | 2013 | Clinical efficacy of various antiviral-based strategies to treat chronic hepatitis patients with positivity for hepatitis B e antigen and rtN236T mutation | Title-abstract exclusion |
| 1338 | Zaman, F.  Bach, C.  Kumar, P.  Kachralis, S.  Buchholz, N.  Masood, J.  Junaid, I. | 2012 | Acceleration of competency in renal access skills by using Virtual Reality Perc-Mentor Trainer | Title-abstract exclusion |
| 1339 | Zavlanou, C.  Savary, V.  Mermet, S.  Sander, D.  Corradi-Dell’Acqua, C.  Rudrauf, D.  Tisserand, Y.  Sahyoun, C. | 2024 | Virtual reality vs. tablet for procedural comfort using an identical game in children undergoing venipuncture: a randomized clinical trial | Title-abstract exclusion |
| 1340 | Zeng, J.  Bauer, J.  Zhang, W.  Sesterhenn, I.  Moul, J.  Mun, S. K. | 1999 | Prostate biopsy schemes: 3-d visualization-based evaluation | Title-abstract exclusion |
| 1341 | Zeng, J.  Bauer, J. J.  Yao, X.  Zhang, W.  McLeod, D. G.  Sesterhenn, I. A.  Connelly, R. R.  Moul, J. W.  Mun, S. K. | 2000 | Investigating 3D tumor distribution for optimized diagnosis of prostate cancer | Title-abstract exclusion |
| 1342 | Zhai, J.  Karuppasamy, K.  Zyayanjanja, R.  Fisher, M.  Fisher, A. C.  Gould, D.  How, T. | 2013 | A sensor for needle puncture force measurement during interventional radiological procedures | Title-abstract exclusion |
| 1343 | Zhang, Fengfeng  Chen, Long  Miao, Wenhua  Sun, Lining | 2020 | Research on Accuracy of Augmented Reality Surgical Navigation System Based on Multi-View Virtual and Real Registration Technology | Title-abstract exclusion |
| 1344 | Zhang, J. S.  Qu, L.  Wang, Q.  Gui, Q. P.  Hou, Y. Z.  et al | 2018 | Implement of multimodal navigation-based virtual reality in the needle biopsy of intracranial eloquent lesions | Title-abstract exclusion |
| 1345 | Zhang, Lyutianyang | 2023 | Optimized Resource Allocation in Mobile Edge Communication, 5G Data Multiplexing, and WiFi Channel Access | Title-abstract exclusion |
| 1346 | Zhang, L.  Ye, Y.  Niu, B.  Xiong, G.  Yang, D. | 2022 | A simulation framework for ultrasound-guided minimally invasive robotic breast surgery | Title-abstract exclusion |
| 1347 | Zhang, L. W.  Liu, Y. G.  Wu, C. Y.  Xu, S. J.  Zhu, S. G. | 2011 | Radiofrequency thermocoagulation rhizotomy for recurrent trigeminal neuralgia after microvascular decompression | Title-abstract exclusion |
| 1348 | Zhang, X.  Duan, J.  Zhu, L.  Kavan, L. | 2018 | A virtual puncture surgery system based on multi-layer soft tissue and force mesh | Title-abstract exclusion |
| 1349 | Zhang, X.  Xiao, H.  Chen, Y. | 2019 | Evaluation of a WeChat‐based life review programme for cancer patients: a quasi‐experimental study | Title-abstract exclusion |
| 1350 | Zhang, Y.  Chen, F.  Liang, T.  Gao, Z.  Hu, L.  Wang, L. | 2023 | Construction of bone marrow cytology examination learning platform based on virtual simulation experiment teaching system | Title-abstract exclusion |
| 1351 | Zhang, Yi  Yu, Cheng-fan  Liu, Jin-shun  Wang, Gang  Zhu, He  Na, Yan-qun | 2013 | Training for percutaneous renal access on a virtual reality simulator | Title-abstract exclusion |
| 1352 | Zhang, Yining  Zhang, Hengsheng  Liu, Xiaokai  Zhao, Chenglin  Xu, Fangmin | 2023 | Digital twin-enabled deep reinforcement learning for joint scheduling of ultra-reliable low latency communication and enhanced mobile broad band: A reliability-guaranteed approach | Title-abstract exclusion |
| 1353 | Zhang, Y.  Zheng, J.  Liu, J. | 2023 | Intraocular Suture Technique for Flapless Two-Point Fixation of Four Fenestrated Haptics Intraocular Lenses | Title-abstract exclusion |
| 1354 | Zhang, Z. S.  Wu, Y.  Zheng, B. | 2024 | A Review of Cognitive Support Systems in the Operating Room | Title-abstract exclusion |
| 1355 | Zhao, Hanjiang  Cheng, Mengjia  Huang, Jingyang  Li, Meng  Cheng, Huanchong  Tian, Kun  Yu, Hongbo | 2023 | A virtual surgical prototype system based on gesture recognition for virtual surgical training in maxillofacial surgery | Title-abstract exclusion |
| 1356 | Zheng, C.  Li, J.  Zeng, G.  Ye, W.  Sun, J.  Hong, J.  Li, C. | 2019 | Development of a Virtual Reality Preoperative Planning System for Postlateral Endoscopic Lumbar Discectomy Surgery and Its Clinical Application | Title-abstract exclusion |
| 1357 | Zheng, Ting  Xie, Huihong  Gao, Fei  Gong, Cansheng  Lin, Wei  Ye, Peng  Liu, Yuqing  He, Bingwei  Zheng, Xiaochun | 2023 | Research and application of a teaching platform for combined spinal-epidural anesthesia based on virtual reality and haptic feedback technology | Title-abstract exclusion |
| 1358 | Zhong, Hualiang  Peters, Terry | 2007 | A real time hyperelastic tissue model | Title-abstract exclusion |
| 1359 | Zhong, J.  Zhong, F.  Tang, L.  Zhang, J.  Feng, H.  Deng, J.  Zhang, J.  He, L. | 2023 | A Virtual Surgery System for Lung Biopsy | Title-abstract exclusion |
| 1360 | Zhou, Jian | 2011 | Study on the application of mscte in diagnosis and treatment of crohn's disease | Title-abstract exclusion |
| 1361 | Zhou, L.  Sato, R. | 2020 | Development and application of a surgical process simulation system using VR technology | Title-abstract exclusion |
| 1362 | Zhou, Yang  Gu, Wei  Peng, Mingzheng  Cheng, Xinghua | 2022 | Research on "Augmented Reality" Assisted Precise Thoracoscopic Resection of Pulmonary Nodules | Title-abstract exclusion |
| 1363 | Zhou, Zhi  Hu, Shuo  Zhao, Yong-zhao  Zhu, Yan-jie  Wang, Chuan-feng  Gu, Xin  Fan, Guo-xin  He, Shi-sheng | 2019 | Feasibility of Virtual Reality Combined with Isocentric Navigation in Transforaminal Percutaneous Endoscopic Discectomy: A Cadaver Study | Title-abstract exclusion |
| 1364 | Zhou, Zeyang  Jiang, Shan  Yang, Zhiyong  Zhou, Lin | 2019 | Personalized planning and training system for brachytherapy based on virtual reality | Title-abstract exclusion |
| 1365 | Zhou, Zeyang  Yang, Zhiyong  Jiang, Shan  Jiang, Bowen  Xu, Bin  Zhu, Tao  Ma, Shixing | 2022 | Personalized virtual reality simulation training system for percutaneous needle insertion and comparison of zSpace and vive | Title-abstract exclusion |
| 1366 | Zhou, Zeyang  Yang, Zhiyong  Jiang, Shan  Zhang, Fujun  Yan, Huzheng | 2019 | Design and validation of a surgical navigation system for brachytherapy based on mixed reality | Title-abstract exclusion |
| 1367 | Zhou, Zeyang  Yang, Zhiyong  Jiang, Shan  Zhuo, Jie  Li, Yuhua  Zhu, Tao  Ma, Shixing  Zhang, Jingqi | 2023 | Validation of a surgical navigation system for hypertensive intracerebral hemorrhage based on mixed reality using an automatic registration method | Title-abstract exclusion |
| 1368 | Zhou, Zeyang  Yang, Zhiyong  Jiang, Shan  Zhuo, Jie  Zhu, Tao  Ma, Shixing | 2022 | Surgical Navigation System for Hypertensive Intracerebral Hemorrhage Based on Mixed Reality | Title-abstract exclusion |
| 1369 | Zhu, Huayuan  Jiang, Rui  Shen, Hui  Wu, Wei  Yang, Yilian  et al | 2021 | Venetoclax Combined with Dose-Adjusted R-EPOCH (VR-DA-EPOCH) As Treatment of Richter's Syndrome: A Real-World Study | Title-abstract exclusion |
| 1370 | Zhu, H.  Li, N.  Zhao, Z.  Wang, H.  Zhang, B.  Na, Y. | 2018 | Collecting system percutaneous access using MR-assisted guidance: first human phantom experience | Title-abstract exclusion |
| 1371 | Zhu, Y.  Jia, X. H.  Zhou, W.  Zhan, W. W.  Zhou, J. Q. | 2020 | Qualitative evaluation of virtual touch imaging quantification: A simple and useful method in the diagnosis of breast lesions | Title-abstract exclusion |
| 1372 | Zhuang, Yu  Chen, Jie  Liu, Qingcheng  Zou, Fan  Lin, Yuheng  An, Qinglong  Yu, Hongbo | 2021 | Preliminary study on mechanical characteristics of maxillofacial soft and hard tissues for virtual surgery | Title-abstract exclusion |
| 1373 | Zhuang, Yan  Sun, Juanjuan  Liu, Jiaqiang | 2021 | RETRACTED: Diagnosis of Chronic Kidney Disease by Three-Dimensional Contrast-Enhanced Ultrasound Combined with Augmented Reality Medical Technology (Retracted Article) | Title-abstract exclusion |
| 1374 | Zito, Francesco Alfredo  Verderio, Paolo  Simone, Giovanni  Angione, Vito  Apicella, Paola  et al | 20 | Reproducibility in the diagnosis of needle core biopsies of non-palpable breast lesions: an international study using virtual slides published on the world-wide web | Title-abstract exclusion |
| 1375 | Zu, Chao | 2009 | Research on key techniques of force feedback in virtual surgery simulation based on deformable models | Title-abstract exclusion |
| 1376 | Zuk, M.  Majak, M.  Dȩbowski, G.  Światek-Najwer, E.  Popek, M.  Pietruski, P.  Jaworowski, J. | 2018 | Virtual reality in the pre-operative planning and training for oncological treatment using MentorEye system: Preliminary results | Title-abstract exclusion |
| 1377 | Zukhra, Ririn Muthia  Wanda, Dessie | 2024 | Pain management associated with arteriovenous fistula cannulation among children undergoing haemodialysis: Systematic review | Title-abstract exclusion |
| 1378 | Bai Gengshen | 2017 | Study on the image quality of lungs with different doses of low-dose CT scanning and feasibility study for clinical application | Title-abstract exclusion |
| 1379 | Bai Meng | 2020 | Application of VR Technology in Teaching Surgery | Title-abstract exclusion |
| 1380 | Bai Yang  Li Weixiong  Zhang Jing  Wei Jieqin  Qin Mingan  Zhao Cheng | 2014 | Comparative analysis of parotid CT imaging, lip gland biopsy and clinical correlation results in dry syndrome | Title-abstract exclusion |
| 1381 | Bu Jun | 2010 | Clinical application of 64-slice spiral CT angiography for analyzing anatomical variations of the left gastric artery | Title-abstract exclusion |
| 1382 | Cai Bulei  Zhang Yufan  Yang Luying  Bao Han  She Jianzhen  Kong Liang  Tian Lei  Deng Zhongrong  Xu Haokun | 2023 | Development and utilization of VR maxillofacial combat trauma first aid training system in the context of modern warfare | Title-abstract exclusion |
| 1383 | Cao Hanbo  Wang Mei  Wang Heping | 2017 | Diagnostic and differential diagnostic value of MSCT in isolated pulmonary nodules (≤2 cm) with pleural depression sign | Title-abstract exclusion |
| 1384 | Zeng Yanwen  Xu Tianchun  Yue Longwang | 2006 | Simulation of microsurgical vessels with forceful sensation | Title-abstract exclusion |
| 1385 | Zeng Yangmei | 2021 | Personalized three-dimensional visualization model of the liver combined with ultrasound in precision-guided radiofrequency ablation of hepatocellular carcinoma | Title-abstract exclusion |
| 1386 | Chen Bo | 2013 | A study of small-dose contrast CTU in percutaneous nephrolithotripsy for lithotripsy (PCNL) | Title-abstract exclusion |
| 1387 | Chen Hongmao | 2007 | A study on the characteristics of Doppler flow spectrum of the superior vena cava in pulmonary hypertension and its significance | Title-abstract exclusion |
| 1388 | Chen Ling  Zhao Zhenyu | 2022 | Mixed reality navigation technology will be a new direction for neurosurgical procedure navigation | Title-abstract exclusion |
| 1389 | Chen Mingji | 2018 | Impact of lumbar virtual reality preoperative planning technology on training for laminectomy surgery | Title-abstract exclusion |
| 1390 | Chen Rui | 2020 | Functional Improvement and Interactive Performance Study of Six Degrees of Freedom Tandem Force Feedback Devices | Title-abstract exclusion |
| 1391 | Chen Shuai  Li Kai Rong  Zhang Nanxin  Meng Gang | 2023 | A pragmatic augmented reality navigation method for hip puncture procedures | Title-abstract exclusion |
| 1392 | Chen Weijian  Wang Xiaotong  Yang Yunjun  Tan Xianxi  Zhong Ming  Duan Yuxia  Wang Yong  Zhuge Qichuan | 2008 | CT angiographic evaluation of a rabbit basilar artery spasm model | Title-abstract exclusion |
| 1393 | Chen Weijian  Wang Xiaotong  Yang Yunjun  Tan Xianxi  Zhong Ming  Wang Yong  Zhuge Qichuan | 2008 | Dynamic evaluation of a rabbit basilar artery spasm model by multislice spiral CT angiography | Title-abstract exclusion |
| 1394 | Chen Xiaomin  Luo Yun  Wu Yuelong  Jiang Min | 2010 | Studies on capsaicin receptor, substance P and mast cell changes in the colonic mucosa of patients with irritable bowel syndrome | Title-abstract exclusion |
| 1395 | Chen Yajin  Zhang Lei | 2023 | The value of laparoscopic surgery in the treatment of gallbladder cancer in the era of comprehensive treatment | Title-abstract exclusion |
| 1396 | Chan Chung Yee  Lau Yu Ching  Ho Ping Wai  Huang Jinyue  Hung Man Yiu  Liao Zhengjian  Chen Shou  Su Xiaohang | 2019 | Mixed reality-based lateral ventricle puncture training system in medical education training | Title-abstract exclusion |
| 1397 | Cheng Jianhong  Yang Lian  Liu Jianfeng  Li Lu  Hong Li | 2022 | Evaluation of the effect of VR/AR technology in teaching obstetrics and gynecology | Title-abstract exclusion |
| 1398 | Cong Zhibin | 2009 | Experimental study of the value of echocardiography in the diagnosis of acute pulmonary embolism in rabbits | Title-abstract exclusion |
| 1399 | Cui Liming | 2005 | Experimental and clinical study on the diagnosis of pulmonary artery embolism by 16-slice spiral CT | Title-abstract exclusion |
| 1400 | Cui Miao Miao  Xu Xiumei  Luo Xiaoyan  Cui Renshan | 2023 | Advances in the use of virtual reality in cancer-related pain care | Title-abstract exclusion |
| 1401 | Cui Minyi | 2006 | A study of 64-slice spiral CT angiography in the abdomen | Title-abstract exclusion |
| 1402 | Cui Yahui  Yu Jianhua  Zhang Binhai  Zhang Zhongdong  Jiang Yuhong | 2019 | A case of 3D-SLICER-assisted cell phone localization for brain biopsy | Title-abstract exclusion |
| 1403 | Dai Yanyan | 2018 | A comparative study of MSCT coronary CTA and CAG in the diagnosis of coronary artery disease in menopausal women | Title-abstract exclusion |
| 1404 | Dai Lian  Yue Kuitao  Liang Peng  Huang Kechang | 2020 | Analysis of anatomical factors affecting the puncture of the pterygopalatine ganglion in the frontal zygomatic angle | Title-abstract exclusion |
| 1405 | Dessa  Si Weixin  Qian Yinling  Zheng Rui  Wang Qiong  Xu Dongliang  Peng Yanjun  Wang Ping'an | 2019 | Design of human-computer interaction interface for virtual microscopic cataract surgery system | Title-abstract exclusion |
| 1406 | Deng Yanjia | 2012 | Study of multislice spiral CT in the portal system of normal subjects and patients with portal hypertension | Title-abstract exclusion |
| 1407 | Ding Juan | 2004 | An applied study of low-dose scanning of the chest with multislice spiral CT | Title-abstract exclusion |
| 1408 | Dong Chunling  Fan Weipeng  Li Shivei | 20 | Diagnostic value of bronchial artery MSCTA in chronic inflammation of the lung and lung cancer | Title-abstract exclusion |
| 1409 | Dong Guoju  Liu Jiangang  Shi Dazhuo  Rao Li  Liu Guihua | 2005 | Effects of healing infarction solution on myocardial energy metabolism and endothelial dysfunction in rats with acute myocardial infarction | Title-abstract exclusion |
| 1410 | Dong Junqiang | 2009 | Study on the application of 64-VCT and multiple post-processing techniques in the diagnosis of colorectal cancer | Title-abstract exclusion |
| 1411 | Du Cheng | 2018 | Study of ultrasound-guided microwave ablation virtual surgery system for tumors | Title-abstract exclusion |
| 1412 | Feng Shiting  Li Ziping  Minyi Cui  Reformed  Zhou Xuhui  Peng Zhenpeng  Sun Chanhui  Fan Miao | 2006 | Application of 64-slice spiral CT angiography to abdominal tumors | Title-abstract exclusion |
| 1413 | Feng Yuan | 2012 | Research on key technology of virtual knee surgery system based on force feedback | Title-abstract exclusion |
| 1414 | Gao Fei | 2012 | Clinical study of cholangiography by low-tension multilayer spiral CT with drinking water | Title-abstract exclusion |
| 1415 | Gao Xuguang | 2023 | Application of 3D-Slicer software three-dimensional reconstruction-assisted puncture drainage for hemorrhagic stroke | Title-abstract exclusion |
| 1416 | Gao Zhan  Pan Fei  Wang Jiehua  Pan Haiyan  Jiang Zheng Zheng | 2015 | Virtual Soft Tissue Needling and Force Sense Interaction | Title-abstract exclusion |
| 1417 | Ge Yaping | 2017 | A comparative study of the diagnostic value of 3.0T TOFu-MRA, TOF-MRA and DSA for carotid artery stenosis | Title-abstract exclusion |
| 1418 | Ge Zhiming  Shi Zhenbushi  Yang Yun  Fan Xianxian  Zhao Hongbo  Li Yuxiang | 2022 | Study on the application of diluted nitrous oxide for analgesia during postoperative dressing change in rectal perianal abscesses | Title-abstract exclusion |
| 1419 | Guo Chenxi  Ou Fengrong | 2021 | Progress in the application of virtual reality technology in clinical practice teaching | Title-abstract exclusion |
| 1420 | Guo Qiao  Zhou Yan  Wang Junhao  Han Jianpeng  Feng Jianyong  Wang Ren  Chen Wenbin  Li Yongzhang | 2024 | Dihydroartemisinin in the treatment of prostatitis in mice by promoting bone marrow mesenchymal stem cell homing | Title-abstract exclusion |
| 1421 | Guo Zhaoxiang | 2020 | Research on key technology of ultrasound-guided renal biopsy training system based on mixed reality | Title-abstract exclusion |
| 1422 | Han Donglian  Liu Weiwei  Jiao Ying  Zhang Wei | 2020 | Advances in the use of simulation in neonatal intensive care medicine | Title-abstract exclusion |
| 1423 | Han Qing | 2023 | Application and research of virtual reality-based diagnostic system for abdominal fluid collection | Title-abstract exclusion |
| 1424 | He Wei | 2023 | Application of local radial basis function collocation method to puncture problem based on potential problem | Title-abstract exclusion |
| 1425 | He Xiaohui | 2011 | Research and Implementation of Virtual Surgical Force Sense Information Acquisition System | Title-abstract exclusion |
| 1426 | He Hui  Bo Chen  Cheng Jianmin  Dai Tingting  Xu Lei  Kong Qiuyan | 2017 | Study on the application of small-dose contrast CTU in percutaneous nephrolithotripsy lithotripsy and stone extraction | Title-abstract exclusion |
| 1427 | Hong Zongqi | 2018 | Study on CT diagnosis of lung fungal disease and with microbiology examination | Title-abstract exclusion |
| 1428 | Hu Xi | 2008 | Research and realization of cluster grass scene animation | Title-abstract exclusion |
| 1429 | Hu Yifan  Huang Liping  Liu Yiquan | 2023 | Observation on the effect of applying video stimulation test to prevent anxiety-induced blood donation-related vagal reaction | Title-abstract exclusion |
| 1430 | Hu Yu  Liu Yi | 2020 | Characteristics of Augmented Reality Technology in Neurosurgery Training | Title-abstract exclusion |
| 1431 | Huang Ankang  Gong Yongsheng | 2022 | Progress of the application of lung nodule localization technology in lung cancer surgery | Title-abstract exclusion |
| 1432 | Huang Huaxing | 2016 | Study on small bowel disease by CTE of large and small bowel double filling method combined with mesenteric CTA | Title-abstract exclusion |
| 1433 | Huang Yibin  Duan Xuejun  Longsha  Hu Pingxiang  Xiao Xiaopeng | 2016 | Clinical value of applying ultrasound navigation technology in percutaneous puncture bile duct placement and drainage surgery | Title-abstract exclusion |
| 1434 | Ji Lixin | 2015 | Clinical application of MSCT in the diagnosis of primary renal cancer | Title-abstract exclusion |
| 1435 | Jiang Hong  Wang Peijun  Zhao Xiaohu  Gao Xiaolong | 2016 | Diagnostic value of CT angiography in lung cancer combined with superior vena cava syndrome | Title-abstract exclusion |
| 1436 | Jiang Wenjin  Qirui Sheng  Sun Borin  Cheng Xueling | 2010 | Anatomical imaging study of transabdominal puncture for nucleus pulposus removal in lumbar 5 sacral 1 intervertebral discs | Title-abstract exclusion |
| 1437 | Jiang Yong  Guan Tianmin  Ci Yuan  Zhu Ye  Zhao Peng  Zheng Jiafa  Yang Tao  Zhang Guangyu | 2024 | Application of mixed reality technology in vertebroplasty treatment | Title-abstract exclusion |
| 1438 | Jiang Yonghang | 2020 | Finite element simulation and experimental study of muscle tissue puncture process | Title-abstract exclusion |
| 1439 | Jin Qian  Deng Kexue  Deng Fusheng  Cao Dongxing  Hu Hejie | 2006 | Application of 16-slice spiral CT angiography in the diagnosis of lower limb vascular lesions | Title-abstract exclusion |
| 1440 | Kang Ya  Li Miaojing | 2019 | Comprehensive puncture virtual training system and DOPS evaluation form in the teaching of bone marrow puncture practice | Title-abstract exclusion |
| 1441 | Kong Fanwu | 2014 | Application value of MSCT pleural depression sign in the diagnosis and differential diagnosis of isolated pulmonary nodules | Title-abstract exclusion |
| 1442 | Kou Siyu  Zhang Yuping  Yang Ganghua | 2021 | Application of Virtual Reality and Augmented Reality Technology in Teaching Geriatric Surgical Arterial Blood Collection Nursing Care | Title-abstract exclusion |
| 1443 | Kuhongbin  Meng Zhiyong  Zhang Yanli  Gao Haixiao  Zhang Weimin  Sun Xinye  Kuhongan | 2021 | Analysis of the application value of simplified augmented reality technology in the preoperative puncture point localization of draining supratentorial cerebral hemorrhage in the elderly | Title-abstract exclusion |
| 1444 | Lan Huan  Ma Xingcai  Zhao Guan Yan  Xie Changji  Li Guang  Wei Chengcong  Zhou Zhiyu | 2020 | Clinical observation of 3D-slicer software-assisted neuroendoscopic surgery in the treatment of hypertensive cerebral hemorrhage | Title-abstract exclusion |
| 1445 | Lang Xiaoyan  Wang Yun | 2024 | Research progress of virtual reality technology in acute pain in children | Title-abstract exclusion |
| 1446 | Li Bojun  Lin Fangyu  Cheng Fan | 2020 | Impact and application of pooled systems on percutaneous nephrolithotripsy | Title-abstract exclusion |
| 1447 | Li Chunhai | 2017 | Methods and significance of constructing virtual reality model of lumbar spine degeneration | Title-abstract exclusion |
| 1448 | Li Fengchun  Zhang Chunning  Liu Guanghun | 2008 | Analysis of the value of 16-slice spiral CT for poor display of venous pyelography | Title-abstract exclusion |
| 1449 | Li Hong  Li Zhikui  Zhu Xiulan  Zhang Deqing  Liu Zhijia | 2016 | Application of spiral CT dual-phase injection in pulmonary angiography combined with lower extremity venography examination | Title-abstract exclusion |
| 1450 | Li Hongyang | 2016 | Measurement of Mechanical Parameters of Ex vivo Soft Tissue and Its Application in Virtual Surgical System | Title-abstract exclusion |
| 1451 | Li Hui | 2015 | Precision Thoracic Surgery: A New Beginning in the Minimally Invasive Era | Title-abstract exclusion |
| 1452 | Li Jiangshan  Li Shaodong  Rong Yutao  Xu Kai | 2007 | Application of 16-slice CT reconstruction technology in the diagnosis of colonic space-occupying lesions. | Title-abstract exclusion |
| 1453 | Li Kai | 2007 | Three-dimensional reconstruction of hepatic veins and visualization of puncture access for intrahepatic portal vein shunt surgery. | Title-abstract exclusion |
| 1454 | Li Mingshen  Wang Yutang  Gong Weiru  Wang Zhifeng  Shi Xiangmin  Li Tiande | 2002 | A case of ICD implantation in a patient with ischemic cardiomyopathy and ventricular tachycardia combined with expiratory failure. | Title-abstract exclusion |
| 1455 | Li Peihe | 2023 | Application of virtual reality technology combined with bronchoscopy in pulmonary hilar and mediastinal space-occupying lesions | Title-abstract exclusion |
| 1456 | Li Ruidong  Yang Changya  Wang Feng  Song Hu  Ding Ning  Zhang Kongyuan | 2023 | Application of three-dimensional modeling digital navigation technology in microwave ablation of liver malignant tumors  The value of multislice spiral CT and its portal vein angiography in the preoperative application of TIPS combined with gastric coronary vein embolization | Title-abstract exclusion |
| 1457 | Li Songwei  Zhang Jie  Yan Dong  Wang Jiaping  Sun Yong  Tong Yuyun  Li Lin  Xie Wenyu  Li Yingchun | 2015 | The value of multislice spiral CT and its portal vein angiography in the preoperative application of TIPS combined with gastric coronary vein embolization | Title-abstract exclusion |
| 1458 | Li Wenchan  Hu Daoyu  Ming Changsheng  Xiao Ming  Huang Wenhua  Song Jinmei | 2006 | Multislice spiral CT performance after combined pancreas-kidney transplantation | Title-abstract exclusion |
| 1459 | Li Xianghua | 2013 | A controlled study of 3.0T MRA and DSA in the diagnosis of anterior cerebral circulation artery stenosis | Title-abstract exclusion |
| 1460 | Li Xiaoqing  Deli Li  Yin Xin  Sun Hairui  Li Hongyan | 2021 | Advances in the use of virtual reality technology in the reduction of operative pain in children | Title-abstract exclusion |
| 1461 | Li Yancui  Xin Ruiqiang  Cai Jianxin  Yang Yanhui  Ma Daqing | 2018 | CT image analysis of spherical lung atelectasis | Title-abstract exclusion |
| 1462 | Li Yanfei  Wang Ronghai  Lin Xia  Wang Hongwei | 2020 | Research on the construction of lumbar puncture surgery simulation model library | Title-abstract exclusion |
| 1463 | Li Yifan  Wang Jing  Wang Changyuan | 2020 | Application of virtual reality technology in emergency medicine education | Title-abstract exclusion |
| 1464 | Li Yong | 2006 | Study of Magnetic Resonance Gd-DTPA Dynamic Enhancement of Intrahepatic Nodular Lesions, 3D Reconstruction and Fusion of MR Images of Liver Pipeline System and Intrahepatic Occupying Lesions | Title-abstract exclusion |
| 1465 | Li Yun | 2012 | Research on key technology of virtual colonoscopy path navigation and preliminary design of auxiliary examination system | Title-abstract exclusion |
| 1466 | Li Zaijun  Ji Gang  Li Qi  Zhang Jianguong  Gan Tian | 2012 | Diagnostic value of multislice spiral CT for retroperitoneal fibrosis | Title-abstract exclusion |
| 1467 | Li Zhiqiang | 2018 | Application of virtual reality and augmented reality based localization technology in minimally invasive puncture treatment of cerebral hemorrhage | Title-abstract exclusion |
| 1468 | Li Zhonghai  Hou Shuxun | 2021 | Rationalization of minimally invasive spinal surgery to emphasize the occurrence and prevention of complications | Title-abstract exclusion |
| 1469 | Liag Dexian | 2020 | Mixed Reality-Based Human-Computer Interaction Control of Prostate Particle Implantation Robot | Title-abstract exclusion |
| 1470 | Liang Jueyin | 2005 | Diagnostic value of multislice CT angiography (MSCTA) in vertebrobasilar ischemic vertigo | Title-abstract exclusion |
| 1471 | Liang Liangliang | 2018 | Study on minimally invasive treatment of renal tumors assisted by magnetic positioning four-dimensional navigation system | Title-abstract exclusion |
| 1472 | Liang Zhimei | 2009 | Comparative study of CT scanning and imaging of parotid gland with X-ray imaging in dry syndrome | Title-abstract exclusion |
| 1473 | Lin Wei | 2021 | Research and Development of Neurosurgical Virtual Surgical Training System with Force Feedback | Title-abstract exclusion |
| 1474 | Liu Chenwu | 2018 | The application value of HRCT signs in the diagnosis of tiny ground-glass nodule-like lung adenocarcinoma | Title-abstract exclusion |
| 1475 | Liu Dezhang  Chow Siu Chung  Mok Siu Fung  Chen Yingtao | 2013 | Application of MSCT in puncture localization for radiofrequency ablation of primary hepatocellular carcinoma. | Title-abstract exclusion |
| 1476 | Liu Dingli | 2007 | Long-term follow-up and efficacy evaluation of α-interferon in the treatment of chronic hepatitis B | Title-abstract exclusion |
| 1477 | Liu Qianhui | 2008 | CT study of the relationship between isolated pulmonary nodules and bronchial tubes and blood vessels | Title-abstract exclusion |
| 1478 | Liu GuangJiu | 2007 | Anatomy and visualization study of head and neck digital three-dimensional cross section | Title-abstract exclusion |
| 1479 | Liu Hao | 2013 | Comparative study of the diagnostic value of 320-row dynamic volumetric CTA and 3D-DSA for intracranial small aneurysms | Title-abstract exclusion |
| 1480 | Liu Hongsheng | 2010 | Study on the application of 64-slice CT negative method of biliopancreatic duct imaging in the diagnosis of biliary atresia in infants | Title-abstract exclusion |
| 1481 | Liu Huan | 2020 | Innovative design and application of minimally invasive spinal surgery guidance system based on augmented reality technology | Title-abstract exclusion |
| 1482 | Liu Jiangang  Dong Guoju  Shi Dazhuo  Rao Li  Liu Guihua | 2005 | Experimental study on the ultrastructure and antioxidant effect of Healing Infarction Solution in protecting cardiomyocytes and mitochondria of rats with heart failure after acute myocardial infarction | Title-abstract exclusion |
| 1483 | Liu Jie  Chen Yong  Zhang Zufeng  Wang Dongmei | 2015 | The clinical value of CT scanning combined with three-dimensional reconstruction technology in the diagnosis of bone infectious lesions | Title-abstract exclusion |
| 1484 | Liu Mingshan | 2013 | The significance of multislice CT angiography in the examination of testicular artery origin variation | Title-abstract exclusion |
| 1485 | Liu Quan | 2023 | Research on hybrid soft tissue modeling and collision detection based on RPIM-IXPBD in virtual surgery simulation | Title-abstract exclusion |
| 1486 | Liu Yingqiang | 2004 | Application value of multislice spiral CT in preoperative staging and evaluation of gastric cancer | Title-abstract exclusion |
| 1487 | Liu Yuqing | 2011 | Study on the validation, innovation and training application of the frontal horn puncture method of lateral ventricle based on digital modeling | Title-abstract exclusion |
| 1488 | Liu Yugui | 2013 | Comparative imaging study of multislice spiral CT and DSA in giant hepatocellular carcinoma | Title-abstract exclusion |
| 1489 | Lu Jicheng | 2020 | Application of dobutamine combined with nitroglycerin in partial hepatectomy under controlled low central venous pressure | Title-abstract exclusion |
| 1490 | Lu Qifang  Xiang Li  Zhao Hong  Zhou Haiyan  Zheng Suisheng | 2013 | The value of multislice spiral CT image post-processing technology for preoperative localization of lung mass puncture | Title-abstract exclusion |
| 1491 | Lu Xiong | 2008 | Theoretical and Methodological Research on Force/Tactile Modeling and Perception in Virtual Environments | Title-abstract exclusion |
| 1492 | Lu Yaohong  Zou Yang  Zhang Feng  Li Ping  Wang Daozhen  Wang Hong  Zhu Xiaoqing | 2015 | Practice and thinking of the virtual training system of thoracic puncture for clinical teaching | Title-abstract exclusion |
| 1493 | Lu Yaohong  Zou Yang  Zhang Feng  Li Ping  Wang Hong  Wang Daozhen  Zhu Xiaoqing | 2015 | Study on the learning curve of virtual thoracocentesis | Title-abstract exclusion |
| 1494 | Luo Jing | 2023 | Evaluation index system of virtual simulation intravenous infusion experimental teaching based on CIPP modeling | Title-abstract exclusion |
| 1495 | Luo Jing  Xuemei Tan  Yan Min  Guan Ji | 2022 | Research Progress on the Application of Virtual Reality Technology in the Teaching of Intravenous Fluid Infusion | Title-abstract exclusion |
| 1496 | Lv Baotao | 2010 | The value of 64-slice CT multisignature analysis combined with dynamic enhancement scanning for benign and malignant differential diagnosis of isolated pulmonary nodules | Title-abstract exclusion |
| 1497 | Lv Fangfei | 2015 | Optimization and GPU Parallel Acceleration of Soft Tissue Physical Modeling in Virtual Surgery | Title-abstract exclusion |
| 1498 | Lv Hongbo, Wang Tianmiao, Liu Da, Hu Lei, Tang Zesheng, Shen Hao, Tian Zenmin | 2001 | Research on Robotic Surgical Simulation and Training System Based on Virtual Reality Technology %J High Tech Letters | Title-abstract exclusion |
| 1499 | Lv Jianan | 2016 | Small-dose pre-injection method of CT portal venography in gastroscopic fundic variceal weaning preoperative application | Title-abstract exclusion |
| 1500 | Lv Yinggang  Stormy Junhui  Liu Guiting  Xu Dengguo  Shi Junling  Wang Baogang  Yan Qiaohuan | 2015 | Early qualitative diagnosis of grinding glass nodules in the lungs by multislice spiral CT with multiple reconstruction and post-processing techniques. | Title-abstract exclusion |
| 1501 | Ma Xiaojun  Lin Yanping  Huang Yalei  Sun Wei  Shen Jiakang  Sun Mengxiong  Zuo Dongqing  Fu Zeze  Wang Lei  Fu Qiang  Zhengdong Cai | 2019 | Exploration and application of mixed reality technology for percutaneous posterior spinal kyphoplasty | Title-abstract exclusion |
| 1502 | Ma Yujia | 2007 | Clinical application study of nickel-titanium memory alloy stent in the treatment of airway disorders | Title-abstract exclusion |
| 1503 | Mustache Meng  Wang Tianmiao  Zhang Yuru  U Wusheng  Tian Zenmin | 2003 | Study on the application of remote operation in neurosurgery | Title-abstract exclusion |
| 1504 | Meng Fanggang  Wu Chengyuan  Liu Base  Cui Miao  Yu Mingguang  Liu Yuguang | 2008 | Virtual reality technology assisted radiofrequency thermocoagulation treatment of trigeminal neuralgia | Title-abstract exclusion |
| 1505 | Ni Zhihua | 2009 | Comparative analysis of intracranial arterial stenosis etiology and diagnosis by CTA and DSA | Title-abstract exclusion |
| 1506 | Ning Aidong  Zhang Linfei  Wang Guowei | 2022 | Exploration of the diagnostic value of CT three-dimensional reconstruction imaging on the display of morphologic features and benign and malignant of isolated pulmonary nodules | Title-abstract exclusion |
| 1507 | Pan Juhong  Hu Daoyu | 2014 | Diagnostic value of 64-slice spiral CT post-processing technology for hepatocellular carcinoma | Title-abstract exclusion |
| 1508 | Qiang Jinwei | 2008 | Multi-row spiral CT study of acute intestinal ischemia | Title-abstract exclusion |
| 1509 | Qiang Jinwei  Li Ruokun  Feng Xiaoyuan  Liao Zhihe  He Cheng  Feng Qin  Zhang Biao  Ye Xuanguang | 2010 | Experimental study on the evaluation of acute intestinal ischemia caused by mesenteric artery embolization using multi-row spiral CT | Title-abstract exclusion |
| 1510 | Qin Hao | 2017 | Establishment of training standards and technical methods for first aid training of combatants in battlefield with battle injuries | Title-abstract exclusion |
| 1511 | Qiu Leiyu  Chen Peiyou  Shi Naichang  Wang Yejun  Ken Hui | 2010 | Diagnostic value of multi-row spiral CT in superior vena cava syndrome | Title-abstract exclusion |
| 1512 | Ren Chao | 2018 | Research on collision detection algorithm of deformed body by integrating adaptive ellipsoidal enclosing box | Title-abstract exclusion |
| 1513 | Sang Jiefeng | 2016 | Basic and applied research on the design of shunt channel for intrahepatic portal vein shunt via left branch of portal vein assessed by multi-row spiral CT angiography | Title-abstract exclusion |
| 1514 | She Shouzhang  Yu Weifeng | 2023 | Strengthening the research of clinical monitoring intelligence to enhance the safety of patients during perianesthesia surgery | Title-abstract exclusion |
| 1515 | Shen Feng  Qian Xuerong  Xie Guohua  Zhang Chuanchen  Di Yujin | 2008 | Application of multislice spiral CT in rib lesions: comparison of cross-sectional, MPR and VR images | Title-abstract exclusion |
| 1516 | Shen Huiqin  Yan Xuetao  Julia Zhang  Deng Jiangtao  Ying Xiong  Linlin Jiang  Qiongyue Zhang  Wu Tangjing  Zhe Jin  Zhang Zongze  Wang Yanlin | 2019 | Comparison of the effects of different concentrations of pyruvate dialysis solution for intraperitoneal resuscitation in rats with hemorrhagic shock. | Title-abstract exclusion |
| 1517 | Shi Yibing | 2008 | Clinical application study of 64-slice spiral CT simple pulmonary arteriography in the diagnosis of pulmonary embolism | Title-abstract exclusion |
| 1518 | Sze Shui Ho  Ying Lu  Yan Weiwei | 2015 | Role of multi-row coronary CT in identifying complete right bundle branch block combined with coronary atherosclerosis | Title-abstract exclusion |
| 1519 | Song Kunpeng | 2009 | Comparative analysis of the diagnostic value of exercise electrocardiogram loading test, 64-row volumetric CT coronary angiography, nuclear myocardial imaging and coronary angiography in the diagnosis of coronary heart disease | Title-abstract exclusion |
| 1520 | Su Lei | 2010 | 64-row spiral CT hepatic arterial vascular imaging and DSA control study of primary liver cancer | Title-abstract exclusion |
| 1521 | Su Zengcun | 2010 | Study on the diagnostic value of 64-slice spiral CT coronary artery imaging on coronary artery in-stent restenosis | Title-abstract exclusion |
| 1522 | Sun Chao  Xue Meng  Yan Zhenhong  Yang Qian  Chang Ting | 2023 | Application of virtual reality combined with simulator teaching mode in lumbar puncture skill training | Title-abstract exclusion |
| 1523 | Sun Dandan | 2011 | Experimental study on the characteristics of spectral changes of blood flow velocity in the superior vena cava in pulmonary arterial hypertension | Title-abstract exclusion |
| 1524 | Sun Guochen  Chen Xiaolei  Hou Yuanzheng  Liu Lei  Zhang Jiasu  Tang Hao  Li Chong  Wang Peng  Xu Xinghua | 2017 | Endoscopic-assisted removal of supratentorial spontaneous intracerebral hematoma based on simple virtual reality and augmented reality localization | Title-abstract exclusion |
| 1525 | Sun Xuelin  Jian Cui  Lulu Qian  Hua Bin  Hua Zhen  Sun Mengnan  Xiao Qian | 2023 | Application of immersive static travel system in patients undergoing breast biopsy surgery | Title-abstract exclusion |
| 1526 | Sun Zhenkui | 2012 | Clinical Study of 3D-TOF-MRA Diagnosis of Intracranial Ruptured Aneurysm and Willis Overlay Stent Treatment | Title-abstract exclusion |
| 1527 | Qin Mingan  Ho Yui Lam  Lu Ping Fung | 2016 | Application of DSA 3DCT Exposure Mode in Percutaneous Puncture Ovarian Hole Localization Technique | Title-abstract exclusion |
| 1528 | Tan Hongna  Benny Yang  Wang Shengping  Peng Weijun  Gui Wu  Gu Yajia  Wu Jian  Qian Min  Hu Xiaoxin | 2010 | Feasibility analysis of CT lymphangiography of anterior sentinel lymph nodes in breast cancer | Title-abstract exclusion |
| 1529 | Tan Yu | 2010 | The value of 64-slice CT simulation vascular endoscopy technology to evaluate coronary heart disease | Title-abstract exclusion |
| 1530 | Tang Chenqiao | 2022 | Application study of virtual reality technology combined with flipped classroom in endovascular fistula puncture training for junior nurses | Title-abstract exclusion |
| 1531 | Tao Min  Zhu Hongjuan  Wu Chunyan | 2021 | Application of virtual reality technology in patients with P ICC placement in hematology department | Title-abstract exclusion |
| 1532 | Tong Jing  Chu Chengchen  Li Bin | 2023 | Research progress of vascular interventional surgery robot and its force feedback technology | Title-abstract exclusion |
| 1533 | Tong Jun | 2017 | Analysis of 62 cases of spinal tuberculosis using spiral CT reconstruction technology | Title-abstract exclusion |
| 1534 | Wang Baotang  Liu Zhiyan  Deng Daming | 2014 | Comparison of imaging diagnostic methods for ureteral lesions | Title-abstract exclusion |
| 1535 | Wang Bin | 2017 | Mechanical characterization and physical modeling of real vascular tissues | Title-abstract exclusion |
| 1536 | Wang Daojing | 2013 | A Controlled Study of 2D-DSA, 3D-DSA and CTA in the Diagnosis of Intracranial Aneurysms | Title-abstract exclusion |
| 1537 | Wang Dong | 2015 | Clinical application value of bronchial artery CTA in hemoptysis interventional therapy | Title-abstract exclusion |
| 1538 | Wang Fangxia  Liu Yuan  Zhang Wanggang  Cao Xingmei  Zhao Wanhong  Gu Liufang | 2012 | Research on virtual teaching training system for lumbar puncture surgery | Title-abstract exclusion |
| 1539 | Wang Gang  Zhang Yi  Na Yanqun  Yu Chengvan | 2011 | Application of virtual simulator in establishing percutaneous renal access skill training | Title-abstract exclusion |
| 1540 | Wang Haoyu | 2019 | Study on the measurement of pulmonary vein inlet diameter by different post-processing techniques of multislice spiral CT | Title-abstract exclusion |
| 1541 | Wang Jiaqiao | 2017 | Application of 320-row CT bronchial artery imaging in central lung cancer | Title-abstract exclusion |
| 1542 | Wang Junliang  Wei Junqiang  Han Tao  Hu Wenshan  Ma Rui  Tao Sheng | 2018 | The value of electronic knee simulator in knee puncture training  A case of hand trapezoidal hemangioma diagnosed by CTA | Title-abstract exclusion |
| 1543 | Wang Kaihua  Chen Lungang  Huang Yan | 2012 | A case of hand trapezoidal hemangioma diagnosed by CTA | Title-abstract exclusion |
| 1544 | Wang Lei  Zhang Yaonan  He Ying  Tang Liang  Zhao Jinlong | 2018 | Preliminary construction of SOFA-based virtual surgical system for jugular vein puncture | Title-abstract exclusion |
| 1545 | Wang Liyong | 2012 | A comparative study of 3.0T ultra-high field magnetic resonance angiography and DSA in the diagnosis of cerebral arterial stenosis | Title-abstract exclusion |
| 1546 | Wang Mei  Cao Hanbo  Le Hanbo  Wang Heping | 2016 | Diagnostic and differential diagnostic value of MSCT for isolated pulmonary nodule (≤2cm) with pleural recess sign | Title-abstract exclusion |
| 1547 | Wang Qiong  Shen Ning  Zhang Yongfu  Law Jianxin  Lai Guozhong  Xiao Lihuan  Chen Xiaojuan | 2008 | The effect of subarachnoid application of sufentanil on the respiratory function of patients undergoing laparoscopic surgery for ectopic pregnancy | Title-abstract exclusion |
| 1548 | Wang Shu | 2014 | Application of low concentration iodixanol combined with low tube voltage in CT angiography of lower extremity atherosclerotic occlusive disease | Title-abstract exclusion |
| 1549 | Wang Wei  Zhang Zhi  Wang Yanhua | 2024 | Application of CT portal and hepatic vein three-dimensional reconstruction imaging in transjugular intrahepatic portosystemic shunt surgery | Title-abstract exclusion |
| 1550 | Wang Xiong | 2016 | Application of double-low CT one-stop direct method in pulmonary embolism and lower extremity deep vein thrombosis | Title-abstract exclusion |
| 1551 | Wang Yazhou | 2010 | Clinical value of multislice spiral CT imaging and post-processing technology in lung cancer diagnosis | Title-abstract exclusion |
| 1552 | Wang Yanjie | 2023 | Construction and application of situational simulation teaching program for basic nursing practical training course for undergraduate nursing students based on INACSL simulation best practice standards | Title-abstract exclusion |
| 1553 | Wang Wei | 2009 | Research on key technology of collision detection in virtual reality | Title-abstract exclusion |
| 1554 | Wei Yini | 2022 | Design and realization of virtual injection simulation system with force feedback | Title-abstract exclusion |
| 1555 | Wen Xingqiao  Zhu Wei'an  Yu Wang  Li Tengcheng  Lai Wenjie  Leng Zu  Han Yuefu  Liu Lilin  Teng Dongdong  Gaoxin | 2020 | Application of DVPV system three-dimensional image and virtual reality navigation in urology complex surgery | Title-abstract exclusion |
| 1556 | Wu Chengyuan  Liu Base  Liu Yuguang  Meng Fangang | 2011 | VR technology guided radiofrequency treatment of trigeminal neuralgia | Title-abstract exclusion |
| 1557 | Wu Dongbo | 2009 | Research on the application of medical three-dimensional visualization and virtual reality technology in laparoscopic surgery for hepatocellular carcinoma | Title-abstract exclusion |
| 1558 | Wu Haolei | 2021 | VR-oriented research on force feedback and deformation of soft tissue needle puncture | Title-abstract exclusion |
| 1559 | Wu Xuebin  Kang Qiang  Zeng Shengtian  Pang Yuen Keung  Weng Zhipong  Chen Hong  Lin Xiangyan | 2018 | Application of 3D-Slicer combined with Sina software in neuroendoscopic surgery for hypertensive cerebral hemorrhage | Title-abstract exclusion |
| 1560 | Wu Xuebin  Kang Qiang  Li Min  Zeng Shengtian  Pang Yuen Keung  Weng Zhipeng  Chen Hong | 2018 | The efficacy of 3D-Slicer combined with sina software in assisting minimally invasive neuroendoscopic surgery for hypertensive cerebral hemorrhage | Title-abstract exclusion |
| 1561 | Wu Qiang  Han Zhiqiang  Zhao Jianchun  Pang Yi-Qiang  Zhang Haitao  Tian Weixia  Lan Xiaoyan  Hao Chenyang | 2024 | Analysis of the efficacy of intraoperative VR-assisted neuroendoscopic hematoma removal and stereotactic intracranial hematoma drainage for the treatment of HICH in the basal ganglia region | Title-abstract exclusion |
| 1562 | Xiao Fan  Liu Fang | 2022 | Mixed Reality Navigation Surgical System Helps Tumor Precision Ablation | Title-abstract exclusion |
| 1563 | Xiao Jingjing | 2010 | Diagnostic study of extrahepatic cholangiocarcinoma by 64-row spiral CT | Title-abstract exclusion |
| 1564 | Xie Guoqiang  Guo Zhenyu  Shi Wei  Zuo Yi  Xiao Sanchao  Lei Zhenhai  Chen Shangjun  Hao Wuji | 2017 | Application of low-cost augmented reality technology in neuroendoscopic treatment of hypertensive cerebral hemorrhage | Title-abstract exclusion |
| 1565 | Xie Guoqiang  Shi Wei  Chen Shangjun  Li Baoming  Huijun  Zhang Bin  Wu Dongfei  Ma Jingku | 2017 | Application value of 3D-slicer software in minimally invasive neuroendoscopic surgical treatment of hypertensive cerebral hemorrhage | Title-abstract exclusion |
| 1566 | Xin Ning | 2023 | Research on the localization method of lung nodule based on mixed reality guidance | Title-abstract exclusion |
| 1567 | Xu Bo  Chen Hui | 2010 | Experimental research on simulation training system of brain puncture surgery | Title-abstract exclusion |
| 1568 | Xu Bo  Chen Hui | 2010 | Research on the Application of Computer Technology in Simulating Neurosurgery Surgeries | Title-abstract exclusion |
| 1569 | Xu Bo  Chen Hui | 2010 | Experimental research on simulation training system of brain puncture surgery | Title-abstract exclusion |
| 1570 | Xu Lang | 2019 | Research on real-time deformation simulation method and application in virtual surgery system | Title-abstract exclusion |
| 1571 | Xu Wei  Shi Meng  Liu Jiangang  Wang Chenglong | 2010 | Expression of Collagen in Ischemic Myocardium of Rats after Acute Myocardial Infarction and the Intervention Effects of Traditional Chinese Medicines for Benefiting Qi, Nourishing Yin and Activating Blood and Detoxifying and Activating Blood in Rats | Title-abstract exclusion |
| 1572 | Xu Xinfeng  Gao Zhengrong  Jun Zhang  Luo Bin  Yao Lidong | 2017 | Study on the diagnostic value of 128-slice CT three-dimensional reorganization technology for solid lung nodules | Title-abstract exclusion |
| 1573 | Felicia Xu  Wei Xing  Nandi Bao | 2024 | Application of mixed-reality holographic imaging technology in the teaching of various common clinical puncture operations | Title-abstract exclusion |
| 1574 | Yan Xu  Zhu Jiemin  Dong Zhi  Li Yujie  Fu Donghai | 2006 | Application of electron-beam CT low-dose scanning for screening early lung cancer in patients with coronary CT imaging | Title-abstract exclusion |
| 1575 | Yan Xiaochen | 2023 | Design and application of VR system for teaching ultrasound-guided puncture surgery | Title-abstract exclusion |
| 1576 | Yan Yinying  He Xiaoxia | 2013 | The effect of psychological intervention on peripheral venous placement of central venous catheter puncture in chemotherapy patients | Title-abstract exclusion |
| 1577 | Yang Fan  Tian Xinli  Hu Xin | 2015 | Clinical value of MSCT in evaluating craniofacial bone fiber dysplasia | Title-abstract exclusion |
| 1578 | Yang Guang | 2013 | The value of Philips iCT bronchial artery CTA for lymph node dissection in esophageal cancer | Title-abstract exclusion |
| 1579 | Yang Jinfang  Xu Huili  Zhou Yumei  Wang Haiping  Nina Luo  Tang Yuting | 2022 | Application of virtual reality technology in pain management of PICC placement in children | Title-abstract exclusion |
| 1580 | Rui Yang  Lu Jinfang  Li Yonghua  Jiang Xin  Yuan Hongbin | 2024 | Application and evaluation of virtual reality-based scenario simulation teaching for subarachnoid puncture anesthesia in standardized residency training | Title-abstract exclusion |
| 1581 | Rui Yang  Lu Jinfang  Li Yonghua  Yuan Hongbin | 2024 | Application of virtual reality-based scenario simulation teaching in the teaching of lumbar-rigid combined anesthesia for standardized training of anesthesia residents. | Title-abstract exclusion |
| 1582 | Yang Yi  Liu Fuhe  Bai Yan  Wang Rongfeng  Chen Zhengguang | 2012 | A preliminary study of the technique of dialysis autologous arteriovenous fistula stenosis imaging by 64-row spiral CT | Title-abstract exclusion |
| 1583 | Yang Zhenxiang | 2011 | Research on the virtual platform of brain puncture and drainage surgery | Title-abstract exclusion |
| 1584 | YIU CHUN HA  Yee Kin Wah  Zhao Lunhua | 2005 | A case of lung mucosa-associated tissue lymphoma combined with gastric cancer | Title-abstract exclusion |
| 1585 | You Jian | 2005 | Clinical anatomy study of percutaneous lumbar disc puncture approach | Title-abstract exclusion |
| 1586 | Yu Hao | 2012 | The application value of dual-source spiral CT in the diagnosis of pediatric cyanosis-type complex congenital heart disease | Title-abstract exclusion |
| 1587 | Yu Ganglong  Zhang Fuzhou | 2014 | Diagnostic value of 16-row spiral CT three-dimensional reconstruction for peripheral small lung cancer | Title-abstract exclusion |
| 1588 | Yu Hui  Zhang Jun | 2021 | Application of 128-slice spiral CT VR image fusion reconstruction technology in preoperative evaluation of precise liver resection | Title-abstract exclusion |
| 1589 | Yu Qi  Feng Lijuan | 2023 | Application of virtual reality technology combined with advance local infiltration anesthesia in PICC placement in school-age children | Title-abstract exclusion |
| 1590 | Yu Kanghui | 2012 | Application of dual-source CT pulmonary vein imaging in radiofrequency ablation of atrial fibrillation | Title-abstract exclusion |
| 1591 | Yumeljiang Balati  He Pengyi | 2023 | Research progress of sST2 in ventricular remodeling after acute myocardial infarction | Title-abstract exclusion |
| 1592 | Yue Wei  Yuan Hong  Mao Xiaorong  Deng Yongdong  Chen Lin | 2013 | Efficacy of different strategies in the treatment of HBeAg-positive chronic hepatitis B with rtN236T locus variant | Title-abstract exclusion |
| 1593 | Yue Wenjun | 2007 | Study on the correlation between CT three-dimensional reconstruction of lung tumor vasculature and CD34-labeled microvessel density | Title-abstract exclusion |
| 1594 | Zhang Chengshun  Cheng Shui Ling | 2016 | Study on the relationship between neutrophil/lymphocyte ratio and ventricular remodeling after percutaneous transluminal coronary intervention in patients with ST-segment elevation myocardial infarction | Title-abstract exclusion |
| 1595 | Zhang Hao  Liu Qianqian  Gao Ling | 2014 | Construction of nursing practice teaching based on virtual reality technology | Title-abstract exclusion |
| 1596 | Zhang Hao | 2007 | CT Detection and Clinical Research on Pulmonary Vascularization and Pulmonary Blood Flow Status | Title-abstract exclusion |
| 1597 | Zhang Jiyu | 2022 | Research on the development, preliminary validation and application of endoscopic therapeutic technique trainer for simulated gastric modeling | Title-abstract exclusion |
| 1598 | Zhangjiazhu  Chen Xiaolei  Wang Qun  Hou Yuanzheng  Sun Guochen  Li Fangye  Zhang Zhizhong  Zhang Jun  Sun Zhenghui  Yu Xinguang  Xu Bainan | 2017 | Application of virtual and augmented reality technology in teaching intracranial lesion puncture surgery | Title-abstract exclusion |
| 1599 | Zhangjiazhu  Qu Ling  Wang Qun  Gui Qiuping  Hou Yuanzheng  Sun Guochen  Li Fangye  Zhang Zhizhong  Chen Xiaolei  Zhang Jun  Sun Zhenghui  Yu Xinguang  Xu Bainan | 2018 | Application of Virtual Reality Technology Based on Multimodal Navigation in Puncture Surgery for Lesions in Functional Areas of the Brain | Title-abstract exclusion |
| 1600 | Zhang Jianjun | 2014 | Application study of 64-slice CT angiography in the diagnosis of pulmonary artery embolism | Title-abstract exclusion |
| 1601 | Zhang Kongyuan  Ding Ning  Feng Wang  Zhang Xiaoyi | 2021 | Application value of three-dimensional digital surgical navigation in lung nodule puncture biopsy | Title-abstract exclusion |
| 1602 | Zhang Liangjian  Zhang Guangchao  Xu Haiping  Li Yanxiang | 2009 | Clinical observation on the treatment of malignant pleural fluid by microtubular drainage infusion of interleukin-2 interspersed with infusion of DDP | Title-abstract exclusion |
| 1603 | Zhang Lin  He Lijun  Liu Saisai  Hao Yonghui  Zhang Hongmei | 2020 | Prospective application of virtual reality technology in the field of blood purification | Title-abstract exclusion |
| 1604 | Zhang Ling  Chen Chen  James Zou | 2012 | Research on the application of digital simulation system in the clinical demonstration of thyroid nodule puncture based on modern educational technology | Title-abstract exclusion |
| 1605 | Zhang Pin | 2020 | Research on Precise Preoperative Planning Technology Based on Augmented Reality | Title-abstract exclusion |
| 1606 | Zhang Qiang | 2020 | Soft Tissue Modeling and Its Application in Virtual Surgical System | Title-abstract exclusion |
| 1607 | Qiongyue Zhang  Julia Zhang  Linlin Jiang  Tangjing Wu  Zongze Zhang  Wang Yanlin | 2022 | Role of intraperitoneal resuscitation with pyruvate peritoneal dialysis solution on ischemia-reperfusion injury of rat kidney | Title-abstract exclusion |
| 1608 | Zhang Shike  Guan Tianming | 2009 | Diagnostic value of 64-slice spiral CT on the nature of breast masses | Title-abstract exclusion |
| 1609 | Zhang Tong | 2021 | Analysis of the clinical application value of mixed reality technology in PCNL management of complex kidney stones | Title-abstract exclusion |
| 1610 | Zhang Xieke  Luo Wen  Zhao Yan  Han Chunzhi  Yang Ming  Zhuang Xiaoming  He Yueming | 2011 | Application of RadioDexter system in teaching basic clinical operation skills | Title-abstract exclusion |
| 1611 | Zhang Xiaoli  Gao Xiaopei  Li Juan  Seed Liting  Chen Hua  Wu Wei  Wang Gang  Li Lianyong | 2023 | Design and application of intravenous infusion training system based on virtual reality technology | Title-abstract exclusion |
| 1612 | Zhang Xin | 2014 | Application study of CT three-dimensional reconstruction of renal collecting system anatomy for PCNL preoperative puncture localization | Title-abstract exclusion |
| 1613 | Zhang Xiulan  Wang Dong  Fan Hongxia  Qian Yong  Bi Yongmin | 2015 | Multi-slice spiral CT presentation and pathologic control of isolated fibrous tumor of pleura | Title-abstract exclusion |
| 1614 | Zhang Xu | 2010 | The value of MSCT image post-processing in showing the structure of pterygopalatine fossa | Title-abstract exclusion |
| 1615 | Zhang Yaping | 2008 | Clinical Application of Time-Resolved 3D Dynamically Enhanced 3.0T MR Angiography | Title-abstract exclusion |
| 1616 | Zhang Yang  Chen Fenghua  Liang Tao  Gao Ziwei  Hu Lihua  Wang Lin | 2023 | Construction of learning platform for bone marrow cytology examination based on virtual simulation laboratory teaching system | Title-abstract exclusion |
| 1617 | Zhang Yi | 2007 | Study on infiltration and growth characteristics of human breast cancer by digital virtual three-dimensional reconstruction and whole breast macroslice | Title-abstract exclusion |
| 1618 | Zhang Yunhao  He Lingmin  Sun Xu  Ma Honggui  Liu Lei  Zhang Jianrong  Mei Aobing | 2024 | Study on the application of CT-based three-dimensional reconstruction model and preoperative virtual surgery in laparoscopic surgery for ureteral stenosis | Title-abstract exclusion |
| 1619 | Zhang Ziqin | 2011 | Diagnosis of Biliary Obstructive Disease by CT Scanning with Percutaneous Hepatic Puncture Cholangiography | Title-abstract exclusion |
| 1620 | Zhao Li  Sun Zheng  Jie Shang  Zhang Yue Zheng  Ma Suwen  Lu Jie | 2020 | Study on the effect of two indwelling needle puncture angles with different contrast injection rates in reducing contrast extravasation | Title-abstract exclusion |
| 1621 | Zhao Xiaodan | 2017 | Accuracy of dual-source CT dual-energy scanning for diagnosing stenosis due to calcified plaque in coronary arteries | Title-abstract exclusion |
| 1622 | Zheng Choshun  Li Chunhai  Zeng Gang  Ye Wei  Sun Jianchao  Hong Junmin | 2018 | Methods and significance of constructing virtual reality model of lumbar spine degeneration | Title-abstract exclusion |
| 1623 | Zheng Shaoli  Li Zongze  Yao Xinqiang  Zhang Jinyuan  Chu Wing-kin  Chan Kin Ting | 2019 | Current status of virtual reality technology in spine surgery | Title-abstract exclusion |
| 1624 | Zheng Shilin  Lai Xueqin  Li Zhong | 2014 | The value of 64-row CT angiography in evaluating ureterocutaneous fistula vessels | Title-abstract exclusion |
| 1625 | Zheng Shilin  Tan Yiqing  Jiang Shengpan  Zeng Ting | 2018 | Application of transcapillary venous balloon dilatation in hemodialysis autologous arteriovenous endovascular stenosis | Title-abstract exclusion |
| 1626 | Zheng Boat  Xie Huihong  Gao Fei  Gong Cansheng  Ye Peng | 2023 | Development and application of teaching platform of lumbar hard and hard joint block based on virtual reality and haptic feedback technology | Title-abstract exclusion |
| 1627 | Zhong Yi  Lieven  Bu Wenjun  Zhang Junhua | 2021 | Application effect of virtual reality technology in teaching training and clinical practice of laparoscopic surgery %J International Medical and Health Herald | Title-abstract exclusion |
| 1628 | Zhou Jianwei  Li Spear  Chi Cheng  Yu Panfeng  Wang Fei  Tang Jiaguang | 2020 | Preliminary exploration and application of percutaneous endoscopic lumbar discectomy guided by mixed reality technology | Title-abstract exclusion |
| 1629 | Zhou Peng  Zheng Xiaohua  Ren Jing  Hu Shibei  Xia Qian  Luo Hongbing  Xu Guohui | 2013 | Application of 64-slice spiral CT vascular fusion imaging in recurrent malignant tumors of the chest and abdominal wall | Title-abstract exclusion |
| 1630 | Zhou Wenjin | 2020 | Research on bio-tissue mechanics modeling algorithm for compression and puncture | Title-abstract exclusion |
| 1631 | Zhu Aiguo  He Yusheng  Zhang Jinsong | 2013 | Application of DSA three-dimensional reconstruction technology in the interventional diagnosis and treatment of cavernous sinus fistula of intracranial artery | Title-abstract exclusion |
| 1632 | Zhu Jin  Liu Te  Ling Rennan  Gong Jingshan  Xu Jianmin | 2011 | Application value of CTA showing perigastric artery and its variations in preoperative evaluation of gastric cancer | Title-abstract exclusion |
| 1633 | Zhu Pengcheng | 2006 | Research and Improvement of GJK Collision Detection Algorithm | Title-abstract exclusion |
| 1634 | Zhu Zhaoju  Gao Chuhang  Shi Jiafeng  Chen Liujing  Liu Yuqing  He Bingwei | 2022 | Force feedback simulation of lateral ventriculostomy | Title-abstract exclusion |
| 1635 | Bamboo Bun Yang  Perry Liu  Di Xin Xin  Gao Xin Yu  Tian Jiawei  Jiang Shuangquan | 2024 | Exploration of the application of simulation teaching method in interventional ultrasound diagnosis and treatment training | Title-abstract exclusion |
| 1636 | Zu Chao | 2009 | Research on the key technology of force feedback based on deformation model in virtual surgery simulation | Title-abstract exclusion |
| 1637 | Zuo Hui | 2022 | Research on robot-assisted brain hematoma removal and trajectory planning based on augmented reality | Title-abstract exclusion |
| 1638 | Guo Yanjie  Zhang Xuemei  Dong Xue | 2024 | Research Progress of Virtual Reality Technology in Reducing the Pain of Venipuncture in Children in Emergency Department | Title-abstract exclusion |
| 1639 | Song Yanfei  Wang Tongju  Wang Jiange  Yang Jipeng  Sun Guozhu | 2024 | Evaluation of the efficacy of 3D printing apparatus based on virtual reality technology in assisting puncture and drainage in treating hypertensive cerebral hemorrhage and its role in doctor-patient communication | Title-abstract exclusion |
| 1640 | Sun Chao  Xue Meng  Yan Zhenhong  Yang Qian  Chang Ting | 2023 | Application of virtual reality and simulator teaching mode in lumbar puncture skill training | Title-abstract exclusion |
| 1641 | Yang Yiling  Wei Chunyan  Pei Yanling | 2023 | Progress in the application of virtual reality technology in the management of pain associated with venipuncture in children | Title-abstract exclusion |
| 1642 | Zhao Hongxia  Lv Xuerui  Chen Yang  Wang Yanfu | 2021 | Meta-analysis of virtual reality therapy on reducing venipuncture pain in children | Title-abstract exclusion |
| 1643 | Zhao Hongxia  Lv Xuerui  Chen Yang  Wang Yanfu | 2021 | Meta-analysis of the effect of virtual reality technology on reducing puncture-related pain and anxiety in pediatric nursing care | Title-abstract exclusion |
| 1644 | Fan Hongwei  Wen-Ting Hu  Zhang Ying  Zhou Hui  Wang Hui  Yuan Zitong | 2021 | Design and application of virtual training system for bone marrow aspiration based on VR technology | Title-abstract exclusion |
| 1645 | Liu Tao  Niu Guoqi  Chen Hui  Liu Lutan  Li Chao | 2021 | Application effect of 3D printing combined with VR technology in teaching percutaneous pedicle puncture | Title-abstract exclusion |
| 1646 | Ding Jing  Liu Jing  Xiao Yihan  Zeng Chaomei | 2020 | Application of virtual reality lumbar puncture training system in pediatric skills training for eight-year medical students | Title-abstract exclusion |
| 1647 | Li Yu  Liu Gang  Wang Xiaorong  Yang Yang  Duan Ran | 2020 | Application of automatic evaluation system of thoracocentesis in teaching based on VR interactive technology | Title-abstract exclusion |
| 1648 | Hong Wenyao  Liu Yuqing  He Bingwei  Huang Jinyue  Liao Zhengjian  Chen Shou | 2018 | Research on simulation training of lateral ventricle puncture based on virtual reality technology | Title-abstract exclusion |
| 1649 | Zhangjiazhu  Qu Ling  Wang Qun  Gui Qiuping  Hou Yuanzheng  Sun Guochen  Li Fangye  Zhang Zhizhong  Chen Xiaolei  Zhang Jun  Sun Zhenghui  Yu Xinguang  Xu Bainan | 2018 | Application of virtual reality technology based on multimodal navigation in brain functional area lesion puncture surgery | Title-abstract exclusion |
| 1650 | Lv Liushuai  Wang Guangzhi  Huang Yibin  Wu Jian | 2016 | Application of virtual reality display system in ultrasound-guided puncture surgery | Title-abstract exclusion |
| 1651 | Sangjae Seo  Kim Mungi | 2010 | Development of Medical Needle Insertion Simulator Using a Haptic Device | Title-abstract exclusion |
| 1652 | Han Qing | 2023 | Application and Research of Virtual Reality-Based Diagnostic System for Abdominal Fluid Accumulation | Title-abstract exclusion |
| 1653 | He Wei | 2023 | Application of local radial basis function collocation method to puncture problem based on potential problem | Title-abstract exclusion |
| 1654 | Toraman RL  Eskici Ilgin V | 2024 | The Effect of Virtual Reality Glasses Application on Pain, Anxiety, and Patient Satisfaction | Included |
| 1655 | Melcer Y, Maymon R  Gal-Kochav M  Pekar-Zlotin M  Levinsohn-Tavor O  Meizner I  Svirsky R | 2021 | Analgesic efficacy of virtual reality for acute pain in amniocentesis: A randomized controlled trial | Woring population |
