# Supplementary material for: The efficacy of virtual reality in adults during puncture biopsy: A systematic review and meta-analysis of randomized controlled trials
Source: PLoS One. 2025 Aug 26;20(8):e0330364. doi: 10.1371/journal.pone.0330364 (PMC12380292; doi:10.1371/journal.pone.0330364)
Supplement: S5 Table — (DOCX) [file pone.0330364.s005.docx]

**S5 Table. Quality and Certainty of Evidence Assessment**

| **Study** | **Random sequence generation** | **Allocation concealment** | **Blinding of participants and personnel (performance bias)** | **Blinding of outcome assessors** | **Incomplete outcome** | **Selective outcome reporting** | **Other risks of bias** |
| --- | --- | --- | --- | --- | --- | --- | --- |
| 1.Genç et al | Low | Unclear ^a^ | High ^b^ | High^b^ | Unclear ^c^ | Low | Low |
| 2.Karaman et al | Low | Unclear ^a^ | High ^b^ | High ^b^ | Unclear ^c^ | Low | Low |
| 3.Korkmaz et al | Low | Unclear ^a^ | High ^b^ | High ^b^ | Unclear ^c^ | Low | Low |
| 4.Le Du et al | Low | Unclear ^a^ | High ^b^ | High ^b^ | Unclear ^c^ | Low | Low |
| 5.Prabhu et al | Low | Unclear ^a^ | High ^b^ | High ^b^ | Unclear ^c^ | Low | Low |
| 6.Toraman et al | Low | Unclear ^a^ | High ^b^ | High ^b^ | Unclear ^c^ | Low | Low |

a：This is due to the authors' lack of sufficient information regarding allocation concealment, a topic not addressed in the article. Consequently, a judgment cannot be made regarding the risk of it.

b: Blinding of patients or personnel during VR applications is very difficult. Therefore, blinding of patients or personnel was not achieved in any of the included studies.

c: As the available information regarding the registration network was incomplete, a detailed comparison of the content of the article and the registration program to assess the risk could not be conducted.
