# Supplementary material for: The efficacy of virtual reality in adults during puncture biopsy: A systematic review and meta-analysis of randomized controlled trials
Source: PLoS One. 2025 Aug 26;20(8):e0330364. doi: 10.1371/journal.pone.0330364 (PMC12380292; doi:10.1371/journal.pone.0330364)
Supplement: S6 Table — (DOCX) [file pone.0330364.s006.docx]

**S6 Table. Summary of GRADE evidence profile**

| Outcome name | Included studies | Design | Effect size  95% CI | Heterogeneity | Patients in VR | Patients in control | Risk of bias | Inconsistency | Indirectness | Imprecision | Publication  bias | Quality | Importance |
| --- | --- | --- | --- | --- | --- | --- | --- | --- | --- | --- | --- | --- | --- |
| Pain score | 6 | RCTs | MD = -1.61; (-2.54, -0.68) | *I^2^* = 90% | 219 | 226 | Serious^a^ | Serious^b^ | Not Serious | Not Serious | Undetected | Low | Critical |
| Anxiety score | 3 | RCTs | MD = -9.49;  (-14.47, -4.50) | *I^2^* = 88% | 114 | 118 | Serious^a^ | Serious^b^ | Not Serious | Not Serious | Undetected | Low | Important |

RCTs, randomized control trials;CI, confidence interval;VR, virtual reality

^a^As the included studies showed higher risk of bias especially with blinding of participants, personnel, and outcome assessors besides other bias

^b^Substantial heterogeneity (*I^2^*=90%) and (*I^2^*=88%) was found
