# Supplementary figures and images for: The efficacy of virtual reality in adults during puncture biopsy: A systematic review and meta-analysis of randomized controlled trials
Source: PLoS One. 2025 Aug 26;20(8):e0330364. doi: 10.1371/journal.pone.0330364 (PMC12380292; doi:10.1371/journal.pone.0330364)

**S7 File. Egger's test**


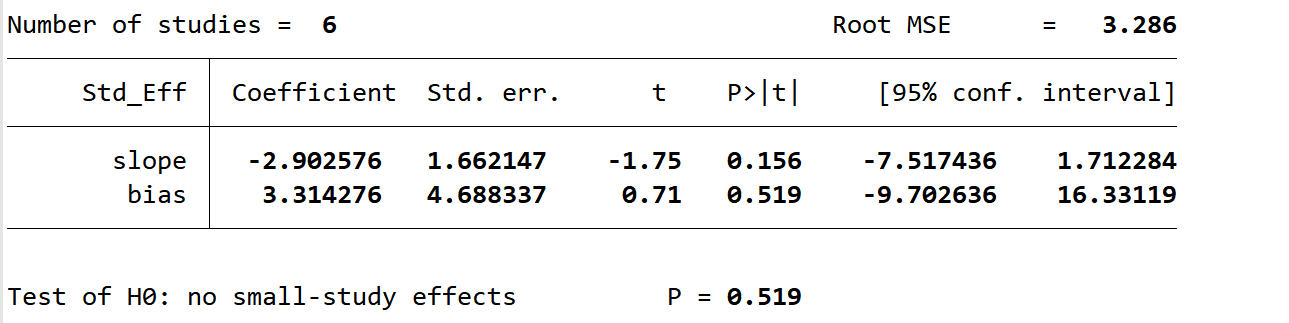

Supplement: S1 File — (DOCX) [file pone.0330364.s007.docx]
